# Unveiling the dynamics of acetylation and phosphorylation in SGBS and 3T3-L1 adipogenesis

**DOI:** 10.1016/j.isci.2024.109711

**Published:** 2024-04-10

**Authors:** Alix Sarah Aldehoff, Isabel Karkossa, Cornelius Goerdeler, Laura Krieg, Jana Schor, Beatrice Engelmann, Martin Wabitsch, Kathrin Landgraf, Jörg Hackermüller, Antje Körner, Ulrike Rolle-Kampczyk, Kristin Schubert, Martin von Bergen

**Affiliations:** 1 Department of Molecular Toxicology, Helmholtz-Centre for Environmental Research GmbH (UFZ), Leipzig, Germany; 2 Department of Computational Biology and Chemistry, Helmholtz-Centre for Environmental Research GmbH (UFZ), Leipzig, Germany; 3 Division of Pediatric Endocrinology and Diabetes, University Hospital for Children and Adolescents Ulm, Ulm, Germany; 4 University Hospital for Children and Adolescents, Center for Pediatric Research, Medical Faculty, University of Leipzig, Leipzig, Germany; 5 Helmholtz Institute for Metabolic Obesity and Vascular Research (HI-MAG) of the Helmholtz-Centre Munich at the University of Leipzig and University Hospital Leipzig, Leipzig, Germany; 6 LIFE–Leipzig Research Center for Civilization Diseases, Medical Faculty, University of Leipzig, Leipzig, Germany; 7 Department of Computer Science, University of Leipzig, Leipzig, Germany; 8 Institute of Biochemistry, Faculty of Biosciences, Pharmacy and Psychology, University of Leipzig, Leipzig, Germany; 9 German Centre for Integrative Biodiversity Research (iDiv) Halle-Jena-Leipzig, Leipzig, Germany

**Keywords:** Biological sciences, Molecular network, Proteomics, Metabolomics, Transcriptomics

## Abstract

Obesity, characterized by enlarged and dysfunctional adipose tissue, is among today’s most pressing global public health challenges with continuously increasing prevalence. Despite the importance of post-translational protein modifications (PTMs) in cellular signaling, knowledge of their impact on adipogenesis remains limited. Here, we studied the temporal dynamics of transcriptome, proteome, central carbon metabolites, and the acetyl- and phosphoproteome during adipogenesis using LC-MS/MS combined with PTM enrichment strategies on human (SGBS) and mouse (3T3-L1) adipocyte models. Both cell lines exhibited unique PTM profiles during adipogenesis, with acetylated proteins being enriched for central energy metabolism, while phosphorylated proteins related to insulin signaling and organization of cellular structures. As candidates with strong correlation to the adipogenesis timeline we identified CD44 and the acetylation sites FASN_K673 and IDH_K272. While results generally aligned between SGBS and 3T3-L1 cells, details appeared cell line specific. Our datasets on SGBS and 3T3-L1 adipogenesis dynamics are accessible for further mining.

## Highlights

• SGBS and 3T3-L1 cells show unique temporal acetyl- and phosphoproteomes during adipogenesis
• Acetylation dominates in core metabolism/phosphorylation relevant for structural organization
• Identification of potential key driver sites and proteins for the changes across adipogenesis
• General agreement between both adipocyte cell lines, with specificities in details

## Graphical abstract

**Figure:**
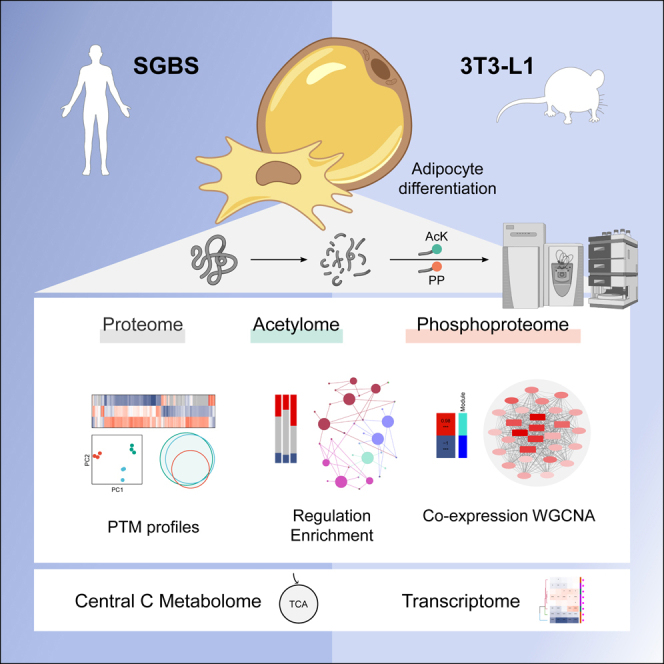

## Introduction

Obesity has emerged as one of the most pressing public health challenges at pandemic dimensions, often placing a significant burden on metabolic and cardiovascular health and hence life expectancy. Increased expansion of white adipose tissue (WAT) is a hallmark of obesity, but it is supposedly the fat depot distribution—visceral or subcutaneous (VAT/SAT) —and the degree of dysregulation that primarily determine the development of associated conditions such as insulin resistance, systemic inflammation, hypertension, and dyslipidaemia, collectively referred to as metabolic syndrome.^1^ While expansion by adipocyte hyperplasia (increase in cell number) in SAT is characteristic for adipose tissue from metabolically healthy individuals, adipocyte hypertrophy (increase in cell size) in VAT is common for those suffering from metabolic syndrome.^2^ The functions of healthy adipose tissue exceed the sole storage and release of triglycerides and fatty acids for the maintenance of energy homeostasis, and account for its role as important secretory endocrine organ of adipokines and metabolic substrates.^3^

Many of these functions are controlled at protein level, and their analysis at global scale by mass spectrometry-based proteomics or underpinning gene expression by transcriptomics has become an integral approach in obesity research.^4^^,^^5^^,^^6^ In addition, post-translational modifications (PTMs) can rapidly and dynamically alter protein structure, activity or interactions and their relevance for adipocyte biology has been extensively reviewed.^7^^,^^8^ Protein phosphorylation is the most well-studied PTM, described to modify >75% of all cellular proteins,^9^ with an essential role in metabolic regulation.^10^ Covalent addition of acetyl-CoA as lysine acetylation provides a direct link between cellular metabolism and signaling, and has emerged as vitally important PTM in the context of metabolism-associated diseases.^11^^,^^12^^,^^13^ Mediators (“writers” and “erasers”) of phosphorylation and acetylation are kinases/phosphatases and lysine acetyltransferases (KATs)/lysine deacetylases (KDACs), respectively. These enzymes are fundamentally involved in the healthy progression of adipose tissue expansion and functional cellular signaling. For instance, within the insulin signaling cascade, multiple phosphorylations of the insulin receptor substrate proteins IRS1 and IRS2 by several protein kinases, is dysregulated in metabolic diseases.^14^ In terms of acetylation, sirtuin 1 (SIRT1) as prominent nuclear KDAC, was found to repress the master regulator of adipogenesis PPARγ, thus promoting fat mobilization in 3T3-L1 adipocytes,^15^ while cytoplasmic SIRT2 interfered with acetylation of the transcription factor FOXO1 causing inhibited differentiation after overexpression and enhanced differentiation after SIRT2 knockdown.^16^ These trends were confirmed in visceral adipose stem cells (ASCs) from individuals with and without obesity that showed reduced SIRT1 and SIRT2 transcript and protein levels, and attenuated adipocyte differentiation after overexpression, whereas the opposite was true after knockdown of both sirtuins.^17^

Acetyl-CoA depicts the critical metabolic intermediate for *de novo* lipogenesis (DNL) and protein acetylation, present in distinct cellular pools. They are supplied by multiple sources including the oxidation of pyruvate from glycolysis, the β-oxidation of fatty acids, degradation of branched chain amino acids (BCAAs) (mitochondrial pool) or enzymatic conversion of citrate or acetate (cytosolic pool).^18^ The tricarboxylic acid (TCA) cycle is thereby central for providing acetyl-CoA, energy equivalents and metabolic building blocks in response to dynamic demands and thus, plays a pivotal role in the healthy expansion of WAT.^19^

To study adipogenesis, different biological matrices (e.g., cell lines, primary cells, tissue) are available and the results can differ considerably. While primary adipocytes exhibit a phenotype similar to physiological conditions of adipose tissue, they usually display pronounced donor and depot differences and are limited in availability and renewal capacity. In contrary, cell lines provide an unlimited source of homogeneous material with stable cellular properties, rendering them essential for the study of adipogenesis *in vitro*. Murine 3T3-L1 fibroblasts are the most commonly used model for adipocyte biology, applicable for the study of a wide range of research questions *in vitro.*^20^ However, over the past 20 years, Simpson-Golabi-Behmel Syndrome (SGBS) derived cells have emerged as the most widely used human adipocyte cell line.^21^ Both cell lines have been directly compared in regards of their lipogenic regulation,^22^ response to chronic insulin stimulation^23^ or for retention of crucial transcription factor binding sites,^24^ revealing species-specificities. On a global level, a comparison of the two cell lines is still pending.

Although not yet comprehensively compared, proteome and transcriptome of SGBS^25^ and 3T3-L1 cells^26^^,^^27^ have been analyzed separately, revealing the major molecular processes involved in adipocyte differentiation. Phosphorylation and acetylation as post-translational modifications with recognized roles in adipogenesis have by now only been studied as single modification and time point in one cell line^28^ and tissue^29^ or are lacking broad mass spectrometry based data.^30^ Nonetheless, a profound understanding of the role of PTMs in adipocyte development and physiology is a key to unveiling alternative therapeutic options for the treatment of obesity and associated conditions. To overcome these data gap, we provide a comprehensive resource of the SGBS transcriptome, follow the metabolites of the central carbon metabolism throughout adipogenesis, and provide an exhaustive analysis of the proteome, acetylome, and phosphoproteome in differentiating SGBS and 3T3-L1 adipocytes. With this approach, we aim to provide a detailed overview of the changes in protein acetylation and phosphorylation that are occurring during adipocyte differentiation in the two most commonly used adipocyte cell models. The assignment to relevant metabolic and regulatory pathways has the potential to support further functional analyses essential to perceive the formation of related diseases, and forms the basis for the identification of early indicators and therapeutic approaches.

## Results

### Proteins, metabolites, and transcripts display distinct temporal profiles during adipogenesis

**Figure 1:**
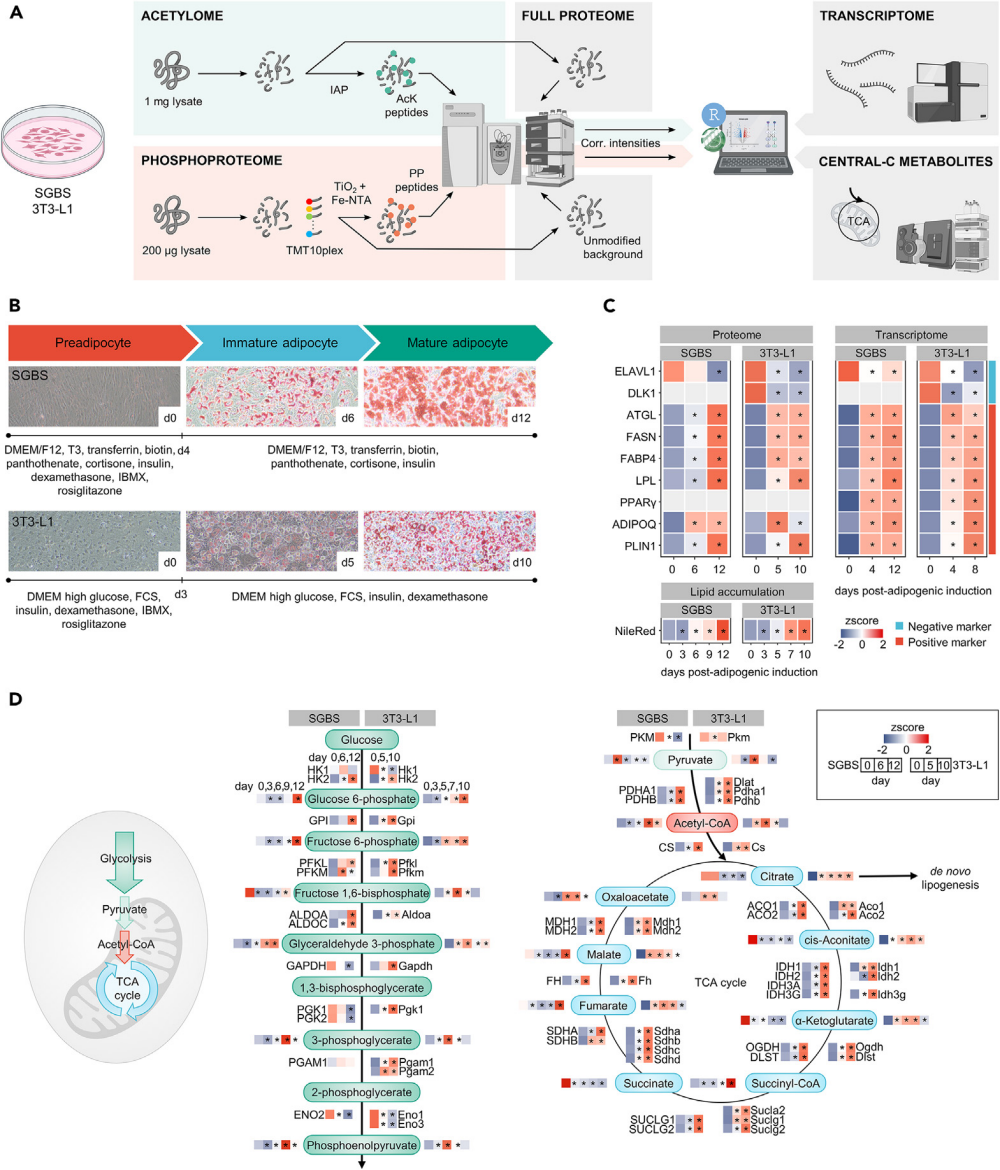
Workflow for the analysis of temporal dynamics in SGBS and 3T3-L1 adipocyte differentiation and comparison of protein, transcript and metabolite profiles (A) Experimental setup for the analysis of proteome, acetylome, and phosphoproteome, as well as the integration of central carbon metabolites and transcripts. The 3T3-L1 transcriptome data have been published previously.^26^ (B) Timelines of adipocyte differentiation in SGBS and 3T3-L1 cells and the days (d) of sample collection (d0/6/12 for SGBS; d0/5/10 for 3T3-L1) with information on differentiation efficiency as indicated by oil red O-stained lipid accumulation. Information about cell culture media composition is provided. (C) z-scores of established positive and negative adipogenesis markers in both cell lines and Nile Red quantified lipid accumulation to confirm differentiation. (D) Profiles of metabolites and proteins (z-scored) involved in glycolysis (cytosolic) and the TCA cycle (mitochondrial) for SGBS (left) and 3T3-L1 cells (right). Enzymes analyzed after d0/6/12 (SGBS) and d0/5/10 (3T3-L1); metabolites analyzed after d0/3/6/9/12 (SGBS) and d0/3/5/7/10 (3T3-L1). Significance compared to initiation of differentiation at d0 was calculated using a Student’s t test and is indicated as SD with asterisk corresponding to *p* < 0.05 ∗. Created with BioRender.com.

A profound understanding of the molecular mechanisms of adipocyte differentiation is needed to advance research on the development of obesity and associated metabolic disorders. To study molecular mechanisms, we used standard protocols for the differentiation of SGBS and 3T3-L1 preadipocytes into mature adipocytes to pursue proteome, transcriptome, central metabolites, and the constitutive profiles of lysine acetylation and phosphorylation during adipogenesis (Figures 1A and 1B). Differentiation efficiency of both cell lines was confirmed based on the accumulation of *de novo* synthesized lipids and molecular markers for adipocyte differentiation. The cells showed the typical increase in accumulated lipids toward adipocyte maturation, visualized by oil red O staining (Figure 1B) and quantified by Nile red fluorescence (Figure 1C).

To elucidate temporal patterns in gene expression, an RNA-seq dataset was generated in SGBS cells (Figures S1A–S1C). Additionally, a published 3T3-L1 dataset^26^ was considered for the comparison of both cell lines. In SGBS cells, differential expression over the course of adipocyte maturation was evident, with distinct clusters exhibiting expression peaks at early, late, or intermediate time points (Figures S1A and S1C). Furthermore, the transcriptome at the different time points displayed distinct separation with high similarity between replicates (Figure S1B).

In addition, protein abundance was analyzed using a temporally resolved quantitative proteomic approach, resulting in 3190 and 4655 reliably quantified proteins in differentiating SGBS and 3T3-L1 cells, respectively. A principal-component analysis (PCA) of the proteomic data displayed a clear separation between the different stages of adipocyte maturation (Figures S2A and S2B) and enriched for processes described to be relevant in adipogenesis in both cell lines (Figures S2C and S2D).^25^^,^^26^ Log2 fold changes across adipogenic differentiation showed a positive and significant correlation between protein and mRNA levels, indicating the molecules to be good surrogates of each other (Figure S1D). Adipocytes exhibited stage-specific markers for adipogenesis with consistency between proteomic and transcriptomic datasets and across species (Figure 1C). Negative markers of adipogenesis such as ELAV-like protein 1 (ELAVL1) or the mouse-specific delta-like homolog 1/preadipocyte factor 1 (DLK1/PREF1) showed high abundances in preadipocytes. In contrary, positive markers for adipogenesis such as fatty acid synthase (FASN), the adipocyte fatty acid-binding protein 4 (FABP4), adiponectin (ADIPOQ), adipose triglyceride and lipoprotein lipases (ATGL, LPL) and perilipin-1 (PLIN1) displayed an increasing abundance with maturation. The transcription factor PPARγ as master regulator of adipogenesis showed the expected increasing expression over the course of adipocyte differentiation in the RNA-seq data, while it was not detectable using the proteomics approach applied here.

*De novo* lipogenesis and the subsequent release of free fatty acids are key properties of WAT to maintain energy balance. Central to this are carbohydrate and lipid metabolism with the TCA cycle as pivot point of metabolic intermediates.^19^ We determined the levels of central carbon proteins and metabolites involved in glycolysis and the TCA cycle to elucidate their profiles across adipogenesis. For information about protein abundance we analyzed preadipocytes (d0, both cell lines), immature adipocytes (SGBS, d6; 3T3-L1, d5) and mature adipocytes (SGBS, d12; 3T3-L1, d10). Metabolites levels were determined for the differentiation stages mentioned, with addition of two more time points (SGBS, d3 and d9; 3T3-L1, d5, and d7).

Pyruvate as a product of cytoplasmic glycolysis can diffuse into the mitochondria in aerobic conditions. In the mitochondria, it undergoes oxidation and enters the TCA cycle as acetyl-CoA (Figure 1D). Apart from fructose-1,6-bisphosphate, glycolytic metabolites showed a similar profile during adipogenesis in both cell lines with highest abundances at later time points. Patterns of glycolysis enzymes appear more complex. They show the same increasing trend over time in both cell lines in upper glycolysis (until glyceraldehyde 3-phosphate), while opposing directionality in lower glycolysis in SGBS cells. Enolase (ENO1-3) catalyses the transformation of 2-phosphoglycerate into phosphoenolpyruvate and showed the same decreasing abundance alongside adipocyte maturation in both cell lines. Although not the only carbon sources of acetyl-CoA, glucose, and glutamine have been shown to account for the majority of lipogenic acetyl-CoA.^31^ Pyruvate, acetyl-CoA and the involved catalyzing enzymes show the same profiles over the time course with the highest abundance during an intermediate state of adipogenic differentiation in SGBS and 3T3-L1 cells. From citrate to fumarate, TCA intermediates showed diametrically opposing abundances in SGBS compared to 3T3-L1 adipocytes. Trends approached again toward malate. Finally, oxaloacetate is combined with acetyl-CoA and both metabolites were found with similar profiles across adipogenesis, indicating synchronized final replenishment of the TCA cycle independent of the cell line.

Enzymes of the TCA cycle appear in comparable profiles throughout the differentiation timeline between the two adipocyte models. Noteworthy, in SGBS cells metabolites and enzymes of the TCA cycle do not behave in synchrony, indicating a more complex picture of carbon metabolism and fatty acid synthesis.

### KATs and KDACs show complex expression profiles across adipogenesis in different adipocyte models

Besides its central role in energy metabolism, fatty acid and cholesterol synthesis, acetyl-CoA is the only substrate for protein acetylation,^32^ which is crucially involved in the regulation of adipogenesis and lipid metabolism.^11^ Modification levels are dynamically regulated by lysine acetyltransferases (KATs) and lysine deacetylases (KDACs) and display a direct link between the cells metabolic state and cellular signaling. KATs and KDACs are widely discussed for their involvement in metabolic diseases such as obesity and as interesting therapeutic targets for the treatment of them.^13^

**Figure 2:**
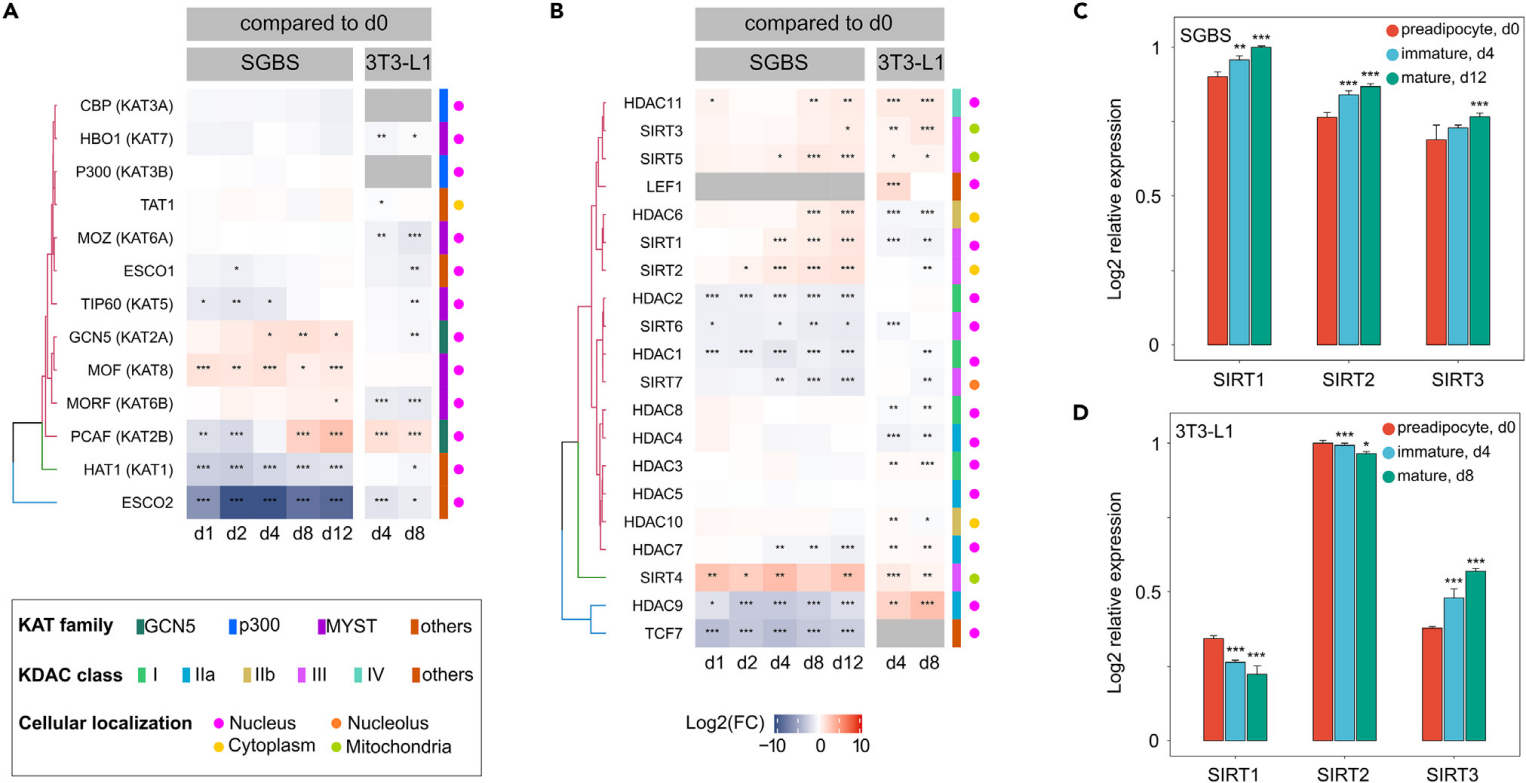
Dynamics of SGBS transcripts and KAT/KDAC expression across adipocyte differentiation (A) KAT and (B) KDAC expression in SGBS and 3T3-L1 cells based on average log2 fold changes compared to initiation of differentiation at day 0. KAT family/KDAC class affiliation and cellular localization indicated according to Narita et al.^77^. (C) Log2 relative expression of SIRT1, SIRT2 and SIRT3 transcripts in differentiating SGBS and (D) 3T3-L1 cells. Data are displayed as mean ± SD and as specified in the axis/panel legends. Significance was calculated using a Student’s t test and is indicated compared to d0 with *p* value ∗ <0.05, ∗∗ <0.01 and ∗∗∗ <0.001.

Differential expression across adipogenesis might indicate particular roles of selected members of KATs and KDACs in certain stages of the differentiation process. Hence, we explored time-dependent changes in KAT and KDAC expression in our SGBS RNA-seq dataset and a published dataset from 3T3-L1 cells.^26^ Expression analysis revealed dynamic time-dependent patterns in KAT and KDAC expression (Figures 2A and 2B), indicating similarities and differences between the two cell lines. KAT expression profiles revealed, nuclear GCN5 (GCN5 and PCAF) and MYST (MOF and MORF) family members in SGBS and PCAF in 3T3-L1 cells to be significantly upregulated toward terminal differentiation, while ESCO2 and HAT1 expression was downregulated after adipogenic induction in both cell lines. The mitochondrial class III KDACs (SIRT3, 4, 5) and the nuclear HDAC11 (class IV) were found significantly upregulated in maturing SGBS and 3T3-L1 adipocytes. Apart from this concordance, KDAC expression profiles in SGBS and 3T3-L1 cells showed predominantly species-specific differences. In SGBS cells, multiple nuclear class I (HDAC1 and 2), IIa (HDAC7 and 9), and III (SIRT 6 and 7) KDACs, as well as the DNA-binding protein TCF7 showed significantly reduced transcript levels after induction throughout major parts of adipogenic differentiation. Significantly increased transcript levels were found for nuclear SIRT1 and cytoplasmic SIRT2 (class III) as well as for cytoplasmic HDAC6 (class IIb). These trends are rather opposing in 3T3-L1 cells. For those, the significant reduction in SIRT1 and SIRT2 levels is consistent with the described role of these enzymes in inhibiting adipocyte differentiation and promoting lipolysis by deacetylating the key transcription factors PPARγ and FOXO1.^15^^,^^16^ The increase in SIRT1 and SIRT2 transcripts in differentiating SGBS cells (Figure 2C), together with the overall transcript levels of SIRT1, SIRT2, and SIRT3, that show a clear difference to 3T3-L1 adipocytes (Figure 2D), suggests a different mechanism in this cell line.

### Proteome, acetylome, and phosphoproteome show time-dependent profiles across adipocyte differentiation

While the transcriptome conveys information on potential protein expression, the proteome is a more direct link to the cells phenotype and further modulated by processes like protein release, accumulation, and degradation. These dynamics are also regulated by PTMs, which further impact protein functionality.^33^ As acetylation and phosphorylation depict important PTMs in adipocyte biology and adipogenesis^28^^,^^30^ and expression patterns of KATs and KDACs appear to be vividly dynamic across adipogenesis, we investigated protein acetylation and phosphorylation to identify relevant modification sites throughout differentiation of SGBS and 3T3-L1 cells, distinguishing between preadipocytes (SGBS/3T3-L1, d0), immature (SGBS, d6; 3T3-L1, d5) and mature adipocytes (SGBS, d12; 3T3-L1, d10).

**Figure 3:**
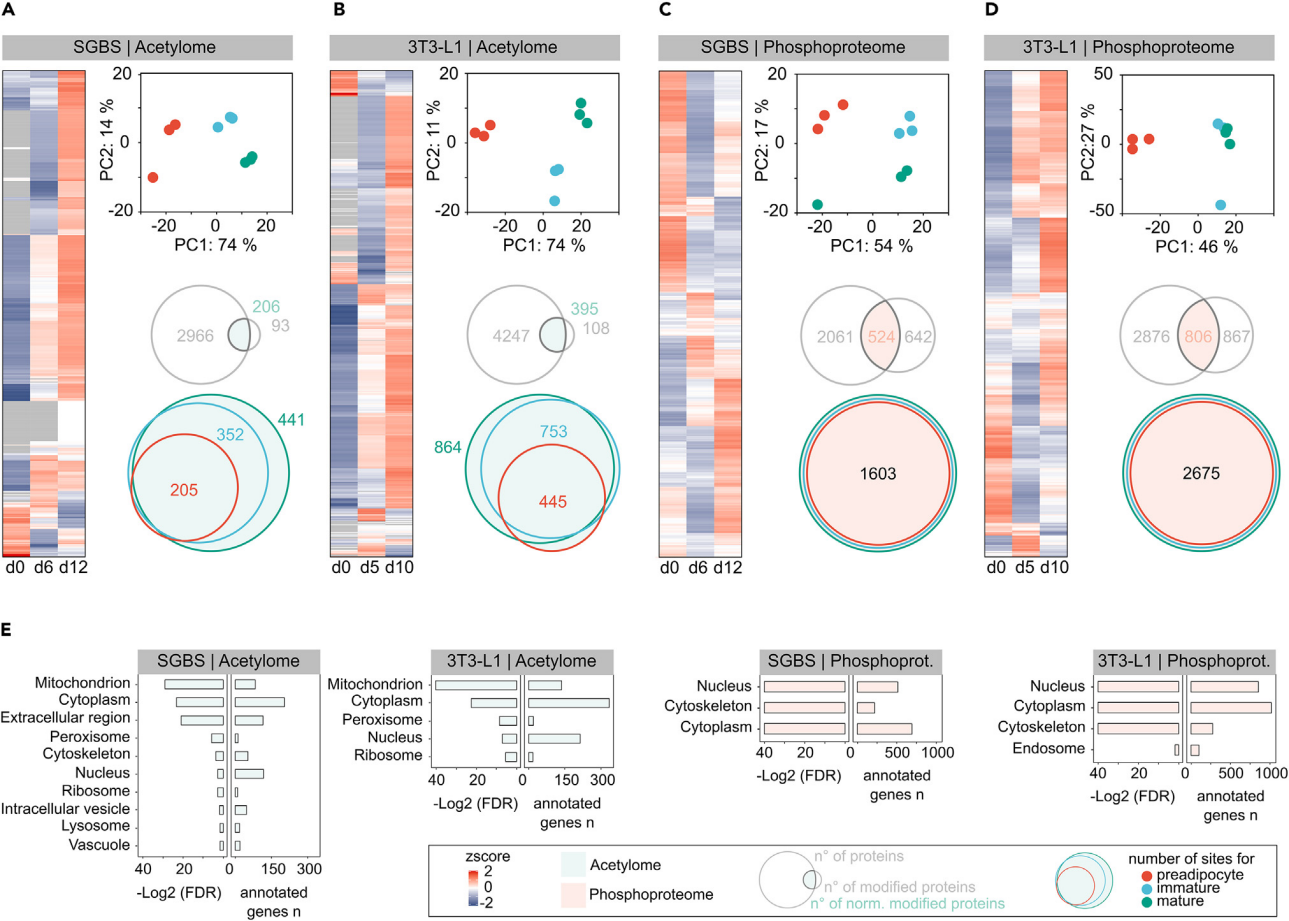
Profiling acetylome and phosphoproteome dynamics across adipogenesis (A) SGBS and (B) 3T3-L1 acetylome profiles. (C) SGBS and (D) 3T3-L1 phosphoproteome profiles. Z-scored heatmaps of average log2 intensities provide information about profiles of modified sites over differentiation. PCAs clearly distinguish preadipocyte, immature and mature adipocytes of both cell lines. Top row of Venn diagrams indicate overlap between full proteome and modified proteins. Upper Venn diagrams show the overlap in proteome-corrected modified sites found for preadipocytes, immature and mature adipocytes. (E) Enriched cellular compartments among acetylated and phosphorylated proteins.

Hence, we applied a global quantitative proteomic approach and used immunoaffinity purification to enrich acetylated label-free peptides, and simultaneously two sequential metal affinity enrichment strategies to enrich phosphorylated tandem mass-tagged peptides (Figure 1A). In SGBS cells, we obtained 3190 global and 310 acetylated proteins, with a total of 476 lysine acetylation sites (AcK) located on 217 proteins that were identified in both datasets and allowed for proteome correction of the site abundances (Figure 3A). In 3T3-L1 cells, 4655 global and 509 acetylated proteins were identified with a subset of 401 proteins found in both datasets and a total of 934 proteome-corrected AcK sites across all time points (Figure 3B). Enrichment of phosphorylation sites (PP) using a tandem mass tag (TMT)-based approach rendered a total of 1187 phosphorylated and 2606 global proteins, with a set of 545 proteins present in both datasets, leading to 1600 proteome-normalized PP sites in SGBS cells (Figure 3C). For 3T3-L1 cells, 1659 phosphorylated and 3668 global proteins with an overlap of 792 proteins representing 2666 normalized PP sites were quantified (Figure 3D).

The differentiation of a preadipocyte into a mature adipocyte represents a dramatic physiological change in the cellular phenotype, that was reflected in the transcriptome and proteome data (Figures S1B, S2A, and S2B) and was also clearly visible in the PCAs for all acetylome and phosphoproteome datasets (Figures 3A–3D). Accordingly, many of the detected AcK and PP sites in SGBS as well as 3T3-L1 cells showed distinct variation in abundance patterns across adipogenesis. For the acetylome of SGBS and 3T3-L1 cells, most changes involved an increase in site intensities with maturation and only a minor part displayed the opposite trend (Figures 3A and 3B). The two acetylome datasets showed a steady increase in the number of sites detected with maturation, with the vast majority of sites found in preadipocytes also present in mature adipocytes. For the enriched phosphoproteome, fewer missing values across the differentiation timeline were seen (Figures 3C and 3D). However, the proportion of sites, without counterpart in the global proteome, thus not eligible to normalization, was even larger. This might indicate a substantial effect of data-dependent acquisition missing out on low abundant proteins that were only detected as a result of PTM enrichments.

Enriched GO cellular compartments (GO CC) of modified proteins of the acetylome and phosphoproteome differed markedly, but were in good agreement between the cell lines (Figure 3E). Acetylated proteins showed the strongest enrichment for mitochondrial, cytoplasmic, and peroxisomal localization. Particularly in SGBS cells, proteins of the extracellular region were found enriched among acetylated proteins. Phosphorylated proteins were broadly enriched for nuclear and cytoplasmic localizations.

### Protein acetylation plays a central role in regulating carbon metabolism in adipogenesis

To gain a better understanding of the acetylation and phosphorylation sites that exhibited the most striking changes during adipogenesis, their links to cellular signaling pathways were analyzed. Differential abundance of sites was analyzed for early (immature vs*.* preadipocytes) and late (mature vs*.* immature adipocytes) adipogenesis and for the complete process (mature vs*.* preadipocytes).

**Figure 4:**
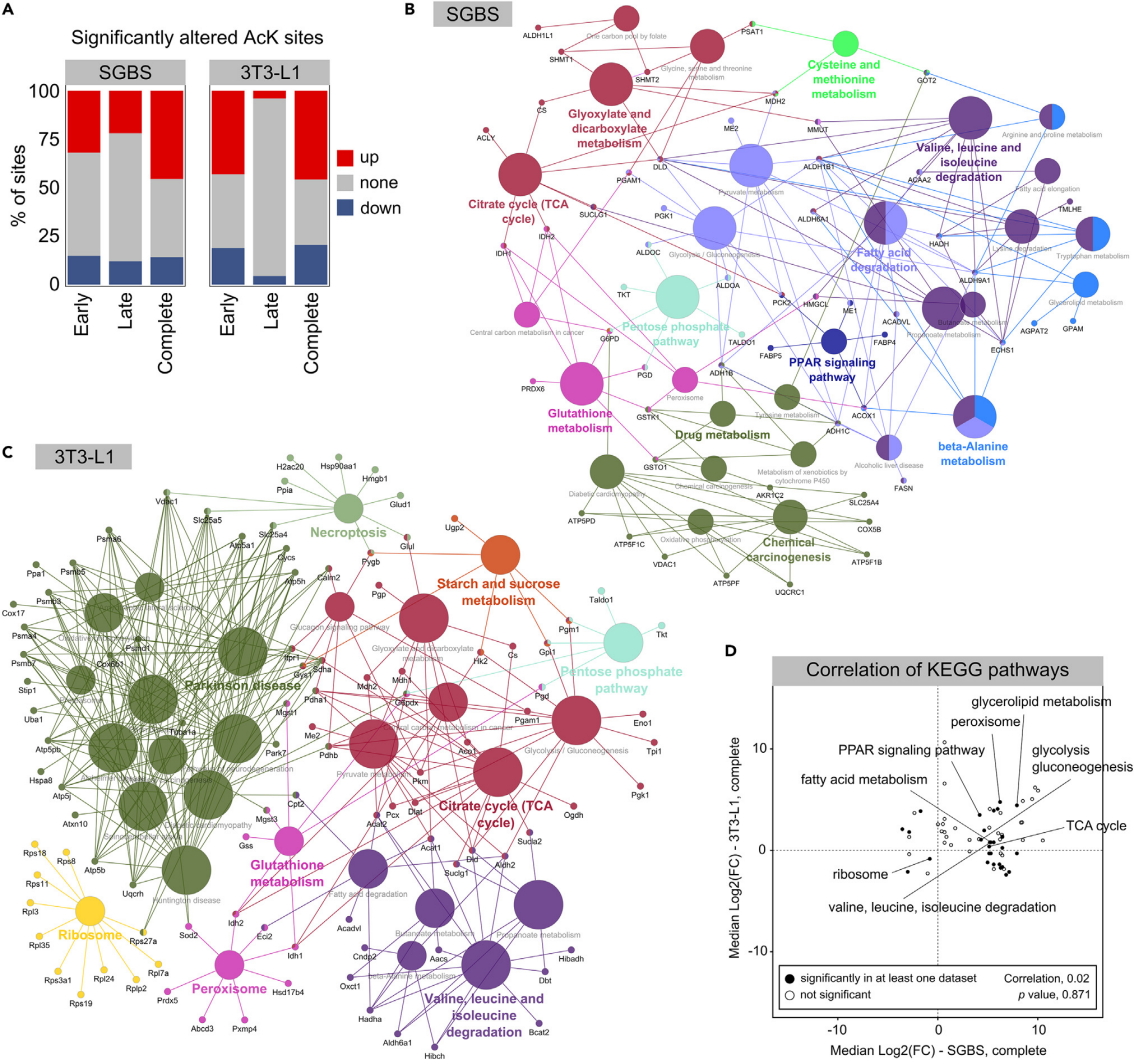
Exploring the role of acetylation in adipocyte differentiation (A) Percentages of significantly altered AcK sites in early, late and the complete process of adipogenesis. Significant alterations were defined as adjusted *p-*value < 0.01. (B) KEGG enrichment of all SGBS and (C) 3T3-L1 proteins that showed differentially abundant acetylation sites across adipogenesis (complete). Displayed are enriched processes with a *p* value < 0.05. (D) Pearson correlation of enriched KEGG pathways between both cell lines for the complete adipogenesis. Processes that showed an upregulation in both cell lines upon maturation were located in the upper right quadrant, while those that appear downregulated in the mature compared to the preadipocytes, for both cell lines were located in the lower left quadrant. Terms with significant enrichment in at least one dataset were marked with a filled dot.

The percentage of differentially abundant AcK sites was largest across the complete process of SGBS and 3T3-L1 adipogenesis, but was already pronounced in early adipogenesis, comparing immature to preadipocytes (Figure 4A). A Kyoto Encyclopedia of Genes and Genomes (KEGG) enrichment was performed for the set of proteins that had sites showing significantly different acetylation levels comparing mature to preadipocytes. In SGBS cells, the enrichment resulted in a variety of metabolic pathways associated with central metabolism, such as the degradation of branched-chain amino acids (BCAAs; valine, leucine, isoleucine), fatty acid degradation, the pentose phosphate pathway, the TCA cycle, or glyoxylate and dicarboxylate metabolism (Figure 4B). The PPAR signaling pathway with its central role in adipogenesis was also found enriched among the acetylated protein population of SGBS cells. For 3T3-L1 adipocytes, differentially acetylated proteins were enriched for several pathways associated with central metabolism, namely BCAA degradation, the TCA cycle and pentose phosphate pathway, glutathione or starch and sucrose metabolism (Figure 4C). Additionally, peroxisomal and ribosomal proteins were found involved according to their assignment to the enriched Parkinson disease. This broad cluster mainly consists of proteins of the proteasome, the mitochondrial ATP synthase subunits and SLC25 carrier family members. Some of the proteins closely linked to acetyl-CoA formation showed differentially abundant acetylation sites across adipogenesis themselves, e.g., ACLY, IDH1, IDH2, CS and ME1 in SGBS and Idh1, Bcat2, Mdh1, Mdh2, Pkm, and Cs in 3T3-L1 cells (Figures S3 and S4).

As the described enrichment analysis is lacking information about the direction of the observed changes and to directly compare the acetylome of the two cell lines, enriched KEGG pathways were correlated for significance and directionality of their changes, which was based on the median log2 fold changes of all differentially abundant sites assigned to the process (Figure 4D). Pearson correlation between the two adipocyte cell lines was weak and non-significant. However, many pathways with demonstrated relevance in adipogenesis were found significantly enriched with the same directionality in both cell lines. Among them PPAR signaling, glycerolipid and fatty acid metabolism, glycolysis and gluconeogenesis, the TCA cycle or BCAA degradation. This might indicate a similar role of protein acetylation in adipogenesis, regardless of species and cell line origin.

### Phosphorylation is a key modification in insulin and glucagon signaling and regulation of cell structural components in differentiating adipocytes

**Figure 5:**
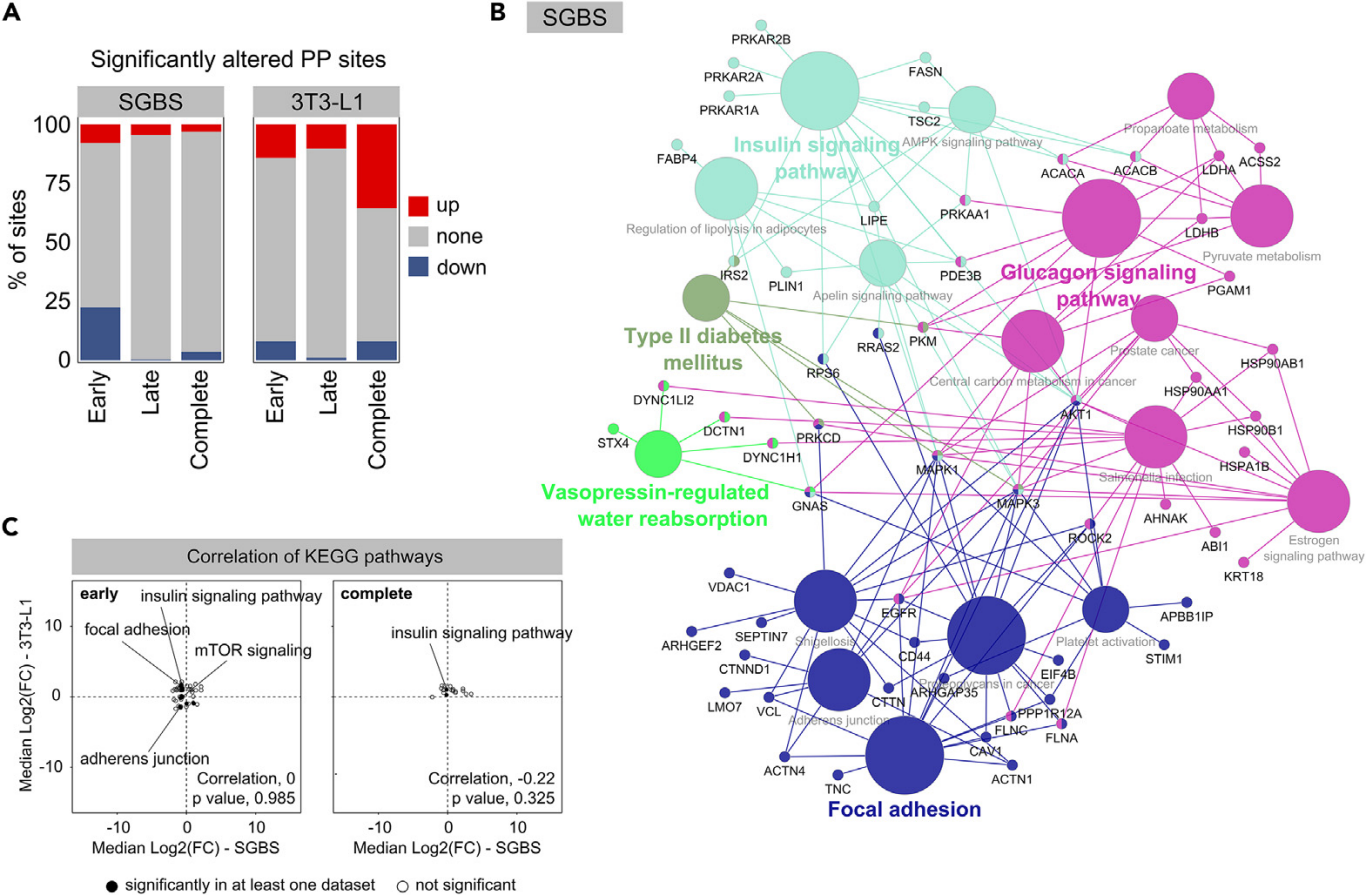
Exploring the role of phosphorylation in adipocyte differentiation (A) Percentages of significantly altered PP sites in early, late and the complete process of adipogenesis. Significant alterations were defined as adjusted *p* value < 0.01. (B) KEGG enrichment of all SGBS proteins that showed differentially abundant PP sites across early adipogenesis. Displayed are enriched processes with a *p* value < 0.05. (C) Pearson correlation of enriched KEGG pathways between both cell lines for early and complete adipogenesis. Processes that showed an upregulation in both cell lines upon maturation were located in the upper right quadrant, while those that appear downregulated in the mature compared to the preadipocytes, for both cell lines were located in the lower left quadrant. Terms with significant enrichment in at least one dataset were marked with a filled dot.

In contrast to the analysis of differentially abundant acetylation sites throughout adipocyte differentiation, differential phosphorylation indicated an even more pronounced difference between the cell lines. Differentially PP site intensities were found mainly in early adipogenesis of SGBS cells and across the complete adipogenesis of 3T3-L1 cells with low numbers of significant alterations in late adipogenesis of both investigated cell lines (Figure 5A). This might indicate important roles of phosphorylation in the onset of adipogenesis, while to a lesser extent associated to the later stages of maturation.

The KEGG enrichments were performed on the comparison with the highest number of differentially phosphorylated proteins of each cell line, namely early adipogenesis in SGBS and complete adipogenesis in 3T3-L1 cells. In SGBS cells, insulin and glucagon signaling, central carbon metabolism as well as structural components such as focal adhesions were found enriched among the differentially phosphorylated population in early adipogenesis (Figure 5B). Enrichment in the set of 3T3-L1 early adipogenesis did not result in any significantly enriched pathways. For the differentially abundant set of proteins during the complete adipogenesis, enrichment was not very pronounced but was found partially comparable to SGBS results in terms of insulin and glucagon signaling and structural components associated to adherens junctions (Figure S5). Few other pathways were found enriched but were not supported by high numbers of assigned proteins (Figure S5).

Compared with protein acetylation, fewer phosphorylation sites showed significant up- or down-regulation during adipocyte differentiation, and the differences between cell lines appear to be more pronounced. The enriched pathways barely correlated with the other cell line during the first half and complete duration of adipogenesis, and even the directionality was inconsistent between SGBS and 3T3-L1 cells (Figure 5C).

### The results of an integrated correlation analysis of proteome, acetylome, and phosphoproteome during the course of adipogenesis are comparable for SGBS and 3T3-L1 adipocytes

To identify key modification sites and proteins involved in the progression of adipogenic differentiation among all involved candidate sites and proteins, we performed a *p* value independent integrated co-expression analysis using global proteome, protein-normalized acetylome, and phosphoproteome data. This *Weighted Gene Correlation Network Analysis* (WGCNA) approach forms modules, named by colors, based on similarity in abundance patterns of the input data, independent of abundance levels (Figures S6A–S6D). Modification sites and proteins that show a similar pattern over the time course of adipogenesis, are grouped into the same module.

**Figure 6:**
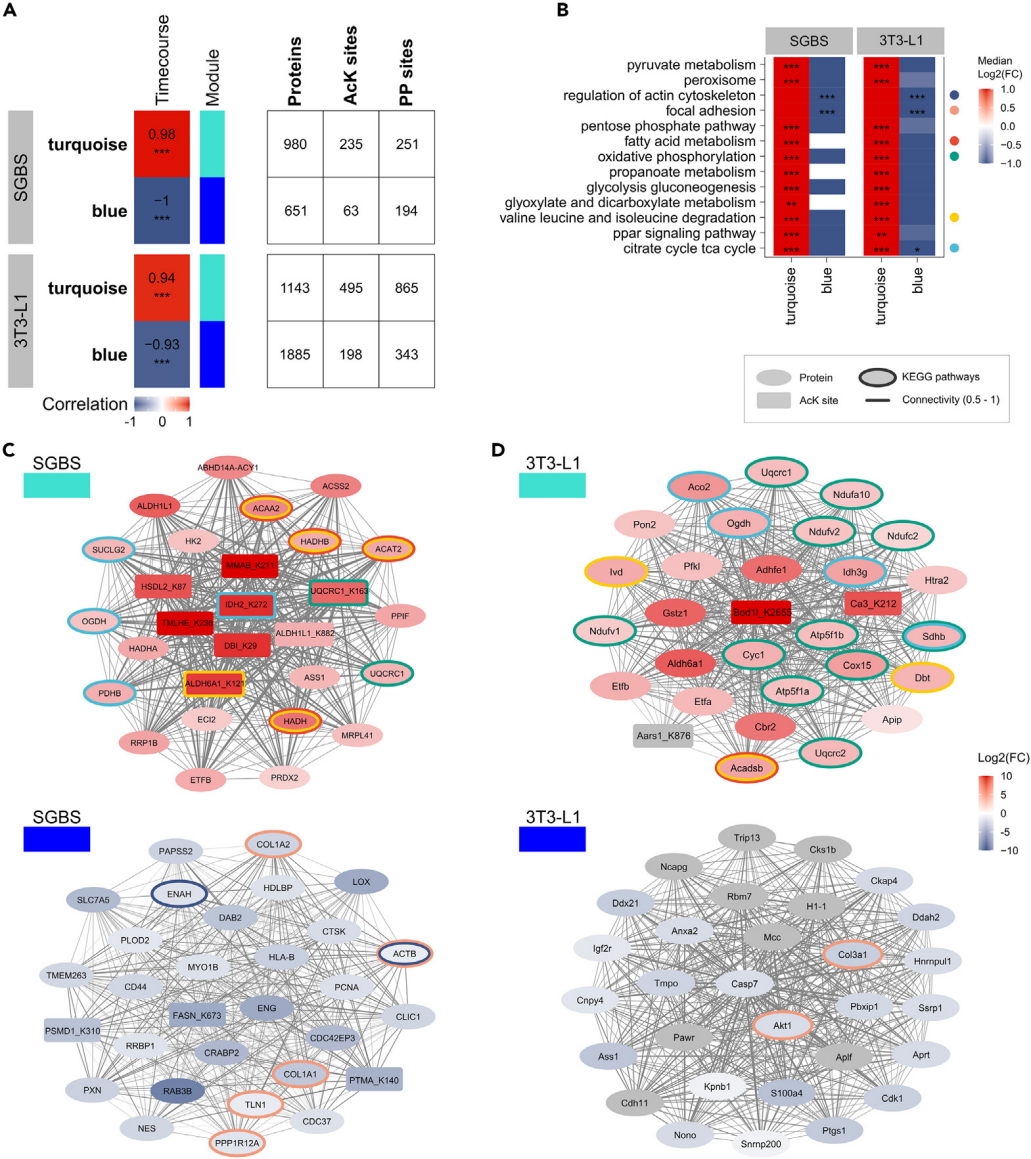
Integrated correlation analysis of proteome, acetylome and phosphoproteome during the course of adipocyte differentiation (A) Selection of modules of co-abundant proteins and modification sites correlating with the time course of adipogenesis. Numbers of proteins, AcK and PP sites contained in each module are indicated. (B) Selection of enriched KEGG pathways of the turquoise and blue modules. (C) Top 30 key drivers of the selected modules for SGBS and (D) 3T3-L1 adipocytes. Dataset is indicated by shape, fold changes across the complete adipogenesis by fill color and affiliation with enriched KEGG pathways as border color. Connectivity in the range of 0.5–1 between the Top 30 candidates marked by line width. Connectivity below 0.5 is indicated by line size 0.1. Pathway data are displayed as median +/− SD, candidate data are displayed as mean ± SD. Significance is indicated as *p* value ∗ <0.05, ∗∗ <0.01 and ∗∗∗ <0.001.

Of all eleven constructed modules, the ones with the strongest correlation for the time course of adipogenesis were the turquoise and blue module for both cell lines (Figures 6A and S6C). The turquoise modules showed a significant positive and the blue modules a significant negative correlation with the course of adipocyte differentiation. These modules were dominated by proteins, with fewer AcK and PP sites. A module-wise KEGG enrichment indicated the turquoise modules to be strongly enriched for pathways of central energy and carbon metabolism and PPAR signaling, and the negatively correlating blue modules to be strongly enriched for the regulation of actin cytoskeleton and focal adhesions (Figures 6B, S7, and S8). A correlation between the module membership for the respective module and trait protein/site significance for the time course (Figure S6D) was used to determine the Top 30 key drivers. Notably, several of the identified key driver candidates were assigned to the pathways we found enriched, which are undoubtedly relevant in adipogenesis, thus rendering also the other identified key drivers potentially impactful (Figures 6C and 6D). Connectivity between the Top 30 potential key driver of each selected module was visualized as network to decipher the most relevant and interlinked candidate proteins and sites (Figures 6C and 6D). No phosphorylation sites were found among the Top 30 key driver selections of the changes across adipogenesis, suggesting that constitutive phosphorylation might not be a strong and continuous driver of the changes across the complete time course of adipocyte differentiation.

In SGBS cells, putative key drivers of the turquoise module that showed a positive correlation in this cell line were associated with fatty acid metabolism/BCAA degradation (ACAA2, HADHB, ACAT2, HADH, ALDH6A1_K121), oxidative phosphorylation (UQCRC1 and its acetylated lysine K163) and the TCA cycle (SUCLG2, OGDH, PDHB, IDH2_K272) (Figure 6C). Strikingly, the included acetylation sites exhibited the strongest connectivity and average log2 fold change among candidates and across adipogenesis, indicating their prominent role in differentiation. The Top 30 candidates for the blue module that was negatively correlated across the time course of adipogenesis were predominantly associated with cell structural components, including focal adhesions (COL1A1, COL1A2, ACTB, TLN1, PPP1R12A) and the actin cytoskeleton (ACTB, ENAH). Motorprotein family member myosin 1b (MYO1B) was also found among the Top 30 candidate selection (Figure 6C).

In 3T3-L1 cells, the Top 30 key driver selection was a good surrogate of the module enriched pathways and included members of BCAA degradation and fatty acid metabolism (Acadsb, Ivd, Dbt), the TCA cycle (Aco2, Ogdh, Idh3g, Sdhb) and many candidates related to energy metabolism of oxidative phosphorylation (Atp5f1a, Atp5f1b, Cox15, Cyc1, Ndufa10, Ndufv1, Ndufv2, Ndufc2, Sdhb, Uqcrc1, Uqcrc2) (Figure 6D). In parallel to the SGBS cells, the few included acetylation sites showed strong average log2 fold changes across adipogenesis and central location in the connectivity network, suggesting high inter-connectivity (Figure 6D). Potential key drivers of the negatively correlating blue module mainly included nuclear proteins and two members regulating the actin cytoskeleton pathway (Akt1, Col3a1) (Figure 6D).

Although the selection of the Top 30 key driver candidates varied between SGBS and 3T3-L1 adipocytes (Figures 6C and 6D), the metabolic pathway affiliations correspond (Figure 6B). In both cell lines, key drivers of the positively correlating turquoise modules are dominantly found in pathways associated to central carbon and energy metabolism, while key drivers of the negatively correlating blue modules are associated to pathways of cell structure (Figure 6B).

Our analysis of transcriptome, proteome, acetylome, and phosphoproteome of differentiating SGBS and 3T3-L1 adipocytes reveal major similarities between the two cell lines, although the details vary.

## Discussion

Model adipocyte cell lines such as SGBS and 3T3-L1 are essential for studying the molecular mechanisms of adipogenesis and adipocyte physiology in health and disease. Adipogenesis is a highly complex process in which preadipocytes differentiate into lipid-laden mature adipocytes, a process that is decisively controlled by the PTMs phosphorylation and acetylation. Here, we provide a comprehensive, time-resolved resource of the levels of central carbon metabolites, transcripts, proteins, phosphorylation, and acetylation sites across adipogenesis in SGBS and 3T3-L1 cells. All datasets have been made publicly available.

The TCA cycle is the central hub of adipocyte metabolism as it provides crucial intermediates for energy supply and downstream metabolic processes. During adipogenesis of 3T3-L1 cells, the abundance of enzymes and metabolites of glycolysis and TCA cycle increased (Figure 1D), once again demonstrating the key role of central carbon metabolism for adipocyte maturation and fatty acid synthesis. This is consistent with studies that found the volume of the TCA cycle to be increased upon differentiation of 3T3-L1 cells on metabolite^34^ and protein levels.^27^ In parallel, our data in differentiating SGBS cells reveal an increased abundance of most glycolysis and TCA enzymes in mature compared to preadipocytes that has also been described in literature.^25^^,^^35^ In contrary to this, the TCA metabolites citrate to fumarate showed decreasing abundances upon SGBS differentiation, thereby opposing the trends in abundance of the enzymes directly involved. This is initially unexpected as citrate together with acetyl-CoA displays the direct precursors for *de novo* lipogenesis, a pronounced feature in adipocytes. In agreement with our data, a relative depletion of TCA cycle metabolites was also seen in a primary cell culture of differentiating insulin resistant subcutaneous adipocytes from fat biopsies of subjects with metabolically unhealthy morbid obesity.^36^ Vice versa, the decreasing metabolite levels might refer to an increased metabolite flux toward lipogenesis. As to our knowledge no study has yet examined TCA cycle metabolites across differentiation of SGBS cells, we can only postulate that this decreasing trend with maturation is characteristic for the SGBS cell line, indicating different metabolic activity, metabolite utilization and intermediary replenishment of the TCA cycle in SGBS compared to 3T3-L1 cells.

Acetyl-CoA is not only the precursor of fatty acid synthesis, but also the only substrate for protein acetylation, present in distinct cellular pools.^18^ In both cell lines, we found acetylated proteins enriched in mitochondria, cytoplasm, and nucleus among others (Figure 3E), which is likely reflecting exploitation of the mitochondrial, cytoplasmic and nuclear pool of acetyl-CoA synthesis and conversion, and agrees with the subcellular localizations of important KATs and KDACs (Figures 2B and 2C).

We observed similarities and differences in the dynamic expression of KATs and KDACs across differentiation of SGBS and 3T3-L1 cells (Figures 2B and 2C). It is worth noting that the analysis of the 3T3-L1 cells is based on published data, possibly affecting direct comparability. SIRT2 is described to be the most abundant sirtuin in 3T3-L1 adipocytes and transcript levels are negatively correlating with the progression of adipogenesis.^16^ While the same was reported for SIRT1 mRNA levels, protein levels of SIRT1 continuously increased post adipogenic induction in another study.^15^ The decreasing trend is clearly visible in the 3T3-L1 transcriptome (Figures 2B and 2C), however, our data on differentiating SGBS cells indicate the opposite. There was neither a significant decrease in SIRT1 and SIRT2 levels with adipocyte differentiation, nor a prominent difference in overall transcript abundance between SIRT1, SIRT2, and SIRT3, implying a differently weighted control of acetylation levels in SGBS cells.

In agreement between differentiating SGBS and 3T3-L1 cells, we observed a significantly increased abundance of the mitochondrial class III KDACs (SIRT3, 4 and 5) as well as of the nuclear HDAC11 (Figure 2C) compared to initiation of differentiation, implicating that especially mitochondrial acetylation mechanisms are well-retained between the two cell lines. Fundamental functions of mitochondrial biology are regulated by sirtuins, including central metabolism, energy production and cellular signaling.^37^^,^^38^ SIRT3 depicts the major mitochondrial lysine deacetylase,^39^ with demonstrated role in the regulation of adipogenesis, expression of adipocyte markers and adipokines.^40^ In addition, several subunits of the inner mitochondrial membrane-located ATP synthase and the mitochondrial SLC25 carrier family^41^ showed differential acetylation during differentiation of SGBS and 3T3-L1 adipocytes (Figures 4B and 4C), indicating protein regulation by lysine acetylation to be impacting diverse mitochondrial inner membrane-associated processes. Both enzyme families are involved in energy production, either directly by utilizing the proton electrochemical gradient for ATP synthesis from ADP, or indirectly by mediating across-membrane transport of nutrients for energy conversion, and have demonstrated roles in adipogenesis.^42^^,^^43^ Despite the finding that downregulation of several HDACs is important for adipogenic differentiation,^44^ HDAC11 deficiency was found to reduce lipid accumulation and PPARγ levels,^45^ thus an increasing abundance of HDAC11 and PPARγ during adipogenesis as seen in our SGBS transcript data inversely confirms this finding (Figures 1C and 2C).

Apart from alterations in PTM abundances as immediate response to a certain stimulus, the constitutive acetylome or phosphoproteome provides valuable information on modifications that might impact the protein carrying them in regards of its activity, structure, or interactions for a longer duration. The analysis of PTMs from tissues often also focuses on these longer-term modifications, still contributing relevant data on, for instance, the phosphoproteome in white^46^ and brown^47^ adipose tissue following diet induced obesity in mice or the acetylome in human adipose tissue during obesity and insulin resistance.^29^ We observed the constitutive acetylome to become more pronounced in differentiating SGBS and 3T3-L1 cells (Figures 3A and 3B), which goes hand in hand with the overall increase in cellular metabolic activity and increase in available cellular acetyl-CoA that accompanies adipocyte differentiation. In SGBS but not 3T3-L1 cells PPAR signaling was found enriched based on the proteins that were differentially acetylated across adipogenesis (Figure 4B), implying that SIRT1 control on PPARγ and pathway-associated enzymes might be a consequence of the differences in SIRT1 prominence between SGBS and 3T3-L1 cells (Figure 2D). In contrast, many cellular pathways that are associated with the synthesis and maintenance of acetyl-CoA were found enriched among the differentially acetylated protein subsets in both cell lines (Figures 4B and 4C). These included central functions like the TCA cycle, BCAA and fatty acid degradation. Even proteins that are directly involved in acetyl-CoA turnover were found acetylated, indicating that the flourishing availability of the modification substrate might enable numerous acetylation’s. Sirtuins as NAD^+^-dependent lysine deacetylases, are highly responsive to physiological conditions. In conditions of nutrient starvation, SIRT1 expression was increased in neural-crest derived PC12 cells,^48^ while it was reduced following high-fat feeding in the diet-induced obese SRC-3 mice.^49^ Additionally, if glycolytic flux is high, the levels of NAD^+^ are low, thus co-factor requirements of sirtuins are not met and acetylation levels are elevated accordingly.^50^^,^^51^^,^^52^ Apart from enzyme-mediated acetylation, non-enzymatic acetylation is favored in environments of alkaline pH, as found in the mitochondrial matrix.^53^ This owes to the assumption that mitochondrial sirtuins have evolved to maintain protein acetylation levels in this organelle,^54^ supporting the hypothesis mentioned earlier, that mitochondrial acetylation levels are well-retained between human SGBS and murine 3T3-L1 adipocytes and that the high acetylation levels observed in mature adipocytes could be attributed to SIRT1 inhibition by low NAD^+^ together with increased non-enzymatic acetylation.

In contrast to acetylation, we found the numbers of differentially phosphorylated proteins to be higher in the first than in the second half of adipogenesis in SGBS and 3T3-L1 adipocytes (Figure 5A), presuming that phosphorylation or dephosphorylation events fulfill particularly important functions in the onset and early progression of adipogenesis. Adipogenic induction is characterized by the initiation of signaling pathways that promote the differentiation of preadipocytes into mature adipocytes, some of which are regulated by (de-)phosphorylation events. Literature mentions for instance, mitogen-activated protein kinase (MAPK)-mediated phosphorylation of PPARγS112 reduces its transcriptional activity, thereby attenuating adipogenesis.^55^ In addition, phosphorylation of histone H2B S36 by the kinase S6K1 is followed by trimethylation of histone H3 K27, facilitating MSCs commitment to the adipogenic lineage and were found elevated in obese humans and in mice fed a high fat diet.^56^ The two examples of transcription factor and histones aforementioned could not be resolved in our proteomic data, but demonstrate early phosphorylation events that may be decisive for the progression of adipogenic differentiation.

Generally, concordant between the differentially phosphorylated proteins in both cell lines are their enriched subcellular localizations nucleus, cytoplasm, and cytoskeleton (Figure 3E), as well as their pathway associations with insulin and glucagon signaling and focal adhesions (Figures 5B and S5). Phosphorylation is known to be central for functional insulin signaling^57^ and has been comprehensively analyzed in 3T3-L1 cells exhibiting defective insulin signaling using global phosphoproteomics, to account for its complex multi-node involvement.^28^ Just like insulin, glucagon is a pancreatic hormone, which together mediate glycemic control.^58^ The binding of glucagon to its membrane receptor initiates a signaling cascade, involving G proteins and adenylate cyclase that culminates in the increase of intracellular cAMP levels, subsequently activating protein kinase A (PKA) that phosphorylates further downstream effectors.^59^ Even though the glucagon receptor was found expressed in adipocytes from rat^60^ and human liposarcoma,^61^ physiological levels of glucagon did not show an effect on lipolysis in murine WAT.^62^ Thus, hepatic glucagon signaling is relying on phosphorylation to mediate fed-state appropriate blood glucose levels, only with peripheral mechanisms acting in adipocytes. Although overall trends in the phosphoproteome agree between SGBS and 3T3-L1 cells, the species and cell line-specific differences seem more pronounced than for the adipocytes acetylomes.

Differences between both cell lines are apparent as SGBS cells derive from the subcutaneous adipose tissue of a male infant with Simpson-Golabi-Behmel syndrome (SGBS),^63^ while 3T3-L1 cells are fibroblast-like cells that originate from a 17- to 19-day-old Swiss 3T3 mouse embryo.^20^ Additionally, the differentiation protocols of both adipocyte models differ slightly in duration (SGBS: 12 days; 3T3-L1: 10 days) and in media supplements (see method section on cell culture). Adipose tissue depot and metabolic state have been shown to impact the acetylome in human subcutaneous and omental adipose tissue from lean, normal-glycaemic and insulin-resistant donors with obesity.^29^ Several acetylated proteins reported therein including ACAA2, ECHS1, HADHA, HADHB, CS, SUCLG1, PGD, UQCRC1, UQCRCH, CYCS, COX5B, ETFA, ALDOA, PGK1, GPD1, PRXL2A, SQOR, and ADH1B were also identified in our acetylome dataset. All of the aforementioned differences in cell line specificities could hence influence the specific profiles of phosphorylation, acetylation, and central carbon metabolites observed throughout differentiation of SGBS and 3T3-L1 adipocytes. However, the consistent data emphasize the relevance of our findings to the *in vivo* situation in adipose tissue in health and disease.

We identified co-abundant protein, AcK- and PP site key driver candidates of the biological changes undertaken during the course of adipocyte differentiation in SGBS and 3T3-L1 cells. Positive correlations with adipogenesis were seen for core metabolic pathways, whereas pathways associated to cell structural components showed negative correlations (Figure 6B). This WGCNA revealed pronounced similarities between both adipocyte models in terms of their global cell physiological makeup. Several of the Top 30 selected putative key drivers were involved in the processes enriched in their corresponding module (Figures 6C and 6D), confirming them as good surrogates of the whole module and supporting the importance of the other identified key driver candidates. Interestingly, AcK sites showed the highest log2 fold changes and strong connectivity within the selection, confirming their pivotal role in adipogenesis.

Lysine 272 (K272) of (NADP) isocitrate dehydrogenase (IDH2) displayed a particularly central position with high log2 fold change and strong connectivity within the Top 30 key drivers of the turquoise module of SGBS adipocytes (Figure 6C). Acetylation at this position has already been described in other cells and tissues, including myeloid leukemia (MV4-11), lymphoid (Jurkat), and pulmonary (A549) cells^64^ such as in insulin-secreting (INS-1E) cells^65^ and mouse liver.^66^ In all of these studies, acetylation levels of IDH2 K272 remained unchanged upon SIRT3-selective inhibition (SAHA, MS-275) or knockdown. This implies that, apart from its demonstrated role in SGBS adipogenesis, acetylation of IDH2 K272 is crucial for central metabolism and well-conserved between different organs, however, remains unaffected by SIRT3 deacetylation.

An interesting key driver candidate of the negatively correlating blue module of SGBS adipocytes (Figure 6C), that has been discussed in the context of lipid accumulation is FASN K673.^67^ FASN as established adipogenesis marker, was found at increased abundance in the transcriptome and proteome of SGBS and 3T3-L1 cells (Figure 1C) and half of the detected FASN AcK sites were increasingly acetylated with SGBS differentiation (Figure S3). FASN K673 was identified as potential key driver of the negatively correlating blue module of differentiating SGBS cells showing decreasing acetylation levels over the course of adipogenesis. Histone lactylation has been introduced as novel epigenetic modification regulating gene transcription from chromatin,^68^ but it has recently become clear that this modification is also widespread in non-histone proteins.^69^ Indeed, lactylation of FASN K673 inhibited enzymatic activity and reduced mitochondrial pyruvate carrier 1 (MPC1)-mediated lipid deposition in the liver in heterozygous MCP1 knockout mice.^67^ This finding is consistent with our data, suggesting de-modification of FASN K673 to be important for the progression of adipogenesis, regardless of the specific modification, i.e., lactylation or acetylation.

Dynamic and balanced remodeling of the extracellular matrix (ECM) is fundamental for functional adipocytes as their structure develops from fibrillar to laminar during adipogenesis.^70^ ECM regulation and its effects on metabolic functionality in obesity appear in depot-^71^ and species-specific differences,^72^ suggesting ECM deposition as an indicator for metabolic disorders. The membrane receptor CD44, differentially phosphorylated in early adipogenesis at S161 (log2 FC 1.11; Figure 5B) and identified as key driver of the blue module of SGBS cell differentiation (Figure 6C), is an established molecule involved in ECM remodeling with recently suggested importance in cell metabolism, including insulin resistance and inflammation in diabetes and obesity.^73^ Expression-based genome-wide association studies (eGWAS) identified CD44 as top candidate associated with adipose tissue from diabetic compared to control subjects,^74^ and only recently it was shown that knocking down CD44 increased differentiation in primary human subcutaneous adipocytes.^75^ These findings support our data that identify CD44 to be among the Top 30 key driver candidates showing a negative correlation with adipogenesis of SGBS subcutaneous human adipocytes, potentially regulated by phosphorylation at CD44 S161, located in proximity to a binding region at the extracellular amino-terminal domain.^76^

**Figure 7:**
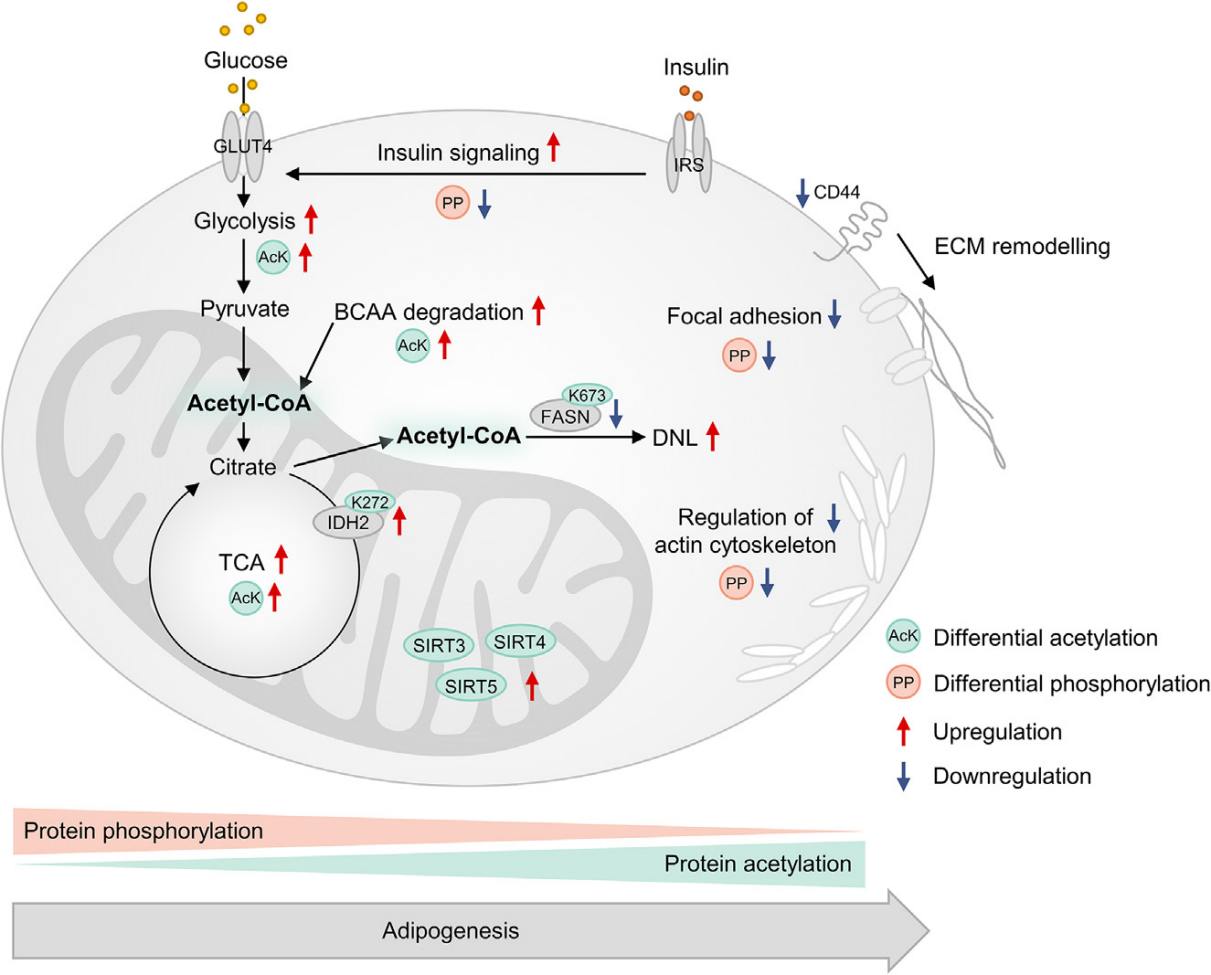
Involvement of acetylation and phosphorylation in adipogenesis Acetylation displays a tight link between cellular metabolism and signaling and appears particularly involved in core metabolic processes centered around the mitochondrial TCA cycle and associated acetyl-CoA provision and utilization. The mitochondrial lysine deacetylases SIRT3, SIRT4, and SIRT5 were upregulated over the course of adipocyte differentiation. Phosphorylation seems most important in the early stages of adipogenesis, and is involved in insulin signaling and cell structural organization associated to focal adhesions and regulation of the actin cytoskeleton. CD44 and the acetylation site FASN K673 were identified as putative key drivers with negative correlation for adipocyte differentiation, while the acetylation site IDH2 K272 was identified as key driver candidate positively correlating with adipogenesis.

We have summarized our main findings in Figure 7. In conclusion, we identified several AcK sites as strong key drivers for the correlation of PTMs with the progression of adipogenesis, while PP sites did not appear in this stringent selection. This may be related to the direct link between protein acetylation and acetyl-CoA availability, leading to the central role of acetylation in carbon metabolism and energy homeostasis, which is an indispensable and inherent trait in adipocyte physiology, regardless of species and details of cell lineage origin. We demonstrated similarities and differences between SGBS and 3T3-L1 adipocytes, with the overall molecular processes during adipogenesis to be relatively similar, while details revealed species-/cell line-specificities. Apart from the examples discussed here, the presented proteomes, phosphoproteomes, and acetylomes of the established adipocyte models SGBS and 3T3-L1 and the included SGBS transcriptome represent valuable resources for future research questions to be explored to advance knowledge on adipogenic differentiation relevant to the development of obesity and related diseases.

### Limitations of the study

The mass spectrometry-based proteomic analysis of PTMs still requires vast amounts of protein input material for the enrichment, rendering potentially incomplete data when input material is limited. This is particularly critical for the antibody-based enrichment of acetylated lysines. Low abundant proteins are difficult to detect in data-dependent acquisition, but it is known that modifications of low abundant key adipogenesis transcription factors such as PPARγ or FOXO1 are critical for regulating the differentiation. Despite enrichment strategies, the PTM information remains limited to prominent modification sites. Information on the functional role of specific modification sites can only be obtained by validating their effect on protein activity, localization, interactions, structure etc. in follow-up experiments using site-specific mutations, selective inhibitors or overexpressions, which is beyond the scope of this study. This clearly limits the mechanistic insight provided herein. As an in-depth understanding of the molecular mechanisms underlying obesity is of great importance, a more mechanistic approach could be the starting point for future studies. Although SGBS and 3T3-L1 cells both depict commonly used *in vitro* models of adipogenesis, they differ in distinct origin and disease background (SGBS), which must be considered when interpreting direct comparisons. Future studies should therefore also consider the analysis of acetylation and phosphorylation in primary cells from diverse depot origins including the analysis of different disease states.

## STAR★Methods

### Key resources table

**Table:** 

REAGENT or RESOURCE	SOURCE	IDENTIFIER
**Chemicals, peptides, and recombinant proteins**		

**DMEM/F12**	Gibco ^TM^	Cat# 11554546
**DMEM high glucose**	Gibco ^TM^	Cat# 11995065
**Biotin**	Sigma-Aldrich	Cat# B4639
**Panthothenate**	Sigma-Aldrich	Cat# PHR1232
**Penicillin/streptomycin**	Sigma-Aldrich	Cat# P4333
**Fetal Bovine Serum**	Gibco ^TM^	Cat# A3160802
**Cortisol**	Sigma-Aldrich	Cat# H0888
**apo-transferrin**	Sigma-Aldrich	Cat# T2036
**Triiodothyronine**	Sigma-Aldrich	Cat# T6397
**Insulin**	Sigma-Aldrich	Cat# I2643
**Rosiglitazone**	Sigma-Aldrich	Cat# R2408
**Dexamethasone**	Sigma-Aldrich	Cat# D1756
**3-isobutyl-1-methylxanthine**	Sigma-Aldrich	Cat# I5879
**Oil Red O**	Sigma-Aldrich	Cat# O0625
**Nile Red**	Sigma-Aldrich	Cat# 72485
**DAPI**	Thermo Fisher Scientific	Cat# D1306
**Saponin**	Sigma-Aldrich	Cat# 47036
**Paraformaldehyde**	Sigma-Aldrich	Cat# 158127
**Sodium orthovanadate**	Sigma-Aldrich	Cat# 567540
**Sodium pyrophosphate dibasic**	Sigma-Aldrich	Cat# P8135
**ß-glycerophosphate**	Alfa Aesar	Cat# L03425
**Dithiothreitol**	GE Healthcare	Cat# GE17-1318-02
**2-Iodoacetamid**	Merck	Cat# 804744
**Sequencing Grade Modified Trypsin**	Promega	Cat# V5111
**TCEP**	Sigma-Aldrich	Cat# C4706
**Ammonium formiat**	Sigma-Aldrich	Cat# 156264
**TRIzol**^**TM**^	Thermo Fisher Scientific	Cat# 15596026
**Tributylamine**	Sigma-Aldrich	Cat# 90780
**Acetic Acid**	Merck	Cat# 100063

**Critical commercial assays**		

**Pierce™ 660 nm Protein-Assay-Reagenz**	Thermo Fisher Scientific	Cat# 22660
**PTMScan**® **Acetyl-Lysine Motif [Ac-K] Kit**	Cell Signaling Technology	Cat# 13416
**High-Select**^**TM**^**TiO2 Phosphopeptide Enrichment Kit**	Thermo Fisher Scientific	Cat# A32993
**High-Select**^**TM**^**Fe-NTA Phosphopeptide Enrichment Kit**	Thermo Fisher Scientific	Cat# A32992
**TMT10plex**^**TM**^	Thermo Fisher Scientific	Cat# 90111
**miRNeasy Mini Kit**	QIAGEN	Cat# 217084
**Ambion TURBO DNA-*free***^**TM**^**Kit**	Thermo Fisher Scientific	Cat# AM1907
**Quant-iT RNA Kit**	Thermo Fisher Scientific	Cat# Q33140
**Quant-iT dsDNA High sensitivity Kit**	Thermo Fisher Scientific	Cat# Q33120
**RNA 6000 Nano Assay**	Agilent	Cat# 5067-1511
**Ribo-Zero Gold H/M/R Magnetic Kit**	Illumina	Cat# FC-401-4003
**ScripSeqv2 Kit**	Illumina	Cat# MS-102
**High-Sensitivity DNA Kit**	Agilent	Cat# 5067-4626
**MinElute Gel Extraction Kit**	QIAGEN	Cat# 28604

**Deposited data**		

**Human reference genome**	GENCODE Human (GRCh38)	https://www.gencodegenes.org/human/
**Human reference proteome**	UniProt, downloaded 04.03.2022	https://www.uniprot.org/
**Mouse reference proteome**	UniProt, downloaded 24.05.2022	https://www.uniprot.org/
**3T3-L1 transcriptome data**	Sun et al.^26^ (GEO)	https://www.ncbi.nlm.nih.gov/geo/
**SGBS transcriptome data**	This paper, GEO: GSE245619	https://www.ncbi.nlm.nih.gov/geo/
**Protein mass spectrometry data**	This paper (PRIDE), https://doi.org/10.6019/PXD045767 (SGBS full proteome), PRIDE: https://doi.org/10.6019/PXD045768 (SGBS acetylome), PRIDE: https://doi.org/10.6019/PXD045770 (SGBS phosphoproteome), PRIDE: https://doi.org/10.6019/PXD045772 (3T3-L1 full proteome), PRIDE: https://doi.org/10.6019/PXD045776 (3T3-L1 acetylome), PRIDE: https://doi.org/10.6019/PXD045778 (3T3-L1 phosphoproteome)	https://www.ebi.ac.uk/pride/
**Metabolomics mass spectrometry data**	This paper, Metabolomics workbench/National Metabolomics Data Repository: Study ST002914 (datatrack_id:4335), https://doi.org/10.21228/M8GX42	https://www.metabolomicsworkbench.org/

**Experimental models: Cell lines**		

**SGBS**	Martin Wabitsch, Wabitsch et al.^38^	RRID:CVCL_GS28
**3T3-L1, CL-173^TM^**	American Type Culture Collection	Cat# CL-173; RRID:CVCL_0123

**Software and algorithms**		

**Proteome Discoverer v2.5.0.400**	Thermo Fisher Scientific	N/A
**R v3.6.1**	The R Project for Statistical Computing	https://www.r-project.org/
**uap**	Kampf et al.^31^	https://github.com/yigbt/uap
**FastQC**	Braham Bioinformatics	https://www.bioinformatics.babraham.ac.uk/projects/fastqc/
**FASTXtoolkit**	FASTX-toolkit	http://hannonlab.cshl.edu/fastx_toolkit/
**Trim Galore**	Braham Bioinformatics	bioinformatics.babraham.ac.uk/projects/trim_galore
**HISAT2**	Kim et al.^32^	http://daehwankimlab.github.io/hisat2/
**SAMtools**	Li et al.^33^	http://www.htslib.org/
**HTseq**	Anders et al.^34^	https://htseq.readthedocs.io/en/master/
**SubcellulaRVis**	Watson et al.^35^	http://phenome.manchester.ac.uk/subcellular/
**Analyst v1.7.1**	Sciex	N/A
**Cytoscape v3.7.2**	Shannon et al.^36^	https://cytoscape.org/
**ClueGO v2.5.9**	Bindea et al.^37^	https://apps.cytoscape.org/apps/cluego

**Other**		

**Oasis HLB 1cc, 30 mg, Vac Cartridges**	Waters	Cat# WAT094225
**ZipTips C18**	Millipore	Cat# ZTC18S
**SpeedBead Magnetix Carboxylate Beads**	Cytiva	Cat# GE65152105050250
**Lyophylle Alpha 2-4 LSC**	Christ	Cat# 102142
**UltiMate^TM^ 3000 RS HPLC**	Thermo Fisher Scientific	Cat# IQLAAAGABHFAPBMBEZ
**Thermo Scientific™ Q Exactive™ HF Hybrid Quadrupole-Orbitrap™ Mass Spectrometer**	Thermo Fisher Scientific	Cat# IQLAAEGAAPFALGMBFZ
**TriVersa NanoMate**	Advion	Cat# TR263
**Acclaim PepMap 100 C18 NanoViper column 3 μm,75 μm × 2 cm**	Thermo Fisher Scientific	Cat# 164946
**Acclaim PepMap 100 C18 NanoViper column 3 μm,75 μm × 25 cm**	Thermo Fisher Scientific	Cat# 164569
**Maxtract High Density tubes**	QIAGEN	Cat# 129056
**Qubit 2.0**	Thermo Fisher Scientific	Cat# Q32866
**Agilent 2100 Bioanalyzer**	Agilent	Cat# G2939BA
**HiSeq 2000 Sequencing System**	Illumina	Cat# SY-401-1001
**QTRAP 6500 +**	Sciex	N/A
**Agilent 1290 II infinity UPLC**	Agilent	N/A
**XSelect HSS T3 XP column 2.1 × 150 mm, 2.5 μm, 100 Å**	Waters	Cat# 186006739
**XP VanGuard® Cartridge HSS T3, 2.1 × 5mm, 2.5 μm**	Waters	Cat# 186007884

### Resource availability

#### Lead contact

Further information and requests for resources should be directed to and will be fulfilled by the lead contact, Kristin Schubert (kristin.schubert@ufz.de).

#### Materials availability

This study did not generate new unique materials or reagents.

#### Data and code availability

• Data. All datasets generated in this study have been deposited at GEO, PRIDE or the Metabolomics Workbench, respectively, and are publicly accessible as of the paper’s publication date. DOIs are listed in the key resources table.
• Code. All original code is available in this paper’s supplemental information.
• Any additional information required to reanalyse the data reported in this paper is available from the lead contact upon request.

### Experimental model and study participant details

#### SGBS cell culture

Human SGBS cells (RRID:CVCL_GS28, male) were provided by the laboratory of Prof. Dr. Wabitsch at the University Clinic Ulm and were tested negative for mycoplasma contamination upon receipt. Cells were differentiated according to the standard protocol.^63^ Briefly, SGBS preadipocytes were grown to reach full confluence in basal culture medium, consisting of Dulbecco’s modified Eagle’s F12 (DMEM/F12; Gibco, USA) supplemented with 33 μM biotin (Sigma Aldrich, USA), 17 μM panthothenate (Sigma Aldrich, USA) and 100 U/l penicillin/streptomycin (Sigma Aldrich, USA) and complemented with 10% fetal calf serum (FCS; Gibco, USA). Differentiation was initiated (day 0 to day 4) and maintained (day 4 to day 12) by an exchange to serum-free basal medium supplemented with 0.1 μM cortisol, 0.01 mg/mL apo-transferrin, 0.2 nM triiodothyronine and 20 nM insulin and specifically for the first four days additionally containing 2 μM rosiglitazone, 25 nM dexamethasone and 200 μM 3-isobutyl-1-methylxanthine (all supplements purchased from Sigma Aldrich, USA). New media was provided every four days.

#### 3T3-L1 cell culture

3T3-L1 mouse embryonic fibroblasts were purchased from the American Type Culture Collection (ATCC, CL-173, USA; RRID:CVCL_0123). Cells were cultured in high glucose DMEM (Gibco, USA) supplemented with 10% FCS (basal; Gibco, USA). Differentiation was initiated after cells reached full confluence by changing to basal media complemented with 100 nM insulin, 1 μM dexamethasone, 1 μM rosiglitazone and 0.5 mM 3-isobutyl-1-methylxanthine for three days (day 0 to 3) and maintained (day 3 to 10) with basal media complemented with 100 nM insulin and 1 μM dexamethasone (all supplements purchased from Sigma Aldrich, USA). From day 3 on media was exchanged every two days.

Both cell lines were maintained under 5% CO_2_ at 37°C and 95% humidity. Cell culture experiments for proteomic (SGBS and 3T3-L1) data analysis were performed in triplicates, for transcriptomic (SGBS) data analysis four replicates and for metabolomic analysis (SGBS and 3T3-L1) six replicates were prepared. Accumulated lipids were stained with Oil Red O (Sigma-Aldrich, USA) at initiation of differentiation (day 0), during early (day 6 for SGBS, day 5 for 3T3-L1) and terminal differentiation (day 12 for SGBS, day 10 for 3T3-L1).

### Method details

#### DAPI and Nile Red fluorescence staining

Simultaneous staining of nuclear DNA and lipids using 4′,6-diamidino-2-phenylindole (DAPI; Thermo Fisher Scientific, USA) and Nile Red (Sigma Aldrich, USA) was adapted from Aldridge et al..^78^ In brief, cells were differentiated on 12-well culture dishes, rinsed with phosphate buffered saline (PBS; biowest, France) at the time of harvest (SGBS at day 0, 3, 6, 9 and 12; 3T3-L1 at day 0, 3, 5, 7 and 9) and fixed with 4% paraformaldehyde (Sigma-Aldrich, USA) in PBS for 1 h at room temperature. Fixed cells were washed with PBS and background fluorescence was determined at excitation/emission 360/480 for DAPI and 485/530 for Nile Red in a 9 × 9 spot pattern. Cells were incubated with the staining solution containing 0.1% saponin (Sigma-Aldrich, USA), 1 ng/μL DAPI and 1 ng/μL Nile Red in PBS for 30 min in the dark, rinsed with PBS three times and fluorescence was read out as before. Experiments were performed in six replicates. The background was subtracted from the fluorescence of the stained cells and Nile Red lipid accumulation was corrected to DAPI fluorescence.

#### RNA extraction and sequencing

For sequencing of RNA transcripts, SGBS cells were differentiated as described above and harvested at day 0, after 6 h and days 1, 2, 4, 8 and 12 post adipogenic induction. RNA transcripts were prepared as reported by Schubert et al..^79^ Briefly, cell lysis was performed in 700 μL TRIzol (Thermo Fisher Scientific, USA), RNA was extracted applying the miRNeasy Mini Kit (Thermo Fisher Scientific, USA) in combination with Maxtract High Density tubes (Qiagen, Germany). DNA remains were removed using the Ambion TURBO DNA-free Kit (Thermo Fisher Scientific, USA), RNA was precipitated for cleaning with ethanol and concentrations were determined using the Quant-iT RNA kit (Thermo Fisher Scientific, USA) on a Qubit 2.0 instrument (Thermo Fisher Scientific, USA). RNA integrity was determined applying the RNA 6000 Nano Assay at an Agilent 2100 Bioanalyzer (Agilent, USA) and only samples with an RNA integrity number (RIN) > 8 were used for rRNA depletion of 100 ng total RNA sample using the Ribo-Zero Gold Human/Mouse/Rat Magnetic Kit (Illumina, USA). A sequencing library was generated utilizing the ScripSeqv2 Kit (Illumina, USA), quality controlled with the High Sensitivity DNA Kit (Agilent, USA) at the Agilent 2100 Bioanalyzer system and the library concentration was determined using the Quant-iT dsDNA High Sensitivity kit (Thermo Fisher Scientific, USA) and a Qubit 2.0. Two pools (one per sequencing FlowCell) were generated with 8 ng of each library and selected for fragments of 150-700 bp on a preparative agarose gel in conjunction with the MinElute Gel Extraction Kit (Qiagen, Germany). Paired-end sequencing at a length of 100 bases with a sequencing depth of 160 million read pairs per sample was carried out on a HiSeq2000 device (Illumina, USA).

#### Transcriptomics data analysis and evaluation

Transcriptomic data analysis was performed in analogue to Schubert et al*.*^79^ Very briefly, obtained sequencing reads were transformed into a quantitative presence/absence table using uap.^80^ Files were merged, quality controlled and quality filtered using FastQC^81^ and FASTX-Toolkit.^82^ Adapter trimming was facilitated using Trim Galore^83^ and HISAT2^84^ mapped the reads to the human genome (GRCh38). Alignments were filtered for correct pairing and sorting with the samtools suite^85^ and further processed with HTseq^86^ to generate read counts overlapping annotated genes. GENCODE gene annotation (Release 34) (gencodegenes.org/human) served as reference.

Differential expression was determined with the R package DESeq2.^87^ Sample-to-sample distances and principal component analyses (PCA) were assessed to explore count data quality. Contrasts were calculated compared to day 0 post adipogenic induction. *p*-values were Benjamini-Hochberg adjusted to account for multiple testing and genes were considered as differentially expressed with an adjusted *p*-value < 0.01. All data has been made available via upload to the Gene Expression Omnibus (GEO)^88^ and can be accessed under record number GEO: GSE245619.

#### Proteome and PTM analysis

For the analysis of proteome, acetylome and phosphoproteome, cells were washed with PBS twice and harvested at initiation of differentiation (day 0), during early (day 6 SGBS, day 5 for 3T3-L1) and terminal differentiation (day 12 for SGBS, day 10 for 3T3-L1) using 1 mL lysis buffer consisting of 20 mM HEPES pH 8.0 (Roth, Germany), 9 M urea (Merck, Germany), 1 mM sodium orthovanadate (Sigma-Aldrich, USA), 2.5 mM sodium pyrophosphate (Sigma-Aldrich, USA) and 1 mM β-glycerophosphate (Alfa Aesar, USA). Samples were incubated for 15 min, frozen at -80°C, thawed on ice and protein concentration was determined using the Pierce 660 nm protein assay (Thermo Fisher Scientific, USA).

#### Immunoaffinity purification of acetylated lysines

Immunoaffinity purification (IAP) of acetylated lysines was performed using the PTMScan® Acetyl-Lysine Motif [Ac-K] Kit (Cell Signaling Technology, USA) according to the manufacturer’s protocol with slight adaptations. In short, 1 mg protein was reduced with 5 mM dithiothreitol (DTT; GE Healthcare) for 1 h and alkylated with 10 mM iodacetamid (IAA; Merck, Germany) for 30 min before urea concentration was diluted to <2 M with 20 mM HEPES pH 8.0. For tryptic digestion, trypsin (Promega, USA) was added in a 1:50 (enzyme:protein) ratio and incubated at 37°C overnight. Samples were acidified to 0.1% formic acid (FA; Fluka Honeywell, USA), fatty acids were precipitated by incubation for 15 min on ice and removed by centrifugation at 4000 x g for 15 min. Cleared peptide solution was desalted on Oasis 1cc 30 mg HLB (Waters, USA) cartridges. Briefly, columns were prepared with decreasing concentrations of acidic acetonitrile (ACN; Roth, Germany; 100%, 40%, 3% ACN in 0.1% FA), peptide solution was applied to the columns and washed with 3% ACN in 0.1% FA twice. Bound peptides were eluted with 40% acidified ACN solution and eluates were frozen at −80°C for 1 h to prepare for lyophilization (Alpha 2-4 LSC, Christ, Germany). Peptides were lyophilized to complete dryness and reconstituted in immunoaffinity purification buffer provided by the kit. Peptide solution was cleared by centrifugation at 10.000 x g for 15 min at 4°C and incubated with a quarter of a vial of antibody beads on a rotator at 4°C overnight. Antibody beads were collected by centrifugation and unbound peptide was transferred to new tubes for subsequent preparation of the complementary proteome. Bound peptides were washed in IAP buffer twice, MS grade water three times and the fraction of acetylated peptide was eluted in 0.15% trifluoroacetic acid (TFA; Merck, Germany). Enriched acetyl-peptides were desalted on C18 ZipTips (Millipore, USA), lyophilized as described above and reconstituted in 0.1% FA for MS analysis. 20 μg of unmodified peptides were prepared for MS analysis of the complementary proteome using paramagnetic beads (Cytiva, USA) and two fraction elution using (1) 87% ACN (v/v) with 10 mM ammonium formate at pH 10 and (2) water with 2% DMSO.^89^

#### Phosphopeptide enrichment

Phosphopeptides were enriched as described by Grosskopf et al.,^90^ with additional tandem mass tag (TMT) labelling (TMT10-plex^TM^, Thermo Fisher Scientific, USA). Briefly, 200 μg protein were reduced with 50 mM TCEP (Sigma-Aldrich, USA) for 1 h at 55°C, alkylated with 100 mM IAA for 30 min at room temperature (RT) in the dark and subjected to proteolytic cleavage using a paramagnetic bead approach^89^ and trypsin in a 1:40 (enzyme:protein) ratio at 37°C overnight. TMT reagents (Thermo Fisher Scientific, USA) were dissolved in ACN and incubated with the peptides for 1 h at RT. The labelling reaction was stopped with 5% hydroxylamine and the different TMT labels were combined. Labelled peptides were desalted on beads, eluted and dried completely using a SpeedVac concentrator (Eppendorf, Germany). Phosphopeptides were sequentially enriched using metal affinity chromatography. First, phosphopeptide enrichment was performed using the High-Select™ TiO_2_ Phosphopeptide Enrichment Kit (Thermo Fisher Scientific, USA) as indicated in the manufacturer’s instructions. Unbound peptides were dried and reconstituted for further enrichment. For the second enrichment step, the High-Select™ Fe-NTA Phosphopeptide Enrichment Kit (Thermo Fisher Scientific, USA) was applied according to the manufacturer’s instructions. Enriched phosphopeptide eluates from both steps were combined, dried and reconstituted in 0.1% FA for MS analysis. 20 μg flow through sample of unmodified peptides were prepared as complementary proteome in two fractions.^89^

#### Mass spectrometry peptide data acquisition

All samples were analysed in data-dependent acquisition on a setup consisting of an Ultimate 3000 RS nano ultra-performance liquid chromatography system (Thermo Fisher Scientific, USA) coupled to a Q Exactive HF Hybrid Quadrupole Orbitrap mass spectrometer (Thermo Fisher Scientific, USA) equipped with a TriVersa NanoMate system (Advion, USA). Peptides were trapped on an Acclaim PepMap 100 C18 column, nanoViper, 3 μm, 75 μm × 2 cm column (Thermo Fisher Scientific, USA) and separated for analysis on an analytical reverse-phase Acclaim PepMap 100 C18, nanoViper, 3 μm, 75 μm × 25 cm column (Thermo Fisher Scientific, USA) at a flow rate of 0.3 μL/min. The measurement method was adjusted to the sample and labelling type.

Enriched acetyl-peptides and the corresponding complementary proteome were measured as described above for label-free quantification (LFQ) sample analysis.^89^ Briefly, peptides were eluted over a 170 min three-step linear gradient starting at 4% solvent B (solvent A: 0.1% FA in water; solvent B: 80% ACN/0.1% FA in water), *via* 30% B after 95 min, 55% B after 135 min reaching 99% B after 150 min followed by flushing of the column for 5 min at 99% B and subsequent equilibration to initial conditions. The MS1 spectra at a scan range of 350-1550 *m/z* were acquired in the orbitrap in positive mode with an automatic gain control (AGC) target of 3 × 10^6^ ions at a resolution of 120,000 and a maximum injection time (maxIT) of 100 ms. An isolation window of 1.4 *m/z* was used to select the top 10 precursors for fragmentation *via* collision-induced dissociation (CID) at a normalized collision energy (NCE) of 28. Gained MS2 spectra were recorded at a resolution of 15,000 with a maxIT of 100 ms and AGC target set to 2 × 10^6^. Dynamic exclusion of selected ions was set to 20 sec.

Labelled phosphopeptides and their corresponding background proteome were eluted and measured analogously to Karkossa et al..^91^ In short, phosphopeptides were eluted over a 170 min four-step linear gradient starting at 4% solvent B (solvent A: 0.1% FA in water; solvent B: 80% ACN/0.1% FA in water), *via* 18% B after 78 min, 30% B after 115 min, 55% B after 145 min reaching 99% B after 150 min followed by flushing of the column for 5 min at 99% B and subsequent equilibration to initial conditions. Settings for the acquisition of positive mode full MS scans were the following: scan range of 350-1550 *m/z*, AGC target of 3 × 10^6^ ions, resolution of 120,000 and a maxIT of 150 ms. The top 15 precursor ions were selected for fragmentation applying a 0.7 *m/z* isolation window and an NCE of 34 for CID fragmentation. Fragment ion spectra were recorded at the following settings: resolution of 60,000, maxIT of 150 ms, AGC target of 2 × 10^5^ and a dynamic exclusion of 45 sec.

The collected mass spectrometry proteomic data was deposited to the ProteomeXchange Consortium via the PRIDE^92^ partner repository with the dataset identifier PRIDE: PXD045767 (SGBS full proteome), PXD045768 (SGBS acetylome), PXD045770 (SGBS phosphoproteome), PXD045772 (3T3-L1 full proteome), PXD045776 (3T3-L1 acetylome) and PXD045778 (3T3-L1 phosphoproteome).

#### Protein database searches and data analysis

Raw files were searched using Proteome Discoverer (2.5.0.400, Thermo Fisher Scientific, USA) against the UniProtKB reference proteome of *Homo sapiens* (downloaded 04.03.2022, 78,120 entries) or *Mus musculus* (downloaded 24.05.2022, 55,315 entries), respectively. Carbamidomethylation of cysteines (C) was set as a fixed modification, while oxidation of methionine (M) and N-terminal acetylation were set as variable modifications. For the analysis of samples enriched for acetylated or phosphorylated peptides, acetylation of lysine (K) and phosphorylation of serine (S), threonine (T) and tyrosine (Y) were added as variable modifications, respectively. The false discovery rate (FDR) for peptide, protein and site identifications was set to 0.01, using a target-decoy approach with reversed decoy database. Four missed tryptic cleavages were allowed for enriched acetylated peptide samples to account for hampered cleavage by acetylated lysines, while for all other samples the parameter was set to 2. Proteins were considered identified by at least two peptides, one of which unique. Quantification was based on the intensities of unique and razor peptides. Only proteins and sites found in all three replicates were considered for further analysis. Site abundances were normalized to their corresponding protein abundance.

R v3.6.1 was used for statistical analysis according to the workflow described for the 879 package proteomicsr v1.0.0,^93^ using the following packages: mixOmics (v6.10.9),^94^ ggplot2 (v.3.3.6),^95^ qpcR (v1.4.1),^96^ extrafont (v0.17), corrplot (v0.92), PerformanceAnalytics (v2.0.4), calibrate (v1.7.7), dendsort (v0.3.4), dendextend (v1.15.2), ComplexHeatmap (v2.2.0), RColorBrewer (v1.1.2), limma (v3.42.2),^97^ plyr (v1.8.6),^98^ reshape2 (v1.4.4),^99^ xlsx (v0.6.5),^100^ DEP (v1.8.0),^101^ ggsci (v2.9),^102^ ggpubr (v0.4.0), pheatmap (v1.0.12), circlize (v0.4.13) and ClassDiscovery (v3.4.0). LFQ and TMT derived corrected site intensities were log2 transformed and LFQ data only was variance stabilized and noise imputed in cases 0 out of 3 replicates displayed an intensity. Mean intensities were computed to calculate fold changes (FC) for different intervals during adipogenesis. A two-sided students t-test was used to calculate significant changes with Benjamini-Hochberg adjustment to account for multiple testing.

#### Network inference, enrichment and correlation

A Weighted Gene Correlation Network Analysis (WGCNA) was performed using the corresponding R package WGCNA^103^^,^^104^ on log2 transformed protein as well as corrected acetyl- and phosphosite intensities. Networks were constructed species-wise based on the following parameters – a soft power threshold of 17, minimum/maximum module size of 50/500, a deepsplit value of 2 and a merge cut height of 0.3 – rendering 11 modules each for SGBS and 3T3-L1 data analysis.

Enrichment of subcellular compartments was performed using the tool SubcellulaRVis^105^ and annotation to Gene Ontology Cellular Components (GO CC). A gene set enrichment analysis of acetylated and phosphorylated proteins (FDR <0.05) was performed using the MSigDB (gsea-msigdb.org/gsea/msigdb/collections.jsp) with the respective species *Kyoto Encyclopedia of Genes and Genomes* (KEGG) curated gene set. Resulting enriched pathways with their directions and *p*-values were used for pearson correlation. A KEGG enrichment analysis of proteins carrying modification sites that showed significant differential abundance across adipogenesis, was performed using the application ClueGO v2.5.9^106^ in Cytoscape v3.7.2.^107^ Used settings were the following: minimum cluster size of 3, 4% genes per term, a kappa connectivity score of 0.4 and only pathways with a *p*-value of <0.05 were shown.

#### Metabolite quenching and extraction

Cells were differentiated in 12-well culture dishes and harvested at initiation of differentiation (day 0), during early (day 3 and 6 for SGBS, day 3 and 5 for 3T3-L1) and terminal differentiation (day 9 and 12 for SGBS, day 7 and 10 for 3T3-L1). Intracellular metabolites were extracted using a 1:1:1 methanol:water:chloroform extraction protocol. Briefly, culture media was removed and cells were rinsed with 1 mL ice-cold 0.9% sodium chloride solution. The rinsing solution was removed and the cells metabolism was quenched by adding equal volumes (400 μL) of methanol at −20°C, followed by ice-cold deionized water. Cells were scraped off the culture plate and combined with 400 μL chloroform (−20°C). Samples were vortexed vigorously and kept on a shaker at 4°C for 20 min at 1400 rpm. Cell debris was removed by centrifugation at 16.000 x g and 4°C for 5 min. A fixed volume (300 μL) of the polar upper phase was transferred to new tubes and dried to completeness. Extracted metabolites were reconstituted in 80 μL MS grade water for subsequent measurement.

#### Targeted metabolite quantification

The mass spectral data were acquired in negative mode electrospray ionization on a setup consisting of a QTRAP 6500+ (Sciex, USA) coupled on-line with an Agilent 1290 II infinity UPLC system (Agilent, USA). 10 μL aqueous sample were separated on a XSelect HSS T3 XP column (2.1 × 150 mm, 2.5 μm, 100 Å; Waters, USA) connected to a XP VanGuard® Cartridge (HSS T3, 2.1 × 5mm, 2.5 μm, Waters, USA). Metabolites were eluted over a five-step non-linear gradient starting at 100% solvent A (10 mM tributylamine, 10 mM acetic acid, 5% methanol and 2% 2-propanol pH 7.0 in water) at a flow rate of 0.4 mL/min, reaching 53% solvent B (100% 2-propanol) after 16.5 min for 6 min, at a flow of 0.15 mL/min. The temperatures for autosampler and column oven were set to 5°C and 40°C, respectively. Identification and relative quantification were based on specific MRM transitions (Table S1) and data analysis was performed using the software Analyst® (v1.7.1). Quantified metabolites were normalized to the conditions DAPI fluorescence. This data is available at the NIH Common Fund's National Metabolomics Data Repository (NMDR) website, the Metabolomics Workbench, https://www.metabolomicsworkbench.org where it has been assigned Project ID PR001812. The data can be accessed directly via it's Project at the Metabolomics Workbench: https://doi.org/10.21228/M8GX42.

### Quantification and statistical analysis

The data was generated from at least three independent biological replicates (*n* = 3, for all proteomics analysis; *n* = 4 for transcriptome analysis; *n* = 6 for metabolite analysis). A two-sided students t-test was used to calculate significant changes in gene expression and protein/site abundance, with subsequent Benjamini-Hochberg adjustment to account for multiple testing. Significance of targeted metabolite data was calculated using a two-sided student’s t-test without further adjustment. Analysis and visualizations were performed in R v3.6.1. Data are displayed as mean ± SD and significance is indicated as follows: *p* value ∗ <0.05, ∗∗ <0.01 and ∗∗∗ <0.001. Pathway enrichment data are displayed as median +/− SD. All details can be found in the figure legends as well as the corresponding detailed methods subsection.

## Supplemental information

Document S1. Figures S1–S8 and Table S1

Table S2. Supplementary tables metabolome data, related to Figure 1

Table S3. Supplementary tables proteome data, related to Figures 1, 3, 4 and 5

Table S4. Supplementary tables transcriptome data, related to Figure 1 and 2
