# Supplementary material for: Unveiling the dynamics of acetylation and phosphorylation in SGBS and 3T3-L1 adipogenesis
Source: iScience. 2024 Apr 10;27(6):109711. doi: 10.1016/j.isci.2024.109711 (PMC11152682; doi:10.1016/j.isci.2024.109711)
Supplement: Document S1. Figures S1–S8 and Table S1 [file mmc1.pdf]

iScience, Volume *27*

Supplemental information

Unveiling the dynamics of acetylation and phosphorylation in SGBS and 3T3-L1 adipogenesis

Alix Sarah Aldehoff, Isabel Karkossa, Cornelius Goerdeler, Laura Krieg, Jana Schor, Beatrice Engelmann, Martin Wabitsch, Kathrin Landgraf, Jörg Hackermüller, Antje Körner, Ulrike Rolle-Kampczyk, Kristin Schubert, and Martin von Bergen

Main Supplementary Information

Supplementary Table 1 | Specific MRM transitions used for the quantification of central carbon metabolites.

Analyte	Precursor ion (m/z)	Fragment ion (m/z)	Collision energy (V)
Acetyl-CoA	808	79	-18
cis-Aconitate (Isocitrate)	173	129	-16
Citrate	191	111	-48
Fructose 1,6-bisphosphate	339	79	-38
Fructose 6-phosphate	259	79	-45
Fumarate	115	71	-80
Glucose 6-phosphate	259	79	-55
Glyceraldehyde 3-phosphate	169	79	-25
α-Ketoglutarate	145	101	-50
Malate	133.01	115	-55
Oxaloacetate	131	87	-47
Phosphoenolpyruvate	167	79	-34
3-phosphoglycerate	185	79	-15
Pyruvate	87	43	-36
Succinate	117	99	-32

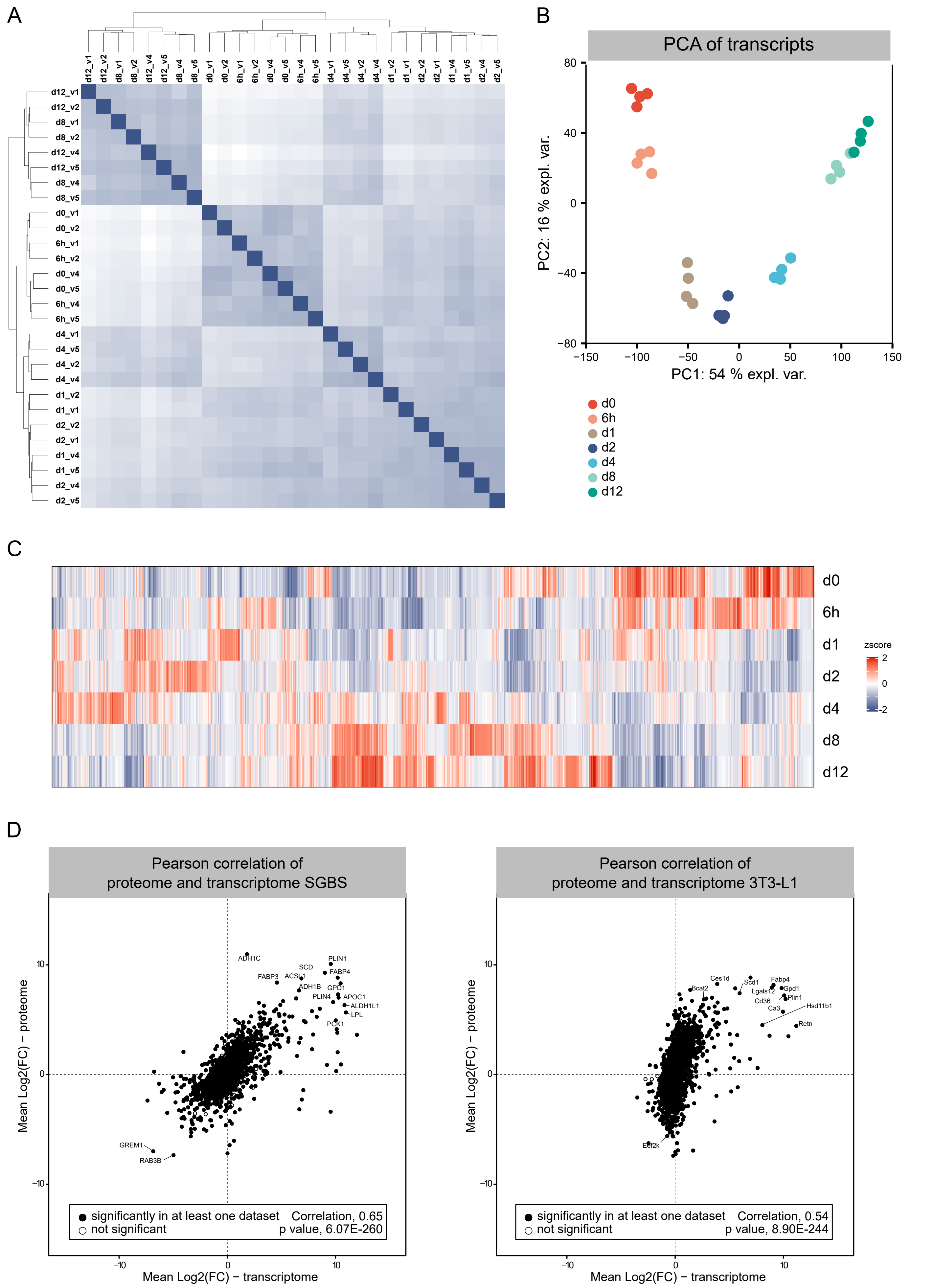

Supplementary Figure 1 | SGBS differentiation RNAseq dataset and correlation to proteomic data. (A) Sample-to-sample distances for transcriptome data of seven timepoints across SGBS adipogenesis. (B) Principal Component Analysis (PCA) of SGBS transcripts at d0/6h/d1/2/4/8/12. (C) Temporal dynamics of the SGBS transcriptome (z -scored) during adipogenesis. (D) Pearson correlation of average log2 fold changes across the complete adipogenesis from proteomic and transcriptomic data in SGBS and 3T3-L1 cells. 3T3-L1 transcriptome data was published by Sun et al. [77].

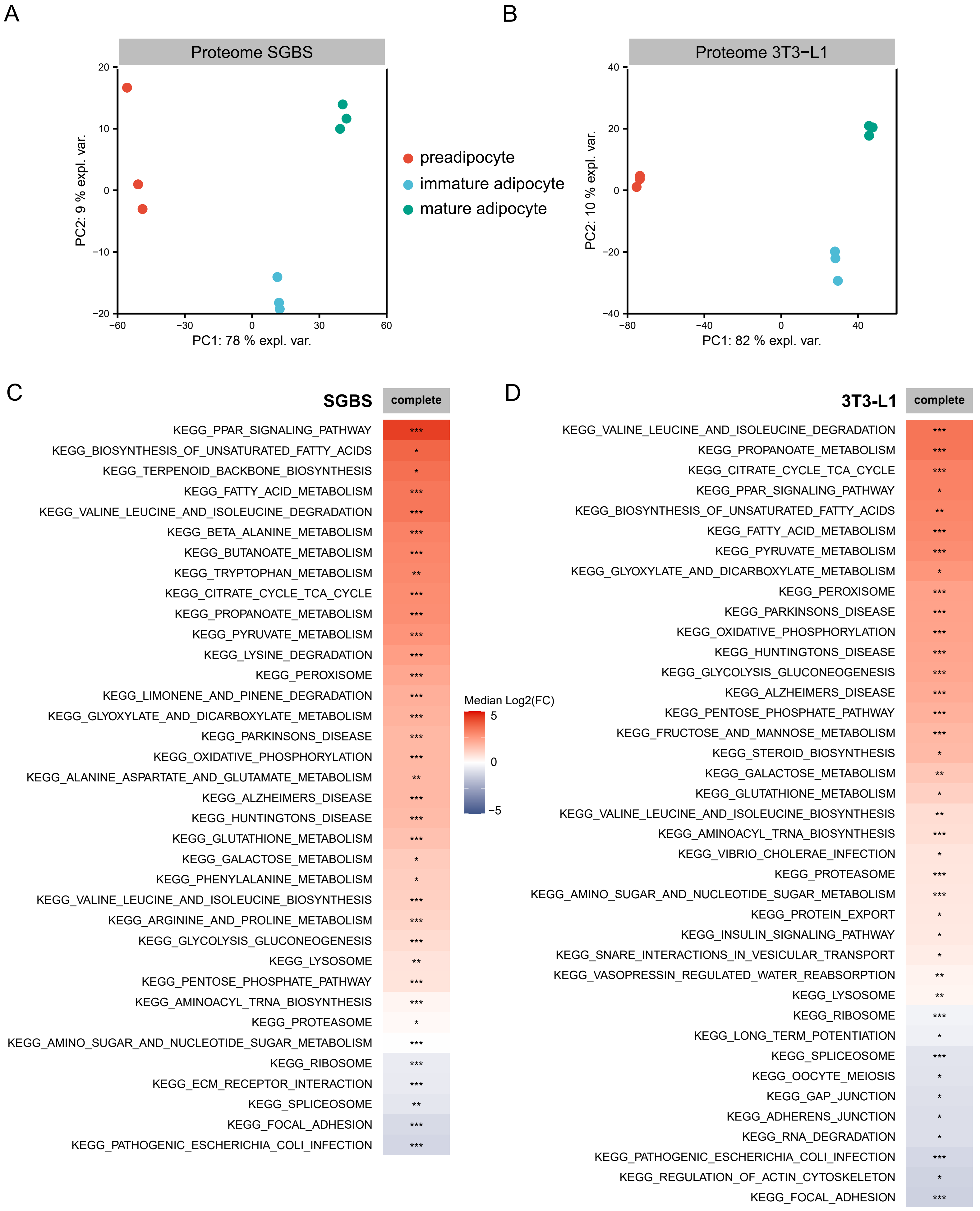

Supplementary Figure 2 | Global proteome alterations across adipogenesis. (A) PCA of the proteomics data of differentiating SGBS and (B) 3T3-L1 adipocytes. (C) KEGG enrichment of the significantly enriched pathways in the subset of differentially abundant proteins in adipogenesis ($p < 0.05$ for mature vs preadipocytes) of SGBS and (D) 3T3-L1 cells. Median log2 fold changes of the pathway across adipogenesis indicates up - or downregulation. Significance is indicated as follows p.adj < 0.05 *, < 0.01 **, < 0.001 ***.

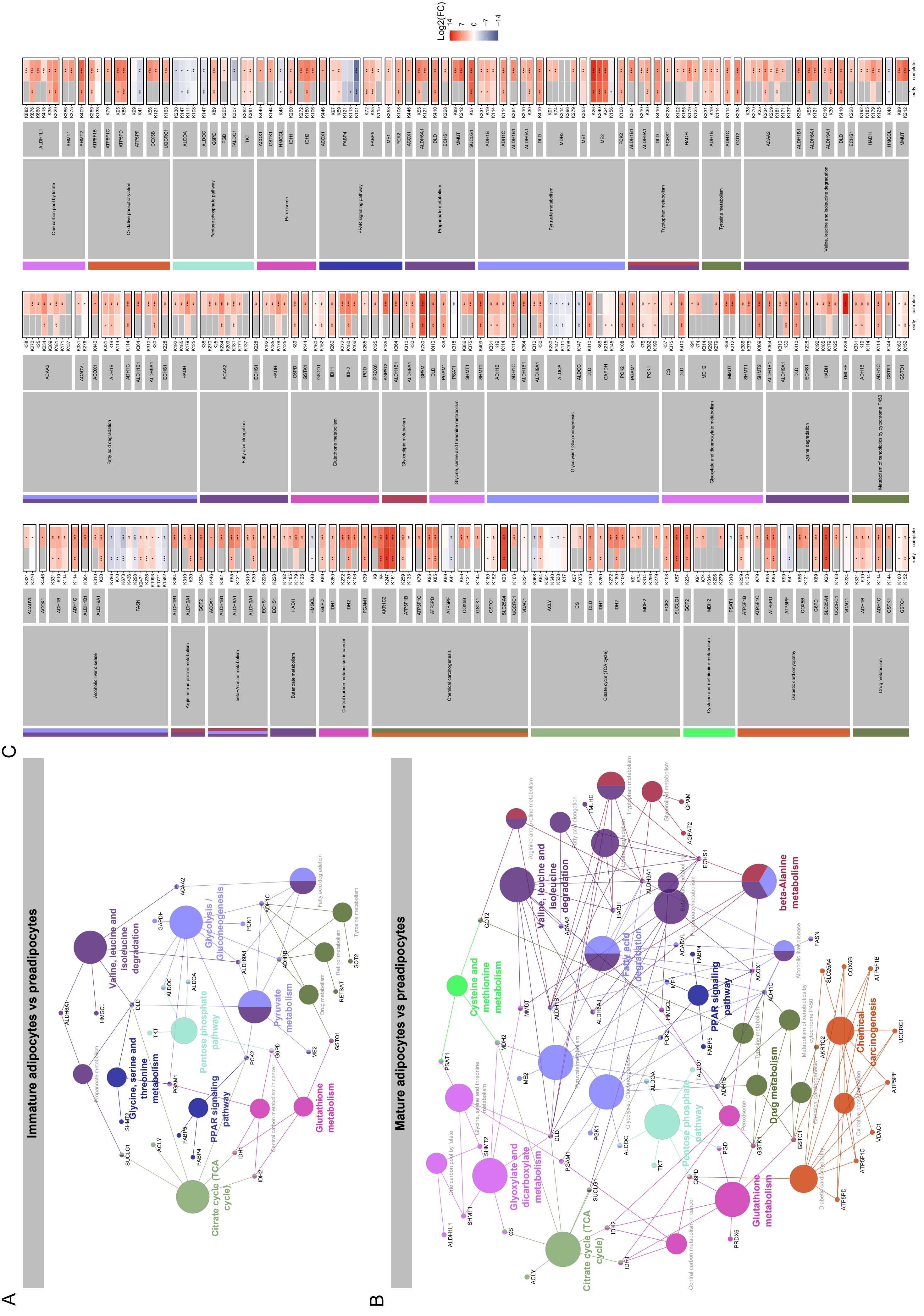

Supplementary Figure 3 | KEGG enrichment of differentially acetylated proteins in SGBS differentiation. (A) KEGG enrichment for differentially acetylated proteins in early and (B) complete adipogenesis. Only significantly enriched pathways are shown and bubble size indicates p value (larger bubble = smaller p value). (C) Average log2 fold changes of all differentially acetylated sites ordered by affiliated KEGG pathway. Significance is indicated as follows p.adj < 0.05 *, < 0.01 **, < 0.001 ***.

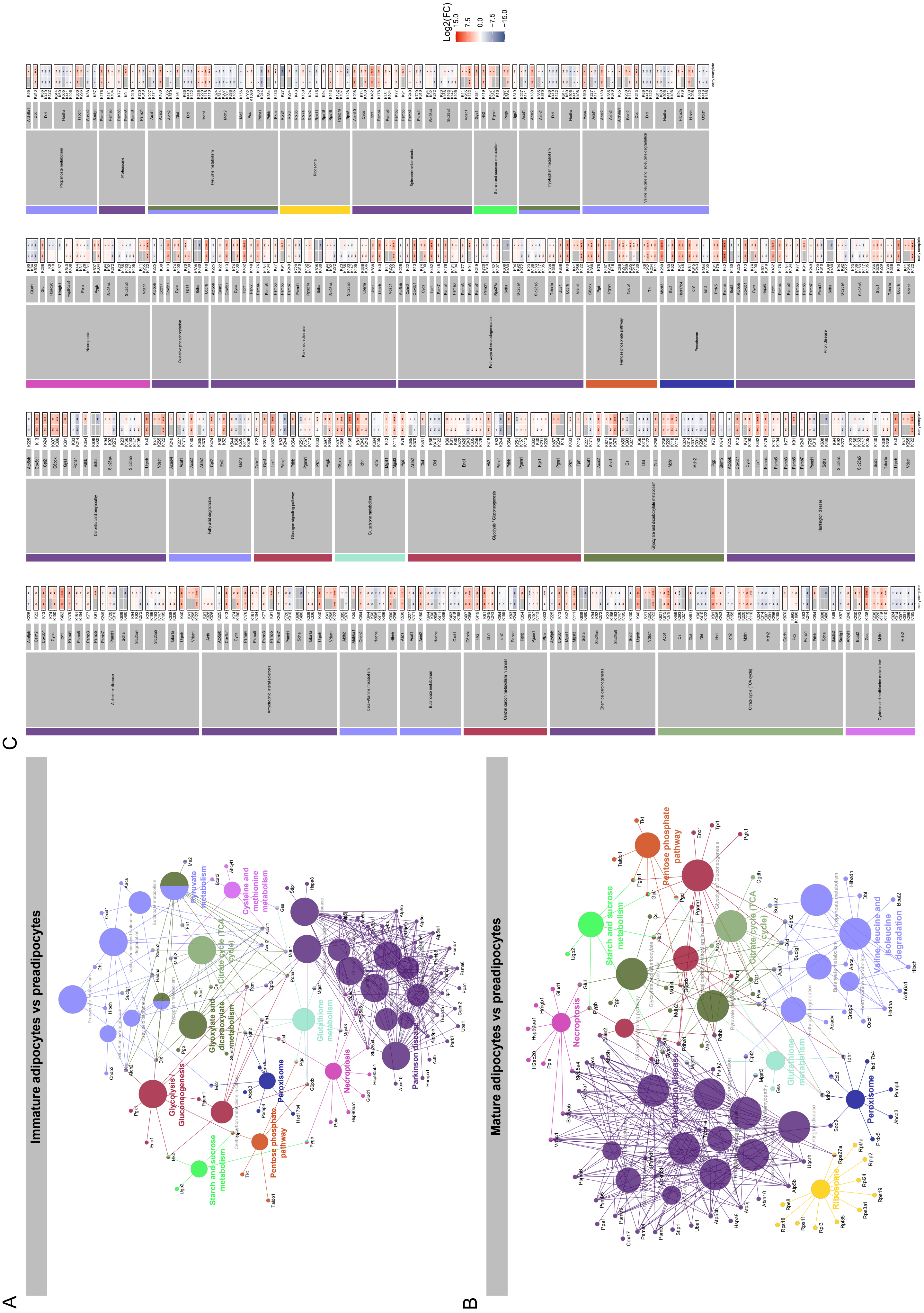

Supplementary Figure 4 | KEGG enrichment of differentially acetylated pr oteins in 3T3 -L1 differentiation. (A) KEGG enrichment for differentially acetylated proteins in early and (B) complete adipogenesis. Only significantly enriched pathway s are shown and bubble size indicates p value (larger bubble = smaller p value). (C) Log2(FC) of all differentially acetylated sites ordered by affiliated KEGG pathway. Significance is indicated as follows p.adj < 0.05 *, < 0.01 **, < 0.001 ***.

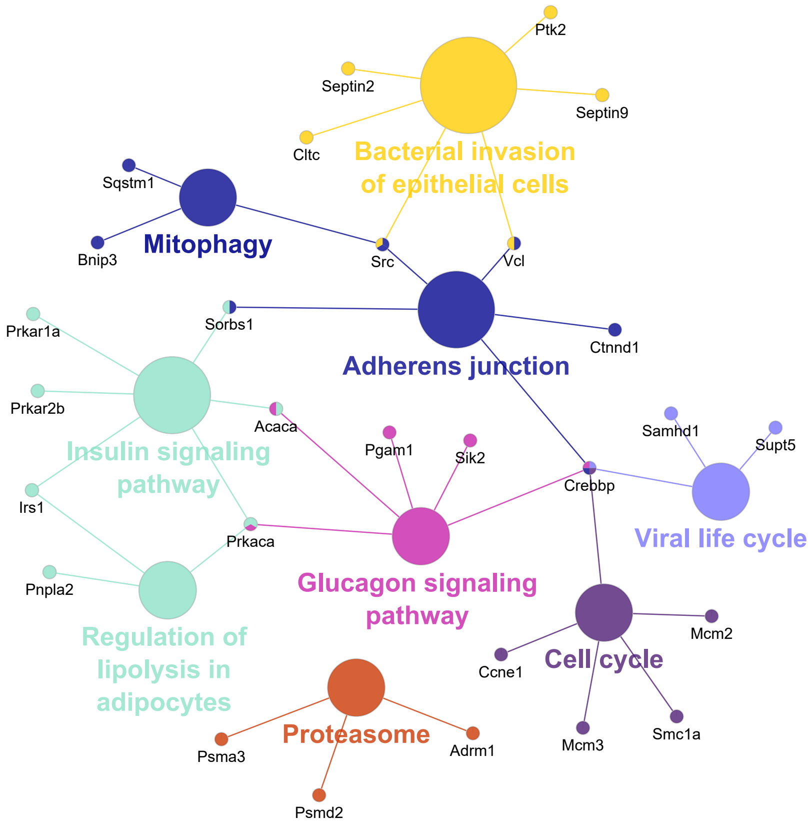

Supplementary Figure 5 | KEGG enrichment of differentially phosphorylated proteins in 3T3-L1 complete differentiation. KEGG enrichment for differentially phosphorylated proteins during complete adipogenesis. Only significantly enriched pathways are shown with associated proteins, and bubble size indicates p value (larger bubble = smaller p value).

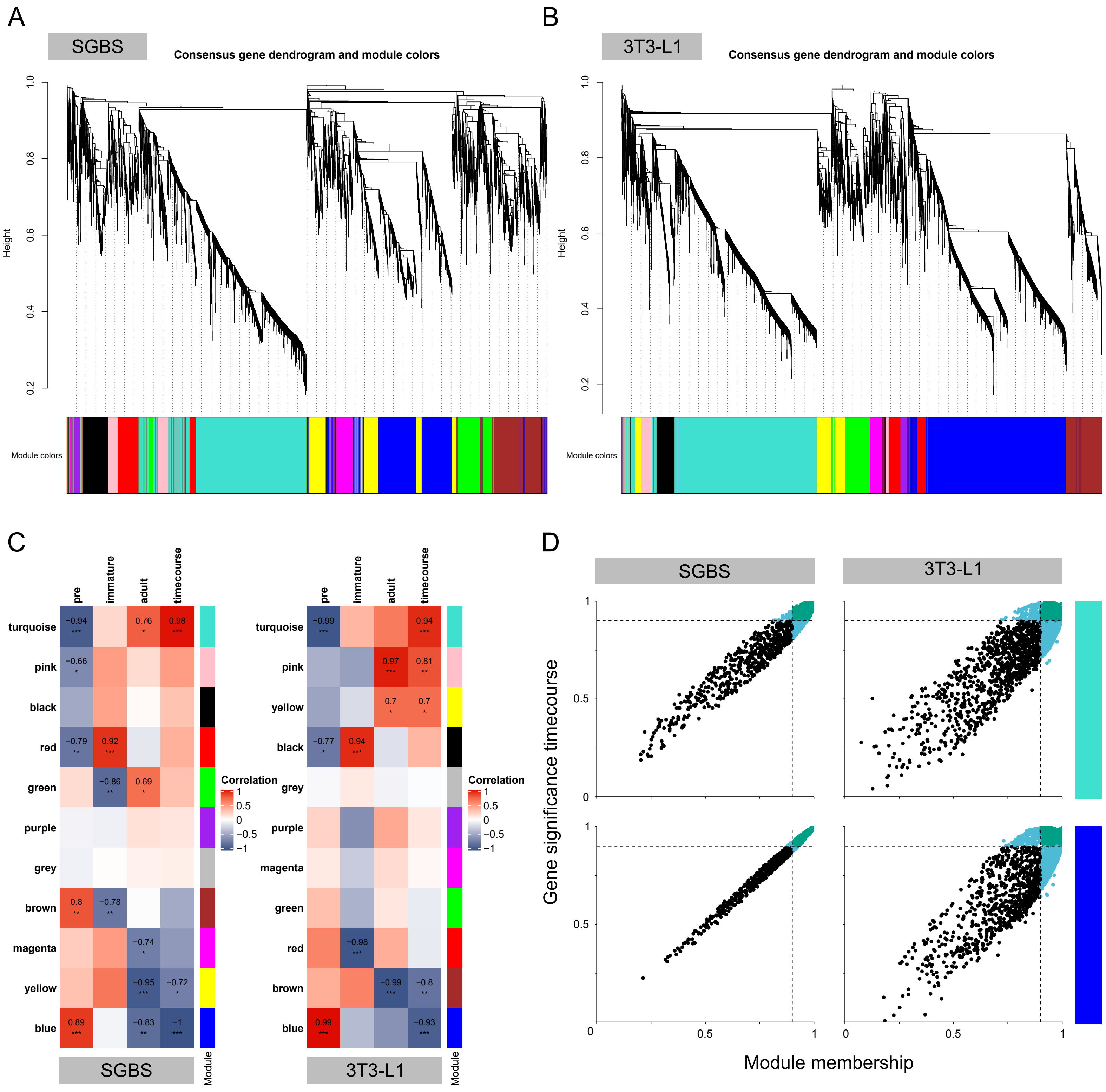

Supplementary Figure 6 | Integrated weighted gene correlation network analysis of proteome, acetylome and phosphoproteome data. (A) Consensus gene dendrogram and module colours for the integrated analysis of the three datasets of adipogenesis in SGBS and (B) 3T3-L1 cells. (C) Module trait correlation matrix of module correlations with the different stages and the whole-time course of adipocyte differentiation. Correlation values are indicated by colour. 11 modules were formed each and are indicated by colour. Significance is indicated as follows p.adj < 0.05 *, < 0.01 **, < 0.001 ***. (D) Determination of key drivers based on trait significance for the time course and module membership with the blue and turquoise module, respectively.

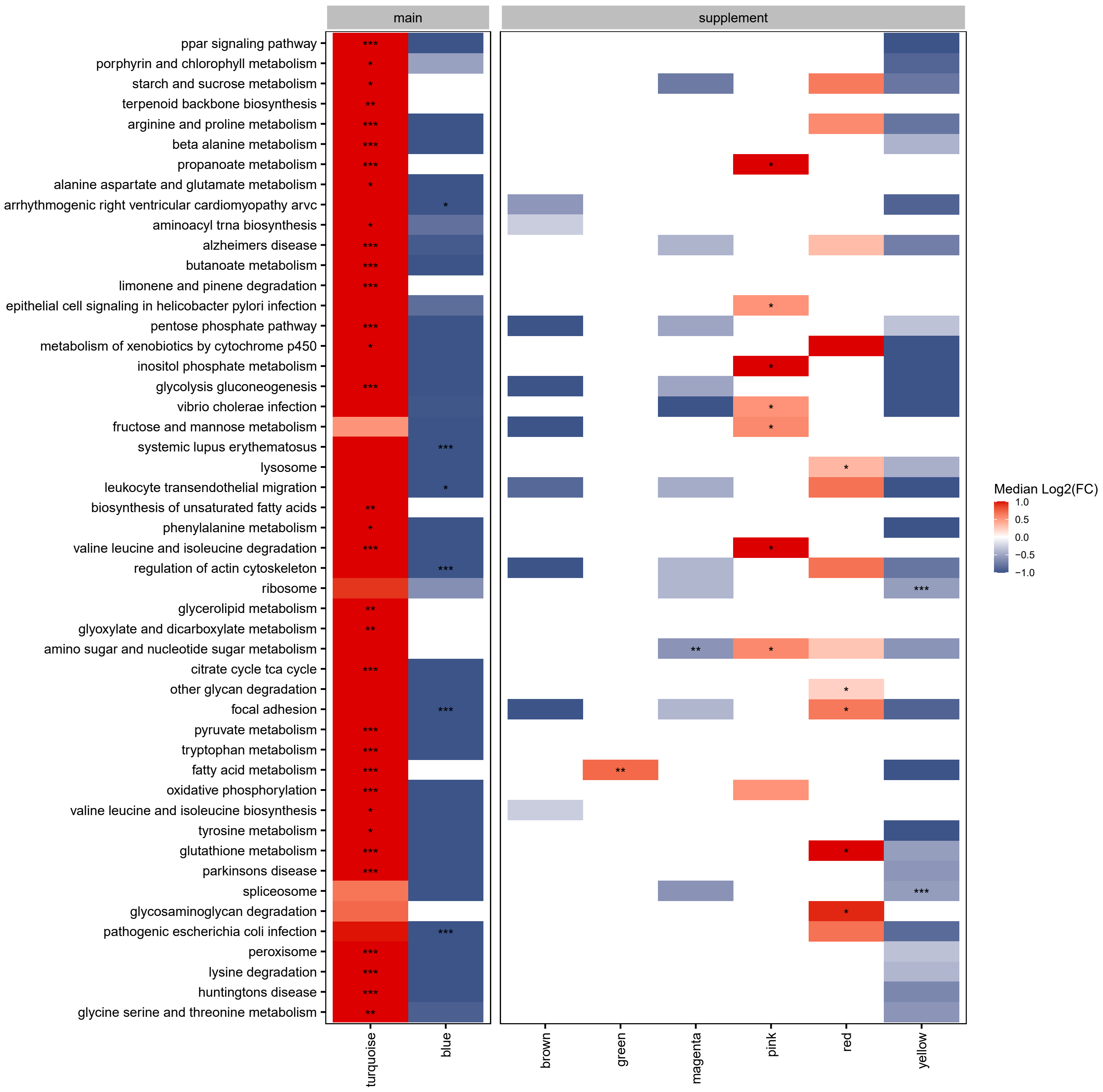

Supplementary Figure 7 | KEGG enrichment of modules from SGBS WGCNA analysis. Significantly enriched pathways across all modules are shown. Median log2 fold changes of the comparison between mature and preadipocytes are indicated by colour, significance as follows p.adj < 0.05 *, < 0.01 **, < 0.001 ***.

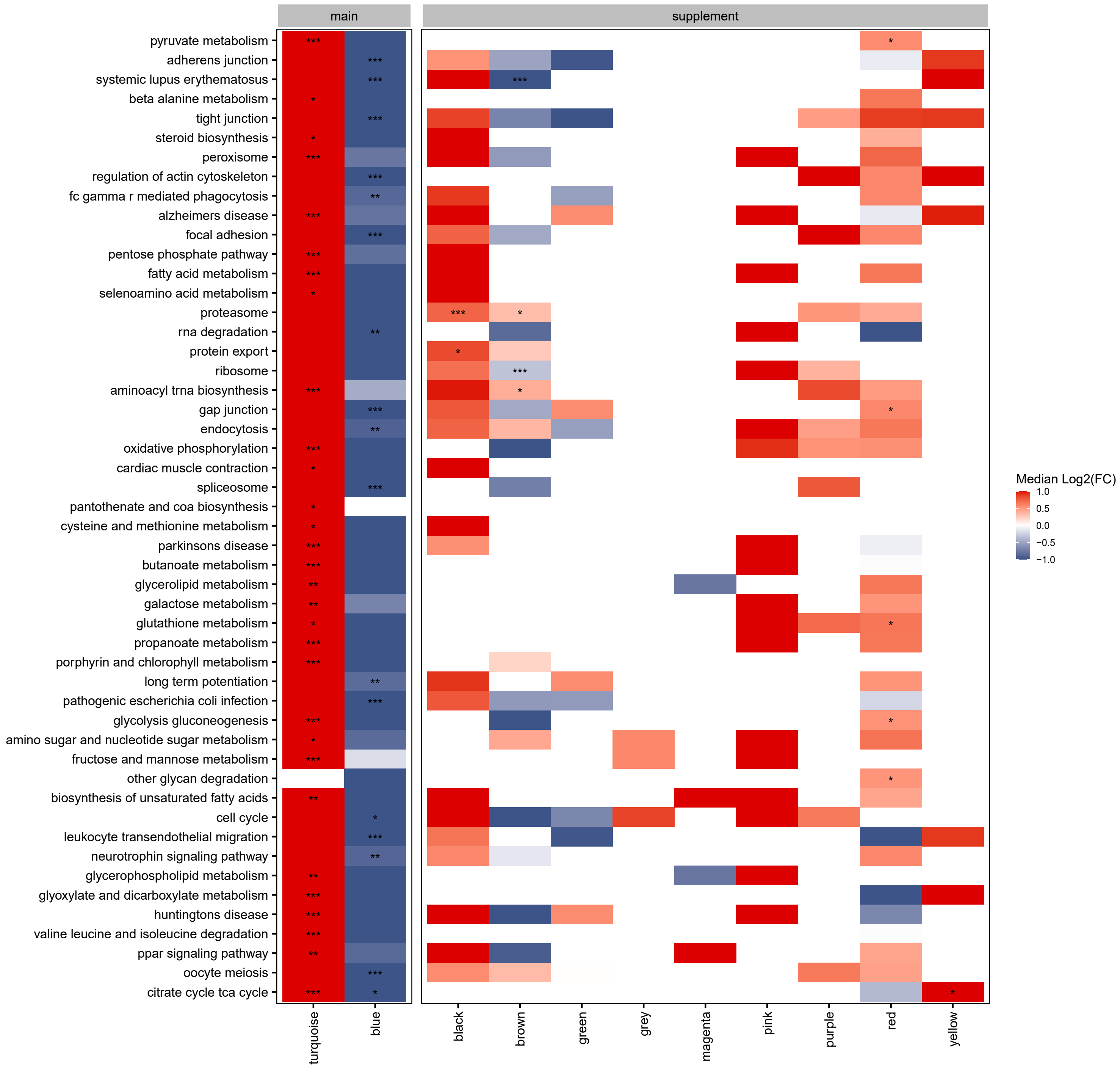

Supplementary Figure 8 | KEGG enrichment of modules from 3T3 -L1 WGCNA analysis. Significantly enriched pathways across all modules are shown. Median log2 fold changes of the comparison between mature and preadipocytes are indicated by colour, significance as follows p.adj < 0.05 *, < 0.01 **, < 0.001 ***.

Code used | Analysis of label-free proteomics data (proteins and sites)

```
---
title: "Analyzing LFQ Intensities"
author: "Isabel Karkossa, Alix Aldehoff"
date: "Februrary 27, 2019"
---

## 1 Set up work space

```{r setup, include=FALSE}
# do not change anything
knitr::opts_chunk$set(echo = TRUE)
knitr::opts_chunk$set(error = TRUE)
# change working directory
knitr::opts_knit$set(root.dir = "//working directory")
```

1.1 Install and Load packages

```{r install_packages}
list.of.packages = c(
  "mixOmics",
  "ggplot2",
  "qpcR",
  "extrafont",
  "corrplot",
  "PerformanceAnalytics",
  "calibrate",
  "dendsort",
  "dendextend",
  "ComplexHeatmap",
  "RColorBrewer",
  "limma",
  "plyr",
  "reshape2",
  "xlsx",
  "DEP",
  "ggsci",
  "ggpubr",
  "ggpubr",
  "pheatmap",
  "circlize",
  "ClassDiscovery"
  )
  new.packages = list.of.packages[!(list.of.packages %in% installed.packages()[,
"Package"])]
  if (length(new.packages))
  install.packages(new.packages)

  new.packages = list.of.packages[!(list.of.packages %in%
installed.packages()[,"Package"])]

  if(length(new.packages)){

    if (!requireNamespace("BiocManager", quietly = TRUE))
      install.packages("BiocManager")

    for (i in new.packages){
      BiocManager::install(i)
    }
  }

# load packages
  for (i in list.of.packages){
    library(i, character.only = TRUE)
```
```

~~~
  }

  #get session info
  sessionInfo()

  # get versions of packages
  for (i in list.of.packages){
    get_version = packageVersion(i)
    print(paste(i, get_version, sep = ": version "))
  }
```

### 1.2 Define paths

This script will produce some output.
Please decide here the base paths to the directories where you want to have it!
Please create these folders!

```{r define_output_paths}
dirsep = "/" # the separator for directories on your system
project_dir = getwd()
plots = "Plots" # subdirectory that will hold all plots in .pdf format
plots_dir = paste(project_dir, plots, sep = dirsep) # leave this like it is!
Rdata = "Rdata" # subdirectory that will hold all intermediate .RData files
Rdata_dir = paste(project_dir, Rdata, sep = dirsep) # leave this like it is!
```

```{r define your font}
#define which font to use for wmf files
windowsFonts("Arial" = windowsFont("Arial"))
define_font = "Arial"
```

```{r import and load fonts and colors}
#use this to install more fonst:
#font_import(pattern = "[A/a]rial")

#have a look which fonts are available
#fonts()
#fonttable()

#load fonts
loadfonts(device = "win")

#define colors for heatmaps with FCs
color_up = ggsci::pal_npg(palette = "nrc")(10)[8]
color_down = ggsci::pal_npg(palette = "nrc")(10)[4]

#define colors for heatmaps with Abundances
color_high = ggsci::pal_npg(palette = "nrc")(10)[8]
color_mid = "white"
color_low = ggsci::pal_npg(palette = "nrc")(10)[4]

barplot(seq(1,10, by = 1), col = ggsci::pal_npg(palette = "nrc")(10))

#create extended color pallette: k colors
#k equals the number of samples that you have
k = 9
npg_colors = get_palette(palette="npg", k=k)
#names(npg_colors) = c(1:k)
barplot(rep(1,k), col=npg_colors, names.arg=names(npg_colors), axes=FALSE)
```

## 2 Load and process data

### 2.1 Import data and define parameters

Put the name of the file with abundance data here. This file should be stored in
the defined project_dir. Be aware that the colnames should contain the sample
~~~

```
names. The first column should contain the protein accessions and these will be
imported as rownames.

```{r define input path of sample table and set parameters}
# define path of sample table with abundance data
sampleTable_file = "Proteins_normalized_intensities.csv" # provide your filename -
don't forget file type!
sampleTable_path = paste(project_dir, sampleTable_file, sep = dirsep) # leave this
like it is!

#import sample table
#sample table has to be imported without header because colnames are not unique yet
sampleTable = read.csv(sampleTable_path, header = FALSE, row.names = 1)

#generate vector of all samples
#should have same length as columns you have
sample_vector = as.vector(t(sampleTable[1, ]))

#generate vector of unique samples
#should have same length as treatments you used
samples = unique(sample_vector)

#add for MaxQuant output
#sample_vector = gsub("LFQ intensity ", "", sample_vector)

#generate a vector of numbers that is just as long as your sample vector
colname_vector_numbers = seq(from = 1, to = length(sample_vector))

# #paste generated numbers to sample vector to get unique entries for each column
# sample_vector_new = paste(sample_vector, colname_vector_numbers, sep = "_")

#use generated vector as new colnames
colnames(sampleTable) = sample_vector

#remove first row that contained the sample names because these are stored in the
header now
sampleTable = sampleTable[-1, ]

#store all accessions in vector
rownames_sampleTable = rownames(sampleTable)

#in case that the columns contain data that are not numeric yet change type to
numeric for all columns
for (i in 1:dim(sampleTable)[2]) {
  sampleTable[, i] = as.numeric(as.character(sampleTable[, i]))
}

#afterwards the rownames are removed and have to be restored by using the generated
rownames vector
rownames(sampleTable) = rownames_sampleTable

#have a look at the intenity distribution of your samples
boxplot(sampleTable, las = 2)


#replace all 0 by NA
sampleTable[sampleTable == 0] = NA
sampleTable[sampleTable == "NaN"] = NA

#give the sample table a new name to not work with the original data
sampleTable_proteome = sampleTable

#have a look at the intenity distribution of your samples
boxplot(sampleTable_proteome, las = 2)

#save original data to Rdata_dir
filename = paste(Rdata_dir, "Proteome_Abundances_cbind.csv", sep = "/")
write.csv(file = filename, x = sampleTable_proteome)
```
```

```
#define number of replicates per protein that are necessary to consider it reliably
identified
#example: reliablereps = 3 --> protein considered reliably identified when it was
identified in 3 of your replicates
reliablereps = 3

#define the name of your control sample --> has to be the same that you gave in the
sample table
controlsample = "t1"

#define, which data to use for the calculation of means and p-values and further
processing
#proteins that were identified with reliablereps over all the treatments -->
"dat_reliable"
#proteins that were identified with reliablereps in at least one treatment -->
"dat_relreps"
datreliable = "dat_relreps"

#set confidence interval for volcano plots
set_conf = 0.995

#import gene information
# the following file has to contain the Accession in the first column and then
columns with Genes and Descriptions for example
sampleTable_file = "genes_proteins.csv" # provide your filename - don't forget file
type!
sampleTable_path = paste(project_dir, sampleTable_file, sep=dirsep) # leave this
like it is!

sampleGenes = read.csv(sampleTable_path, header = TRUE, row.names = 1)

#decide whether to use p-value or adjusted p-value - "pvalue.adj" or "pvalue"
pvalue_decision = "pvalue.adj"

#decide whether to use variance stabilzed data or not
#imputed data use variance stabilized values anyway
vsn_decision = "TRUE"
\`\`\`

*** from here you can knitr because nothing has to be changed ***

### 2.2 Check sample quality

#### 2.2.1 PCA and correlation

\`\`\`{r sample quality testing before removal of outliers}
#generate vector of unique samples
#should have same length as treatments you used
samples = unique(gsub("(.*)_.*", "\\1", colnames(sampleTable_proteome)))

#samples = samples[samples != controlsample]

#perform a PCA for all your replicates per treatment
for (i in samples) {
  #extract all replicates of the treatment
  dat_subset = subset(sampleTable_proteome, select = grepl(pattern = i,

colnames(sampleTable_proteome)))

  #generate a matrix
  PCA.proteins = as.matrix(dat_subset)

  #transpose the matrix
  PCA.proteins = t(PCA.proteins)

  #define PCA.classes to color  replicates in the PCA
  PCA.class = row.names(PCA.proteins)
  PCA.class = gsub("(.*)_.*", "\\1", PCA.class)
  PCA.class
```

```
  #replace all NAs by 0 because PCA can't handle NAs
  PCA.proteins = PCA.proteins[, colSums(is.na(PCA.proteins)) == 0]
  #define how many components to calculate
  PCA.PCA = mixOmics::pca(PCA.proteins, ncomp = ifelse(floor(nrow(PCA.proteins) /
2) < 2, 2, floor(nrow(PCA.proteins) / 2)))

  #plot generated components
  plot(PCA.PCA)

  #plot PCA plot as pdf
  #for pdf never define a family (font)
  plotname = paste("PCA_proteins_initial_", i, ".pdf", sep = "")
  filename = paste(plots_dir, plotname, sep = dirsep)

  pdf(
    file = filename,
    width = 10,
    height = 10,
    pointsize = 12
  )

  #2D Score plot
  mixOmics::plotIndiv(
    PCA.PCA,
    comp = c(1, 2),
    ind.names = TRUE,
    #wenn T, dann kein Zeichen (pch)
    #pch = 16,  #bestimmt Form - in diesem Fall gef?llter Kreis
    #col.per.group = PCA.col,
    group = PCA.class,
    legend = FALSE,
    ellipse = FALSE,
    #ellipse.level = 0.75,
    title = "PCA",
    star = FALSE,
    style = "graphics"
  )

  dev.off()

  #generate correlation matrix for all replicates
  res = cor(dat_subset, use = "complete")

  #plot correlations as bubble plot
  plotname = paste("CorrelationBubbles_initial_", i, ".wmf", sep = "")
  filename = paste(plots_dir, plotname, sep = dirsep)

  win.metafile(
    file = filename,
    width = 10,
    height = 10,
    pointsize = 12,
    family = define_font
  )

  corrplot(
    res,
    type = "upper",
    #order = "hclust",
    tl.col = "black",
    sig.level = 0.05,
    tl.srt = 45
  )

  dev.off()

  #plot correlations as correlation matrix
  plotname = paste("Correlation_initial_", i, ".wmf", sep = "")
  filename = paste(plots_dir, plotname, sep = dirsep)
```

````
  win.metafile(
    file = filename,
    width = 10,
    height = 10,
    pointsize = 12,
    family = define_font
  )

  chart.Correlation(dat_subset, histogram = TRUE, pch = 19)

  dev.off()
}
```

#### 2.2.2 Sample-2-Sample distances

```{r sample2sample description matrix before normalization}
#my_palette = colorRampPalette( rev(brewer.pal(9, "Blues")) )(255)

my_color = ggsci::pal_npg(palette = "nrc")(10)[4]
my_palette = colorRampPalette(colors = c(my_color, "white"))(255)

#transpose data for sample2sample distance plotting
x = t(sampleTable_proteome)

sampleDists = dist(x)
m = as.matrix(dist(x))

plot_file = "Sample2Sample-Distances_initial.wmf"
plot_path = paste(plots_dir, plot_file, sep = dirsep)
win.metafile(file = plot_path,
      width = 15, height = 15,
      pointsize = 10,
      family = define_font)

ht = Heatmap(m,
        col = my_palette,
        color_space = "sRGB",
        #na_col="grey",
        cluster_rows = TRUE,
        cluster_columns= TRUE,
        clustering_distance_columns = "euclidean",
        #clustering_method_columns = "complete",
        column_dend_side="top",
        column_dend_height= unit(3,"cm"),
        column_names_side="top",
        show_row_names = TRUE,
        row_names_side = "left",
        row_names_max_width = unit(10, "cm"),
        column_names_max_height = unit(10, "cm"),
        show_row_dend = TRUE,
        row_dend_width = unit(3,"cm"),
        row_names_gp = gpar(cex = 1.5, fontface = "bold"),
        column_names_gp = gpar(cex = 1.5, fontface = "bold"),
        #column_dend_reorder=c(1,3,4,5,2),
        #row_dend_reorder=F,
        #split = find_rows$k,
        gap = unit(2, "mm"),
        # heatmap_legend_param = list(title = "-Log10(FC)",
        #                              title_gp = gpar(cex = 1, fontface = "bold"),
        #                              color_bar = c("discrete"),
        #                              at = c(0, 0.1, 1.3),
        #                              labels = c("NA", "n.s.", "*")),
        show_heatmap_legend=FALSE)

print(ht)
dev.off()
```

### 2.3 Exclude outliers if necessary
````
````

````
The easiest way to define which samples to exclude, is looking at the numbers at
the end of the sample name. This number should be added as pattern in the following
chunk.

```{r remove identified outliers in the beginning}
# sampleTable_proteome = sampleTable_proteome[,
which(grepl(colnames(sampleTable_proteome),
# pattern = "_9$") == FALSE)]
# sampleTable_proteome = sampleTable_proteome[,
which(grepl(colnames(sampleTable_proteome),
# pattern = "_17$") == FALSE)]

boxplot(sampleTable_proteome, las = 2)
```

2.4 Prepare data for processing

```{r prepare data set proteins}
dat_to_impute = sampleTable_proteome

colnames(dat_to_impute)

accession = rownames(dat_to_impute)
```

2.4.1 Log2-transformation and optionally median normalization

```{r log2_transformation}
boxplot(dat_to_impute, las = 2)

dat_log2 = log2(dat_to_impute)

filename = paste(Rdata_dir, "dat_log2.csv", sep = "/")
write.csv(file = filename,x = dat_log2)

dat_to_impute = dat_log2

boxplot(dat_log2, las = 2)
```


```{r median normalization, eval=FALSE, include=FALSE}

dat_log2 = dat_to_impute

boxplot(dat_log2, las = 2)

dat_to_normalize = dat_log2

nSamples = dim(dat_to_normalize)[2]
sample_vector = colnames(dat_to_normalize)

# create empty vector that will be filled with median values
median_abundance_vector = vector(mode = "expression", length = nSamples)
for (i in 1:nSamples){
  M = na.omit(dat_to_normalize[,i])
  median_abundance_vector[i] = median(M)
}
head(median_abundance_vector)

#Normalize sample data --> every sample gets same median
#alternativ geht auch normalizeMedianAbsValues()
#dat_norm = normalizeBetweenArrays(dat_to_normalize)

#normalize to 0
for (i in 1:nSamples){
  M = na.omit(dat_to_normalize[,i])
  dat_median = median(M)
  M_norm = dat_to_normalize[,i] - dat_median
````
````

````
  assign(x = paste("dat_norm", sample_vector[i], sep = "_"), value = M_norm)
}

dat_norm = do.call(qpcR:::cbind.na, lapply(ls(pattern = "dat_norm_"),
                                  function(x){
                                    get_dat = get(x)
                                    get_dat
                                  }))

dat_norm = as.data.frame(dat_norm)

sample_vector = as.vector(ls(pattern = "dat_norm_"))
sample_vector = gsub("dat_norm_", "", sample_vector)
colnames(dat_norm) = sample_vector
row.names(dat_norm) = row.names(dat_to_normalize)

median_abundance_norm_vector = vector(mode = "expression", length = nSamples)

for (i in 1:nSamples){
  M = na.omit(dat_norm[,i])
  median_abundance_norm_vector[i] = median(M)
}
head(median_abundance_norm_vector)

boxplot(dat_norm, las = 2)

filename = paste(Rdata_dir, "dat_norm.csv", sep = "/")
write.csv(file = filename,x = dat_norm)

dat_to_impute = dat_norm
```

#### 2.4.2 PCA

Do a principle component analysis for your data set after removal of the outliers.
The replicates will be colored based on the tratements.

```{r PCA of complete data set}

#do everything as described in initial PCA
PCA.proteins = as.matrix(dat_to_impute)
PCA.proteins = t(PCA.proteins)

#define PCA.class
#therefore your treatments are used
PCA.class = row.names(PCA.proteins)
PCA.class = gsub("(.*)_.*", "\\1", PCA.class)
PCA.class

PCA.proteins = PCA.proteins[, colSums(is.na(PCA.proteins)) == 0]
PCA.PCA = mixOmics::pca(PCA.proteins, ncomp = floor(nrow(PCA.proteins) / 2))

plot(PCA.PCA)

plotname = paste("PCA_proteins", ".wmf", sep = "")
filename = paste(plots_dir, plotname, sep = dirsep)

win.metafile(
  file = filename,
  width = 10,
  height = 10,
  pointsize = 12,
  family = define_font
)

#2D Score plot
mixOmics::plotIndiv(
  PCA.PCA,
  comp = c(1, 2),
  ind.names = TRUE,
````

```
  #wenn T, dann kein Zeichen (pch)
  #pch = 16,  #bestimmt Form - in diesem Fall gef?llter Kreis
  #col.per.group = PCA.col,
  group = PCA.class,
  legend = FALSE,
  ellipse = FALSE,
  #ellipse.level = 0.75,
  title = "PCA",
  star = FALSE,
  style = "graphics"
)

dev.off()

plotname = paste("PCA_proteins_ellipse", ".wmf", sep = "")
filename = paste(plots_dir, plotname, sep = dirsep)

win.metafile(
  file = filename,
  width = 6.5,
  height = 5,
  pointsize = 12,
  family = define_font
)

#2D Score plot
pI = mixOmics::plotIndiv(
  PCA.PCA,
  comp = c(1, 2),
  ind.names = FALSE,
  point.lwd = 1,
  pch = 19,
  legend.bty = "n",
  #wenn T, dann kein Zeichen (pch)
  #pch = 16,  #bestimmt Form - in diesem Fall gef?llter Kreis
  col.per.group = ggsci::pal_npg(palette = "nrc")(length(unique(PCA.class))),
  group = PCA.class,
  legend = TRUE,
  legend.position = "right",
  size.legend.title = rel(1),
  size.legend = rel(1),
  ellipse = TRUE,
  ellipse.level = 0.75,
  title = "PCA",
  star = FALSE,
  style = "graphics"
)

print(pI)

dev.off()

plotname = paste("PCA_proteins_ellipse", ".svg", sep = "")
filename = paste(plots_dir, plotname, sep = dirsep)

svg(
  file = filename,
  width = 6.5,
  height = 5,
  pointsize = 12,
  family = define_font
)

print(pI)

dev.off()

PCA_ggplot = as.data.frame(PCA.PCA$x)
PCA_ggplot = PCA_ggplot[, 1:2]
PCA_ggplot$Class = gsub("(.*)_.*", "\\1", row.names(PCA_ggplot))
```

```
minPC1 = round_any(min(PCA_ggplot$PC1), 20, f = floor)
maxPC1 = round_any(max(PCA_ggplot$PC1), 20, f = ceiling)
minPC2 = round_any(min(PCA_ggplot$PC2), 20, f = floor)
maxPC2 = round_any(max(PCA_ggplot$PC2), 20, f = ceiling)

plotname = paste("PCA_proteins_ggplot", ".pdf", sep = "")
filename = paste(plots_dir, plotname, sep = dirsep)

pdf(
  file = filename,
  width = 6.5,
  height = 5,
  pointsize = 12
)

gg = ggplot(PCA_ggplot, aes(x = PC1, y = PC2, color = Class)) +
  geom_point(size = 3) +
  # scale_color_npg() +
  scale_color_manual(values = npg_colors) +
  stat_ellipse(level = 0.8) +
  scale_x_continuous(expand = c(0,0), limits = c(minPC1, maxPC1)) +
  scale_y_continuous(expand = c(0,0), limits = c(minPC2, maxPC2)) +
    theme_classic() +
    theme(axis.title.y = element_text(size = rel(1), color = "black", face =
"plain"),
          axis.title.x = element_text(size = rel(1), color = "black", face =
"plain"),
          axis.text.x = element_text(size = rel(1), color = "black", face =
"plain"),
          axis.text.y = element_text(size = rel(1), color = "black", hjust = 1,
face = "plain"),
          axis.line = element_line(colour = "black", size = 0.5),
          axis.ticks = element_line(colour = "black", size = 0.5),
          legend.title = element_text(size = rel(1), face = "bold", color =
"black"),
          panel.spacing = unit(0.5, "cm"),
          plot.margin = margin(t = 0.5, r = 0.5, b = 0.5, l = 0.5, "cm"),
          #panel.border = element_rect(colour = "black", fill=NA, size=1),
          plot.title = element_text(size = rel(1), face = "bold",
                                    color = "black", hjust = 0.5)) +
    ylab(paste("PC2: ", round(PCA.PCA$explained_variance[[2]]*100, digits = 0),
               " % explained variance", sep = "")) +
    xlab(paste("PC1: ", round(PCA.PCA$explained_variance[[1]]*100, digits = 0),
               " % explained variance", sep = "")) +
  labs(color = "Treatment")

print(gg)

dev.off()

plotname = paste("PCA_proteins_ggplot", ".wmf", sep = "")
filename = paste(plots_dir, plotname, sep = dirsep)

win.metafile(
  file = filename,
  width = 6.5,
  height = 5,
  pointsize = 12,
  family = define_font,
)

print(gg)

dev.off()

plotname = paste("PCA_proteins_ggplot", ".svg", sep = "")
filename = paste(plots_dir, plotname, sep = dirsep)

svg(
```

````
  file = filename,
  width = 6.5,
  height = 5,
  pointsize = 12,
  family = define_font,
)

print(gg)

dev.off()

plotname = paste("PCA_proteins_ggplot", ".png", sep = "")
filename = paste(plots_dir, plotname, sep = dirsep)

png(
  file = filename,
  units = "in",
  res = 800,
  width = 6.5,
  height = 5,
  pointsize = 12,
  family = define_font,
)

print(gg)

dev.off()
```

```{r mahalanobis distance}

spca = ClassDiscovery::SamplePCA(t(PCA.proteins))
plot(spca)

round(cumsum(spca@variances)/sum(spca@variances), digits=2)

#We see that we need 8 components in order to explain 70% of the variation in the
data.  Next, we compute
# the Mahalanobis distance of each sample from the center of an
# N-dimensional principal component space. We apply the mahalanobisQC function
using different numbers of components between 2 and 8.

maha2 = mahalanobisQC(spca, 2)
maha4 = mahalanobisQC(spca, 4)
maha8 = mahalanobisQC(spca, 8)

myd = data.frame(maha2, maha4, maha8)

row.names(maha2)[which(maha2$p.value <= 0.05)]

filename = "Mahalanobis_2_Comp.csv"
pathname = paste(Rdata_dir, filename, sep = dirsep)

write.csv(file = pathname, x = maha2)

filename = "Mahalanobis_2_4_8_Comp.csv"
pathname = paste(Rdata_dir, filename, sep = dirsep)

write.csv(file = pathname, x = myd)

```



#### 2.4.3 Double-check sample quality for treatments after removal of outliers

##### 2.4.3.1 PCA and correlation

Here the same is done as in the initial quality check to have a look if more
outliers can be found after removing the obvious ones.
````
````

```{r sample quality testing after removal of outliers}
samples = unique(gsub("(.*)_.*", "\\1", colnames(dat_to_impute)))

#samples = samples[samples != controlsample]

for (i in samples) {
  dat_subset = subset(dat_to_impute, select = grepl(pattern = i,
colnames(dat_to_impute)))

  PCA.proteins = as.matrix(dat_subset)
  PCA.proteins = t(PCA.proteins)

  PCA.class = row.names(PCA.proteins)
  PCA.class = gsub("(.*)_.*", "\\1", PCA.class)
  PCA.class

  #PCA.col = randomColor(as.numeric(as.factor((PCA.class))), luminosity = "bright")

  PCA.proteins = PCA.proteins[, colSums(is.na(PCA.proteins)) == 0]
  PCA.PCA = mixOmics::pca(PCA.proteins, ncomp = ifelse(floor(nrow(PCA.proteins) /
2) < 2, 2,
                                                       floor(nrow(PCA.proteins) /
2)))

  plot(PCA.PCA)

  plotname = paste("PCA_proteins_", i, ".wmf", sep = "")
  filename = paste(plots_dir, plotname, sep = dirsep)

  win.metafile(
    file = filename,
    width = 10,
    height = 10,
    pointsize = 12,
    family = define_font
  )

  #2D Score plot
  mixOmics::plotIndiv(
    PCA.PCA,
    comp = c(1, 2),
    ind.names = TRUE,
    #wenn T, dann kein Zeichen (pch)
    #pch = 16,  #bestimmt Form - in diesem Fall gef?llter Kreis
    #col.per.group = PCA.col,
    group = PCA.class,
    legend = FALSE,
    ellipse = FALSE,
    #ellipse.level = 0.75,
    title = "PCA",
    star = FALSE,
    style = "graphics"
  )

  dev.off()

  res = cor(dat_subset, use = "complete")

  plotname = paste("CorrelationBubbles_", i, ".wmf", sep = "")
  filename = paste(plots_dir, plotname, sep = dirsep)

  win.metafile(
    file = filename,
    width = 10,
    height = 10,
    pointsize = 12,
    family = define_font
  )
```

````
  corrplot(
    res,
    type = "upper",
    #order = "hclust",
    tl.col = "black",
    sig.level = 0.05,
    tl.srt = 45
)

  dev.off()

  plotname = paste("Correlation_", i, ".wmf", sep = "")
  filename = paste(plots_dir, plotname, sep = dirsep)

  win.metafile(
    file = filename,
    width = 10,
    height = 10,
    pointsize = 12,
    family = define_font
)

  chart.Correlation(dat_subset, histogram = TRUE, pch = 19)

  dev.off()
}
```

##### 2.4.3.2 Sample-2-Sample distances

```{r sample2sample description matrix after exclusion of outliers}
#my_palette = colorRampPalette( rev(brewer.pal(9, "Blues")) )(255)

my_color = ggsci::pal_npg(palette = "nrc")(10)[4]
my_palette = colorRampPalette(colors = c(my_color, "white"))(255)

#transpose data for sample2sample distance plotting
x = t(dat_to_impute)

sampleDists = dist(x)
m = as.matrix(dist(x))

plot_file = "Sample2Sample-Distances.wmf"
plot_path = paste(plots_dir, plot_file, sep = dirsep)
win.metafile(file = plot_path,
      width = 15, height = 15,
      pointsize = 10,
      family = define_font)

ht = Heatmap(m,
        col = my_palette,
        color_space = "sRGB",
        #na_col="grey",
        cluster_rows = TRUE,
        cluster_columns= TRUE,
        clustering_distance_columns = "euclidean",
        #clustering_method_columns = "complete",
        column_dend_side="top",
        column_dend_height= unit(3,"cm"),
        column_names_side="top",
        show_row_names = TRUE,
        row_names_side = "left",
        row_names_max_width = unit(10, "cm"),
        column_names_max_height = unit(10, "cm"),
        show_row_dend = TRUE,
        row_dend_width = unit(3,"cm"),
        row_names_gp = gpar(cex = 1.5, fontface = "bold"),
        column_names_gp = gpar(cex = 1.5, fontface = "bold"),
        #column_dend_reorder=c(1,3,4,5,2),
        #row_dend_reorder=F,
````

````
        #split = find_rows$k,
        gap = unit(2, "mm"),
        # heatmap_legend_param = list(title = "-Log10(FC)",
        #                              title_gp = gpar(cex = 1, fontface = "bold"),
        #                              color_bar = c("discrete"),
        #                              at = c(0, 0.1, 1.3),
        #                              labels = c("NA", "n.s.", "*")),
        show_heatmap_legend=FALSE)

print(ht)
dev.off()
```

#### 2.4.4 Plot of identified proteins

```{r plot of identified proteins - replicates}
dat_to_plot = dat_to_impute

dat_count = apply(dat_to_plot, 2, function(x){
  sum(!is.na(x))
})

dat_to_plot_long = as.data.frame(dat_count)
dat_to_plot_long$Sample = rownames(dat_to_plot_long)

max_count = round_any(max(dat_to_plot_long$dat_count), 500, f = ceiling)

dat_to_plot_long$Class = gsub("(.*)_.*", "\\1", row.names(dat_to_plot_long))

plot_file = paste("NumberIdentifiedProteins_Replicates", ".wmf", sep = "")
  plot_path = paste(plots_dir, plot_file, sep = dirsep)
  win.metafile(file = plot_path,
               width = dim(dat_to_plot_long)[1]/3+1, height = 3,
               pointsize = 12,
               family = define_font)

gg = ggplot(data=dat_to_plot_long, aes(x=Sample, y = dat_count, fill = Class)) +
  geom_bar(stat = "identity") +
  # scale_fill_npg() +
  scale_fill_manual(values = npg_colors) +
    scale_y_continuous(limits = c(0, max_count), expand = c(0, 0),
                       breaks = c(0, max_count/2, max_count)) +
    theme_classic() +
    theme(axis.title.y = element_blank(),
          axis.title.x = element_blank(),
          axis.text.x = element_text(size = rel(1), color = "black", face = "bold",
                                     angle = 45, vjust = 1, hjust = 1),
          axis.text.y = element_text(size = rel(1), color = "black", hjust = 1,
face = "bold"),
          axis.line = element_line(colour = "black", size = 1),
          axis.ticks = element_line(colour = "black", size = 1),
          legend.title = element_blank(),
          panel.spacing = unit(0.5, "cm"),
          plot.margin = margin(t = 0.5, r = 0.5, b = 0.5, l = 0.5, "cm"),
          plot.title = element_text(size = rel(1.5), face = "bold",
                                    color = "black", hjust = 0.5)) +
    ggtitle("Number of identified proteins")

print(gg)
dev.off()
```



### 2.5 Identify reliable data
#### 2.5.1 Proteins that were quantified in a certain number of replicates over all
treatments

Split data just keeping proteins that have been quantified in a certain number of
replicates per treatment.
````
````

````
```{r split data proteins}
#generate sample vector
samples = unique(gsub("(.*)_.*", "\\1", colnames(dat_to_impute)))

for (i in samples) {
  #get all replicates of the chosen treatment
  dat_subset = subset(dat_to_impute, select = grepl(pattern = i,
colnames(dat_to_impute)))

  #determine dimensions
  nReps = dim(dat_subset)[2]
  nRows = dim(dat_subset)[1]

  #count NAs per row
  NACounts = apply(dat_subset, 1, function(x) {
    sum(is.na(x))
  })

  #determine in how many replicates the protein was identified
  nCounts = dim(dat_subset)[2] - NACounts

  #extract proteins that were identified in a certain number of replicates
  dat_relreplicates = dat_subset[which(nCounts >= reliablereps), ]

  #store result in environment
  assign(x = paste("RelReplicates", i, sep = "_"), value = dat_relreplicates)

  #store nCounts in original dataframe
  dat_subset$nCounts = nCounts

  #store Accesssions in original dataframe
  dat_subset$Accessions = rownames(dat_subset)

  #store result in environment
  assign(x = paste("Replicates", i, sep = "_"), value = dat_subset)

  #create a vector only containing the nCounts
  nProteins = dat_subset[which(dat_subset$nCounts >= reliablereps), ]
  dim_sample = c(i, dim(nProteins)[1])

  #store result in environment
  assign(x = paste("nProteins", i, sep = "_"), value = nProteins)
}
```
````

The filtered data sets have to be combined to identify the proteins that have been identified for each treatment in a certain number of replicates. Then all others are excluded.

````
```{r exclude proteins}
#generate list of files for further processing
list_of_replicate_files = ls(pattern = "^Replicates_")

#cbind all dataframes from the list
nCounts_df = do.call(cbind, lapply(ls(pattern = "^Replicates"),
                                   function(x) {
                                     get_dat = get(x)
                                     get_dat$nCounts
                                   }))
#format result as dataframe
nCounts_df = as.data.frame(nCounts_df)

#add accessions to dataframe and use them as rownames
nCounts_df$Accessions = rownames(dat_to_impute)
row.names(nCounts_df) = nCounts_df$Accessions

#store result with new name
nCounts_df_complete = nCounts_df
````
````

````
#extract for all the treatments in the dataframe containing only the nCounts the
proteins that were identified in a certain number of replicates
for (i in 1:length(samples)) {
  nCounts_df = nCounts_df[which(nCounts_df[, i] >= reliablereps),]
}

#add the accessions to the nCounts dataframe and use these as rownames
Accessions_nCounts_df = as.data.frame(nCounts_df$Accessions)
colnames(Accessions_nCounts_df) = c("Accessions")
rownames(Accessions_nCounts_df) = Accessions_nCounts_df$Accessions

#match to create a deccision fachto the datar for future filtering of proteins
that were identified in a certain number of replicates for all the treatments
Replicates_nCounts_df = match(nCounts_df_complete$Accessions,
                              Accessions_nCounts_df$Accessions)
Replicates_nCounts_df = as.data.frame(Replicates_nCounts_df)
colnames(Replicates_nCounts_df) = c("Decision_factor")

#cbind all data
summary_replicates = do.call(qpcR:::cbind.na, lapply(ls(pattern = "^Replicates_"),
                                                     function(x) {
                                                       get_dat = get(x)
                                                       get_dat
                                                     }))

#shorten data based on the decision factor
summary_replicates_short =
summary_replicates[!is.na(summary_replicates$Decision_factor),]

#which percentage of proteins was identified in a certain number of replicates over
all the treatments?
percentage_quant_proteins = dim(summary_replicates_short)[1] /
dim(summary_replicates)[1] *
  100
percentage_quant_proteins

#which percentage of proteins was removed?
percentage_removed_proteins = 100 - percentage_quant_proteins
percentage_removed_proteins

#clean workspace
remove(Replicates_nCounts_df)
```

Split data to extract finally the proteins that were identified in a certain number
of replicates over all the treatments.

```{r split data short proteins}
for (i in samples) {
  dat_subset = subset(summary_replicates_short, select = grepl(pattern = i,
colnames(summary_replicates_short)))

  dim(dat_subset)

  assign(x = paste("Replicates_short", i, sep = "_"),
         value = dat_subset)
}
```

Combine final results.

```{r cbind reliable data proteins}
dat_reliable = do.call(cbind, lapply(ls(pattern = "Replicates_short_"),
                     function(x){
                       get_dat = get(x)
                       get_dat
                     }))

filename = paste(Rdata_dir, "dat_reliable_short.csv", sep = dirsep)
write.csv(dat_reliable, file = filename)
````

````
```

#### 2.5.2 Proteins that were quantified in a certain number of replicates for at
least one treatment

```{r cbind data were nCount larger than reliablereps for one treatment}
#generate a list of files
list_of_replicate_files = ls(pattern = "RelReplicates_")

vector_relreps = unique(unlist(do.call(list, lapply(ls(pattern = "RelReplicates_"),
                     function(x){
                       get_dat = get(x)
                       row.names(get_dat)
                     }))))

vector_relreps_df = as.data.frame(vector_relreps)
colnames(vector_relreps_df) = c("Accession")

dat_relreps = merge(vector_relreps_df, dat_to_impute, by.x = "Accession", by.y =
"row.names")
row.names(dat_relreps) = dat_relreps$Accession
dat_relreps = dat_relreps[, -1]

#remove rows that contain only empty cells with NAs
dat_relreps = dat_relreps[rowSums(is.na(dat_relreps)) != ncol(dat_relreps),]

filename = "dat_relreps.csv"
pathname = paste(Rdata_dir, filename, sep = dirsep)
write.csv(pathname, x = dat_relreps)
```

### 2.6 Calculations
#### 2.6.1 Variance stabilization

```{r sample_vector proteins}
#either dat_relreps or dat_reliable are used for calculations
dat_to_calculate = get(datreliable)

sample_vector = unique(gsub("(.*)_.*","\\1", colnames(dat_to_calculate)))
```

Data are background corrected and normalized by variance stabilizing transformation
(vsn). For LFQ data no major changes are expected. For this step proteins with too
many missing values are excluded. Therefore, it is done after filtering for
reliable proteins.

```{r vsn normalization - variance stabilization}
boxplot(dat_to_calculate, las = 2)

# data as summarized experiment are needed (se)

dat_to_vsn = 2^dat_to_calculate

experimental_design = as.data.frame(matrix(ncol = 3, nrow = ncol(dat_to_vsn)))
colnames(experimental_design) = c("label", "condition", "replicate")
experimental_design$label = colnames(dat_to_vsn)
experimental_design$condition = gsub("(.*)_.*","\\1", experimental_design$label)
experimental_design$replicate = sub('.*\\_', '', experimental_design$label)

experimental_design

ncol_vsn = seq(1:ncol(dat_to_vsn))

dat_to_vsn$Names = row.names(dat_to_vsn)

dat_to_se = make_unique(dat_to_vsn, names = "Names", ids = "Names", delim = ";")

dat_to_se$name %>% duplicated() %>% any()

boxplot(dat_to_se[, ncol_vsn], las = 2)
````

````
dat_to_se_final = make_se(proteins_unique = dat_to_se, columns = ncol_vsn,
                          expdesign = experimental_design)

dat_to_se_final_check = get_df_wide(dat_to_se_final)
boxplot(dat_to_se_final_check[, ncol_vsn+1], las = 2)

dat_vsn = normalize_vsn(dat_to_se_final)

plot_normalization(dat_to_se_final, dat_vsn)

plot_detect(dat_to_se_final)
plot_detect(dat_vsn)
```

#### 2.6.2 Imputation

If the proteins with missing values have on average low intensities the data should
be imputed by a left-censored imputation method because they are missing not at
random (MNAR) and are close to the detection limit.

```{r impute data - MNAR}
data_imp = impute(dat_vsn, fun = "MinProb", q = 0.01)

plot_imputation(dat_to_se_final, dat_vsn, data_imp)
```

```{r extract vsn data for further analyses}
dat_to_calculate = get_df_wide(dat_vsn)

row.names(dat_to_calculate) = dat_to_calculate$Names
dat_to_calculate = dat_to_calculate[, -1]
dat_to_calculate = dat_to_calculate[, ncol_vsn]
#colnames(dat_to_calculate) = colnames(sampleTable)

dat_stabilized = dat_to_calculate

filename = "dat_relreps_vsn.csv"
pathname = paste(Rdata_dir, filename, sep = dirsep)

write.csv(x = dat_stabilized, file = pathname)

boxplot(dat_to_calculate, las = 2)
```

```{r choose vsn or not vsn data}

if(vsn_decision == "TRUE") {dat_to_plot = dat_stabilized}
if(vsn_decision == "FALSE") {dat_to_plot = get(datreliable)}

boxplot(dat_to_plot)
dat_to_calculate = dat_to_plot
```


#### 2.6.3 Variance-stabilized data are used for further calculations and imputed
data are stored for later calculations

Count identified proteins in reliable data.

```{r plot of identified proteins - reliable replicates}

dat_count = apply(dat_to_plot, 2, function(x){
  sum(!is.na(x))
})

dat_to_plot_long = as.data.frame(dat_count)
dat_to_plot_long$Sample = rownames(dat_to_plot_long)

max_count = round_any(max(dat_to_plot_long$dat_count), 500, f = ceiling)
````

````
dat_to_plot_long$Class = gsub("(.*)_.*", "\\1", row.names(dat_to_plot_long))

plot_file = paste("NumberIdentifiedProteins_RepliableReplicates", ".wmf", sep = "")
  plot_path = paste(plots_dir, plot_file, sep = dirsep)
  win.metafile(file = plot_path,
               width = dim(dat_to_plot_long)[1]/3+1, height = 3,
               pointsize = 12,
               family = define_font)

gg = ggplot(data=dat_to_plot_long, aes(x=Sample, y = dat_count, fill = Class)) +
  geom_bar(stat = "identity") +
  #scale_fill_npg() +
  scale_fill_manual(values = npg_colors) +
    scale_y_continuous(limits = c(0, max_count), expand = c(0, 0),
                       breaks = c(0, max_count/2, max_count)) +
    theme_classic() +
    theme(axis.title.y = element_blank(),
          axis.title.x = element_blank(),
          axis.text.x = element_text(size = rel(1), color = "black", face = "bold",
                                     angle = 45, vjust = 1, hjust = 1),
          axis.text.y = element_text(size = rel(1), color = "black", hjust = 1,
face = "bold"),
          axis.line = element_line(colour = "black", size = 1),
          axis.ticks = element_line(colour = "black", size = 1),
          legend.title = element_blank(),
          panel.spacing = unit(0.5, "cm"),
          plot.margin = margin(t = 0.5, r = 0.5, b = 0.5, l = 0.5, "cm"),
          plot.title = element_text(size = rel(1.5), face = "bold",
                                    color = "black", hjust = 0.5)) +
    ggtitle("Number of identified proteins")

print(gg)
dev.off()
```

Check for sample quality in reliable data by using another Sample-2-Sample distance
correlation.

```{r sample2sample description matrix of reliable data}
#transpose data for sample2sample distance plotting
x = t(dat_to_calculate)

sampleDists = dist(x)
m = as.matrix(dist(x))

plot_file = "Sample2Sample-Distances_reliable.wmf"
plot_path = paste(plots_dir, plot_file, sep = dirsep)
win.metafile(file = plot_path,
      width = 15, height = 15,
      pointsize = 10,
      family = define_font)

ht = Heatmap(m,
        col = my_palette,
        color_space = "sRGB",
        #na_col="grey",
        cluster_rows = TRUE,
        cluster_columns= TRUE,
        clustering_distance_columns = "euclidean",
        #clustering_method_columns = "complete",
        column_dend_side="top",
        column_dend_height= unit(3,"cm"),
        column_names_side="top",
        show_row_names = TRUE,
        row_names_side = "left",
        row_names_max_width = unit(10, "cm"),
        column_names_max_height = unit(10, "cm"),
        show_row_dend = TRUE,
        row_dend_width = unit(3,"cm"),
````

```
        row_names_gp = gpar(cex = 1.5, fontface = "bold"),
        column_names_gp = gpar(cex = 1.5, fontface = "bold"),
        #column_dend_reorder=c(1,3,4,5,2),
        #row_dend_reorder=F,
        #split = find_rows$k,
        gap = unit(2, "mm"),
        # heatmap_legend_param = list(title = "-Log10(FC)",
        #                             title_gp = gpar(cex = 1, fontface = "bold"),
        #                             color_bar = c("discrete"),
        #                             at = c(0, 0.1, 1.3),
        #                             labels = c("NA", "n.s.", "*")),
        show_heatmap_legend=FALSE)

print(ht)
dev.off()
```

Have a look at NA distribution in reliable data.

```{r heatmap NA distribution}
NA_palette = colorRampPalette(colors = c(my_color, "white"))(10)[c(1,6)]

dat_to_plot = dat_to_calculate

dat_to_plot[!is.na(dat_to_plot)] = 0
dat_to_plot[is.na(dat_to_plot)] = 1

dend_rows = hclust(dist(dat_to_plot)) #can not be used with NAs
dend_cols = dendsort(hclust(dist(t(dat_to_plot))))
dend_cols = color_branches(dend_cols, k = floor(dim(dat_to_plot)[2]/2))

dat_matrix = as.matrix(dat_to_plot)

plot_file = "Heatmap_NA_Distribution.wmf"
plot_path = paste(plots_dir, plot_file, sep = dirsep)
win.metafile(file = plot_path,
             width = dim(dat_to_plot)[2]/3+2, height = 8,
             pointsize = 10,
             family = define_font)

ht = Heatmap(dat_matrix,
             col = NA_palette,
             color_space = "sRGB",
             #na_col="grey",
             cluster_rows = dend_rows,
             cluster_columns= dend_cols,
             clustering_distance_columns = "euclidean",
             #clustering_method_columns = "complete",
             column_dend_side="top",
             column_dend_height= unit(3,"cm"),
             column_names_side="top",
             show_row_names = FALSE,
             #row_names_side = "left",
             #row_names_max_width = unit(10, "cm"),
             column_names_max_height = unit(10, "cm"),
             show_row_dend = FALSE,
             #row_dend_width = unit(3,"cm"),
             #row_names_gp = gpar(cex = 1.5, fontface = "bold"),
             column_names_gp = gpar(cex = 1.5, fontface = "bold"),
             #column_dend_reorder=c(1,3,4,5,2),
             #row_dend_reorder=F,
             #split = find_rows$k,
             #gap = unit(2, "mm"),
             heatmap_legend_param = list(title = "",
                                         title_gp = gpar(cex = 1, fontface =
"bold"),
                                         color_bar = c("discrete"),
                                         at = c(0, 1),
                                         labels_gp = gpar(cex = 1),
                                         labels = c("Value", "NA")),
```

````
             show_heatmap_legend=TRUE)

print(ht)
dev.off()

plot_file = "Heatmap_NA_Distribution.pdf"
plot_path = paste(plots_dir, plot_file, sep = dirsep)
pdf(file = plot_path,
             width = dim(dat_to_plot)[2]/3+2, height = 8,
             pointsize = 10)

print(ht)
dev.off()

```

#### 2.6.4 Calculation of means, p-values, FCs, zscores

Extract data for Calculation.

```{r subset_replicates proteins}
for ( i in sample_vector){
  dat_subset = subset(dat_to_calculate, select = grepl(pattern = i,
colnames(dat_to_calculate)))
  assign(x = paste("Replicates_reliable", i, sep = "_"), value = dat_subset)
}
```

```{r calculation proteins}
#create a list of data to use for calculation
sample_list = ls(pattern = "^Replicates_reliable_")

#extract data of control sample
control_sample = get(paste("Replicates_reliable_", controlsample, sep = ""))

#calculate means for control sample
Mean_ctrl = apply(control_sample, 1, function(x) {
  if (length(na.exclude(x)) >= reliablereps){
    mean(x, na.rm = TRUE)} else NA
})

for (i in sample_list) {
  #get all replicates for treatment
  get_dat = get(i)

  #store accessions
  accessions = row.names(get_dat)

  #define dimensions
  nReps = dim(get_dat)[2]
  nRows = dim(get_dat)[1]

  #count NAs per row
  NACounts = apply(get_dat, 1, function(x) {
    sum(is.na(x))
  })

  #determine in how many replicates protein was quantified
  nCounts = dim(get_dat)[2] - NACounts

  #calculate mean
  Mean = apply(get_dat, 1, function(x) {
    if (length(na.exclude(x)) >= reliablereps){
      mean(x, na.rm = TRUE)} else NA
  })

  #calculate SD
  SD = apply(get_dat, 1, function(x) {
    if (length(na.exclude(x)) >= reliablereps){
      sd(x, na.rm = TRUE)} else NA
````

````
  })

  #calculate FC against control
  FC = Mean - Mean_ctrl

  #summarize replicate data and calculation results
  get_dat = as.data.frame(cbind(get_dat, nCounts, Mean, SD, FC))
  #get_dat = get_dat[which(get_dat$nCounts > 1), ]

  #perform Student's t-test against control
  #get data from replicates and control
  get_dat_ttest = get_dat[, 1:nReps]
  nZero = dim(get_dat_ttest)[1]
  zero_matrix = control_sample
  get_dat_ttest = as.data.frame(cbind(get_dat_ttest, zero_matrix))
  #define dimensions
  nReps_next = nReps + 1
  nCols = dim(get_dat_ttest)[2]
  #determine p-value and return NA if t-test fails

  pvalue = apply(get_dat_ttest, 1, function(x) {
    if (length(na.exclude(x[1:nReps])) >= reliablereps &
        length(na.exclude(x[nReps_next:nCols])) >= reliablereps){
      obj = try(t.test(
      x[1:nReps],
      x[nReps_next:nCols],
      alternative = "two.sided",
      var.equal = TRUE,
      paired = FALSE
    ),
    silent = TRUE)
    if (is(obj, "try-error")){
      return(NA)}
    else {
      return(obj$p.value)}}
    else NA
  })

  #adjust p-values using FDR (same as Benjamini & Hochberg)
  pvalue.adj = p.adjust(pvalue, method = "fdr")

  #add results to summary table
  calculation_results = as.data.frame(cbind(get_dat, pvalue, pvalue.adj))

  zscore_mean = mean(calculation_results$FC, na.rm = TRUE)
  zscore_sd = sd(calculation_results$FC, na.rm = TRUE)

  calculation_results$zscore = (calculation_results$FC - zscore_mean)/zscore_sd

  colnames(calculation_results)[nReps_next:ncol(calculation_results)] =
paste(gsub(i, pattern = "Replicates_reliable_", replacement = ""),
colnames(calculation_results)[nReps_next:ncol(calculation_results)], sep = "_")

  #store summary table in environment
  assign(x = paste("Calculated", i, sep = "_"), value = calculation_results)
}

#clean workspace
remove(Mean_ctrl)
```

Summarize data for all treatments.

```{r cbind data proteins}
summary_calculated_data = do.call(qpcR:::cbind.na, lapply(ls(pattern =
"Calculated_Replicates_"),
                                                          function(x){
                                                            get_dat = get(x)
                                                            get_dat
                                                          }))
````

````
filename = paste(Rdata_dir, "dat_calculated_cbind_proteins.csv", sep = "/")
write.csv(file = filename,x = summary_calculated_data)
```

### 2.7 Extract data

Extract the FCs for all treatments and summarize the values in one dataframe.

```{r extract FCs proteins}
sample_list = ls(pattern = "Calculated_Replicates_")
sample_names = gsub("Calculated_Replicates_", "", sample_list)
sample_names = gsub("reliable_", "", sample_names)

for (i in 1:length(sample_list)) {
  get_dat = get(sample_list[[i]])
  dat_mean = as.data.frame(get_dat[, grepl(colnames(get_dat), pattern = "_FC$")])
  colnames(dat_mean) = sample_names[i]
  accessions = rownames(get_dat)
  row.names(dat_mean) = accessions
  assign(x = paste("Mean", sample_names[i], sep = "_"),
         value = dat_mean)
}

summary_means = do.call(qpcR:::cbind.na, lapply(ls(pattern = "Mean_"),
                                                function(x) {
                                                  get_dat = get(x)
                                                  get_dat
                                                }))

head(summary_means)
boxplot(summary_means, las = 2)

filename = paste(Rdata_dir, "dat_means_cbind_proteins.csv", sep = "/")
write.csv(file = filename, x = summary_means)
```

Export the FCs.

```{r export FCs}
colnames(summary_means)

summary_means_short = summary_means

colnames(summary_means_short)

dat_means_short_proteins = summary_means_short

filename = paste(Rdata_dir, "dat_means_cbind_proteins_short.csv", sep = "/")
write.csv(file = filename,x = summary_means_short)

boxplot(summary_means_short, las = 2)

analyte_to_dataset_mapping_proteins =
as.data.frame(rownames(dat_means_short_proteins))
analyte_to_dataset_mapping_proteins$Dataset = "Proteome"
colnames(analyte_to_dataset_mapping_proteins) = c("Analyte", "Dataset")
```

```{r plot of identified proteins - mean of FCs}
dat_to_plot = summary_means_short

dat_count = apply(dat_to_plot, 2, function(x){
  sum(!is.na(x))
})

dat_to_plot_long = as.data.frame(dat_count)
dat_to_plot_long$Sample = rownames(dat_to_plot_long)

max_count = round_any(max(dat_to_plot_long$dat_count), 500, f = ceiling)
````

```
plot_file = paste("NumberIdentifiedProteins_FCMeans", ".wmf", sep = "")
  plot_path = paste(plots_dir, plot_file, sep = dirsep)
  win.metafile(file = plot_path,
               width = dim(dat_to_plot_long)[1]/3+1, height = 3,
               pointsize = 12,
               family = define_font)

gg = ggplot(data=dat_to_plot_long, aes(x=Sample, y = dat_count, fill = Sample)) +
  geom_bar(stat = "identity")+
  #scale_fill_npg() +
  scale_fill_manual(values = npg_colors) +
    scale_y_continuous(limits = c(0, max_count), expand = c(0, 0),
                       breaks = c(0, max_count/2, max_count)) +
    theme_classic() +
    theme(axis.title.y = element_blank(),
          axis.title.x = element_blank(),
          axis.text.x = element_text(size = rel(1), color = "black", face = "bold",
                                     angle = 45, vjust = 1, hjust = 1),
          axis.text.y = element_text(size = rel(1), color = "black", hjust = 1,
face = "bold"),
          axis.line = element_line(colour = "black", size = 1),
          axis.ticks = element_line(colour = "black", size = 1),
          legend.title = element_blank(),
          panel.spacing = unit(0.5, "cm"),
          plot.margin = margin(t = 0.5, r = 0.5, b = 0.5, l = 0.5, "cm"),
          plot.title = element_text(size = rel(1.5), face = "bold",
                                    color = "black", hjust = 0.5)) +
    ggtitle("Number of identified proteins")

print(gg)
dev.off()
```

Export FCs and adjusted p-values in a format that works for IPA as well as for all other further analysis steps. Furthermore, a table is created that summarizes the counts of significant changes (up and down).

```
```{r extract protein dat for IPA}
sample_list = ls(pattern = "Calculated_Replicates_")
sample_names = gsub("Calculated_Replicates_", "", sample_list)
sample_names = gsub("reliable_", "", sample_names)
sample_names_mean = paste(sample_names)
sample_names_pvalue = paste(sample_names, "pvalue", sep = "_")

for (i in 1:length(sample_list)) {
  get_dat = get(sample_list[[i]])
  #extract FCs and p-values
  #change this to pvalue.adj if you want the adjusted p-values
  #change this to pvalue if you want the NOT adjusted p-values
  #dat_mean = as.data.frame(cbind(get_dat$FC, get_dat$pvalue))
  dat_mean = as.data.frame(cbind(get_dat[, grepl(colnames(get_dat), pattern =
"_FC$")],
                                 get_dat[, grepl(colnames(get_dat), pattern =
paste("_",

pvalue_decision,

"$", sep = ""))]))

  #count significantly up/down regulated proteins
  dat_up = dim(dat_mean[which(dat_mean[, 2] < 0.05 &
                                dat_mean[, 1] > 0),])[1]
  dat_down = dim(dat_mean[which(dat_mean[, 2] < 0.05 &
                                  dat_mean[, 1] < 0),])[1]
  dat_not = dim(dat_mean[which(dat_mean[, 2] >= 0.05),])[1]
  dat_sum = dat_up + dat_down + dat_not

  #summarize data in dataframe and save dataframe
```
```

```
  dat_count_summary = as.data.frame(cbind(sample_names[i], dat_up, dat_not, 
dat_down, dat_sum))
  colnames(dat_count_summary) = c("Treatment", "up", "none", "down", "sum")
  assign(x = paste("RegulationCount", sample_names[i], sep = "_"),
         value = dat_count_summary)

  #store data in environment
  colnames(dat_mean) = c(sample_names_mean[i], sample_names_pvalue[i])
  accessions = rownames(get_dat)
  row.names(dat_mean) = accessions
  assign(x = paste("IPA", sample_names[i], sep = "_"),
         value = dat_mean)
}

#summarize FCs and p-values for all treatments
summary_IPA = do.call(qpcR:::cbind.na, lapply(ls(pattern = "IPA_"),
                                              function(x) {
                                                get_dat = get(x)
                                                get_dat
                                              }))

#remove all rows that contain only NA and save data
summary_IPA[summary_IPA == "NaN"] = NA
summary_IPA = summary_IPA[rowSums(is.na(summary_IPA)) != ncol(summary_IPA),]

filename = paste(Rdata_dir, paste("dat_IPA_cbind_proteins_", pvalue_decision, 
".csv", sep = ""), sep = "/")
write.csv(file = filename, x = summary_IPA)

filename = paste(Rdata_dir, paste("dat_IPA_cbind_proteins_", pvalue_decision, 
".xlsx", sep = ""), sep = "/")
write.xlsx(file = filename, x = summary_IPA)

#export FCs and p-values together with gene names
summary_IPA_genes = merge(summary_IPA, sampleGenes, by = "row.names", all.x = TRUE)
rownames(summary_IPA_genes) = summary_IPA_genes$Row.names
summary_IPA_genes = summary_IPA_genes[, -1]

filename = paste(Rdata_dir, paste("dat_IPA_cbind_proteins_", pvalue_decision, 
"_genes.csv", sep = ""), sep = "/")
write.csv(file = filename,x = summary_IPA_genes)

filename = paste(Rdata_dir, paste("dat_IPA_cbind_proteins_", pvalue_decision, 
"_genes.xlsx", sep = ""), sep = "/")
write.xlsx(file = filename,x = summary_IPA_genes)

#summarize regulation counts for all treatments and save them
summary_CalculationCount = do.call(rbind, lapply(ls(pattern = "RegulationCount_"),
                                                 function(x) {
                                                   get_dat = get(x)
                                                   get_dat
                                                 }))

filename = paste(Rdata_dir, paste("dat_RegulationCount_proteins_", pvalue_decision, 
".csv", sep = ""), sep = "/")
write.csv(file = filename, x = summary_CalculationCount)
```

```{r plot stacked bar plots of regulation counts}
sample_dat_proteins = summary_CalculationCount

sample_dat_proteins = sample_dat_proteins[sample_dat_proteins$Treatment != 
controlsample, ]

treatment_vector = as.character(sample_dat_proteins$Treatment)

sample_dat_proteins$Treatment = treatment_vector

for (i in 2:ncol(sample_dat_proteins)){sample_dat_proteins[,i] = 
as.numeric(as.character(sample_dat_proteins[,i]))}
```

```
sample_dat_proteins$up =
as.numeric(sample_dat_proteins$up/sample_dat_proteins$sum)*100
sample_dat_proteins$none = sample_dat_proteins$none/sample_dat_proteins$sum*100
sample_dat_proteins$down = sample_dat_proteins$down/sample_dat_proteins$sum*100
sample_dat_proteins$sum = sample_dat_proteins$up + sample_dat_proteins$none +
sample_dat_proteins$down

samples = unique(sample_dat_proteins$Treatment)

sample_dat_proteins = sample_dat_proteins[, -5]

sample_dat_proteins_reshape = melt(sample_dat_proteins, id.var = "Treatment")

sample_dat_proteins_reshape$Treatment =
as.character(sample_dat_proteins_reshape$Treatment)
sample_dat_proteins_reshape$variable =
as.character(sample_dat_proteins_reshape$variable)

  get_dat = sample_dat_proteins_reshape
  get_dat = get_dat[order(get_dat$variable), ]

  get_dat$variable = factor(get_dat$variable, levels = c("up", "none", "down"))

  ggplot_title = "Significantly altered proteins"

  plotfile = paste(plots_dir, paste("RegulationCount_Stacked", ".wmf", sep = ""),
sep=dirsep)
  win.metafile(file = plotfile,
      width = length(samples)/2 + 4, height = 2,
      pointsize = 12,
      family = define_font)

  #get_dat$Treatment = factor(x = get_dat$Treatment, levels = c("d6", "d12"))
#order data as desired
  gg = ggplot(data = get_dat, aes(x = Treatment, y = value, fill = variable)) +
  geom_bar(stat = "identity") +
    scale_fill_manual(values = c(color_up, "grey", color_down))+
    scale_y_continuous(expand = c(0,0)) +
    theme_classic() +
    theme(axis.title.y = element_text(size = rel(1), face = "bold", color =
"black"),
            axis.title.x = element_blank(),
            axis.text.x = element_text(size = rel(1), color = "black", face =
"bold", angle = 45,
                                       vjust = 1, hjust = 1),
            axis.text.y = element_text(size = rel(1), color = "black", hjust = 1,
face = "bold"),
          axis.line = element_line(colour = "black", size = 1),
          axis.ticks = element_line(colour = "black", size = 1),
          legend.title = element_blank(),
          #plot.margin = margin(1,1,1,1, "cm"),
          plot.title = element_text(size = rel(1), face = "bold", color = "black",
hjust = 0.5)) +
    ggtitle(ggplot_title) +
    ylab("Percent Proteins")

  print(gg)

  dev.off()

  plotfile = paste(plots_dir, paste("RegulationCount_Stacked", ".pdf", sep = ""),
sep=dirsep)
  pdf(file = plotfile,
      width = 3, height = 2,
      pointsize = 12)

   print(gg)

  dev.off()
```

````
```


```{r extract z-score data}
sample_list = ls(pattern = "Calculated_Replicates_")
sample_names = gsub("Calculated_Replicates_", "", sample_list)
sample_names = gsub("reliable_", "", sample_names)
sample_names_mean = paste(sample_names)
sample_names_pvalue = paste(sample_names, "pvalue", sep = "_")

for (i in 1:length(sample_list)) {
  get_dat = get(sample_list[[i]])
  #extract FCs and p-values
  #change this to pvalue.adj if you want the adjusted p-values
  #change this to pvalue if you want the NOT adjusted p-values
  #dat_mean = as.data.frame(cbind(get_dat$zscore, get_dat$pvalue))
  dat_mean = as.data.frame(cbind(get_dat[, grepl(colnames(get_dat), pattern =
"_zscore$")],
                                 get_dat[, grepl(colnames(get_dat), pattern =
paste("_",

pvalue_decision,

"$", sep = ""))]))

  #count significantly up/down regulated proteins
  dat_up = dim(dat_mean[which(dat_mean[, 2] < 0.05 &
                              dat_mean[, 1] > 0),])[1]
  dat_down = dim(dat_mean[which(dat_mean[, 2] < 0.05 &
                                dat_mean[, 1] < 0),])[1]
  dat_not = dim(dat_mean[which(dat_mean[, 2] >= 0.05),])[1]
  dat_sum = dat_up + dat_down + dat_not

  #summarize data in dataframe and save dataframe
  dat_count_summary = as.data.frame(cbind(sample_names[i], dat_up, dat_not,
dat_down, dat_sum))
  colnames(dat_count_summary) = c("Treatment", "up", "none", "down", "sum")
  assign(x = paste("RegulationCount", sample_names[i], sep = "_"),
         value = dat_count_summary)

  #store data in environment
  colnames(dat_mean) = c(sample_names_mean[i], sample_names_pvalue[i])
  accessions = rownames(get_dat)
  row.names(dat_mean) = accessions
  assign(x = paste("IPA", sample_names[i], sep = "_"),
         value = dat_mean)
}

#summarize FCs and p-values for all treatments
summary_zscore = do.call(qpcR:::cbind.na, lapply(ls(pattern = "^IPA_"),
                                             function(x) {
                                               get_dat = get(x)
                                               get_dat
                                             }))

#remove all rows that contain only NA and save data
summary_zscore[summary_zscore == "NaN"] = NA
summary_zscore = summary_zscore[rowSums(is.na(summary_zscore)) <
ncol(summary_zscore), ]

filename = paste(Rdata_dir, paste("dat_zscore_", pvalue_decision, ".csv", sep =
""), sep = "/")
write.csv(file = filename, x = summary_zscore)

filename = paste(Rdata_dir, paste("dat_zscore_", pvalue_decision, ".xlsx", sep =
""), sep = "/")
write.xlsx(file = filename, x = summary_zscore)

#export FCs and p-values together with gene names
````

~~~~
summary_IPA_genes = merge(summary_zscore, sampleGenes, by = "row.names", all.x =
TRUE)
rownames(summary_IPA_genes) = summary_IPA_genes$Row.names
summary_IPA_genes = summary_IPA_genes[, -1]

filename = paste(Rdata_dir, paste("dat_zscore_", pvalue_decision, "_genes.csv", sep
= ""), sep = "/")
write.csv(file = filename,x = summary_IPA_genes)

filename = paste(Rdata_dir, paste("dat_zscore_", pvalue_decision, "_genes.xlsx",
sep = ""), sep = "/")
write.xlsx(file = filename,x = summary_IPA_genes)

#summarize regulation counts for all treatments and save them
summary_CalculationCount = do.call(rbind, lapply(ls(pattern = "RegulationCount_"),
                                                 function(x) {
                                                   get_dat = get(x)
                                                   get_dat
                                                 }))

filename = paste(Rdata_dir, paste("dat_RegulationCount_proteins_zscores_",
pvalue_decision, ".csv", sep = ""),
                 sep = "/")
write.csv(file = filename, x = summary_CalculationCount)
```

### 2.8 Volcano Plots
#### 2.8.1 Volcano Facets

```{r volcano facets, echo=FALSE, message=FALSE, warning=FALSE, paged.print=FALSE}
#choose data set
sample_dat_FC_pvalue = summary_IPA

#exclude control because it contains only 1 and 0 values
sample_dat_FC_pvalue = sample_dat_FC_pvalue[,
which(grepl(colnames(sample_dat_FC_pvalue), pattern = controlsample) == FALSE)]

#create sample vector
sample_vector = unique(gsub(
  colnames(sample_dat_FC_pvalue),
  pattern = "_pvalue",
  replacement = ""
))

#bring data to long format, which is necessary for ggplot
for (j in sample_vector) {
  tryCatch({
    dat_subset = as.data.frame(sample_dat_FC_pvalue[, grepl(j,

colnames(sample_dat_FC_pvalue))])
    dat_subset$Treatment = j

    dat_merge = merge(dat_subset, sampleGenes, by = "row.names", all.x = TRUE)

    row.names(dat_merge) = dat_merge$Row.names
    dat_merge = dat_merge[, -1]

    dat_subset = dat_merge

    dat_subset$Accession = rownames(dat_subset)

    colnames(dat_subset) = c("FC", "pvalue", "Treatment", "Gene", "Accession")

    #dat_cbind = dat_cbind[order(dat_cbind$Module), ]

    #head(dat_cbind)

    assign(x = paste("dat_dotplot", j, sep = "_"), value = dat_subset)
  }, error = function(e) {
    cat("ERROR:", conditionMessage(e), i, j, "\n")
~~~~

```
  })
}

#rbind treatment data to get long format
dat_rbind = do.call(rbind, lapply(ls(pattern = "dat_dotplot_"),
                                  function(x) {
                                    get_dat = get(x)
                                    get_dat
                                  }))

dat_rbind$Gene = as.character(dat_rbind$Gene)

#change treatment to see comparison mean
dat_to_plot$Treatment = paste(dat_to_plot$Treatment, " vs ", controlsample)

#use data set for plot
dat_to_plot = dat_rbind

#calculate min and max in data set
FC_min = abs(min(dat_to_plot$FC, na.rm = TRUE))
FC_max = abs(max(dat_to_plot$FC, na.rm = TRUE))

#define limits based on min and max
FC_limit = ceiling(max(FC_min, FC_max))

#determine p-value limits
maxp = ceiling(ifelse(-log10(min(
  dat_to_plot$pvalue, na.rm = TRUE
)) <= 5, 5,-log10(min(
  dat_to_plot$pvalue, na.rm = TRUE
))))

#calculate -Log10(p-value)
dat_to_plot$Log10pvalue = -log10(dat_to_plot$pvalue)

#define confidence intervals
conf_up = quantile(dat_to_plot$FC, set_conf, na.rm = TRUE)
conf_down = quantile(dat_to_plot$FC, 1-set_conf, na.rm = TRUE)
conf_pvalue = quantile(dat_to_plot$Log10pvalue, set_conf, na.rm = TRUE)

#add a column containing colors
dat_to_plot$Color = "black"

#add a column containing labels
dat_to_plot$Label = NA
dat_to_plot$GeneLabel = NA

#for all rows with significant entries change color and label
for (k in 1:dim(dat_to_plot)[1]) {
  tryCatch({
    if (dat_to_plot$FC[k] < 0 &
        dat_to_plot$Log10pvalue[k] > 1.3)
      dat_to_plot$Color[k] = color_down
    if (dat_to_plot$FC[k] < conf_down &
        dat_to_plot$Log10pvalue[k] > 1.3){
      dat_to_plot$Label[k] = dat_to_plot$Accession[k]
      dat_to_plot$GeneLabel[k] = dat_to_plot$Gene[k]
    }
    if (dat_to_plot$FC[k] > 0 &
        dat_to_plot$Log10pvalue[k] > 1.3)
      dat_to_plot$Color[k] = color_up
    if (dat_to_plot$FC[k] > conf_up &
        dat_to_plot$Log10pvalue[k] > 1.3){
      dat_to_plot$Label[k] = dat_to_plot$Accession[k]
      dat_to_plot$GeneLabel[k] = dat_to_plot$Gene[k]
    }
    if (dat_to_plot$Log10pvalue[k] > conf_pvalue){
      dat_to_plot$Label[k] = dat_to_plot$Accession[k]
      dat_to_plot$GeneLabel[k] = dat_to_plot$Gene[k]
    }
```

```
  }, error = function(e) {
    cat("ERROR:", conditionMessage(e), k, "\n")
  })
}

#change treatment to see comparison mean
dat_to_plot$Treatment = paste(dat_to_plot$Treatment, " vs ", controlsample)

#use data for plotting
get_dat = dat_to_plot

#generate wrapped volcano plot as metafile
filetitle = paste("Volcano_Wrap_", pvalue_decision, ".wmf", sep = "")
plotfile = paste(plots_dir, filetitle, sep = dirsep)
win.metafile(
  file = plotfile,
  width =
length(unique(get_dat$Treatment))/ceiling(length(unique(get_dat$Treatment)) / 3) *
3 + 0.5,
  height = ceiling(length(unique(get_dat$Treatment)) / 3) * 3 + 0.5,
  pointsize = 12,
  family = define_font
)

gp = ggplot(get_dat, aes(
  x = FC,
  y = Log10pvalue,
  #label = Label,
  colour = Color
)) +
  scale_color_identity() +
  geom_point(size = 1) +
  #geom_hline(yintercept = 1.3, linetype = "dashed", color = "red") +
  scale_y_continuous(
    limits = c(0, maxp),
    expand = c(0, 0),
    breaks = c(0, maxp / 2, maxp)
  ) +
  scale_x_continuous(limits = c(-FC_limit, FC_limit),
                     expand = c(0, 0)) +
  # geom_text(
  #   aes(label = Label),
  #   hjust = 0,
  #   vjust = 0.5,
  #   size = rel(2)
  # ) +
  theme_classic() +
  theme(
    axis.title.y = element_text(
      size = rel(1),
      face = "plain",
      color = "black"
    ),
    axis.title.x = element_text(
      size = rel(1),
      face = "plain",
      color = "black"
    ),
    axis.text.x = element_text(
      size = rel(1),
      color = "black",
      face = "plain",
      angle = 0,
      vjust = 1,
      hjust = 0.5
    ),
    axis.text.y = element_text(
      size = rel(1),
      color = "black",
      hjust = 1,
```

```
      face = "plain"
    ),
    axis.line = element_line(colour = "black", size = 0.5),
    axis.ticks = element_line(colour = "black", size = 0.5),
    legend.title = element_blank(),
    panel.spacing = unit(0.5, "cm"),
    #plot.margin = margin(1,1,1,1, "cm"),
    plot.title = element_text(
      size = rel(1.5),
      face = "bold",
      color = "black",
      hjust = 0.5
    )
  ) +
  #ggtitle(i) +
  ylab(paste("-Log10(", pvalue_decision, ")", sep = "")) +
  xlab("Log2(FC)") +
  facet_wrap( ~ Treatment, nrow = ceiling(length(unique(get_dat$Treatment)) / 3)) +
  theme(
    panel.background = element_rect(fill = NA, color = "black"),
    strip.text.x = element_text(
      size = rel(1.5),
      face = "bold",
      color = "black"
    ),
    strip.text.y = element_text(
      size = rel(1.5),
      face = "bold",
      color = "black"
    ),
    strip.background = element_rect(colour = "white", fill = "grey")
  )

print(gp)
dev.off()

#generate wrapped volcano plot as pdf - don't define family
filetitle = paste("Volcano_Wrap_", pvalue_decision, ".pdf", sep = "")
plotfile = paste(plots_dir, filetitle, sep = dirsep)
pdf(
  file = plotfile,
  width =
length(unique(get_dat$Treatment))/ceiling(length(unique(get_dat$Treatment)) / 3) *
3 + 0.5,
  height = ceiling(length(unique(get_dat$Treatment)) / 3) * 3 + 0.5,
  pointsize = 12
)

print(gp)
dev.off()

#plot gene names
#generate wrapped volcano plot as metafile
filetitle = paste("Volcano_Wrap_Genes_", pvalue_decision, ".wmf", sep = "")
plotfile = paste(plots_dir, filetitle, sep = dirsep)
win.metafile(
  file = plotfile,
  width =
length(unique(get_dat$Treatment))/ceiling(length(unique(get_dat$Treatment)) / 3) *
3 + 0.5,
  height = ceiling(length(unique(get_dat$Treatment)) / 3) * 3 + 0.5,
  pointsize = 12,
  family = define_font
)

gp = ggplot(get_dat, aes(
  x = FC,
  y = Log10pvalue,
  label = GeneLabel,
  colour = Color
```

```
)) +
  scale_color_identity() +
  geom_point(size = 1) +
  #geom_hline(yintercept = 1.3, linetype = "dashed", color = "red") +
  scale_y_continuous(
    limits = c(0, maxp),
    expand = c(0, 0),
    breaks = c(0, maxp / 2, maxp)
  ) +
  scale_x_continuous(limits = c(-FC_limit, FC_limit),
                     expand = c(0, 0)) +
  geom_text(
    aes(label = GeneLabel),
    hjust = "outward",
    vjust = 0.4,
    size = rel(2)
  ) +
  theme_classic() +
  theme(
    axis.title.y = element_text(
      size = rel(1),
      face = "plain",
      color = "black"
    ),
    axis.title.x = element_text(
      size = rel(1),
      face = "plain",
      color = "black"
    ),
    axis.text.x = element_text(
      size = rel(1),
      color = "black",
      face = "plain",
      angle = 0,
      vjust = 1,
      hjust = 0.5
    ),
    axis.text.y = element_text(
      size = rel(1),
      color = "black",
      hjust = 1,
      face = "plain"
    ),
    axis.line = element_line(colour = "black", size = 0.5),
    axis.ticks = element_line(colour = "black", size = 0.5),
    legend.title = element_blank(),
    panel.spacing = unit(0.5, "cm"),
    #plot.margin = margin(1,1,1,1, "cm"),
    plot.title = element_text(
      size = rel(1.5),
      face = "bold",
      color = "black",
      hjust = 0.5
    )
  ) +
  #ggtitle(i) +
  ylab(paste("-Log10(", pvalue_decision, ")", sep = "")) +
  xlab("Log2(FC)") +
  facet_wrap( ~ Treatment, nrow = ceiling(length(unique(get_dat$Treatment)) / 3)) +
  theme(
    panel.background = element_rect(fill = NA, color = "black"),
    strip.text.x = element_text(
      size = rel(1.5),
      face = "bold",
      color = "black"
    ),
    strip.text.y = element_text(
      size = rel(1.5),
      face = "bold",
      color = "black"
```

````
    ),
    strip.background = element_rect(colour = "white", fill = "grey")
  )

print(gp)
dev.off()

filetitle = paste("Volcano_Wrap_Genes_", pvalue_decision, ".pdf", sep = "")
plotfile = paste(plots_dir, filetitle, sep = dirsep)
pdf(
  file = plotfile,
  width =
length(unique(get_dat$Treatment))/ceiling(length(unique(get_dat$Treatment)) / 3) *
3 + 0.5,
  height = ceiling(length(unique(get_dat$Treatment)) / 3) * 3 + 0.5,
  pointsize = 12
)

print(gp)

dev.off()
```

#### 2.8.2 Single volcano plots

```{r single volcano plots}

for (i in unique(dat_to_plot$Treatment)){

  get_dat = dat_to_plot[dat_to_plot$Treatment == i, ]

  #generate wrapped volcano plot as metafile
filetitle = paste("Volcano_Wrap_", pvalue_decision, "_", i, ".wmf", sep = "")
plotfile = paste(plots_dir, filetitle, sep = dirsep)
win.metafile(
  file = plotfile,
  width = 3.5,
  height = 3.5,
  pointsize = 12,
  family = define_font
)

gp = ggplot(get_dat, aes(
  x = FC,
  y = Log10pvalue,
  #label = Label,
  colour = Color
)) +
  scale_color_identity() +
  geom_point(size = 1) +
  #geom_hline(yintercept = 1.3, linetype = "dashed", color = "red") +
  scale_y_continuous(
    limits = c(0, maxp),
    expand = c(0, 0),
    breaks = c(0, maxp / 2, maxp)
  ) +
  scale_x_continuous(limits = c(-FC_limit, FC_limit),
                     expand = c(0, 0)) +
  # geom_text(
  #   aes(label = Label),
  #   hjust = 0,
  #   vjust = 0.5,
  #   size = rel(2)
  # ) +
  theme_classic() +
  theme(
    axis.title.y = element_text(
      size = rel(1),
      face = "plain",
      color = "black"
````

```
    ),
    axis.title.x = element_text(
      size = rel(1),
      face = "plain",
      color = "black"
    ),
    axis.text.x = element_text(
      size = rel(1),
      color = "black",
      face = "plain",
      angle = 0,
      vjust = 1,
      hjust = 0.5
    ),
    axis.text.y = element_text(
      size = rel(1),
      color = "black",
      hjust = 1,
      face = "plain"
    ),
    axis.line = element_line(colour = "black", size = 0.5),
    axis.ticks = element_line(colour = "black", size = 0.5),
    legend.title = element_blank(),
    panel.spacing = unit(0.5, "cm"),
    #plot.margin = margin(1,1,1,1, "cm"),
    plot.title = element_text(
      size = rel(1.5),
      face = "bold",
      color = "black",
      hjust = 0.5
    )
  ) +
  #ggtitle(i) +
  ylab(paste("-Log10(", pvalue_decision, ")", sep = "")) +
  xlab("Log2(FC)") +
  facet_wrap( ~ Treatment, nrow = ceiling(length(unique(get_dat$Treatment)) / 3)) +
  theme(
    panel.background = element_rect(fill = NA, color = "black"),
    strip.text.x = element_text(
      size = rel(1.5),
      face = "bold",
      color = "black"
    ),
    strip.text.y = element_text(
      size = rel(1.5),
      face = "bold",
      color = "black"
    ),
    strip.background = element_rect(colour = "white", fill = "grey")
  )

print(gp)
dev.off()

#generate wrapped volcano plot as pdf - don't define family
filetitle = paste("Volcano_Wrap_", pvalue_decision, "_", i, ".pdf", sep = "")
plotfile = paste(plots_dir, filetitle, sep = dirsep)
pdf(
  file = plotfile,
  width = 3.5,
  height = 3.5,
  pointsize = 12
)

print(gp)
dev.off()

#plot gene names
#generate wrapped volcano plot as metafile
filetitle = paste("Volcano_Wrap_Genes_", pvalue_decision, "_", i, ".wmf", sep = "")
```

```
plotfile = paste(plots_dir, filetitle, sep = dirsep)
win.metafile(
  file = plotfile,
  width = 3.5,
  height = 3.5,
  pointsize = 12,
  family = define_font
)

gp = ggplot(get_dat, aes(
  x = FC,
  y = Log10pvalue,
  label = GeneLabel,
  colour = Color
)) +
  scale_color_identity() +
  geom_point(size = 1) +
  #geom_hline(yintercept = 1.3, linetype = "dashed", color = "red") +
  scale_y_continuous(
    limits = c(0, maxp),
    expand = c(0, 0),
    breaks = c(0, maxp / 2, maxp)
  ) +
  scale_x_continuous(limits = c(-FC_limit, FC_limit),
                     expand = c(0, 0)) +
  geom_text(
    aes(label = GeneLabel),
    hjust = "outward",
    vjust = 0.4,
    size = rel(2)
  ) +
  theme_classic() +
  theme(
    axis.title.y = element_text(
      size = rel(1),
      face = "plain",
      color = "black"
    ),
    axis.title.x = element_text(
      size = rel(1),
      face = "plain",
      color = "black"
    ),
    axis.text.x = element_text(
      size = rel(1),
      color = "black",
      face = "plain",
      angle = 0,
      vjust = 1,
      hjust = 0.5
    ),
    axis.text.y = element_text(
      size = rel(1),
      color = "black",
      hjust = 1,
      face = "plain"
    ),
    axis.line = element_line(colour = "black", size = 0.5),
    axis.ticks = element_line(colour = "black", size = 0.5),
    legend.title = element_blank(),
    panel.spacing = unit(0.5, "cm"),
    #plot.margin = margin(1,1,1,1, "cm"),
    plot.title = element_text(
      size = rel(1.5),
      face = "bold",
      color = "black",
      hjust = 0.5
    )
  ) +
  #ggtitle(i) +
```

````
  ylab(paste("-Log10(", pvalue_decision, ")", sep = "")) +
  xlab("Log2(FC)") +
  facet_wrap( ~ Treatment, nrow = ceiling(length(unique(get_dat$Treatment)) / 3)) +
  theme(
    panel.background = element_rect(fill = NA, color = "black"),
    strip.text.x = element_text(
      size = rel(1.5),
      face = "bold",
      color = "black"
    ),
    strip.text.y = element_text(
      size = rel(1.5),
      face = "bold",
      color = "black"
    ),
    strip.background = element_rect(colour = "white", fill = "grey")
  )

print(gp)
dev.off()

filetitle = paste("Volcano_Wrap_Genes_", pvalue_decision, "_", i, ".pdf", sep = "")
plotfile = paste(plots_dir, filetitle, sep = dirsep)
pdf(
  file = plotfile,
  width = 3.5,
  height = 3.5,
  pointsize = 12
)

print(gp)

dev.off()
}
```


#### 2.8.3 Volcano Facets zscores

```{r volcano facets zscores}
#choose data set
sample_dat_FC_pvalue = summary_zscore

#exclude control because it contains only 1 and 0 values
sample_dat_FC_pvalue = sample_dat_FC_pvalue[,
which(grepl(colnames(sample_dat_FC_pvalue), pattern = controlsample) == FALSE)]

#create sample vector
sample_vector = unique(gsub(
  colnames(sample_dat_FC_pvalue),
  pattern = "_pvalue",
  replacement = ""
))

#bring data to long format, which is necessary for ggplot
for (j in sample_vector) {
  tryCatch({
    dat_subset = as.data.frame(sample_dat_FC_pvalue[, grepl(j,

colnames(sample_dat_FC_pvalue))])
    dat_subset$Treatment = j

    dat_subset$Accession = rownames(dat_subset)

    colnames(dat_subset) = c("FC", "pvalue", "Treatment", "Accession")

    #dat_cbind = dat_cbind[order(dat_cbind$Module), ]

    #head(dat_cbind)
````

```
    dat_subset = merge(dat_subset, sampleGenes, by = "row.names")

    rownames(dat_subset) = dat_subset$Row.names

    dat_subset = dat_subset[, -1]

    assign(x = paste("dat_dotplot", j, sep = "_"), value = dat_subset)
  }, error = function(e) {
    cat("ERROR:", conditionMessage(e), i, j, "\n")
  })
}

#rbind treatment data to get long format
dat_rbind = do.call(rbind, lapply(ls(pattern = "dat_dotplot_"),
                                  function(x) {
                                    get_dat = get(x)
                                    get_dat
                                  }))

#use data set for plot
dat_to_plot = dat_rbind

#gene data as character
dat_to_plot$gene_names = as.character(dat_to_plot$gene_names)

#add to the treatment the control sample
dat_to_plot$Treatment = paste(dat_to_plot$Treatment, " vs ", controlsample)

#calculate min and max in data set
FC_min = abs(min(dat_to_plot$FC, na.rm = TRUE))
FC_max = abs(max(dat_to_plot$FC, na.rm = TRUE))

#define limits based on min and max
FC_limit = ceiling(max(FC_min, FC_max))

#determine p-value limits
maxp = ceiling(ifelse(-log10(min(
  dat_to_plot$pvalue, na.rm = TRUE
)) <= 5, 5,-log10(min(
  dat_to_plot$pvalue, na.rm = TRUE
))))

#calculate -Log10(p-value)
dat_to_plot$Log10pvalue = -log10(dat_to_plot$pvalue)

#define confidence intervals
conf_up = quantile(dat_to_plot$FC, set_conf, na.rm = TRUE)
conf_down = quantile(dat_to_plot$FC, 1-set_conf, na.rm = TRUE)
conf_pvalue = quantile(dat_to_plot$Log10pvalue, set_conf, na.rm = TRUE)

#add a column containing colors
dat_to_plot$Color = "black"

#add a column containing labels
dat_to_plot$Label = NA

#for all rows with significant entries change color and label
for (k in 1:dim(dat_to_plot)[1]) {
  tryCatch({
    if (dat_to_plot$FC[k] < 0 &
        dat_to_plot$Log10pvalue[k] > 1.3) #1.9 is 95 % interval
      dat_to_plot$Color[k] = color_down
    if (dat_to_plot$FC[k] <= conf_down &
        dat_to_plot$Log10pvalue[k] > 1.3) #2.57 is 99 % interval
      dat_to_plot$Label[k] = dat_to_plot$Gene[k]
    if (dat_to_plot$FC[k] > 0 &
        dat_to_plot$Log10pvalue[k] > 1.3)
      dat_to_plot$Color[k] = color_up
    if (dat_to_plot$FC[k] >= conf_up &
        dat_to_plot$Log10pvalue[k] > 1.3)
```

```
      dat_to_plot$Label[k] = dat_to_plot$Gene[k]
    if (dat_to_plot$Log10pvalue[k] > conf_pvalue)
      dat_to_plot$Label[k] = dat_to_plot$Gene[k]
  }, error = function(e) {
    cat("ERROR:", conditionMessage(e), k, "\n")
  })
}

#use data for plotting
get_dat = dat_to_plot

#generate wrapped volcano plot as metafile
filetitle = paste("Volcano_Wrap_zscore_", pvalue_decision, ".wmf", sep = "")
plotfile = paste(plots_dir, filetitle, sep = dirsep)
win.metafile(
  file = plotfile,
  width =
length(unique(get_dat$Treatment))/ceiling(length(unique(get_dat$Treatment)) / 3) *
3 + 0.5,
  height = ceiling(length(unique(get_dat$Treatment)) / 3) * 3 + 0.5,
  pointsize = 12,
  family = define_font
)

gp = ggplot(get_dat, aes(
  x = FC,
  y = Log10pvalue,
  label = Label,
  colour = Color
)) +
  scale_color_identity() +
  geom_point(size = 1) +
  #geom_hline(yintercept = 1.3, linetype = "dashed", color = "red") +
  scale_y_continuous(
    limits = c(0, maxp),
    expand = c(0, 0),
    breaks = c(0, maxp / 2, maxp)
  ) +
  scale_x_continuous(limits = c(-FC_limit, FC_limit),
                     expand = c(0, 0)) +
  geom_text(
    aes(label = Label),
    hjust = "outward",
    vjust = 0.4,
    size = rel(2)
  ) +
  theme_classic() +
  theme(
    axis.title.y = element_text(
      size = rel(1),
      face = "plain",
      color = "black"
    ),
    axis.title.x = element_text(
      size = rel(1),
      face = "plain",
      color = "black"
    ),
    axis.text.x = element_text(
      size = rel(1),
      color = "black",
      face = "plain",
      angle = 0,
      vjust = 1,
      hjust = 0.5
    ),
    axis.text.y = element_text(
      size = rel(1),
      color = "black",
      hjust = 1,
```

```
      face = "plain"
    ),
    axis.line = element_line(colour = "black", size = 0.5),
    axis.ticks = element_line(colour = "black", size = 0.5),
    legend.title = element_blank(),
    panel.spacing = unit(0.5, "cm"),
    #plot.margin = margin(1,1,1,1, "cm"),
    plot.title = element_text(
      size = rel(1.5),
      face = "bold",
      color = "black",
      hjust = 0.5
    )
  ) +
  #ggtitle(i) +
  ylab(paste("-Log10(", pvalue_decision, ")", sep = "")) +
  xlab("z-score") +
  facet_wrap( ~ Treatment, nrow = ceiling(length(unique(get_dat$Treatment)) / 3)) +
  theme(
    panel.background = element_rect(fill = NA, color = "black"),
    strip.text.x = element_text(
      size = rel(1.5),
      face = "bold",
      color = "black"
    ),
    strip.text.y = element_text(
      size = rel(1.5),
      face = "bold",
      color = "black"
    ),
    strip.background = element_rect(colour = "white", fill = "grey")
  )

print(gp)
dev.off()

#generate wrapped volcano plot as pdf - don't define family
filetitle = paste("Volcano_Wrap_zscore_", pvalue_decision, ".pdf", sep = "")
plotfile = paste(plots_dir, filetitle, sep = dirsep)
pdf(
  file = plotfile,
  width =
length(unique(get_dat$Treatment))/ceiling(length(unique(get_dat$Treatment)) / 3) *
3 + 0.5,
  height = ceiling(length(unique(get_dat$Treatment)) / 3) * 3 + 0.5,
  pointsize = 12
)

print(gp)
dev.off()

filetitle = paste("Volcano_Wrap_zscore_", pvalue_decision, ".svg", sep = "")
plotfile = paste(plots_dir, filetitle, sep = dirsep)
svg(
  file = plotfile,
  width =
length(unique(get_dat$Treatment))/ceiling(length(unique(get_dat$Treatment)) / 3) *
3 + 0.5,
  height = ceiling(length(unique(get_dat$Treatment)) / 3) * 3 + 0.5,
  pointsize = 12,
  family = define_font
)

print(gp)
dev.off()

filetitle = paste("Volcano_Wrap_zscore_", pvalue_decision, ".png", sep = "")
plotfile = paste(plots_dir, filetitle, sep = dirsep)
png(
  file = plotfile,
```

````
  width =
length(unique(get_dat$Treatment))/ceiling(length(unique(get_dat$Treatment)) / 3) *
3 + 0.5,
  height = ceiling(length(unique(get_dat$Treatment)) / 3) * 3 + 0.5,
  pointsize = 12,
  units = "in",
  res = 800,
  family = define_font
)

print(gp)
dev.off()
```


### 2.9 Export table of nCounts

```{r summary table proteins}
#create lists of data
summary_table_list = ls(pattern = "nProteins_")
summary_table_list

remove(Calculated_list)
Calculated_list = ls(pattern = "Calculated_")
Calculated_list

#create empty vectors that will be replaced in the next steps
empty_vector_colnames = gsub(summary_table_list, pattern = "nProteins_",
replacement = "")
empty_vector_nreps = rep(NA, length(summary_table_list))
empty_vector_full = rep(NA, length(summary_table_list))
empty_vector_subset = rep(NA, length(summary_table_list))
empty_vector_p005 = rep(NA, length(summary_table_list))
empty_vector_p001 = rep(NA, length(summary_table_list))

#calculate how many proteins were significantly altered
for (i in 1:length(summary_table_list)) {
  get_dat = get(summary_table_list[i])
  empty_vector_nreps[i] = max(get_dat$nCounts)

  get_dat = get_dat[which(get_dat$nCounts > 0),]
  empty_vector_full[i] = dim(get_dat)[1]
  get_dat = get_dat[which(get_dat$nCounts >= reliablereps),]
  empty_vector_subset[i] = dim(get_dat)[1]

  get_dat = get(Calculated_list[i])
  get_dat = get_dat[, grepl(colnames(get_dat), pattern = "_pvalue.adj$")]
  get_dat = na.exclude(get_dat)
  get_dat = get_dat[get_dat <= 0.05]
  empty_vector_p005[i] = length(get_dat)

  get_dat = get_dat[get_dat <= 0.01]
  empty_vector_p001[i] = length(get_dat)
}

#summarize results
summary_table = as.data.frame(do.call(cbind, lapply(ls(pattern = "empty_vector_"),
function (x) {
  get_dat = get(x)
  get_dat
})))

colnames_summary_table = c(gsub(as.vector(ls(pattern = "empty_vector_")),
                                pattern = "empty_vector_",
                                replacement = ""))

colnames(summary_table) = colnames_summary_table

summary_table_final = as.data.frame(
  cbind(
````

````
    as.character(summary_table$colnames),
    as.numeric(as.character(summary_table$nreps)),
    as.numeric(as.character(summary_table$full)),
    as.numeric(as.character(summary_table$subset)),
    as.numeric(as.character(summary_table$p005)),
    as.numeric(as.character(summary_table$p001))
  )
)

colnames(summary_table_final) = c(
  "Treatment",
  "nReps",
  "nProteins",
  paste("nProteins (", reliablereps, "/", nReps, ")", sep = ""),
  "p.adj<0.05",
  "p.adj<0.01"
)

filename = paste(Rdata_dir, "summary_table_nCounts_proteins.csv", sep = "/")
write.csv(file = filename, x = summary_table_final)
```

### 2.10 Heatmaps
#### 2.10.1 Heatmap with FCs
##### 2.10.1.1 All reliably identified proteins

```{r heatmap FCs}
#define clustering method
distance_measure = "euclidean" #default: "euclidean"
clustering_method = "complete" #default: "complete"

for (i in ls(pattern = "dat_means_short_")) {
  dat_to_scale = get(i)

  #remove all rows that contain only NA
  dat_to_scale[dat_to_scale == "NaN"] = NA
  dat_to_scale = dat_to_scale[rowSums(is.na(dat_to_scale)) != ncol(dat_to_scale),]

  #remove control sample because it contains only values of 1
  dat_to_scale = dat_to_scale[, which(grepl(colnames(dat_to_scale),
                                            pattern = controlsample) == FALSE)]

  boxplot(dat_to_scale, las = 2)

  dat_to_scale = dat_to_scale[rowSums(is.na(dat_to_scale)) < ncol(dat_to_scale), ]

  merge_dat = dat_to_scale

  #replace NA by 0 to allow for row clustering
  dat_to_scale[is.na(dat_to_scale)] = 0

  boxplot(dat_to_scale, las = 2)

  min = min(dat_to_scale, na.rm = TRUE)
  max = max(dat_to_scale, na.rm = TRUE)

  limits = ceiling(max(abs(min), abs(max)))/2

  # create empty vector that will be filled with median values
  median_abundance_vector = vector(mode = "expression", length =
length(colnames(dat_to_scale)))
  for (j in 1:length(colnames(merge_dat))){
    M = na.omit(merge_dat[,j])
    median_abundance_vector[j] = median(M)
  }
  head(median_abundance_vector)

  mid = round(median(unlist(lapply(median_abundance_vector, as.numeric))), digits =
0)
  min = -limits
````

```
  max = limits

  dend_rows = dendsort(hclust(dist(dat_to_scale))) #can not be used with NAs
  dend_cols = dendsort(hclust(dist(t(dat_to_scale))))
  dend_cols = color_branches(dend_cols, k = floor(dim(dat_to_scale)[2]/2))

  plot_file = paste("Heatmap_", i, ".pdf", sep = "")
  plot_path = paste(plots_dir, plot_file, sep = dirsep)
  pdf(file = plot_path,
      width = dim(merge_dat)[2],
      height = 8)

  h = Heatmap(
    merge_dat,
    col = colorRamp2(c(min, mid, max), c(color_down, "white", color_up)),
    color_space = "sRGB",
    na_col = "grey",
    cluster_rows = dend_rows,
    cluster_columns = dend_cols,
    clustering_distance_columns = distance_measure,
    clustering_distance_rows = distance_measure,
    clustering_method_columns = clustering_method,
    clustering_method_rows = clustering_method,
    column_dend_side = "top",
    column_dend_height = unit(3, "cm"),
    column_names_side = "top",
    show_row_names = "FALSE",
    #row_names_side = "left",
    #row_names_max_width = unit(15, "cm"),
    column_names_max_height = unit(6, "cm"),
    show_row_dend = TRUE,
    #row_title_gp = gpar(cex = 0.6),
    column_names_gp = gpar(cex = 1, fontface = "bold"),
    #column_dend_reorder=c(1,3,4,5,2),
    #row_dend_reorder=F,
    heatmap_legend_param = list(
      title = "Log2(FC)",
      title_gp = gpar(cex = 1, fontface = "bold"),
      color_bar = c("continuous")
    ),
    show_heatmap_legend = TRUE
  )

  print(h)

  dev.off()

  #plot with discrete breaks
discrete_sequence = seq(from = min, to = max, by = 0.5)
discrete_colors = colorRampPalette(colors = c(color_down, "white",
                                              color_up))(length(discrete_sequence))

  plot_file = paste("Heatmap_discrete_", i, ".pdf", sep = "")
  plot_path = paste(plots_dir, plot_file, sep = dirsep)
  pdf(file = plot_path,
      width = dim(merge_dat)[2],
      height = 8)

  h = Heatmap(
  merge_dat,
  col = colorRamp2(breaks = discrete_sequence, colors = discrete_colors),
  color_space = "sRGB",
  na_col = "grey",
  cluster_rows = dend_rows,
  cluster_columns = dend_cols,
  clustering_distance_columns = distance_measure,
  clustering_distance_rows = distance_measure,
  clustering_method_columns = clustering_method,
  clustering_method_rows = clustering_method,
  column_dend_side = "top",
```

````
  column_dend_height = unit(3, "cm"),
  column_names_side = "top",
  show_row_names = "FALSE",
  #row_names_side = "left",
  #row_names_max_width = unit(15, "cm"),
  column_names_max_height = unit(6, "cm"),
  show_row_dend = TRUE,
  row_dend_width = unit(2, "cm"),
  #row_title_gp = gpar(cex = 0.6),
  column_names_gp = gpar(cex = 1, fontface = "bold"),
  #column_dend_reorder=c(3,2,1,7,4,5,6),
  #row_dend_reorder=F,
  heatmap_legend_param = list(
    title = "Log2(FC)",
    at = seq(from = min, to = max, by = max/2),
    title_gp = gpar(cex = 1, fontface = "bold"),
    color_bar = c("discrete")
  ),
  show_heatmap_legend = TRUE
)

print(h)

dev.off()

plot_file = paste("Heatmap_discrete_", i, ".wmf", sep = "")
  plot_path = paste(plots_dir, plot_file, sep = dirsep)
  win.metafile(file = plot_path,
      width = dim(merge_dat)[2],
      family = define_font,
      height = 8)

  print(h)

dev.off()

plot_file = paste("Heatmap_discrete_", i, ".svg", sep = "")
  plot_path = paste(plots_dir, plot_file, sep = dirsep)
  svg(file = plot_path,
      width = dim(merge_dat)[2],
      family = define_font,
      height = 8)

  print(h)

dev.off()

plot_file = paste("Heatmap_discrete_", i, ".png", sep = "")
  plot_path = paste(plots_dir, plot_file, sep = dirsep)
  png(file = plot_path,
      width = dim(merge_dat)[2],
      family = define_font,
      units = "in",
      res = 800,
      height = 8)

  print(h)

dev.off()
}
```

#### 2.10.2 Heatmap with Abundances

```{r heatmap abundances}
#plot abundances of means
#extract abundance means
heatmap_abundances_df = as.data.frame(do.call(cbind, lapply(ls(pattern =
"Calculated_Replicates_reliable_"),
                                                        function(x) {
````

```
                                                              get_dat = get(x)
                                                              get_dat = get_dat[,

grepl(colnames(get_dat),

pattern = "_Mean$")]
                                                              as.vector(get_dat)
                                                            })))

heatmap_abundances_colnames = as.vector(ls(pattern =
"Calculated_Replicates_reliable_"))
heatmap_abundances_colnames = gsub(heatmap_abundances_colnames,
                                   pattern = "Calculated_Replicates_reliable_",
                                   replacement = "")
colnames(heatmap_abundances_df) = heatmap_abundances_colnames

heatmap_abundances_df[heatmap_abundances_df == "NaN"] = NA

#define limits
max_heatmap = max(heatmap_abundances_df, na.rm = TRUE)*0.9
max = ceiling(max_heatmap)

# create empty vector that will be filled with median values
median_abundance_vector = vector(mode = "expression",
                                 length = length(colnames(heatmap_abundances_df)))

for (j in 1:length(colnames(heatmap_abundances_df))){
  M = na.omit(heatmap_abundances_df[,j])
  median_abundance_vector[j] = median(M)
}
head(median_abundance_vector)

mid = round(median(unlist(lapply(median_abundance_vector, as.numeric))), digits =
0)

min = mid - (max-mid)

heatmap_abundances_df = heatmap_abundances_df[rowSums(is.na(heatmap_abundances_df))
<
                                                ncol(heatmap_abundances_df), ]

dat_to_plot = heatmap_abundances_df

#replace NAs by 0
heatmap_abundances_df[is.na(heatmap_abundances_df)] = 0

dend_rows = dendsort(hclust(dist(heatmap_abundances_df))) #can't be used with NAs
dend_cols = dendsort(hclust(dist(t(heatmap_abundances_df))))
dend_cols = color_branches(dend_cols, k = floor(dim(heatmap_abundances_df)[2] / 2))

plot_file = "Heatmap_AbundanceMeans.pdf"
plot_path = paste(plots_dir, plot_file, sep = dirsep)
pdf(file = plot_path,
    width = dim(dat_to_plot)[2] + 2,
    height = 8)

h = Heatmap(
  dat_to_plot,
  col = colorRamp2(c(min, mid, max), c(color_low, color_mid, color_high)),
  color_space = "sRGB",
  na_col = "grey",
  cluster_rows = dend_rows,
  cluster_columns = dend_cols,
  clustering_distance_columns = distance_measure,
  clustering_distance_rows = distance_measure,
  clustering_method_columns = clustering_method,
  clustering_method_rows = clustering_method,
  column_dend_side = "top",
  column_dend_height = unit(3, "cm"),
  column_names_side = "top",
```

```
  show_row_names = "FALSE",
  #row_names_side = "left",
  #row_names_max_width = unit(15, "cm"),
  column_names_max_height = unit(6, "cm"),
  show_row_dend = TRUE,
  #row_title_gp = gpar(cex = 0.6),
  column_names_gp = gpar(cex = 1, fontface = "bold"),
  #column_dend_reorder=c(1,3,4,5,2),
  #row_dend_reorder=F,
  heatmap_legend_param = list(
    title = "Log2(Abundance)",
    title_gp = gpar(cex = 1, fontface = "bold"),
    at = c(min, mid, max),
    labels = c(min, mid, max),
    color_bar = c("continuous")
  ),
  show_heatmap_legend = TRUE
)

print(h)

dev.off()

#plot with discrete breaks
discrete_sequence = seq(from = min, to = max, by = 1)
discrete_colors = colorRampPalette(colors = c(color_low, color_mid,

color_high))(length(discrete_sequence))

plot_file = "Heatmap_AbundanceMeans_discrete.pdf"
plot_path = paste(plots_dir, plot_file, sep = dirsep)
pdf(file = plot_path,
    width = dim(dat_to_plot)[2] + 2,
    height = 8)

h = Heatmap(
  dat_to_plot,
  col = colorRamp2(breaks = discrete_sequence, colors = discrete_colors),
  color_space = "sRGB",
  na_col = "grey",
  cluster_rows = dend_rows,
  cluster_columns = dend_cols,
  clustering_distance_columns = distance_measure,
  clustering_distance_rows = distance_measure,
  clustering_method_columns = clustering_method,
  clustering_method_rows = clustering_method,
  column_dend_side = "top",
  column_dend_height = unit(3, "cm"),
  column_names_side = "top",
  show_row_names = "FALSE",
  #row_names_side = "left",
  #row_names_max_width = unit(15, "cm"),
  column_names_max_height = unit(6, "cm"),
  show_row_dend = TRUE,
  row_dend_width = unit(2, "cm"),
  #row_title_gp = gpar(cex = 0.6),
  column_names_gp = gpar(cex = 1, fontface = "bold"),
  #column_dend_reorder=c(3,2,1,7,4,5,6),
  #row_dend_reorder=F,
  heatmap_legend_param = list(
    title = "Log2(Abundance)",
    at = seq(from = min, to = max, by = 2),
    title_gp = gpar(cex = 1, fontface = "bold"),
    color_bar = c("discrete")
  ),
  show_heatmap_legend = TRUE
)

print(h)
```

```
dev.off()

plot_file = "Heatmap_AbundanceMeans_discrete.wmf"
plot_path = paste(plots_dir, plot_file, sep = dirsep)
win.metafile(file = plot_path,
    width = dim(dat_to_plot)[2] + 2,
    family = define_font,
    height = 8)

print(h)

dev.off()

plot_file = "Heatmap_AbundanceMeans_discrete.svg"
plot_path = paste(plots_dir, plot_file, sep = dirsep)
svg(file = plot_path,
    width = dim(dat_to_plot)[2] + 2,
    family = define_font,
    height = 8)

print(h)

dev.off()

plot_file = "Heatmap_AbundanceMeans_discrete.png"
plot_path = paste(plots_dir, plot_file, sep = dirsep)
png(file = plot_path,
    width = dim(dat_to_plot)[2] + 2,
    units = "in",
    res = 800,
    family = define_font,
    height = 8)

print(h)

dev.off()

#plot abundances of replicates
#use log2-transformed data

dat_log2 = dat_stabilized
dat_log2 = dat_log2[, order(names(dat_log2))]

#define limits
max_heatmap = max(dat_log2, na.rm = TRUE)*0.9
max = ceiling(max_heatmap)

# create empty vector that will be filled with median values
median_abundance_vector = vector(mode = "expression",
                                 length = length(colnames(dat_log2)))

for (j in 1:length(colnames(dat_log2))){
  M = na.omit(dat_log2[,j])
  median_abundance_vector[j] = median(M)
}
head(median_abundance_vector)

mid = round(median(unlist(lapply(median_abundance_vector, as.numeric))), digits =
0)

min = mid - (max-mid)

dat_to_plot = dat_log2

#replace NA by 0 for plotting
dat_to_plot[is.na(dat_to_plot)] = 0

dend_rows = dendsort(hclust(dist(dat_to_plot)))
dend_cols = dendsort(hclust(dist(t(dat_to_plot))))
dend_cols = color_branches(dend_cols, k = floor(dim(dat_to_plot)[2] / 2))
```

```
plot_file = "Heatmap_AbundanceReplicates.pdf"
plot_path = paste(plots_dir, plot_file, sep = dirsep)
pdf(file = plot_path,
    width = dim(dat_log2)[2]/2 + 2,
    height = 8)

h = Heatmap(
  dat_log2,
  col = colorRamp2(c(min, mid, max), c(color_low, color_mid, color_high)),
  color_space = "sRGB",
  na_col = "grey",
  cluster_rows = dend_rows,
  cluster_columns = dend_cols,
  clustering_distance_columns = distance_measure,
  clustering_distance_rows = distance_measure,
  clustering_method_columns = clustering_method,
  clustering_method_rows = clustering_method,
  column_dend_side = "top",
  column_dend_height = unit(3, "cm"),
  column_names_side = "top",
  show_row_names = "FALSE",
  #row_names_side = "left",
  #row_names_max_width = unit(15, "cm"),
  column_names_max_height = unit(6, "cm"),
  show_row_dend = FALSE,
  #row_title_gp = gpar(cex = 0.6),
  column_names_gp = gpar(cex = 1, fontface = "bold"),
  #column_dend_reorder=c(1,3,4,5,2),
  #row_dend_reorder=F,
  heatmap_legend_param = list(
    title = "Log2(Abundance)",
    title_gp = gpar(cex = 1, fontface = "bold"),
    at = c(min, mid, max),
    labels = c(min, mid, max),
    color_bar = c("continuous")
  ),
  show_heatmap_legend = TRUE
)

print(h)

dev.off()

#plot with discrete colors
discrete_sequence = seq(from = min, to = max, by = 1)
discrete_colors = colorRampPalette(colors = c(color_low, color_mid,

color_high))(length(discrete_sequence))

plot_file = "Heatmap_AbundanceReplicates_discrete.pdf"
plot_path = paste(plots_dir, plot_file, sep = dirsep)
pdf(file = plot_path,
    width = dim(dat_log2)[2]/2 + 2,
    height = 8)

h = Heatmap(
  dat_log2,
  col = colorRamp2(breaks = discrete_sequence, colors = discrete_colors),
  color_space = "sRGB",
  na_col = "grey",
  cluster_rows = dend_rows,
  cluster_columns = dend_cols,
  clustering_distance_columns = distance_measure,
  clustering_distance_rows = distance_measure,
  clustering_method_columns = clustering_method,
  clustering_method_rows = clustering_method,
  column_dend_side = "top",
  column_dend_height = unit(3, "cm"),
  column_names_side = "top",
```

```
  show_row_names = "FALSE",
  #row_names_side = "left",
  #row_names_max_width = unit(15, "cm"),
  column_names_max_height = unit(6, "cm"),
  show_row_dend = TRUE,
  row_dend_width = unit(2, "cm"),
  #row_title_gp = gpar(cex = 0.6),
  column_names_gp = gpar(cex = 1, fontface = "bold"),
  #column_dend_reorder=c(1, 2, 3, 4),
  #row_dend_reorder=F,
  heatmap_legend_param = list(
    title = "Log2(Abundance)",
    at = seq(from = min, to = max, by = 2),
    title_gp = gpar(cex = 1, fontface = "bold"),
    color_bar = c("discrete")
  ),
  show_heatmap_legend = TRUE
)

print(h)

dev.off()

#plot with pheatmap and row-wise z-scoring
filename = "Pheatmap_AbundanceReplicates_discrete.pdf"
plotfile = paste(plots_dir, filename, sep = dirsep)

pdf(file = plotfile,
    width = 5,
    height = 5)
pheatmap(dat_log2,
         color = discrete_colors,
         cluster_rows = dend_rows,
         show_rownames = FALSE,
         treeheight_row = 0,
         cluster_cols = FALSE,
         show_colnames = TRUE,
         treeheight_col = 0,
         scale = "row")
dev.off()

filename = "Pheatmap_AbundanceReplicates_discrete.wmf"
plotfile = paste(plots_dir, filename, sep = dirsep)

win.metafile(file = plotfile,
    width = 5,
    height = 5,
    family = define_font)
pheatmap(dat_log2,
         color = discrete_colors,
         cluster_rows = dend_rows,
         show_rownames = FALSE,
         treeheight_row = 0,
         cluster_cols = FALSE,
         show_colnames = TRUE,
         treeheight_col = 0,
         scale = "row")
dev.off()
```

2.11. Heatmap with z-scores
2.11.1 All reliably identified proteins

```{r heatmap z-scores}

  dat_to_scale = summary_zscore

  #remove control sample because it contains only values of 1
  dat_to_scale = dat_to_scale[, which(grepl(colnames(dat_to_scale),
                                           pattern = controlsample) == FALSE)]
```

```
  dat_to_scale = dat_to_scale[, which(grepl(colnames(dat_to_scale),
                                          pattern = "_pvalue") == FALSE)]

  dat_to_scale = as.data.frame(dat_to_scale)
  dat_to_scale = dat_to_scale[!is.na(dat_to_scale)]
  dat_to_scale = as.data.frame(dat_to_scale)
  dat_to_scale = dat_to_scale[rowSums(is.na(dat_to_scale)) < ncol(dat_to_scale), ]
  dat_to_scale = as.data.frame(dat_to_scale)
  merge_dat = dat_to_scale

  #replace NA by 0 to allow for row clustering
  dat_to_scale[is.na(dat_to_scale)] = 0

  boxplot(dat_to_scale, las = 2)

  min = min(dat_to_scale, na.rm = TRUE)
  max = max(dat_to_scale, na.rm = TRUE)

  limits = floor(max(abs(min), abs(max)))/2

  # create empty vector that will be filled with median values
  median_abundance_vector = vector(mode = "expression", length =
length(colnames(dat_to_scale)))
  for (j in 1:length(colnames(merge_dat))){
    M = na.omit(merge_dat[,j])
    median_abundance_vector[j] = median(M)
  }
  head(median_abundance_vector)

  mid = round(median(unlist(lapply(median_abundance_vector, as.numeric))), digits =
0)
  min = -limits
  max = limits

  dend_rows = dendsort(hclust(dist(dat_to_scale))) #can not be used with NAs
  dend_cols = dendsort(hclust(dist(t(dat_to_scale))))
  dend_cols = color_branches(dend_cols, k = floor(dim(dat_to_scale)[2]/2))

  plot_file = "Heatmap_zscores.pdf"
  plot_path = paste(plots_dir, plot_file, sep = dirsep)
  pdf(file = plot_path,
      width = dim(merge_dat)[2],
      height = 8)

  h = Heatmap(
    merge_dat,
    col = colorRamp2(c(min, mid, max), c(color_down, "white", color_up)),
    color_space = "sRGB",
    na_col = "grey",
    cluster_rows = dend_rows,
    cluster_columns = dend_cols,
    clustering_distance_columns = distance_measure,
    clustering_distance_rows = distance_measure,
    clustering_method_columns = clustering_method,
    clustering_method_rows = clustering_method,
    column_dend_side = "top",
    column_dend_height = unit(3, "cm"),
    column_names_side = "top",
    show_row_names = "FALSE",
    #row_names_side = "left",
    #row_names_max_width = unit(15, "cm"),
    column_names_max_height = unit(6, "cm"),
    show_row_dend = TRUE,
    #row_title_gp = gpar(cex = 0.6),
    column_names_gp = gpar(cex = 1, fontface = "bold"),
    #column_dend_reorder=c(1,3,4,5,2),
    #row_dend_reorder=F,
    heatmap_legend_param = list(
      title = "z-score",
```

```
      title_gp = gpar(cex = 1, fontface = "bold"),
      color_bar = c("continuous")
    ),
    show_heatmap_legend = TRUE
  )

  print(h)

  dev.off()

  #plot with discrete breaks
discrete_sequence = seq(from = min, to = max, by = 0.5)
discrete_colors = colorRampPalette(colors = c(color_down,
                                              "white",
color_up))(length(discrete_sequence))

  plot_file = "Heatmap_zscores_discrete.pdf"
  plot_path = paste(plots_dir, plot_file, sep = dirsep)
  pdf(file = plot_path,
      width = dim(merge_dat)[2],
      height = 8)

  h = Heatmap(
  merge_dat,
  col = colorRamp2(breaks = discrete_sequence, colors = discrete_colors),
  color_space = "sRGB",
  na_col = "grey",
  cluster_rows = dend_rows,
  cluster_columns = dend_cols,
  clustering_distance_columns = distance_measure,
  clustering_distance_rows = distance_measure,
  clustering_method_columns = clustering_method,
  clustering_method_rows = clustering_method,
  column_dend_side = "top",
  column_dend_height = unit(3, "cm"),
  column_names_side = "top",
  show_row_names = "FALSE",
  #row_names_side = "left",
  #row_names_max_width = unit(15, "cm"),
  column_names_max_height = unit(6, "cm"),
  show_row_dend = TRUE,
  row_dend_width = unit(2, "cm"),
  #row_title_gp = gpar(cex = 0.6),
  column_names_gp = gpar(cex = 1, fontface = "bold"),
  #column_dend_reorder=c(3,2,1,7,4,5,6),
  #row_dend_reorder=F,
  heatmap_legend_param = list(
    title = "z-score",
    at = seq(from = min, to = max, by = max/2),
    title_gp = gpar(cex = 1, fontface = "bold"),
    color_bar = c("discrete")
  ),
  show_heatmap_legend = TRUE
)

print(h)

dev.off()

plot_file = "Heatmap_zscores_discrete.wmf"
  plot_path = paste(plots_dir, plot_file, sep = dirsep)
  win.metafile(file = plot_path,
      width = dim(merge_dat)[2],
      family = define_font,
      height = 8)

  print(h)

dev.off()
```

````
plot_file = "Heatmap_zscores_discrete.svg"
  plot_path = paste(plots_dir, plot_file, sep = dirsep)
  svg(file = plot_path,
      width = dim(merge_dat)[2],
      family = define_font,
      height = 8)

  print(h)

dev.off()

plot_file = "Heatmap_zscores_discrete.png"
  plot_path = paste(plots_dir, plot_file, sep = dirsep)
  png(file = plot_path,
      width = dim(merge_dat)[2],
      family = define_font,
      units = "in",
      res = 800,
      height = 8)

  print(h)

dev.off()
```

##### 2.11.2 Reliably identified proteins with significant changes for at least one
treatment

```{r heatmap DE z-scores}

  dat_to_scale = summary_zscore

  #remove control sample because it contains only values of 1
  dat_to_scale = dat_to_scale[, which(grepl(colnames(dat_to_scale),
                                            pattern = controlsample) == FALSE)]

  dat_pvalues = dat_to_scale[, which(grepl(colnames(dat_to_scale),
                                            pattern = "_pvalue") == TRUE)]

  dat_to_scale = dat_to_scale[, which(grepl(colnames(dat_to_scale),
                                            pattern = "_pvalue") == FALSE)]

  dat_pvalues[dat_pvalues > 0.05] = NA
  dat_to_scale = dat_to_scale[!apply(dat_pvalues, 1, function(x) all(is.na(x))), ]

  dat_to_scale = dat_to_scale[!apply(dat_to_scale, 1, function(x) all(is.na(x))), ]

  merge_dat = dat_to_scale

  #replace NA by 0 to allow for row clustering
  dat_to_scale[is.na(dat_to_scale)] = 0

  boxplot(dat_to_scale, las = 2)

  min = min(dat_to_scale, na.rm = TRUE)
  max = max(dat_to_scale, na.rm = TRUE)

  limits = floor(max(abs(min), abs(max)))/2

  # create empty vector that will be filled with median values
  median_abundance_vector = vector(mode = "expression", length =
length(colnames(dat_to_scale)))
  for (j in 1:length(colnames(merge_dat))){
    M = na.omit(merge_dat[,j])
    median_abundance_vector[j] = median(M)
  }
  head(median_abundance_vector)

  mid = round(median(unlist(lapply(median_abundance_vector, as.numeric))), digits =
0)
````

```
  min = -limits
  max = limits

  dend_rows = dendsort(hclust(dist(dat_to_scale))) #can not be used with NAs
  dend_cols = dendsort(hclust(dist(t(dat_to_scale))))
  dend_cols = color_branches(dend_cols, k = floor(dim(dat_to_scale)[2]/2))

  plot_file = "Heatmap_zscores_DEproteins.pdf"
  plot_path = paste(plots_dir, plot_file, sep = dirsep)
  pdf(file = plot_path,
      width = dim(merge_dat)[2],
      height = 8)

  h = Heatmap(
    merge_dat,
    col = colorRamp2(c(min, mid, max), c(color_down, "white", color_up)),
    color_space = "sRGB",
    na_col = "grey",
    cluster_rows = dend_rows,
    cluster_columns = dend_cols,
    clustering_distance_columns = distance_measure,
    clustering_distance_rows = distance_measure,
    clustering_method_columns = clustering_method,
    clustering_method_rows = clustering_method,
    column_dend_side = "top",
    column_dend_height = unit(3, "cm"),
    column_names_side = "top",
    show_row_names = "FALSE",
    #row_names_side = "left",
    #row_names_max_width = unit(15, "cm"),
    column_names_max_height = unit(6, "cm"),
    show_row_dend = TRUE,
    #row_title_gp = gpar(cex = 0.6),
    column_names_gp = gpar(cex = 1, fontface = "bold"),
    #column_dend_reorder=c(1,3,4,5,2),
    #row_dend_reorder=F,
    heatmap_legend_param = list(
      title = "z-score",
      title_gp = gpar(cex = 1, fontface = "bold"),
      color_bar = c("continuous")
    ),
    show_heatmap_legend = TRUE
  )

  print(h)

  dev.off()

  #plot with discrete breaks
discrete_sequence = seq(from = min, to = max, by = 0.5)
discrete_colors = colorRampPalette(colors = c(color_down,
                                              "white",
color_up))(length(discrete_sequence))

  plot_file = "Heatmap_zscores_DEproteins_discrete.pdf"
  plot_path = paste(plots_dir, plot_file, sep = dirsep)
  pdf(file = plot_path,
      width = dim(merge_dat)[2],
      height = 8)

  h = Heatmap(
  merge_dat,
  col = colorRamp2(breaks = discrete_sequence, colors = discrete_colors),
  color_space = "sRGB",
  na_col = "grey",
  cluster_rows = dend_rows,
  cluster_columns = dend_cols,
  clustering_distance_columns = distance_measure,
  clustering_distance_rows = distance_measure,
  clustering_method_columns = clustering_method,
```

```
  clustering_method_rows = clustering_method,
  column_dend_side = "top",
  column_dend_height = unit(3, "cm"),
  column_names_side = "top",
  show_row_names = "FALSE",
  #row_names_side = "left",
  #row_names_max_width = unit(15, "cm"),
  column_names_max_height = unit(6, "cm"),
  show_row_dend = TRUE,
  row_dend_width = unit(2, "cm"),
  #row_title_gp = gpar(cex = 0.6),
  column_names_gp = gpar(cex = 1, fontface = "bold"),
  #column_dend_reorder=c(3,2,1,7,4,5,6),
  #row_dend_reorder=F,
  heatmap_legend_param = list(
    title = "z-score",
    at = seq(from = min, to = max, by = max/2),
    title_gp = gpar(cex = 1, fontface = "bold"),
    color_bar = c("discrete")
  ),
  show_heatmap_legend = TRUE
)

print(h)

dev.off()

plot_file = "Heatmap_zscores_DEproteins_discrete.wmf"
  plot_path = paste(plots_dir, plot_file, sep = dirsep)
  win.metafile(file = plot_path,
      width = dim(merge_dat)[2],
      family = define_font,
      height = 8)

  print(h)

dev.off()

plot_file = "Heatmap_zscores_DEproteins_discrete.svg"
  plot_path = paste(plots_dir, plot_file, sep = dirsep)
  svg(file = plot_path,
      width = dim(merge_dat)[2],
      family = define_font,
      height = 8)

  print(h)

dev.off()

plot_file = "Heatmap_zscores_DEproteins_discrete.png"
  plot_path = paste(plots_dir, plot_file, sep = dirsep)
  png(file = plot_path,
      width = dim(merge_dat)[2],
      family = define_font,
      units = "in",
      res = 800,
      height = 8)

  print(h)

dev.off()
```

3 Assuming controls to be 0

```{r prepare control sample with NA to 0}
#extract data of control sample
control_sample = get(paste("Replicates_reliable_", controlsample, sep = ""))

#replace all NAs with 0 in cases were all values are NA in control sample
```

```
for (i in 1:nrow(control_sample)){
  if (sum(is.na(control_sample[i, ])) == ncol(control_sample)){
    get_vector = control_sample[i, ]
    get_vector = gsub(get_vector, pattern = "NA", replacement = "0")
    control_sample[i, ] = as.numeric(get_vector)
  }
}

dat_subset = control_sample[apply(control_sample,1,function(x) all(x==0, na.rm =
TRUE)), ]

dat_unique_for_treatment = dat_subset
```


```{r calculation proteins with ctrl 0}
#create a list of data to use for calculation
sample_list = ls(pattern = "^Replicates_reliable_")

#calculate means for control sample
Mean_ctrl = apply(control_sample, 1, function(x) {
  mean(x, na.rm = TRUE)
})

for (i in sample_list) {
  #get all replicates for treatment
  get_dat = get(i)

  #store accessions
  accessions = row.names(get_dat)

  #define dimensions
  nReps = dim(get_dat)[2]
  nRows = dim(get_dat)[1]

  #count NAs per row
  NACounts = apply(get_dat, 1, function(x) {
    sum(is.na(x))
  })

  #determine in how many replicates protein was quantified
  nCounts = dim(get_dat)[2] - NACounts

  #calculate mean
  Mean = apply(get_dat, 1, function(x) {
    mean(x, na.rm = TRUE)
  })

  #calculate SD
  SD = apply(get_dat, 1, function(x) {
    sd(x, na.rm = TRUE)
  })

  #calculate FC against control
  FC = Mean - Mean_ctrl

  #summarize replicate data and calculation results
  get_dat = as.data.frame(cbind(get_dat, nCounts, Mean, SD, FC))
  #get_dat = get_dat[which(get_dat$nCounts > 1), ]

  #perform Student's t-test against control
  #get data from replicates and control
  get_dat_ttest = get_dat[, 1:nReps]
  nZero = dim(get_dat_ttest)[1]
  zero_matrix = control_sample
  get_dat_ttest = as.data.frame(cbind(get_dat_ttest, zero_matrix))
  #define dimensions
  nReps_next = nReps + 1
  nCols = dim(get_dat_ttest)[2]
  #determine p-value and return NA if t-test fails
```

````
  pvalue = apply(get_dat_ttest, 1, function(x) {
    obj = try(t.test(
      x[1:nReps],
      x[nReps_next:nCols],
      alternative = "two.sided",
      var.equal = TRUE,
      paired = FALSE
    ),
    silent = TRUE)
    if (is(obj, "try-error"))
      return(NA)
    else
      return(obj$p.value)
  })

  #adjust p-values using FDR (same as Benjamini & Hochberg)
  pvalue.adj = p.adjust(pvalue, method = "fdr")

  #add results to summary table
  calculation_results = as.data.frame(cbind(get_dat, pvalue, pvalue.adj))

  #store summary table in environment
  assign(x = paste("CalculatedCtrl0", i, sep = "_"), value = calculation_results)
}

#clean workspace
remove(Mean_ctrl)
```

Summarize data for all treatments.

```{r cbind data proteins ctrl 0}
summary_calculated_data = do.call(qpcR:::cbind.na,
                                  lapply(ls(pattern =
"CalculatedCtrl0_Replicates_"),
                                                          function(x){
                                                            get_dat = get(x)
                                                            get_dat
                                                          }))

summary_calculated_data_short = merge(dat_subset, summary_calculated_data,
                                      by = "row.names")

rownames(summary_calculated_data_short) = summary_calculated_data_short$Row.names
summary_calculated_data_short = summary_calculated_data_short[, -1]
removing_vector = seq(1, ncol(dat_subset))
summary_calculated_data_short = summary_calculated_data_short[, -removing_vector]

filename = paste(Rdata_dir, "dat_calculated_cbind_proteins_ctrl0.csv", sep = "/")
write.csv(file = filename,x = summary_calculated_data_short)
```

### 3.1 Extract data

```{r extract protein dat for IPA ctrl 0}
sample_list = ls(pattern = "CalculatedCtrl0_Replicates_")
sample_names = gsub("CalculatedCtrl0_Replicates_", "", sample_list)
sample_names = gsub("reliable_", "", sample_names)
sample_names_mean = paste(sample_names)
sample_names_pvalue = paste(sample_names, "pvalue", sep = "_")

for (i in 1:length(sample_list)) {
  get_dat = get(sample_list[[i]])
  #extract FCs and p-values
  #change this to pvalue.adj if you want the adjusted p-values
  #change this to pvalue if you want the NOT adjusted p-values
  #dat_mean = as.data.frame(cbind(get_dat$FC, get_dat$pvalue))
  dat_mean = as.data.frame(cbind(get_dat$FC, get_dat[, pvalue_decision]))

  #store data in environment
````

```
  colnames(dat_mean) = c(sample_names_mean[i], sample_names_pvalue[i])
  accessions = rownames(get_dat)
  row.names(dat_mean) = accessions
  assign(x = paste("IPActrl0", sample_names[i], sep = "_"),
         value = dat_mean)
}

#summarize FCs and p-values for all treatments
summary_IPA = do.call(qpcR:::cbind.na, lapply(ls(pattern = "IPActrl0_"),
                                              function(x) {
                                                get_dat = get(x)
                                                get_dat
                                              }))


summary_IPA[summary_IPA == "NaN"] = NA
#remove all rows that contain only NA and save data
#summary_IPA = summary_IPA[rowSums(is.na(summary_IPA)) != ncol(summary_IPA),]

summary_IPA_short = merge(dat_subset, summary_IPA,
                                      by = "row.names")

rownames(summary_IPA_short) = summary_IPA_short$Row.names
summary_IPA_short = summary_IPA_short[, -1]
summary_IPA_short = summary_IPA_short[, -removing_vector]

filename = paste(Rdata_dir, paste("dat_IPA_cbind_proteins_Ctrl0_", pvalue_decision,
".csv", sep = ""), sep = "/")
write.csv(file = filename, x = summary_IPA_short)

filename = paste(Rdata_dir, paste("dat_IPA_cbind_proteins_Ctrl0_", pvalue_decision,
".xlsx", sep = ""), sep = "/")
write.xlsx(file = filename, x = summary_IPA_short)

#export FCs and p-values together with gene names
summary_IPA_genes = merge(summary_IPA_short, sampleGenes, by = "row.names", all.x =
TRUE)
rownames(summary_IPA_genes) = summary_IPA_genes$Row.names
summary_IPA_genes = summary_IPA_genes[, -1]

filename = paste(Rdata_dir, paste("dat_IPA_cbind_proteins_genes_Ctrl0_",
pvalue_decision, ".csv", sep = ""), sep = "/")
write.csv(file = filename,x = summary_IPA_genes)

filename = paste(Rdata_dir, paste("dat_IPA_cbind_proteins_genes_Ctrl0_",
pvalue_decision, ".xlsx", sep = ""), sep = "/")
write.xlsx(file = filename,x = summary_IPA_genes)
```

Plot counts of proteins that were unique for treatments.

```{r plot of identified proteins - unique for treatment}
dat_to_plot = summary_IPA_short

dat_to_plot = dat_to_plot[, which(grepl(colnames(dat_to_plot), pattern = "_pvalue")
== FALSE)]

dat_count = apply(dat_to_plot, 2, function(x){
  sum(!is.na(x))
})

dat_to_plot_long = as.data.frame(dat_count)
dat_to_plot_long$Sample = rownames(dat_to_plot_long)

max_count = round_any(max(dat_to_plot_long$dat_count), 100, f = ceiling)

plot_file = paste("NumberIdentifiedProteins_UniqueForTreatment", ".wmf", sep = "")
  plot_path = paste(plots_dir, plot_file, sep = dirsep)
  win.metafile(file = plot_path,
               width = 5, height = 3,
```

```
                pointsize = 12,
                family = define_font)

gg = ggplot(data=dat_to_plot_long, aes(x=Sample, y = dat_count, fill = Sample)) +
  geom_bar(stat = "identity")+
  #scale_fill_npg() +
  scale_fill_manual(values = npg_colors) +
    scale_y_continuous(limits = c(0, max_count), expand = c(0, 0),
                       breaks = c(0, max_count/2, max_count)) +
    theme_classic() +
    theme(axis.title.y = element_blank(),
          axis.title.x = element_blank(),
          axis.text.x = element_text(size = rel(1), color = "black", face = "bold",
                                     angle = 45, vjust = 1, hjust = 1),
          axis.text.y = element_text(size = rel(1), color = "black", hjust = 1,
face = "bold"),
          axis.line = element_line(colour = "black", size = 1),
          axis.ticks = element_line(colour = "black", size = 1),
          legend.title = element_blank(),
          panel.spacing = unit(0.5, "cm"),
          plot.margin = margin(t = 0.5, r = 0.5, b = 0.5, l = 0.5, "cm"),
          plot.title = element_text(size = rel(1.5), face = "bold",
                                    color = "black", hjust = 0.5)) +
    ggtitle("Number of identified proteins")

print(gg)
dev.off()
```

3.2 Plot data

3.2.1 Heatmap

```{r heatmap of data - unique for treatment}

heatmap_abundances_df = summary_IPA_short[,
which(grepl(colnames(summary_IPA_short),
                                                        pattern = "_pvalue") ==
FALSE &

grepl(colnames(summary_IPA_short),
                                                        pattern = controlsample) ==
FALSE)]

#define limits
max_heatmap = max(heatmap_abundances_df, na.rm = TRUE)*0.9
max = ceiling(max_heatmap)

# create empty vector that will be filled with median values
median_abundance_vector = vector(mode = "expression",
                                 length = length(colnames(heatmap_abundances_df)))

for (j in 1:length(colnames(heatmap_abundances_df))){
  M = na.omit(heatmap_abundances_df[,j])
  median_abundance_vector[j] = median(M)
}
head(median_abundance_vector)

mid = round(median(unlist(lapply(median_abundance_vector, as.numeric))), digits =
0)

min = mid - (max - mid)

dat_to_plot = heatmap_abundances_df

#replace NAs by 0
heatmap_abundances_df[is.na(heatmap_abundances_df)] = 0

dend_rows = dendsort(hclust(dist(heatmap_abundances_df))) #can't be used with NAs
dend_cols = dendsort(hclust(dist(t(heatmap_abundances_df))))
```

```
dend_cols = color_branches(dend_cols, k = floor(dim(heatmap_abundances_df)[2] / 2))

cluster_membership = cutree(dend_rows, ifelse(dim(heatmap_abundances_df)[1] < 10,
2,

ceiling(dim(heatmap_abundances_df)[1]/10)))

summary_IPA_short$Order = dend_rows[[3]]
summary_IPA_short$Cluster = cluster_membership

filename = paste(Rdata_dir, "dat_IPA_cbind_proteins_Ctrl0_Clusters.csv", sep = "/")
write.csv(file = filename, x = summary_IPA_short)

plot_file = "Heatmap_AbundanceMeans_UniqueForTreatment.pdf"
plot_path = paste(plots_dir, plot_file, sep = dirsep)
pdf(file = plot_path,
    width = dim(dat_to_plot)[2] + 2,
    height = 8)

h = Heatmap(
  dat_to_plot,
  col = colorRamp2(c(min, mid, max), c(color_low, color_mid, color_high)),
  color_space = "sRGB",
  na_col = "grey",
  cluster_rows = dend_rows,
  cluster_columns = dend_cols,
  column_dend_side = "top",
  column_dend_height = unit(3, "cm"),
  column_names_side = "top",
  show_row_names = "FALSE",
  #row_names_side = "left",
  #row_names_max_width = unit(15, "cm"),
  column_names_max_height = unit(6, "cm"),
  show_row_dend = TRUE,
  #row_title_gp = gpar(cex = 0.6),
  column_names_gp = gpar(cex = 1, fontface = "bold"),
  #column_dend_reorder=c(1,3,4,5,2),
  #row_dend_reorder=F,
  heatmap_legend_param = list(
    title = "Log2(Abundance)",
    title_gp = gpar(cex = 1, fontface = "bold"),
    at = c(min, mid, max),
    labels = c(min, mid, max),
    color_bar = c("continuous")
  ),
  show_heatmap_legend = TRUE
)

print(h)

dev.off()

#plot with discrete breaks
discrete_sequence = seq(from = min, to = max, by = 1)
discrete_colors = colorRampPalette(colors = c(color_low, color_mid,

color_high))(length(discrete_sequence))

plot_file = "Heatmap_AbundanceMeans_UniqueForTreatment_discrete.pdf"
plot_path = paste(plots_dir, plot_file, sep = dirsep)
pdf(file = plot_path,
    width = dim(dat_to_plot)[2] + 2,
    height = 8)

h = Heatmap(
  dat_to_plot,
  col = colorRamp2(breaks = discrete_sequence, colors = discrete_colors),
  color_space = "sRGB",
  na_col = "grey",
  cluster_rows = dend_rows,
```

```
  cluster_columns = dend_cols,
  clustering_distance_columns = distance_measure,
  clustering_distance_rows = distance_measure,
  clustering_method_columns = clustering_method,
  clustering_method_rows = clustering_method,
  column_dend_side = "top",
  column_dend_height = unit(3, "cm"),
  column_names_side = "top",
  show_row_names = "FALSE",
  #row_names_side = "left",
  #row_names_max_width = unit(15, "cm"),
  column_names_max_height = unit(6, "cm"),
  show_row_dend = TRUE,
  row_dend_width = unit(2, "cm"),
  #row_title_gp = gpar(cex = 0.6),
  column_names_gp = gpar(cex = 1, fontface = "bold"),
  #column_dend_reorder=c(3,2,1,7,4,5,6),
  #row_dend_reorder=F,
  heatmap_legend_param = list(
    title = "Log2(Abundance)",
    at = seq(from = min, to = max, by = 1),
    title_gp = gpar(cex = 1, fontface = "bold"),
    color_bar = c("discrete")
  ),
  show_heatmap_legend = TRUE
)

print(h)

dev.off()

#plot with discrete breaks
cluster_sequence = unique(cluster_membership)
cluster_colors = colorRampPalette(colors = c("lightgrey",
"#252525"))(length(cluster_sequence))

plot_file = "Heatmap_AbundanceMeans_UniqueForTreatment_discrete_SplitClusters.pdf"
plot_path = paste(plots_dir, plot_file, sep = dirsep)
pdf(file = plot_path,
    width = dim(dat_to_plot)[2] + 2,
    height = 8)

h = Heatmap(
  dat_to_plot,
  col = colorRamp2(breaks = discrete_sequence, colors = discrete_colors),
  color_space = "sRGB",
  na_col = "grey",
  cluster_rows = dend_rows,
  cluster_columns = dend_cols,
  clustering_distance_columns = distance_measure,
  clustering_distance_rows = distance_measure,
  clustering_method_columns = clustering_method,
  clustering_method_rows = clustering_method,
  column_dend_side = "top",
  column_dend_height = unit(3, "cm"),
  column_names_side = "top",
  show_row_names = "FALSE",
  #row_names_side = "left",
  #row_names_max_width = unit(15, "cm"),
  column_names_max_height = unit(6, "cm"),
  show_row_dend = TRUE,
  row_dend_width = unit(2, "cm"),
  #row_title_gp = gpar(cex = 0.6),
  column_names_gp = gpar(cex = 1, fontface = "bold"),
  #column_dend_reorder=c(3,2,1,7,4,5,6),
  #row_dend_reorder=F,
  heatmap_legend_param = list(
    title = "Log2(Abundance)",
    at = seq(from = min, to = max, by = 1),
    title_gp = gpar(cex = 1, fontface = "bold"),
```

```
    color_bar = c("discrete")
  ),
  show_heatmap_legend = TRUE
) +
  Heatmap(cluster_membership,
          name = "Cluster",
            color_space = "sRGB",
            col = colorRamp2(breaks = cluster_sequence, colors = cluster_colors),
    na_col = "grey",
    column_title_side = "top",
    show_row_names = "FALSE",
    width = unit(0.5, "cm"),
    column_names_gp = gpar(cex = 1, fontface = "bold"),
      heatmap_legend_param = list(
      title = "Cluster",
      at = cluster_sequence,
      title_gp = gpar(cex = 1, fontface = "bold"),
      color_bar = c("discrete")
    ))

print(h)

dev.off()

plot_file = "Heatmap_AbundanceMeans_UniqueForTreatment_discrete_SplitClusters.wmf"
plot_path = paste(plots_dir, plot_file, sep = dirsep)
win.metafile(file = plot_path,
    width = dim(dat_to_plot)[2] + 2,
    family = define_font,
    height = 8)

print(h)

dev.off()

plot_file = "Heatmap_AbundanceMeans_UniqueForTreatment_discrete_SplitClusters.svg"
plot_path = paste(plots_dir, plot_file, sep = dirsep)
svg(file = plot_path,
    width = dim(dat_to_plot)[2] + 2,
    family = define_font,
    height = 8)

print(h)

dev.off()

plot_file = "Heatmap_AbundanceMeans_UniqueForTreatment_discrete_SplitClusters.png"
plot_path = paste(plots_dir, plot_file, sep = dirsep)
png(file = plot_path,
    units = "in",
    res = 800,
    width = dim(dat_to_plot)[2] + 2,
    family = define_font,
    height = 8)

print(h)

dev.off()
```

```
## 4 Use imputed data for proteins that were unique for certain treatments

```{r connect vsn data and imputed data for proteins unique for at least one treatment}
sample_vector = unique(gsub("(.*)_.*","\\1", colnames(dat_stabilized)))

dat_imputed = get_df_wide(data_imp)
row.names(dat_imputed) = dat_imputed$Names
dat_imputed = dat_imputed[, -1]
dat_imputed = dat_imputed[, ncol_vsn]
```
```

```
#get imputed data for control sample
get_dat = dat_stabilized[, grepl(colnames(dat_stabilized), pattern =
controlsample)]

get_imp = dat_imputed[, grepl(colnames(dat_imputed), pattern = controlsample)]

dat_imputed_info = rep(FALSE, nrow(get_dat))
dat_imputed_info[rowSums(is.na(get_dat)) == ncol(get_dat)] = TRUE

get_dat[rowSums(is.na(get_dat)) == ncol(get_dat), ] =
get_imp[rowSums(is.na(get_dat)) == ncol(get_dat), ]

assign(value = get_dat, x = paste("ExtractedImputedData", controlsample, sep =
"_"))

sample_vector = sample_vector[which(sample_vector != controlsample)]

for (i in sample_vector){

  get_dat = dat_stabilized[, grepl(colnames(dat_stabilized), pattern = i)]

  get_imp = dat_imputed[, grepl(colnames(dat_imputed), pattern = i)]

  get_dat[rowSums(is.na(get_dat)) == ncol(get_dat) & dat_imputed_info == FALSE, ] =
get_imp[rowSums(is.na(get_dat)) == ncol(get_dat) &
                                                                  dat_imputed_info
== FALSE, ]

  assign(value = get_dat, x = paste("ExtractedImputedData", i, sep = "_"))

}

dat_rbind = do.call(cbind, lapply(ls(pattern = "ExtractedImputedData_"),
                                  function(x) {
                                    get_dat = get(x)
                                    get_dat
                                  }))

filename = "dat_relreps_imputed.csv"
pathname = paste(Rdata_dir, filename, sep = dirsep)

write.csv(x = dat_rbind, file = pathname)

dat_to_calculate = dat_rbind
```

Count identified proteins in imputed data.

```{r plot of identified proteins in imputed data - reliable replicates}
dat_to_plot = dat_to_calculate

dat_count = apply(dat_to_plot, 2, function(x){
  sum(!is.na(x))
})

dat_to_plot_long = as.data.frame(dat_count)
dat_to_plot_long$Sample = rownames(dat_to_plot_long)

max_count = round_any(max(dat_to_plot_long$dat_count), 500, f = ceiling)

dat_to_plot_long$Class = gsub("(.*)_.*", "\\1", row.names(dat_to_plot_long))

plot_file = paste("NumberIdentifiedProteins_Imputed", ".wmf", sep = "")
  plot_path = paste(plots_dir, plot_file, sep = dirsep)
  win.metafile(file = plot_path,
               width = 5, height = 3,
               pointsize = 12,
```

```
               family = define_font)

gg = ggplot(data=dat_to_plot_long, aes(x=Sample, y = dat_count, fill = Class)) +
  geom_bar(stat = "identity")+
  #scale_fill_npg() +
  scale_fill_manual(values = npg_colors) +
    scale_y_continuous(limits = c(0, max_count), expand = c(0, 0),
                       breaks = c(0, max_count/2, max_count)) +
    theme_classic() +
    theme(axis.title.y = element_blank(),
          axis.title.x = element_blank(),
          axis.text.x = element_text(size = rel(1), color = "black", face = "bold",
                                     angle = 45, vjust = 1, hjust = 1),
          axis.text.y = element_text(size = rel(1), color = "black", hjust = 1,
face = "bold"),
          axis.line = element_line(colour = "black", size = 1),
          axis.ticks = element_line(colour = "black", size = 1),
          legend.title = element_blank(),
          panel.spacing = unit(0.5, "cm"),
          plot.margin = margin(t = 0.5, r = 0.5, b = 0.5, l = 0.5, "cm"),
          plot.title = element_text(size = rel(1.5), face = "bold",
                                    color = "black", hjust = 0.5)) +
    ggtitle("Number of identified proteins")

print(gg)
dev.off()
```

Check for sample quality in imputed data by using another Sample-2-Sample distance correlation.

```
```{r sample2sample description matrix of imputed data}
#transpose data for sample2sample distance plotting
x = t(dat_to_calculate)

sampleDists = dist(x)
m = as.matrix(dist(x))

plot_file = "Sample2Sample-Distances_imputed.wmf"
plot_path = paste(plots_dir, plot_file, sep = dirsep)
win.metafile(file = plot_path,
      width = 15, height = 15,
      pointsize = 10,
      family = define_font)

ht = Heatmap(m,
        col = my_palette,
        color_space = "sRGB",
        #na_col="grey",
        cluster_rows = TRUE,
        cluster_columns= TRUE,
        clustering_distance_columns = "euclidean",
        #clustering_method_columns = "complete",
        column_dend_side="top",
        column_dend_height= unit(3,"cm"),
        column_names_side="top",
        show_row_names = TRUE,
        row_names_side = "left",
        row_names_max_width = unit(10, "cm"),
        column_names_max_height = unit(10, "cm"),
        show_row_dend = TRUE,
        row_dend_width = unit(3,"cm"),
        row_names_gp = gpar(cex = 1.5, fontface = "bold"),
        column_names_gp = gpar(cex = 1.5, fontface = "bold"),
        #column_dend_reorder=c(1,3,4,5,2),
        #row_dend_reorder=F,
        #split = find_rows$k,
        gap = unit(2, "mm"),
        # heatmap_legend_param = list(title = "-Log10(FC)",
        #                             title_gp = gpar(cex = 1, fontface = "bold"),
```
```

````
        #   color_bar = c("discrete"),
        #   at = c(0, 0.1, 1.3),
        #   labels = c("NA", "n.s.", "*")),
        show_heatmap_legend=FALSE)

print(ht)
dev.off()
```

Have a look at NA distribution in imputed data.

```{r heatmap NA distribution imputed data}

dat_to_plot = dat_to_calculate

dat_to_plot[!is.na(dat_to_plot)] = 0
dat_to_plot[is.na(dat_to_plot)] = 1

dend_rows = hclust(dist(dat_to_plot)) #can not be used with NAs
dend_cols = dendsort(hclust(dist(t(dat_to_plot))))
dend_cols = color_branches(dend_cols, k = floor(dim(dat_to_plot)[2]/2))

dat_matrix = as.matrix(dat_to_plot)

plot_file = "Heatmap_NA_Distribution_AfterImputation.wmf"
plot_path = paste(plots_dir, plot_file, sep = dirsep)
win.metafile(file = plot_path,
             width = dim(dat_to_plot)[2]/3+2, height = 8,
             pointsize = 10,
             family = define_font)

ht = Heatmap(dat_matrix,
             col = NA_palette,
             color_space = "sRGB",
             #na_col="grey",
             cluster_rows = dend_rows,
             cluster_columns= dend_cols,
             clustering_distance_columns = "euclidean",
             #clustering_method_columns = "complete",
             column_dend_side="top",
             column_dend_height= unit(3,"cm"),
             column_names_side="top",
             show_row_names = FALSE,
             #row_names_side = "left",
             #row_names_max_width = unit(10, "cm"),
             column_names_max_height = unit(10, "cm"),
             show_row_dend = FALSE,
             #row_dend_width = unit(3,"cm"),
             #row_names_gp = gpar(cex = 1.5, fontface = "bold"),
             column_names_gp = gpar(cex = 1.5, fontface = "bold"),
             #column_dend_reorder=c(1,3,4,5,2),
             #row_dend_reorder=F,
             #split = find_rows$k,
             #gap = unit(2, "mm"),
             heatmap_legend_param = list(title = "",
                                         title_gp = gpar(cex = 1, fontface =
"bold"),
                                         color_bar = c("discrete"),
                                         at = c(0, 1),
                                         labels_gp = gpar(cex = 1),
                                         labels = c("Value", "NA")),
             show_heatmap_legend=TRUE)

print(ht)
dev.off()

plot_file = "Heatmap_NA_Distribution_AfterImputation.pdf"
plot_path = paste(plots_dir, plot_file, sep = dirsep)
pdf(file = plot_path,
             width = dim(dat_to_plot)[2]/3+2, height = 8,
````

````
             pointsize = 10)

print(ht)
dev.off()

```

Extract data for Calculation.

```{r subset_replicates proteins imputed data}
sample_vector = unique(gsub("(.*)_.*","\\1", colnames(dat_to_calculate)))
for ( i in sample_vector){
  dat_subset = subset(dat_to_calculate, select = grepl(pattern = i,
colnames(dat_to_calculate)))
  assign(x = paste("ReplicatesIMPUTED_reliable", i, sep = "_"), value = dat_subset)
}
```

```{r calculation proteins imputed data}
#create a list of data to use for calculation
sample_list = ls(pattern = "^ReplicatesIMPUTED_reliable_")

#extract data of control sample
control_sample = get(paste("ReplicatesIMPUTED_reliable_", controlsample, sep = ""))

#calculate means for control sample
Mean_ctrl = apply(control_sample, 1, function(x) {
  if (length(na.exclude(x)) >= reliablereps){
    mean(x, na.rm = TRUE)} else NA
})

for (i in sample_list) {
  #get all replicates for treatment
  get_dat = get(i)

  #store accessions
  accessions = row.names(get_dat)

  #define dimensions
  nReps = dim(get_dat)[2]
  nRows = dim(get_dat)[1]

  #count NAs per row
  NACounts = apply(get_dat, 1, function(x) {
    sum(is.na(x))
  })

  #determine in how many replicates protein was quantified
  nCounts = dim(get_dat)[2] - NACounts

  #calculate mean
  Mean = apply(get_dat, 1, function(x) {
    if (length(na.exclude(x)) >= reliablereps){
      mean(x, na.rm = TRUE)} else NA
  })

  #calculate SD
  SD = apply(get_dat, 1, function(x) {
    if (length(na.exclude(x)) >= reliablereps){
      sd(x, na.rm = TRUE)} else NA
  })

  #calculate FC against control
  FC = Mean - Mean_ctrl

  #summarize replicate data and calculation results
  get_dat = as.data.frame(cbind(get_dat, nCounts, Mean, SD, FC))
  #get_dat = get_dat[which(get_dat$nCounts > 1), ]

  #perform Student's t-test against control
````

````
  #get data from replicates and control
  get_dat_ttest = get_dat[, 1:nReps]
  nZero = dim(get_dat_ttest)[1]
  zero_matrix = control_sample
  get_dat_ttest = as.data.frame(cbind(get_dat_ttest, zero_matrix))
  #define dimensions
  nReps_next = nReps + 1
  nCols = dim(get_dat_ttest)[2]
  #determine p-value and return NA if t-test fails

  pvalue = apply(get_dat_ttest, 1, function(x) {
    if (length(na.exclude(x[1:nReps])) >= reliablereps &
        length(na.exclude(x[nReps_next:nCols])) >= reliablereps){
      obj = try(t.test(
      x[1:nReps],
      x[nReps_next:nCols],
      alternative = "two.sided",
      var.equal = TRUE,
      paired = FALSE
    ),
    silent = TRUE)
    if (is(obj, "try-error")){
      return(NA)}
    else {
      return(obj$p.value)}}
    else NA
  })

  #adjust p-values using FDR (same as Benjamini & Hochberg)
  pvalue.adj = p.adjust(pvalue, method = "fdr")

  #add results to summary table
  calculation_results = as.data.frame(cbind(get_dat, pvalue, pvalue.adj))

  zscore_mean = mean(calculation_results$FC, na.rm = TRUE)
  zscore_sd = sd(calculation_results$FC, na.rm = TRUE)

  calculation_results$zscore = (calculation_results$FC - zscore_mean)/zscore_sd

  colnames(calculation_results)[nReps_next:ncol(calculation_results)] =
paste(gsub(i, pattern = "ReplicatesIMPUTED_reliable_", replacement = ""),
colnames(calculation_results)[nReps_next:ncol(calculation_results)], sep = "_")

  #store summary table in environment
  assign(x = paste("Calculated", i, sep = "_"), value = calculation_results)
}

#clean workspace
remove(Mean_ctrl)
```

Summarize data for all treatments.

```{r cbind data proteins imputed data}
summary_calculated_data = do.call(qpcR:::cbind.na, lapply(ls(pattern =

"Calculated_ReplicatesIMPUTED_"),
                                                          function(x){
                                                            get_dat = get(x)
                                                            get_dat
                                                          }))

filename = paste(Rdata_dir, "dat_calculated_cbind_proteins_imputed.csv", sep = "/")
write.csv(file = filename,x = summary_calculated_data)
```

### 4.1 Extract data

Extract the FCs for all treatments and summarize the values in one dataframe.
````
````

```
```{r extract FCs proteins imputed data}
sample_list = ls(pattern = "Calculated_ReplicatesIMPUTED_")
sample_names = gsub("Calculated_ReplicatesIMPUTED_", "", sample_list)
sample_names = gsub("reliable_", "", sample_names)

for (i in 1:length(sample_list)) {
  get_dat = get(sample_list[[i]])
  dat_mean = as.data.frame(get_dat[, grepl(colnames(get_dat), pattern = "_FC$")])
  colnames(dat_mean) = sample_names[i]
  accessions = rownames(get_dat)
  row.names(dat_mean) = accessions
  assign(x = paste("MeanIMPUTED", sample_names[i], sep = "_"),
         value = dat_mean)
}

summary_means = do.call(qpcR:::cbind.na, lapply(ls(pattern = "MeanIMPUTED_"),
                                                function(x) {
                                                  get_dat = get(x)
                                                  get_dat
                                                }))

head(summary_means)
boxplot(summary_means, las = 2)

filename = paste(Rdata_dir, "dat_means_cbind_proteins_imputed.csv", sep = "/")
write.csv(file = filename, x = summary_means)
```

Export the FCs.

```{r export FCs imputed data}
colnames(summary_means)

summary_means_short = summary_means

colnames(summary_means_short)

dat_means_short_proteins = summary_means_short

filename = paste(Rdata_dir, "dat_means_cbind_proteins_short_imputed.csv", sep =
"/")
write.csv(file = filename,x = summary_means_short)

boxplot(summary_means_short, las = 2)

analyte_to_dataset_mapping_proteins =
as.data.frame(rownames(dat_means_short_proteins))
analyte_to_dataset_mapping_proteins$Dataset = "Proteome"
colnames(analyte_to_dataset_mapping_proteins) = c("Analyte", "Dataset")
```

```{r plot of identified proteins - mean of FCs - imputed data}
dat_to_plot = summary_means_short

dat_count = apply(dat_to_plot, 2, function(x){
  sum(!is.na(x))
})

dat_to_plot_long = as.data.frame(dat_count)
dat_to_plot_long$Sample = rownames(dat_to_plot_long)

max_count = round_any(max(dat_to_plot_long$dat_count), 500, f = ceiling)

plot_file = paste("NumberIdentifiedProteins_FCMeans_imputed", ".wmf", sep = "")
  plot_path = paste(plots_dir, plot_file, sep = dirsep)
  win.metafile(file = plot_path,
               width = 5, height = 3,
               pointsize = 12,
               family = define_font)
```
```

````
gg = ggplot(data=dat_to_plot_long, aes(x=Sample, y = dat_count, fill = Sample)) +
  geom_bar(stat = "identity")+
  #scale_fill_npg() +
  scale_fill_manual(values = npg_colors) +
    scale_y_continuous(limits = c(0, max_count), expand = c(0, 0),
                       breaks = c(0, max_count/2, max_count)) +
    theme_classic() +
    theme(axis.title.y = element_blank(),
          axis.title.x = element_blank(),
          axis.text.x = element_text(size = rel(1), color = "black", face = "bold",
                                     angle = 45, vjust = 1, hjust = 1),
          axis.text.y = element_text(size = rel(1), color = "black", hjust = 1,
face = "bold"),
          axis.line = element_line(colour = "black", size = 1),
          axis.ticks = element_line(colour = "black", size = 1),
          legend.title = element_blank(),
          panel.spacing = unit(0.5, "cm"),
          plot.margin = margin(t = 0.5, r = 0.5, b = 0.5, l = 0.5, "cm"),
          plot.title = element_text(size = rel(1.5), face = "bold",
                                    color = "black", hjust = 0.5)) +
    ggtitle("Number of identified proteins")

print(gg)
dev.off()
```

Export FCs and adjusted p-values in a format that works for IPA as well as for all
other further analysis steps. Furthermore, a table is created that summarizes the
counts of significant changes (up and down).

```{r extract protein dat for IPA - imputed data}
sample_list = ls(pattern = "Calculated_ReplicatesIMPUTED_")
sample_names = gsub("Calculated_ReplicatesIMPUTED_", "", sample_list)
sample_names = gsub("reliable_", "", sample_names)
sample_names_mean = paste(sample_names)
sample_names_pvalue = paste(sample_names, "pvalue", sep = "_")

for (i in 1:length(sample_list)) {
  get_dat = get(sample_list[[i]])
  #extract FCs and p-values
  #change this to pvalue.adj if you want the adjusted p-values
  #change this to pvalue if you want the NOT adjusted p-values
  #dat_mean = as.data.frame(cbind(get_dat$FC, get_dat$pvalue))
  dat_mean = as.data.frame(cbind(get_dat[, grepl(colnames(get_dat), pattern =
"_FC$")],
                                 get_dat[, grepl(colnames(get_dat), pattern =
paste("_", pvalue_decision,

"$", sep = ""))]))

  #count significantly up/down regulated proteins
  dat_up = dim(dat_mean[which(dat_mean[, 2] < 0.05 &
                                dat_mean[, 1] > 0),])[1]
  dat_down = dim(dat_mean[which(dat_mean[, 2] < 0.05 &
                                  dat_mean[, 1] < 0),])[1]
  dat_not = dim(dat_mean[which(dat_mean[, 2] >= 0.05),])[1]
  dat_sum = dat_up + dat_down + dat_not

  #summarize data in dataframe and save dataframe
  dat_count_summary = as.data.frame(cbind(sample_names[i], dat_up, dat_not,
dat_down, dat_sum))
  colnames(dat_count_summary) = c("Treatment", "up", "none", "down", "sum")
  assign(x = paste("RegulationCountIMPUTED", sample_names[i], sep = "_"),
         value = dat_count_summary)

  #store data in environment
  colnames(dat_mean) = c(sample_names_mean[i], sample_names_pvalue[i])
  accessions = rownames(get_dat)
  row.names(dat_mean) = accessions
  assign(x = paste("IPAimputed", sample_names[i], sep = "_"),
````

```
        value = dat_mean)
}

#summarize FCs and p-values for all treatments
summary_IPA = do.call(qpcR:::cbind.na, lapply(ls(pattern = "^IPAimputed_"),
                                              function(x) {
                                                get_dat = get(x)
                                                get_dat
                                              }))

#remove all rows that contain only NA and save data
summary_IPA[summary_IPA == "NaN"] = NA
summary_IPA = summary_IPA[rowSums(is.na(summary_IPA)) != ncol(summary_IPA),]

filename = paste(Rdata_dir, paste("dat_IPA_cbind_proteins_imputed_",
pvalue_decision, ".csv", sep = ""), sep = "/")
write.csv(file = filename, x = summary_IPA)

filename = paste(Rdata_dir, paste("dat_IPA_cbind_proteins_imputed_",
pvalue_decision, ".xlsx", sep = ""), sep = "/")
write.xlsx(file = filename, x = summary_IPA)

#export FCs and p-values together with gene names
summary_IPA_genes = merge(summary_IPA, sampleGenes, by = "row.names", all.x = TRUE)
rownames(summary_IPA_genes) = summary_IPA_genes$Row.names
summary_IPA_genes = summary_IPA_genes[, -1]

filename = paste(Rdata_dir, paste("dat_IPA_cbind_proteins_imputed_genes_",
pvalue_decision, ".csv", sep = ""),
                 sep = "/")
write.csv(file = filename,x = summary_IPA_genes)

filename = paste(Rdata_dir, paste("dat_IPA_cbind_proteins_imputed_genes_",
pvalue_decision, ".xlsx", sep = ""),
                 sep = "/")
write.xlsx(file = filename,x = summary_IPA_genes)

#summarize regulation counts for all treatments and save them
summary_CalculationCount = do.call(rbind, lapply(ls(pattern =
"RegulationCountIMPUTED_"),
                                                 function(x) {
                                                   get_dat = get(x)
                                                   get_dat
                                                 }))

filename = paste(Rdata_dir, paste("dat_RegulationCount_proteins_imputed_",
pvalue_decision, ".csv", sep = ""),
                 sep = "/")
write.csv(file = filename, x = summary_CalculationCount)
```

```{r plot stacked bar plots of regulation counts imputed data}
sample_dat_proteins = summary_CalculationCount

sample_dat_proteins = sample_dat_proteins[sample_dat_proteins$Treatment !=
controlsample, ]

treatment_vector = sample_dat_proteins$Treatment

sample_dat_proteins = as.data.frame(apply(sample_dat_proteins, 2, function(x){
  as.numeric(as.character(x))
}))

sample_dat_proteins$Treatment = treatment_vector

sample_dat_proteins$up = sample_dat_proteins$up/sample_dat_proteins$sum*100
sample_dat_proteins$none = sample_dat_proteins$none/sample_dat_proteins$sum*100
sample_dat_proteins$down = sample_dat_proteins$down/sample_dat_proteins$sum*100
sample_dat_proteins$sum = sample_dat_proteins$up + sample_dat_proteins$none +
sample_dat_proteins$down
```

```
samples = unique(sample_dat_proteins$Treatment)

sample_dat_proteins = sample_dat_proteins[, -5]

sample_dat_proteins_reshape = melt(sample_dat_proteins, id.var = "Treatment")

sample_dat_proteins_reshape$Treatment =
as.character(sample_dat_proteins_reshape$Treatment)
sample_dat_proteins_reshape$variable =
as.character(sample_dat_proteins_reshape$variable)

  get_dat = sample_dat_proteins_reshape
  get_dat = get_dat[order(get_dat$variable), ]

  get_dat$variable = factor(get_dat$variable, levels = c("up", "none", "down"))

  ggplot_title = "Significantly altered proteins"

  plotfile = paste(plots_dir, paste("RegulationCount_Stacked_Imputed", ".wmf", sep
= ""), sep=dirsep)
  win.metafile(file = plotfile,
      width = length(samples)/2 + 2, height = 2,
      pointsize = 12,
      family = define_font)

  gg = ggplot(data = get_dat, aes(x = Treatment, y = value, fill = variable)) +
  geom_bar(stat = "identity") +
    scale_fill_manual(values = c(color_up, "grey", color_down))+
    scale_y_continuous(expand = c(0,0)) +
    theme_classic() +
    theme(axis.title.y = element_text(size = rel(1), face = "bold", color =
"black"),
            axis.title.x = element_blank(),
            axis.text.x = element_text(size = rel(1), color = "black", face =
"bold", angle = 45,
                                       vjust = 1, hjust = 1),
            axis.text.y = element_text(size = rel(1), color = "black", hjust = 1,
face = "bold"),
          axis.line = element_line(colour = "black", size = 1),
          axis.ticks = element_line(colour = "black", size = 1),
          legend.title = element_blank(),
          #plot.margin = margin(1,1,1,1, "cm"),
          plot.title = element_text(size = rel(1), face = "bold", color = "black",
hjust = 0.5)) +
    ggtitle(ggplot_title) +
    ylab("Percent Proteins")

  print(gg)

  dev.off()

  plotfile = paste(plots_dir, paste("RegulationCount_Stacked_Imputed", ".pdf", sep
= ""), sep=dirsep)
  pdf(file = plotfile,
      width = 4, height = 2,
      pointsize = 12)

   print(gg)

  dev.off()
```

````
```{r extract z-score data of imputed data}
sample_list = ls(pattern = "Calculated_ReplicatesIMPUTED_")
sample_names = gsub("Calculated_ReplicatesIMPUTED_", "", sample_list)
sample_names = gsub("reliable_", "", sample_names)
sample_names_mean = paste(sample_names)
sample_names_pvalue = paste(sample_names, "pvalue", sep = "_")
````
````

```
for (i in 1:length(sample_list)) {
  get_dat = get(sample_list[[i]])
  #extract FCs and p-values
  #change this to pvalue.adj if you want the adjusted p-values
  #change this to pvalue if you want the NOT adjusted p-values
  #dat_mean = as.data.frame(cbind(get_dat$zscore, get_dat$pvalue))
  dat_mean = as.data.frame(cbind(get_dat[, grepl(colnames(get_dat), pattern =
"_zscore$")],
                                 get_dat[, grepl(colnames(get_dat), pattern =
paste("_", pvalue_decision,

"$", sep = ""))]))

  #count significantly up/down regulated proteins
  dat_up = dim(dat_mean[which(dat_mean[, 2] < 0.05 &
                                dat_mean[, 1] > 0),])[1]
  dat_down = dim(dat_mean[which(dat_mean[, 2] < 0.05 &
                                  dat_mean[, 1] < 0),])[1]
  dat_not = dim(dat_mean[which(dat_mean[, 2] >= 0.05),])[1]
  dat_sum = dat_up + dat_down + dat_not

  #summarize data in dataframe and save dataframe
  dat_count_summary = as.data.frame(cbind(sample_names[i], dat_up, dat_not,
dat_down, dat_sum))
  colnames(dat_count_summary) = c("Treatment", "up", "none", "down", "sum")
  assign(x = paste("RegulationCountIMPUTED", sample_names[i], sep = "_"),
         value = dat_count_summary)

  #store data in environment
  colnames(dat_mean) = c(sample_names_mean[i], sample_names_pvalue[i])
  accessions = rownames(get_dat)
  row.names(dat_mean) = accessions
  assign(x = paste("IPAimputed", sample_names[i], sep = "_"),
         value = dat_mean)
}

#summarize FCs and p-values for all treatments
summary_zscore = do.call(qpcR:::cbind.na, lapply(ls(pattern = "^IPAimputed_"),
                                              function(x) {
                                                get_dat = get(x)
                                                get_dat
                                              }))

#remove all rows that contain only NA and save data
summary_zscore[summary_zscore == "NaN"] = NA

summary_zscore = summary_zscore[rowSums(is.na(summary_zscore)) <
ncol(summary_zscore), ]

filename = paste(Rdata_dir, paste("dat_zscore_", pvalue_decision, "_imputed.csv",
sep = ""), sep = "/")
write.csv(file = filename, x = summary_zscore)

filename = paste(Rdata_dir, paste("dat_zscore_", pvalue_decision, "_imputed.xlsx",
sep = ""), sep = "/")
write.xlsx(file = filename, x = summary_zscore)

#export FCs and p-values together with gene names
summary_IPA_genes = merge(summary_zscore, sampleGenes, by = "row.names", all.x =
TRUE)
rownames(summary_IPA_genes) = summary_IPA_genes$Row.names
summary_IPA_genes = summary_IPA_genes[, -1]

filename = paste(Rdata_dir, paste("dat_zscore_", pvalue_decision,
"_imputed_genes.csv", sep = ""), sep = "/")
write.csv(file = filename,x = summary_IPA_genes)

filename = paste(Rdata_dir, paste("dat_zscore_", pvalue_decision,
"_imputed_genes.xlsx", sep = ""), sep = "/")
```

~~~~
write.xlsx(file = filename,x = summary_IPA_genes)

#summarize regulation counts for all treatments and save them
summary_CalculationCount = do.call(rbind, lapply(ls(pattern =
"RegulationCountIMPUTED_"),
                                                 function(x) {
                                                   get_dat = get(x)
                                                   get_dat
                                                 }))

filename = paste(Rdata_dir, paste("dat_RegulationCount_proteins_zscores_",
pvalue_decision, "_imputed.csv", sep = ""),
                 sep = "/")
write.csv(file = filename, x = summary_CalculationCount)
```

### 4.2 Volcano Plots
#### 4.2.1 Volcano Wrap

```{r volcano facets imputed data, echo=FALSE, message=FALSE, warning=FALSE,
paged.print=FALSE}
#choose data set
sample_dat_FC_pvalue = summary_IPA

#exclude control because it contains only 1 and 0 values
sample_dat_FC_pvalue = sample_dat_FC_pvalue[,
which(grepl(colnames(sample_dat_FC_pvalue), pattern = controlsample) == FALSE)]

#create sample vector
sample_vector = unique(gsub(
  colnames(sample_dat_FC_pvalue),
  pattern = "_pvalue",
  replacement = ""
))

#bring data to long format, which is necessary for ggplot
for (j in sample_vector) {
  tryCatch({
    dat_subset = as.data.frame(sample_dat_FC_pvalue[, grepl(j,

colnames(sample_dat_FC_pvalue))])
    dat_subset$Treatment = j

    dat_merge = merge(dat_subset, sampleGenes, by = "row.names", all.x = TRUE)

    row.names(dat_merge) = dat_merge$Row.names
    dat_merge = dat_merge[, -1]

    dat_subset = dat_merge

    dat_subset$Accession = rownames(dat_subset)

    colnames(dat_subset) = c("FC", "pvalue", "Treatment", "Gene", "Accession")

    #dat_cbind = dat_cbind[order(dat_cbind$Module), ]

    #head(dat_cbind)

    assign(x = paste("dat_dotplotIMPUTED", j, sep = "_"), value = dat_subset)
  }, error = function(e) {
    cat("ERROR:", conditionMessage(e), i, j, "\n")
  })
}

#rbind treatment data to get long format
dat_rbind = do.call(rbind, lapply(ls(pattern = "dat_dotplotIMPUTED_"),
                                  function(x) {
                                    get_dat = get(x)
                                    get_dat
                                  }))
~~~~

```
dat_rbind$Gene = as.character(dat_rbind$Gene)

#change treatment to see comparison mean
dat_to_plot$Treatment = paste(dat_to_plot$Treatment, " vs ", controlsample)


#use data set for plot
dat_to_plot = dat_rbind

#calculate min and max in data set
FC_min = abs(min(dat_to_plot$FC, na.rm = TRUE))
FC_max = abs(max(dat_to_plot$FC, na.rm = TRUE))

#define limits based on min and max
FC_limit = ceiling(max(FC_min, FC_max))

#determine p-value limits
maxp = ceiling(ifelse(-log10(min(
  dat_to_plot$pvalue, na.rm = TRUE
)) <= 5, 5,-log10(min(
  dat_to_plot$pvalue, na.rm = TRUE
))))

#calculate -Log10(p-value)
dat_to_plot$Log10pvalue = -log10(dat_to_plot$pvalue)

#define confidence intervals
conf_up = quantile(dat_to_plot$FC, set_conf, na.rm = TRUE)
conf_down = quantile(dat_to_plot$FC, 1-set_conf, na.rm = TRUE)
conf_pvalue = quantile(dat_to_plot$Log10pvalue, set_conf, na.rm = TRUE)

#add a column containing colors
dat_to_plot$Color = "black"

#add a column containing labels
dat_to_plot$Label = NA
dat_to_plot$GeneLabel = NA

#for all rows with significant entries change color and label
for (k in 1:dim(dat_to_plot)[1]) {
  tryCatch({
    if (dat_to_plot$FC[k] < 0 &
        dat_to_plot$Log10pvalue[k] > 1.3)
      dat_to_plot$Color[k] = color_down
    if (dat_to_plot$FC[k] < conf_down &
        dat_to_plot$Log10pvalue[k] > 1.3){
      dat_to_plot$Label[k] = dat_to_plot$Accession[k]
      dat_to_plot$GeneLabel[k] = dat_to_plot$Gene[k]
    }
    if (dat_to_plot$FC[k] > 0 &
        dat_to_plot$Log10pvalue[k] > 1.3)
      dat_to_plot$Color[k] = color_up
    if (dat_to_plot$FC[k] > conf_up &
        dat_to_plot$Log10pvalue[k] > 1.3){
      dat_to_plot$Label[k] = dat_to_plot$Accession[k]
      dat_to_plot$GeneLabel[k] = dat_to_plot$Gene[k]
    }
    if (dat_to_plot$Log10pvalue[k] > conf_pvalue){
      dat_to_plot$Label[k] = dat_to_plot$Accession[k]
      dat_to_plot$GeneLabel[k] = dat_to_plot$Gene[k]
    }
  }, error = function(e) {
    cat("ERROR:", conditionMessage(e), k, "\n")
  })
}

#change treatment to see comparison mean
dat_to_plot$Treatment = paste(dat_to_plot$Treatment, " vs ", controlsample)
```

```
#use data for plotting
get_dat = dat_to_plot

#generate wrapped volcano plot as metafile
filetitle = paste("Volcano_Wrap_Imputed_", pvalue_decision, ".wmf", sep = "")
plotfile = paste(plots_dir, filetitle, sep = dirsep)
win.metafile(
  file = plotfile,
  width =
length(unique(get_dat$Treatment))/ceiling(length(unique(get_dat$Treatment)) / 3) *
3 + 0.5,
  height = ceiling(length(unique(get_dat$Treatment)) / 3) * 3 + 0.5,
  pointsize = 12,
  family = define_font
)

gp = ggplot(get_dat, aes(
  x = FC,
  y = Log10pvalue,
  #label = Label,
  colour = Color
)) +
  scale_color_identity() +
  geom_point(size = 1) +
  #geom_hline(yintercept = 1.3, linetype = "dashed", color = "red") +
  scale_y_continuous(
    limits = c(0, maxp),
    expand = c(0, 0),
    breaks = c(0, maxp / 2, maxp)
  ) +
  scale_x_continuous(limits = c(-FC_limit, FC_limit),
                     expand = c(0, 0)) +
  # geom_text(
  #   aes(label = Label),
  #   hjust = 0,
  #   vjust = 0.5,
  #   size = rel(2)
  # ) +
  theme_classic() +
  theme(
    axis.title.y = element_text(
      size = rel(1),
      face = "plain",
      color = "black"
    ),
    axis.title.x = element_text(
      size = rel(1),
      face = "plain",
      color = "black"
    ),
    axis.text.x = element_text(
      size = rel(1),
      color = "black",
      face = "plain",
      angle = 0,
      vjust = 1,
      hjust = 0.5
    ),
    axis.text.y = element_text(
      size = rel(1),
      color = "black",
      hjust = 1,
      face = "plain"
    ),
    axis.line = element_line(colour = "black", size = 0.5),
    axis.ticks = element_line(colour = "black", size = 0.5),
    legend.title = element_blank(),
    panel.spacing = unit(0.5, "cm"),
    #plot.margin = margin(1,1,1,1, "cm"),
    plot.title = element_text(
```

```
      size = rel(1.5),
      face = "bold",
      color = "black",
      hjust = 0.5
    )
  ) +
  #ggtitle(i) +
  ylab(paste("-Log10(", pvalue_decision, ")", sep = "")) +
  xlab("Log2(FC)") +
  facet_wrap( ~ Treatment, nrow = ceiling(length(unique(get_dat$Treatment)) / 3)) +
  theme(
    panel.background = element_rect(fill = NA, color = "black"),
    strip.text.x = element_text(
      size = rel(1.5),
      face = "bold",
      color = "black"
    ),
    strip.text.y = element_text(
      size = rel(1.5),
      face = "bold",
      color = "black"
    ),
    strip.background = element_rect(colour = "white", fill = "grey")
  )

print(gp)
dev.off()

#generate wrapped volcano plot as pdf - don't define family
filetitle = paste("Volcano_Wrap_Imputed_", pvalue_decision, ".pdf", sep = "")
plotfile = paste(plots_dir, filetitle, sep = dirsep)
pdf(
  file = plotfile,
  width =
length(unique(get_dat$Treatment))/ceiling(length(unique(get_dat$Treatment)) / 3) *
3 + 0.5,
  height = ceiling(length(unique(get_dat$Treatment)) / 3) * 3 + 0.5,
  pointsize = 12
)

print(gp)
dev.off()

#plot gene names
#generate wrapped volcano plot as metafile
filetitle = paste("Volcano_Wrap_Genes_Imputed_", pvalue_decision, ".wmf", sep = "")
plotfile = paste(plots_dir, filetitle, sep = dirsep)
win.metafile(
  file = plotfile,
  width =
length(unique(get_dat$Treatment))/ceiling(length(unique(get_dat$Treatment)) / 3) *
3 + 0.5,
  height = ceiling(length(unique(get_dat$Treatment)) / 3) * 3 + 0.5,
  pointsize = 12,
  family = define_font
)

gp = ggplot(get_dat, aes(
  x = FC,
  y = Log10pvalue,
  label = GeneLabel,
  colour = Color
)) +
  scale_color_identity() +
  geom_point(size = 1) +
  #geom_hline(yintercept = 1.3, linetype = "dashed", color = "red") +
  scale_y_continuous(
    limits = c(0, maxp),
    expand = c(0, 0),
    breaks = c(0, maxp / 2, maxp)
```

```
  ) +
  scale_x_continuous(limits = c(-FC_limit, FC_limit),
                     expand = c(0, 0)) +
  geom_text(
    aes(label = GeneLabel),
    hjust = "outward",
    vjust = 0.4,
    size = rel(2)
  ) +
  theme_classic() +
  theme(
    axis.title.y = element_text(
      size = rel(1),
      face = "plain",
      color = "black"
    ),
    axis.title.x = element_text(
      size = rel(1),
      face = "plain",
      color = "black"
    ),
    axis.text.x = element_text(
      size = rel(1),
      color = "black",
      face = "plain",
      angle = 0,
      vjust = 1,
      hjust = 0.5
    ),
    axis.text.y = element_text(
      size = rel(1),
      color = "black",
      hjust = 1,
      face = "plain"
    ),
    axis.line = element_line(colour = "black", size = 0.5),
    axis.ticks = element_line(colour = "black", size = 0.5),
    legend.title = element_blank(),
    panel.spacing = unit(0.5, "cm"),
    #plot.margin = margin(1,1,1,1, "cm"),
    plot.title = element_text(
      size = rel(1.5),
      face = "bold",
      color = "black",
      hjust = 0.5
    )
  ) +
  #ggtitle(i) +
  ylab(paste("-Log10(", pvalue_decision, ")", sep = "")) +
  xlab("Log2(FC)") +
  facet_wrap( ~ Treatment, nrow = ceiling(length(unique(get_dat$Treatment)) / 3)) +
  theme(
    panel.background = element_rect(fill = NA, color = "black"),
    strip.text.x = element_text(
      size = rel(1.5),
      face = "bold",
      color = "black"
    ),
    strip.text.y = element_text(
      size = rel(1.5),
      face = "bold",
      color = "black"
    ),
    strip.background = element_rect(colour = "white", fill = "grey")
  )

print(gp)
dev.off()

filetitle = paste("Volcano_Wrap_Genes_Imputed_", pvalue_decision, ".pdf", sep = "")
```

````
plotfile = paste(plots_dir, filetitle, sep = dirsep)
pdf(
  file = plotfile,
  width =
length(unique(get_dat$Treatment))/ceiling(length(unique(get_dat$Treatment)) / 3) *
3 + 0.5,
  height = ceiling(length(unique(get_dat$Treatment)) / 3) * 3 + 0.5,
  pointsize = 12
)

print(gp)
dev.off()
```

#### 4.2.2 Single volcano plots per treatment

```{r single volcano plots imputed}

for (i in unique(dat_to_plot$Treatment)){

  get_dat = dat_to_plot[dat_to_plot$Treatment == i, ]

  #generate wrapped volcano plot as metafile
filetitle = paste("Volcano_Wrap_Imputed_", pvalue_decision, "_", i, ".wmf", sep =
"")
plotfile = paste(plots_dir, filetitle, sep = dirsep)
win.metafile(
  file = plotfile,
  width = 3.5,
  height = 3.5,
  pointsize = 12,
  family = define_font
)

gp = ggplot(get_dat, aes(
  x = FC,
  y = Log10pvalue,
  #label = Label,
  colour = Color
)) +
  scale_color_identity() +
  geom_point(size = 1) +
  #geom_hline(yintercept = 1.3, linetype = "dashed", color = "red") +
  scale_y_continuous(
    limits = c(0, maxp),
    expand = c(0, 0),
    breaks = c(0, maxp / 2, maxp)
  ) +
  scale_x_continuous(limits = c(-FC_limit, FC_limit),
                     expand = c(0, 0)) +
  # geom_text(
  #   aes(label = Label),
  #   hjust = 0,
  #   vjust = 0.5,
  #   size = rel(2)
  # ) +
  theme_classic() +
  theme(
    axis.title.y = element_text(
      size = rel(1),
      face = "plain",
      color = "black"
    ),
    axis.title.x = element_text(
      size = rel(1),
      face = "plain",
      color = "black"
    ),
    axis.text.x = element_text(
      size = rel(1),
````

```
      color = "black",
      face = "plain",
      angle = 0,
      vjust = 1,
      hjust = 0.5
    ),
    axis.text.y = element_text(
      size = rel(1),
      color = "black",
      hjust = 1,
      face = "plain"
    ),
    axis.line = element_line(colour = "black", size = 0.5),
    axis.ticks = element_line(colour = "black", size = 0.5),
    legend.title = element_blank(),
    panel.spacing = unit(0.5, "cm"),
    #plot.margin = margin(1,1,1,1, "cm"),
    plot.title = element_text(
      size = rel(1.5),
      face = "bold",
      color = "black",
      hjust = 0.5
    )
  ) +
  #ggtitle(i) +
  ylab(paste("-Log10(", pvalue_decision, ")", sep = "")) +
  xlab("Log2(FC)") +
  facet_wrap( ~ Treatment, nrow = ceiling(length(unique(get_dat$Treatment)) / 3)) +
  theme(
    panel.background = element_rect(fill = NA, color = "black"),
    strip.text.x = element_text(
      size = rel(1.5),
      face = "bold",
      color = "black"
    ),
    strip.text.y = element_text(
      size = rel(1.5),
      face = "bold",
      color = "black"
    ),
    strip.background = element_rect(colour = "white", fill = "grey")
  )

print(gp)
dev.off()

#generate wrapped volcano plot as pdf - don't define family
filetitle = paste("Volcano_Wrap_Imputed_", pvalue_decision, "_", i, ".pdf", sep =
"")
plotfile = paste(plots_dir, filetitle, sep = dirsep)
pdf(
  file = plotfile,
  width = 3.5,
  height = 3.5,
  pointsize = 12
)

print(gp)
dev.off()

#plot gene names
#generate wrapped volcano plot as metafile
filetitle = paste("Volcano_Wrap_Imputed_Genes_", pvalue_decision, "_", i, ".wmf",
sep = "")
plotfile = paste(plots_dir, filetitle, sep = dirsep)
win.metafile(
  file = plotfile,
  width = 3.5,
  height = 3.5,
  pointsize = 12,
```

```
  family = define_font
)

gp = ggplot(get_dat, aes(
  x = FC,
  y = Log10pvalue,
  label = GeneLabel,
  colour = Color
)) +
  scale_color_identity() +
  geom_point(size = 1) +
  #geom_hline(yintercept = 1.3, linetype = "dashed", color = "red") +
  scale_y_continuous(
    limits = c(0, maxp),
    expand = c(0, 0),
    breaks = c(0, maxp / 2, maxp)
  ) +
  scale_x_continuous(limits = c(-FC_limit, FC_limit),
                     expand = c(0, 0)) +
  geom_text(
    aes(label = GeneLabel),
    hjust = "outward",
    vjust = 0.4,
    size = rel(2)
  ) +
  theme_classic() +
  theme(
    axis.title.y = element_text(
      size = rel(1),
      face = "plain",
      color = "black"
    ),
    axis.title.x = element_text(
      size = rel(1),
      face = "plain",
      color = "black"
    ),
    axis.text.x = element_text(
      size = rel(1),
      color = "black",
      face = "plain",
      angle = 0,
      vjust = 1,
      hjust = 0.5
    ),
    axis.text.y = element_text(
      size = rel(1),
      color = "black",
      hjust = 1,
      face = "plain"
    ),
    axis.line = element_line(colour = "black", size = 0.5),
    axis.ticks = element_line(colour = "black", size = 0.5),
    legend.title = element_blank(),
    panel.spacing = unit(0.5, "cm"),
    #plot.margin = margin(1,1,1,1, "cm"),
    plot.title = element_text(
      size = rel(1.5),
      face = "bold",
      color = "black",
      hjust = 0.5
    )
  ) +
  #ggtitle(i) +
  ylab(paste("-Log10(", pvalue_decision, ")", sep = "")) +
  xlab("Log2(FC)") +
  facet_wrap( ~ Treatment, nrow = ceiling(length(unique(get_dat$Treatment)) / 3)) +
  theme(
    panel.background = element_rect(fill = NA, color = "black"),
    strip.text.x = element_text(
```

````
      size = rel(1.5),
      face = "bold",
      color = "black"
    ),
    strip.text.y = element_text(
      size = rel(1.5),
      face = "bold",
      color = "black"
    ),
    strip.background = element_rect(colour = "white", fill = "grey")
  )

print(gp)
dev.off()

filetitle = paste("Volcano_Wrap_Imputed_Genes_", pvalue_decision, "_", i, ".pdf",
sep = "")
plotfile = paste(plots_dir, filetitle, sep = dirsep)
pdf(
  file = plotfile,
  width = 3.5,
  height = 3.5,
  pointsize = 12
)

print(gp)

dev.off()
}
```

### 4.3 Volcano Facets zscores

```{r volcano facets zscores imputed data}
#choose data set
sample_dat_FC_pvalue = summary_zscore

#exclude control because it contains only 1 and 0 values
sample_dat_FC_pvalue = sample_dat_FC_pvalue[,
which(grepl(colnames(sample_dat_FC_pvalue), pattern = controlsample) == FALSE)]

#create sample vector
sample_vector = unique(gsub(
  colnames(sample_dat_FC_pvalue),
  pattern = "_pvalue",
  replacement = ""
))

#bring data to long format, which is necessary for ggplot
for (j in sample_vector) {
  tryCatch({
    dat_subset = as.data.frame(sample_dat_FC_pvalue[, grepl(j,

colnames(sample_dat_FC_pvalue))])
    dat_subset$Treatment = j

    dat_subset$Accession = rownames(dat_subset)

    colnames(dat_subset) = c("FC", "pvalue", "Treatment", "Accession")

    #dat_cbind = dat_cbind[order(dat_cbind$Module), ]

    #head(dat_cbind)

    dat_subset = merge(dat_subset, sampleGenes, by = "row.names")

    rownames(dat_subset) = dat_subset$Row.names

    dat_subset = dat_subset[, -1]
````

```
    assign(x = paste("dat_dotplotIMPUTED", j, sep = "_"), value = dat_subset)
  }, error = function(e) {
    cat("ERROR:", conditionMessage(e), i, j, "\n")
  })
}

#rbind treatment data to get long format
dat_rbind = do.call(rbind, lapply(ls(pattern = "dat_dotplotIMPUTED_"),
                                  function(x) {
                                    get_dat = get(x)
                                    get_dat
                                  }))

#use data set for plot
dat_to_plot = dat_rbind

#gene data as character
dat_to_plot$Gene = as.character(dat_to_plot$Gene)

#calculate min and max in data set
FC_min = abs(min(dat_to_plot$FC, na.rm = TRUE))
FC_max = abs(max(dat_to_plot$FC, na.rm = TRUE))

#define limits based on min and max
FC_limit = ceiling(max(FC_min, FC_max))

#determine p-value limits
maxp = ceiling(ifelse(-log10(min(
  dat_to_plot$pvalue, na.rm = TRUE
)) <= 5, 5,-log10(min(
  dat_to_plot$pvalue, na.rm = TRUE
))))

#calculate -Log10(p-value)
dat_to_plot$Log10pvalue = -log10(dat_to_plot$pvalue)

#define confidence intervals
conf_up = quantile(dat_to_plot$FC, set_conf, na.rm = TRUE)
conf_down = quantile(dat_to_plot$FC, 1-set_conf, na.rm = TRUE)
conf_pvalue = quantile(dat_to_plot$Log10pvalue, set_conf, na.rm = TRUE)

#add a column containing colors
dat_to_plot$Color = "black"

#add a column containing labels
dat_to_plot$Label = NA

#for all rows with significant entries change color and label
for (k in 1:dim(dat_to_plot)[1]) {
  tryCatch({
    if (dat_to_plot$FC[k] < 0 &
        dat_to_plot$Log10pvalue[k] > 1.3) #1.9 is 95 % interval
      dat_to_plot$Color[k] = color_down
    if (dat_to_plot$FC[k] <= conf_down &
        dat_to_plot$Log10pvalue[k] > 1.3) #2.57 is 99 % interval
      dat_to_plot$Label[k] = dat_to_plot$Gene[k]
    if (dat_to_plot$FC[k] > 0 &
        dat_to_plot$Log10pvalue[k] > 1.3)
      dat_to_plot$Color[k] = color_up
    if (dat_to_plot$FC[k] >= conf_up &
        dat_to_plot$Log10pvalue[k] > 1.3)
      dat_to_plot$Label[k] = dat_to_plot$Gene[k]
    if (dat_to_plot$Log10pvalue[k] > conf_pvalue)
      dat_to_plot$Label[k] = dat_to_plot$Gene[k]
  }, error = function(e) {
    cat("ERROR:", conditionMessage(e), k, "\n")
  })
}

#use data for plotting
```

```
get_dat = dat_to_plot

#generate wrapped volcano plot as metafile
filetitle = paste("Volcano_Wrap_zscore_imputed_", pvalue_decision, ".wmf", sep =
"")
plotfile = paste(plots_dir, filetitle, sep = dirsep)
win.metafile(
  file = plotfile,
  width =
length(unique(get_dat$Treatment))/ceiling(length(unique(get_dat$Treatment)) / 3) *
3 + 0.5,
  height = ceiling(length(unique(get_dat$Treatment)) / 3) * 3 + 0.5,
  pointsize = 12,
  family = define_font
)

gp = ggplot(get_dat, aes(
  x = FC,
  y = Log10pvalue,
  label = Label,
  colour = Color
)) +
  scale_color_identity() +
  geom_point(size = 1) +
  #geom_hline(yintercept = 1.3, linetype = "dashed", color = "red") +
  scale_y_continuous(
    limits = c(0, maxp),
    expand = c(0, 0),
    breaks = c(0, maxp / 2, maxp)
  ) +
  scale_x_continuous(limits = c(-FC_limit, FC_limit),
                     expand = c(0, 0)) +
  geom_text(
    aes(label = Label),
    hjust = "outward",
    vjust = 0.4,
    size = rel(2)
  ) +
  theme_classic() +
  theme(
    axis.title.y = element_text(
      size = rel(1),
      face = "plain",
      color = "black"
    ),
    axis.title.x = element_text(
      size = rel(1),
      face = "plain",
      color = "black"
    ),
    axis.text.x = element_text(
      size = rel(1),
      color = "black",
      face = "plain",
      angle = 0,
      vjust = 1,
      hjust = 0.5
    ),
    axis.text.y = element_text(
      size = rel(1),
      color = "black",
      hjust = 1,
      face = "plain"
    ),
    axis.line = element_line(colour = "black", size = 0.5),
    axis.ticks = element_line(colour = "black", size = 0.5),
    legend.title = element_blank(),
    panel.spacing = unit(0.5, "cm"),
    #plot.margin = margin(1,1,1,1, "cm"),
    plot.title = element_text(
```

```
      size = rel(1.5),
      face = "bold",
      color = "black",
      hjust = 0.5
    )
  ) +
  #ggtitle(i) +
  ylab(paste("-Log10(", pvalue_decision, ")", sep = "")) +
  xlab("z-score") +
  facet_wrap( ~ Treatment, nrow = ceiling(length(unique(get_dat$Treatment)) / 3)) +
  theme(
    panel.background = element_rect(fill = NA, color = "black"),
    strip.text.x = element_text(
      size = rel(1.5),
      face = "bold",
      color = "black"
    ),
    strip.text.y = element_text(
      size = rel(1.5),
      face = "bold",
      color = "black"
    ),
    strip.background = element_rect(colour = "white", fill = "grey")
  )

print(gp)
dev.off()

#generate wrapped volcano plot as pdf - don't define family
filetitle = paste("Volcano_Wrap_zscore_imputed_", pvalue_decision, ".pdf", sep =
"")
plotfile = paste(plots_dir, filetitle, sep = dirsep)
pdf(
  file = plotfile,
  width =
length(unique(get_dat$Treatment))/ceiling(length(unique(get_dat$Treatment)) / 3) *
3 + 0.5,
  height = ceiling(length(unique(get_dat$Treatment)) / 3) * 3 + 0.5,
  pointsize = 12
)

print(gp)
dev.off()

filetitle = paste("Volcano_Wrap_zscore_imputed_", pvalue_decision, ".svg", sep =
"")
plotfile = paste(plots_dir, filetitle, sep = dirsep)
svg(
  file = plotfile,
  width =
length(unique(get_dat$Treatment))/ceiling(length(unique(get_dat$Treatment)) / 3) *
3 + 0.5,
  height = ceiling(length(unique(get_dat$Treatment)) / 3) * 3 + 0.5,
  family = define_font,
  pointsize = 12
)

print(gp)
dev.off()

filetitle = paste("Volcano_Wrap_zscore_imputed_", pvalue_decision, ".png", sep =
"")
plotfile = paste(plots_dir, filetitle, sep = dirsep)
png(
  file = plotfile,
  units = "in",
  res = 800,
  width =
length(unique(get_dat$Treatment))/ceiling(length(unique(get_dat$Treatment)) / 3) *
3 + 0.5,
```

```
  height = ceiling(length(unique(get_dat$Treatment)) / 3) * 3 + 0.5,
  family = define_font,
  pointsize = 12
)

print(gp)
dev.off()
```

4.4 Heatmaps

```{r heatmap FCs imputed}
#define clustering method
distance_measure = "euclidean" #default: "euclidean"
clustering_method = "complete" #default: "complete"

  dat_to_scale = summary_IPA
  dat_to_scale = dat_to_scale[, which(grepl(colnames(dat_to_scale), pattern =
"_pvalue") == FALSE)]

  #remove all rows that contain only NA
  dat_to_scale[dat_to_scale == "NaN"] = NA
  dat_to_scale = dat_to_scale[rowSums(is.na(dat_to_scale)) != ncol(dat_to_scale),]

  #remove control sample because it contains only values of 1
  dat_to_scale = dat_to_scale[, which(grepl(colnames(dat_to_scale),
                                            pattern = controlsample) == FALSE)]

  boxplot(dat_to_scale, las = 2)

  dat_to_scale = dat_to_scale[rowSums(is.na(dat_to_scale)) < ncol(dat_to_scale), ]

  merge_dat = dat_to_scale

  #replace NA by 0 to allow for row clustering
  dat_to_scale[is.na(dat_to_scale)] = 0

  boxplot(dat_to_scale, las = 2)

  min = min(dat_to_scale, na.rm = TRUE)
  max = max(dat_to_scale, na.rm = TRUE)

  limits = ceiling(max(abs(min), abs(max)))/2

  # create empty vector that will be filled with median values
  median_abundance_vector = vector(mode = "expression", length =
length(colnames(dat_to_scale)))
  for (j in 1:length(colnames(merge_dat))){
    M = na.omit(merge_dat[,j])
    median_abundance_vector[j] = median(M)
  }
  head(median_abundance_vector)

  mid = round(median(unlist(lapply(median_abundance_vector, as.numeric))), digits =
0)
  min = -limits
  max = limits

  dend_rows = dendsort(hclust(dist(dat_to_scale))) #can not be used with NAs
  dend_cols = dendsort(hclust(dist(t(dat_to_scale))))
  dend_cols = color_branches(dend_cols, k = floor(dim(dat_to_scale)[2]/2))

  plot_file = paste("Heatmap_FCs_imputed", ".pdf", sep = "")
  plot_path = paste(plots_dir, plot_file, sep = dirsep)
  pdf(file = plot_path,
      width = dim(merge_dat)[2],
      height = 8)

  h = Heatmap(
```

```
    merge_dat,
    col = colorRamp2(c(min, mid, max), c(color_down, "white", color_up)),
    color_space = "sRGB",
    na_col = "grey",
    cluster_rows = dend_rows,
    cluster_columns = dend_cols,
    clustering_distance_columns = distance_measure,
    clustering_distance_rows = distance_measure,
    clustering_method_columns = clustering_method,
    clustering_method_rows = clustering_method,
    column_dend_side = "top",
    column_dend_height = unit(3, "cm"),
    column_names_side = "top",
    show_row_names = "FALSE",
    #row_names_side = "left",
    #row_names_max_width = unit(15, "cm"),
    column_names_max_height = unit(6, "cm"),
    show_row_dend = TRUE,
    #row_title_gp = gpar(cex = 0.6),
    column_names_gp = gpar(cex = 1, fontface = "bold"),
    #column_dend_reorder=c(1,3,4,5,2),
    #row_dend_reorder=F,
    heatmap_legend_param = list(
      title = "Log2(FC)",
      title_gp = gpar(cex = 1, fontface = "bold"),
      color_bar = c("continuous")
    ),
    show_heatmap_legend = TRUE
  )

  print(h)

  dev.off()

  #plot with discrete breaks
discrete_sequence = seq(from = min, to = max, by = 0.5)
discrete_colors = colorRampPalette(colors = c(color_down, "white",
                                             color_up))(length(discrete_sequence))

  plot_file = paste("Heatmap_discrete_FCs_imputed", ".pdf", sep = "")
  plot_path = paste(plots_dir, plot_file, sep = dirsep)
  pdf(file = plot_path,
      width = dim(merge_dat)[2],
      height = 8)

  h = Heatmap(
  merge_dat,
  col = colorRamp2(breaks = discrete_sequence, colors = discrete_colors),
  color_space = "sRGB",
  na_col = "grey",
  cluster_rows = dend_rows,
  cluster_columns = dend_cols,
  column_dend_side = "top",
  column_dend_height = unit(3, "cm"),
  column_names_side = "top",
  show_row_names = "FALSE",
  #row_names_side = "left",
  #row_names_max_width = unit(15, "cm"),
  column_names_max_height = unit(6, "cm"),
  show_row_dend = TRUE,
  row_dend_width = unit(2, "cm"),
  #row_title_gp = gpar(cex = 0.6),
  column_names_gp = gpar(cex = 1, fontface = "bold"),
  #column_dend_reorder=c(3,2,1,7,4,5,6),
  #row_dend_reorder=F,
  heatmap_legend_param = list(
    title = "Log2(FC)",
    at = seq(from = min, to = max, by = max/2),
    title_gp = gpar(cex = 1, fontface = "bold"),
    color_bar = c("discrete")
```

```
  ),
  show_heatmap_legend = TRUE
)

print(h)

dev.off()

plot_file = paste("Heatmap_discrete_FCs_imputed", ".wmf", sep = "")
  plot_path = paste(plots_dir, plot_file, sep = dirsep)
  win.metafile(file = plot_path,
      width = dim(merge_dat)[2],
      family = define_font,
      height = 8)

  print(h)

dev.off()

plot_file = paste("Heatmap_discrete_FCs_imputed", ".svg", sep = "")
  plot_path = paste(plots_dir, plot_file, sep = dirsep)
 svg(file = plot_path,
      width = dim(merge_dat)[2],
      family = define_font,
      height = 8)

  print(h)

dev.off()

plot_file = paste("Heatmap_discrete_FCs_imputed", ".png", sep = "")
  plot_path = paste(plots_dir, plot_file, sep = dirsep)
 png(file = plot_path,
     units = "in",
     res = 800,
      width = dim(merge_dat)[2],
      family = define_font,
      height = 8)

  print(h)

dev.off()
```

```{r heatmap abundances imputed data}
#plot abundances of means
#extract abundance means
heatmap_abundances_df = as.data.frame(do.call(cbind,
                                              lapply(ls(pattern =
"Calculated_ReplicatesIMPUTED_reliable_"),
                                                            function(x) {
                                                              get_dat = get(x)
                                                              get_dat =
as.vector(get_dat[, grepl(colnames(get_dat),

pattern = "_Mean$")])
                                                            })))

heatmap_abundances_colnames = as.vector(ls(pattern =
"Calculated_ReplicatesIMPUTED_reliable_"))
heatmap_abundances_colnames = gsub(heatmap_abundances_colnames,
                                   pattern =
"Calculated_ReplicatesIMPUTED_reliable_",
                                   replacement = "")
colnames(heatmap_abundances_df) = heatmap_abundances_colnames

heatmap_abundances_df[heatmap_abundances_df == "NaN"] = NA

#define limits
max_heatmap = max(heatmap_abundances_df, na.rm = TRUE)*0.9
```

```
max = ceiling(max_heatmap)

# create empty vector that will be filled with median values
median_abundance_vector = vector(mode = "expression",
                                 length = length(colnames(heatmap_abundances_df)))

for (j in 1:length(colnames(heatmap_abundances_df))){
  M = na.omit(heatmap_abundances_df[,j])
  median_abundance_vector[j] = median(M)
}
head(median_abundance_vector)

mid = round(median(unlist(lapply(median_abundance_vector, as.numeric))), digits =
0)

min = mid - (max-mid)

heatmap_abundances_df = heatmap_abundances_df[rowSums(is.na(heatmap_abundances_df))
<
                                                ncol(heatmap_abundances_df), ]

dat_to_plot = heatmap_abundances_df

#replace NAs by 0
heatmap_abundances_df[is.na(heatmap_abundances_df)] = 0

dend_rows = dendsort(hclust(dist(heatmap_abundances_df))) #can't be used with NAs
dend_cols = dendsort(hclust(dist(t(heatmap_abundances_df))))
dend_cols = color_branches(dend_cols, k = floor(dim(heatmap_abundances_df)[2] / 2))

plot_file = "Heatmap_AbundanceMeans_Imputed.pdf"
plot_path = paste(plots_dir, plot_file, sep = dirsep)
pdf(file = plot_path,
    width = dim(dat_to_plot)[2] + 2,
    height = 8)

h = Heatmap(
  dat_to_plot,
  col = colorRamp2(c(min, mid, max), c(color_low, color_mid, color_high)),
  color_space = "sRGB",
  na_col = "grey",
  cluster_rows = dend_rows,
  cluster_columns = dend_cols,
  column_dend_side = "top",
  column_dend_height = unit(3, "cm"),
  column_names_side = "top",
  show_row_names = "FALSE",
  #row_names_side = "left",
  #row_names_max_width = unit(15, "cm"),
  column_names_max_height = unit(6, "cm"),
  show_row_dend = TRUE,
  #row_title_gp = gpar(cex = 0.6),
  column_names_gp = gpar(cex = 1, fontface = "bold"),
  #column_dend_reorder=c(1,3,4,5,2),
  #row_dend_reorder=F,
  heatmap_legend_param = list(
    title = "Log2(Abundance)",
    title_gp = gpar(cex = 1, fontface = "bold"),
    at = c(min, mid, max),
    labels = c(min, mid, max),
    color_bar = c("continuous")
  ),
  show_heatmap_legend = TRUE
)

print(h)

dev.off()

#plot with discrete breaks
```

```
discrete_sequence = seq(from = min, to = max, by = 1)
discrete_colors = colorRampPalette(colors = c(color_low, color_mid,

color_high))(length(discrete_sequence))

plot_file = "Heatmap_AbundanceMeans_Imputed_discrete.pdf"
plot_path = paste(plots_dir, plot_file, sep = dirsep)
pdf(file = plot_path,
    width = dim(dat_to_plot)[2] + 2,
    height = 8)

h = Heatmap(
  dat_to_plot,
  col = colorRamp2(breaks = discrete_sequence, colors = discrete_colors),
  color_space = "sRGB",
  na_col = "grey",
  cluster_rows = dend_rows,
  cluster_columns = dend_cols,
  column_dend_side = "top",
  column_dend_height = unit(3, "cm"),
  column_names_side = "top",
  show_row_names = "FALSE",
  #row_names_side = "left",
  #row_names_max_width = unit(15, "cm"),
  column_names_max_height = unit(6, "cm"),
  show_row_dend = TRUE,
  row_dend_width = unit(2, "cm"),
  #row_title_gp = gpar(cex = 0.6),
  column_names_gp = gpar(cex = 1, fontface = "bold"),
  #column_dend_reorder=c(3,2,1,7,4,5,6),
  #row_dend_reorder=F,
  heatmap_legend_param = list(
    title = "Log2(Abundance)",
    at = seq(from = min, to = max, by = 2),
    title_gp = gpar(cex = 1, fontface = "bold"),
    color_bar = c("discrete")
  ),
  show_heatmap_legend = TRUE
)

print(h)

dev.off()

plot_file = "Heatmap_AbundanceMeans_Imputed_discrete.wmf"
plot_path = paste(plots_dir, plot_file, sep = dirsep)
win.metafile(file = plot_path,
    width = dim(dat_to_plot)[2] + 2,
    family = define_font,
    height = 8)

print(h)

dev.off()

plot_file = "Heatmap_AbundanceMeans_Imputed_discrete.svg"
plot_path = paste(plots_dir, plot_file, sep = dirsep)
svg(file = plot_path,
    width = dim(dat_to_plot)[2] + 2,
    family = define_font,
    height = 8)

print(h)

dev.off()

plot_file = "Heatmap_AbundanceMeans_Imputed_discrete.png"
plot_path = paste(plots_dir, plot_file, sep = dirsep)
png(file = plot_path,
    units = "in",
```

````
    res = 800,
    width = dim(dat_to_plot)[2] + 2,
    family = define_font,
    height = 8)

print(h)

dev.off()
```

```{r Pheatmap abundance replicates imputed data}
dat_to_plot = dat_to_calculate

dat_pvalues = summary_IPA[, which(grepl(colnames(summary_IPA), pattern =
"_pvalue"))]
dat_pvalues = dat_pvalues[apply(dat_pvalues <= 0.05, 1, any), ]
dat_pvalues = dat_pvalues[rowSums(is.na(dat_pvalues)) < ncol(dat_pvalues), ]

get_DE = as.data.frame(rownames(dat_pvalues))
colnames(get_DE) = c("Row.names")

sampleTable_DE = merge(get_DE, dat_to_plot, by.x = "Row.names", by.y = "row.names")
rownames(sampleTable_DE) = sampleTable_DE$Row.names
sampleTable_DE = sampleTable_DE[, -1]

dat_to_plot = as.matrix(sampleTable_DE)

discrete_colors = colorRampPalette(colors = c(color_low, color_mid,

color_high))(length(discrete_sequence))

filename = "Pheatmap_Replicates_Imputed_Rowscale_DE.pdf"
plotfile = paste(plots_dir, filename, sep = dirsep)

pdf(file = plotfile,
    width = 5,
    height = 5)
pheatmap(dat_to_plot,
         color = discrete_colors,
         cluster_rows = TRUE,
         show_rownames = FALSE,
         treeheight_row = 0,
         cluster_cols = FALSE,
         show_colnames = TRUE,
         treeheight_col = 0,
         scale = "row")
dev.off()

filename = "Pheatmap_Replicates_Imputed_Rowscale_DE.wmf"
plotfile = paste(plots_dir, filename, sep = dirsep)

win.metafile(file = plotfile,
    width = 5,
    height = 5,
    family = define_font)
pheatmap(dat_to_plot,
         color = discrete_colors,
         cluster_rows = TRUE,
         show_rownames = FALSE,
         treeheight_row = 0,
         cluster_cols = FALSE,
         show_colnames = TRUE,
         treeheight_col = 0,
         scale = "row")
dev.off()
```



```{r heatmap z-scores imputed data}
````

```
  dat_to_scale = summary_zscore

  #remove control sample because it contains only values of 1
  dat_to_scale = dat_to_scale[, which(grepl(colnames(dat_to_scale),
                                           pattern = controlsample) == FALSE)]

  dat_to_scale = dat_to_scale[, which(grepl(colnames(dat_to_scale),
                                           pattern = "_pvalue") == FALSE)]

  dat_to_scale = dat_to_scale[!apply(dat_to_scale, 1, function(x) all(is.na(x))), ]

  dat_to_scale = dat_to_scale[rowSums(is.na(dat_to_scale)) < ncol(dat_to_scale), ]

  merge_dat = dat_to_scale

  #replace NA by 0 to allow for row clustering
  dat_to_scale[is.na(dat_to_scale)] = 0

  boxplot(dat_to_scale, las = 2)

  min = min(dat_to_scale, na.rm = TRUE)
  max = max(dat_to_scale, na.rm = TRUE)

  limits = floor(max(abs(min), abs(max)))/4

  # create empty vector that will be filled with median values
  median_abundance_vector = vector(mode = "expression", length =
length(colnames(dat_to_scale)))
  for (j in 1:length(colnames(merge_dat))){
    M = na.omit(merge_dat[,j])
    median_abundance_vector[j] = median(M)
  }
  head(median_abundance_vector)

  mid = round(median(unlist(lapply(median_abundance_vector, as.numeric))), digits =
0)
  min = -limits
  max = limits

  dend_rows = dendsort(hclust(dist(dat_to_scale))) #can not be used with NAs
  dend_cols = dendsort(hclust(dist(t(dat_to_scale))))
  dend_cols = color_branches(dend_cols, k = floor(dim(dat_to_scale)[2]/2))

  plot_file = "Heatmap_zscores_imputed.pdf"
  plot_path = paste(plots_dir, plot_file, sep = dirsep)
  pdf(file = plot_path,
      width = dim(merge_dat)[2],
      height = 8)

  h = Heatmap(
    merge_dat,
    col = colorRamp2(c(min, mid, max), c(color_down, "white", color_up)),
    color_space = "sRGB",
    na_col = "grey",
    cluster_rows = dend_rows,
    cluster_columns = dend_cols,
    clustering_distance_columns = distance_measure,
    clustering_distance_rows = distance_measure,
    clustering_method_columns = clustering_method,
    clustering_method_rows = clustering_method,
    column_dend_side = "top",
    column_dend_height = unit(3, "cm"),
    column_names_side = "top",
    show_row_names = "FALSE",
    #row_names_side = "left",
    #row_names_max_width = unit(15, "cm"),
    column_names_max_height = unit(6, "cm"),
    show_row_dend = TRUE,
    #row_title_gp = gpar(cex = 0.6),
    column_names_gp = gpar(cex = 1, fontface = "bold"),
```

```
    #column_dend_reorder=c(1,3,4,5,2),
    #row_dend_reorder=F,
    heatmap_legend_param = list(
      title = "z-score",
      title_gp = gpar(cex = 1, fontface = "bold"),
      color_bar = c("continuous")
    ),
    show_heatmap_legend = TRUE
  )

  print(h)

  dev.off()

  #plot with discrete breaks
discrete_sequence = seq(from = min, to = max, by = 0.5)
discrete_colors = colorRampPalette(colors = c(color_down, "white",
                                              color_up))(length(discrete_sequence))

  plot_file = "Heatmap_zscores_imputed_discrete.pdf"
  plot_path = paste(plots_dir, plot_file, sep = dirsep)
  pdf(file = plot_path,
      width = dim(merge_dat)[2],
      height = 8)

  h = Heatmap(
  merge_dat,
  col = colorRamp2(breaks = discrete_sequence, colors = discrete_colors),
  color_space = "sRGB",
  na_col = "grey",
  cluster_rows = dend_rows,
  cluster_columns = dend_cols,
  column_dend_side = "top",
  column_dend_height = unit(3, "cm"),
  column_names_side = "top",
  show_row_names = "FALSE",
  #row_names_side = "left",
  #row_names_max_width = unit(15, "cm"),
  column_names_max_height = unit(6, "cm"),
  show_row_dend = TRUE,
  row_dend_width = unit(2, "cm"),
  #row_title_gp = gpar(cex = 0.6),
  column_names_gp = gpar(cex = 1, fontface = "bold"),
  #column_dend_reorder=c(3,2,1,7,4,5,6),
  #row_dend_reorder=F,
  heatmap_legend_param = list(
    title = "z-score",
    at = seq(from = min, to = max, by = max/2),
    title_gp = gpar(cex = 1, fontface = "bold"),
    color_bar = c("discrete")
  ),
  show_heatmap_legend = TRUE
)

print(h)

dev.off()

plot_file = "Heatmap_zscores_imputed_discrete.wmf"
  plot_path = paste(plots_dir, plot_file, sep = dirsep)
  win.metafile(file = plot_path,
      width = dim(merge_dat)[2],
      family = define_font,
      height = 8)

  print(h)

dev.off()

plot_file = "Heatmap_zscores_imputed_discrete.svg"
```

```
  plot_path = paste(plots_dir, plot_file, sep = dirsep)
  svg(file = plot_path,
      width = dim(merge_dat)[2],
      family = define_font,
      height = 8)

  print(h)

dev.off()

plot_file = "Heatmap_zscores_imputed_discrete.png"
  plot_path = paste(plots_dir, plot_file, sep = dirsep)
  png(file = plot_path,
      units = "in",
      res = 800,
      width = dim(merge_dat)[2],
      family = define_font,
      height = 8)

  print(h)

dev.off()
```

```{r heatmap DE z-scores imputed data}

  dat_to_scale = summary_zscore

  #remove control sample because it contains only values of 1
  dat_to_scale = dat_to_scale[, which(grepl(colnames(dat_to_scale),
                                            pattern = controlsample) == FALSE)]

  dat_pvalues = dat_to_scale[, which(grepl(colnames(dat_to_scale),
                                            pattern = "_pvalue") == TRUE)]

  dat_to_scale = dat_to_scale[, which(grepl(colnames(dat_to_scale),
                                            pattern = "_pvalue") == FALSE)]

  dat_pvalues[dat_pvalues > 0.05] = NA
  dat_to_scale = dat_to_scale[!apply(dat_pvalues, 1, function(x) all(is.na(x))), ]

  dat_to_scale = dat_to_scale[!apply(dat_to_scale, 1, function(x) all(is.na(x))), ]

  merge_dat = dat_to_scale

  #replace NA by 0 to allow for row clustering
  dat_to_scale[is.na(dat_to_scale)] = 0

  boxplot(dat_to_scale, las = 2)

  min = min(dat_to_scale, na.rm = TRUE)
  max = max(dat_to_scale, na.rm = TRUE)

  limits = floor(max(abs(min), abs(max)))/2

  # create empty vector that will be filled with median values
  median_abundance_vector = vector(mode = "expression", length =
length(colnames(dat_to_scale)))
  for (j in 1:length(colnames(merge_dat))){
    M = na.omit(merge_dat[,j])
    median_abundance_vector[j] = median(M)
  }
  head(median_abundance_vector)

  mid = round(median(unlist(lapply(median_abundance_vector, as.numeric))), digits =
0)
  min = -limits
  max = limits

  dend_rows = dendsort(hclust(dist(dat_to_scale))) #can not be used with NAs
```

```
  dend_cols = dendsort(hclust(dist(t(dat_to_scale))))
  dend_cols = color_branches(dend_cols, k = floor(dim(dat_to_scale)[2]/2))

  plot_file = "Heatmap_zscores_DEproteins_imputed.pdf"
  plot_path = paste(plots_dir, plot_file, sep = dirsep)
  pdf(file = plot_path,
      width = dim(merge_dat)[2],
      height = 8)

  h = Heatmap(
    merge_dat,
    col = colorRamp2(c(min, mid, max), c(color_down, "white", color_up)),
    color_space = "sRGB",
    na_col = "grey",
    cluster_rows = dend_rows,
    cluster_columns = dend_cols,
    clustering_distance_columns = distance_measure,
    clustering_distance_rows = distance_measure,
    clustering_method_columns = clustering_method,
    clustering_method_rows = clustering_method,
    column_dend_side = "top",
    column_dend_height = unit(3, "cm"),
    column_names_side = "top",
    show_row_names = "FALSE",
    #row_names_side = "left",
    #row_names_max_width = unit(15, "cm"),
    column_names_max_height = unit(6, "cm"),
    show_row_dend = TRUE,
    #row_title_gp = gpar(cex = 0.6),
    column_names_gp = gpar(cex = 1, fontface = "bold"),
    #column_dend_reorder=c(1,3,4,5,2),
    #row_dend_reorder=F,
    heatmap_legend_param = list(
      title = "z-score",
      title_gp = gpar(cex = 1, fontface = "bold"),
      color_bar = c("continuous")
    ),
    show_heatmap_legend = TRUE
  )

  print(h)

  dev.off()

  #plot with discrete breaks
discrete_sequence = seq(from = min, to = max, by = 0.5)
discrete_colors = colorRampPalette(colors = c(color_down,
                                              "white",
color_up))(length(discrete_sequence))

  plot_file = "Heatmap_zscores_DEproteins_imputed_discrete.pdf"
  plot_path = paste(plots_dir, plot_file, sep = dirsep)
  pdf(file = plot_path,
      width = dim(merge_dat)[2],
      height = 8)

  h = Heatmap(
  merge_dat,
  col = colorRamp2(breaks = discrete_sequence, colors = discrete_colors),
  color_space = "sRGB",
  na_col = "grey",
  cluster_rows = dend_rows,
  cluster_columns = dend_cols,
  clustering_distance_columns = distance_measure,
  clustering_distance_rows = distance_measure,
  clustering_method_columns = clustering_method,
  clustering_method_rows = clustering_method,
  column_dend_side = "top",
  column_dend_height = unit(3, "cm"),
  column_names_side = "top",
```

```
  show_row_names = "FALSE",
  #row_names_side = "left",
  #row_names_max_width = unit(15, "cm"),
  column_names_max_height = unit(6, "cm"),
  show_row_dend = TRUE,
  row_dend_width = unit(2, "cm"),
  #row_title_gp = gpar(cex = 0.6),
  column_names_gp = gpar(cex = 1, fontface = "bold"),
  #column_dend_reorder=c(3,2,1,7,4,5,6),
  #row_dend_reorder=F,
  heatmap_legend_param = list(
    title = "z-score",
    at = seq(from = min, to = max, by = max/2),
    title_gp = gpar(cex = 1, fontface = "bold"),
    color_bar = c("discrete")
  ),
  show_heatmap_legend = TRUE
)

print(h)

dev.off()

plot_file = "Heatmap_zscores_DEproteins_imputed_discrete.wmf"
  plot_path = paste(plots_dir, plot_file, sep = dirsep)
  win.metafile(file = plot_path,
      width = dim(merge_dat)[2],
      family = define_font,
      height = 8)

  print(h)

dev.off()

plot_file = "Heatmap_zscores_DEproteins_imputed_discrete.svg"
  plot_path = paste(plots_dir, plot_file, sep = dirsep)
  svg(file = plot_path,
      width = dim(merge_dat)[2],
      family = define_font,
      height = 8)

  print(h)

dev.off()

plot_file = "Heatmap_zscores_DEproteins_imputed_discrete.png"
  plot_path = paste(plots_dir, plot_file, sep = dirsep)
  png(file = plot_path,
      units = "in",
      res = 800,
      width = dim(merge_dat)[2],
      family = define_font,
      height = 8)

  print(h)

dev.off()
` ` `
```

Code used | Enrichment using MSigDB

```
---
title: "Pathway Analysis MSIGDB"
author: "Isabel Karkossa, Alix Aldehoff"
date: "December 14, 2017"
---

## 1. Data input

### 1.1 Set session options
```{r setup, include=FALSE}
knitr::opts_chunk$set(echo = TRUE)
knitr::opts_chunk$set(error = TRUE)
knitr::opts_knit$set(root.dir = "//working-directory") # directory containing this
Rscript
```

```{r define path}
dirsep = "/" # the separator for directories on your system
project_dir = getwd()
plots = "Plots" # subdirectory that will hold all plots in .pdf format
plots_dir = paste(project_dir, plots, sep=dirsep) # leave this like it is!
Rdata = "Rdata" # subdirectory that will hold all intermediate .RData files
Rdata_dir = paste(project_dir, Rdata, sep=dirsep) # leave this like it is!
```

```{r define your font}
define_font = "Arial"
windowsFonts("Arial" = windowsFont("Arial"))
```

1.2 Install necessary packages and load organism-specific genome

the organism-specfic packages have names of the form org.Xx.eg.db, where Xx stands
for organism code, for example, Mm for mouse, Hs for human, Rn for rattus
norvegicus.

```{r install_packages, include=FALSE, message=FALSE, results='hide'}
list.of.packages = c("ggplot2", "DOSE", "clusterProfiler", "splitstackshape",
"extrafont", "msigdbr", "biomaRt", "clusterProfiler", "topGO", "ggplot2",
"pathview", "ggsci", "dendsort", "dendextend", "circlize", "scales",
"org.Hs.eg.db")
new.packages = list.of.packages[!(list.of.packages %in%
installed.packages()[,"Package"])]
if (length(new.packages)) install.packages(new.packages)

# the organism-specfic packages have names of the form org.Xx.eg.db, where Xx
stands for organism code, for example, Mm ("org.Mm.eg.db") for mouse, Hs for human


new.packages = list.of.packages[!(list.of.packages %in%
installed.packages()[,"Package"])]

if(length(new.packages)){

  if (!requireNamespace("BiocManager", quietly = TRUE))
    install.packages("BiocManager")

  for (i in new.packages){
    BiocManager::install(i)
  }
}

# load packages
for (i in list.of.packages){
```
```

````
  library(i, character.only = TRUE)
}

#get session info
sessionInfo()

# get versions of packages
for (i in list.of.packages){
  get_version = packageVersion(i)
  print(paste(i, get_version, sep = ": version "))
}
```


### 1.3 Load required packages

Installed genome database and loaded database should be the same!


```{r define font}
#font_import(pattern = "[A/a]rial")
#fonts()
#fonttable()

loadfonts(device = "win")
```

```{r import and load fonts and colors}
#use this to install more fonst:
#font_import(pattern = "[A/a]rial")

#have a look which fonts are available
#fonts()
#fonttable()

#load fonts
loadfonts(device = "win")
windowsFonts("Arial" = windowsFont("Arial"))

#define colors for heatmaps with FCs
color_up = ggsci::pal_npg(palette = "nrc")(10)[8]
color_down = ggsci::pal_npg(palette = "nrc")(10)[4]

#define colors for heatmaps with Abundances
color_high = ggsci::pal_npg(palette = "nrc")(10)[8]
color_mid = "grey"
color_low = ggsci::pal_npg(palette = "nrc")(10)[4]

barplot(seq(1,10, by = 1), col = ggsci::pal_npg(palette = "nrc")(10))
```

1.4 Load data

```{r load_data}
msigdbr_show_species()
myOrg = "Mus musculus"
topx = 10

#load FC and p-value data
sampleTable_file = "dat_IPA_cbind_proteins_pvalue.adj_proteins.csv" # provide your
filename
sampleTable_path = paste(project_dir, sampleTable_file, sep=dirsep) # leave this
like it is!
sample_dat_FC_pvalue = read.csv(sampleTable_path, header = TRUE)

sample_dat_FC_pvalue = sample_dat_FC_pvalue[, !apply(sample_dat_FC_pvalue == 0, 2,
all)]
sample_dat_FC_pvalue = sample_dat_FC_pvalue[, !apply(sample_dat_FC_pvalue == 1, 2,
all)]
````

````
dat_vector = colnames(sample_dat_FC_pvalue)
dat_vector = dat_vector[-1]
dat_vector

sample_vector = unique(gsub(dat_vector, pattern = "_pvalue", replacement = ""))
sample_vector

colnames(sample_dat_FC_pvalue) = c("Accession", dat_vector)
sample_dat_FC_pvalue$Accession = as.character(sample_dat_FC_pvalue$Accession)

head(sample_dat_FC_pvalue)

accessions = data.frame(do.call("rbind", strsplit(sample_dat_FC_pvalue$Accession,
";",
                                                  fixed = TRUE)))

sample_dat_FC_pvalue$Accession = as.character(accessions[, 1])

filename = "dat_IPA_split.csv"
pathname = paste(Rdata_dir, filename, sep = dirsep)

write.csv(x = sample_dat_FC_pvalue, file = pathname, row.names = FALSE)


#load uniprot gene mapping
sampleTable_file = "genes_proteins.csv" # provide your filename
sampleTable_path = paste(project_dir, sampleTable_file, sep=dirsep) # leave this
like it is!
sampleGenes = read.csv(sampleTable_path, header = TRUE, row.names = 1)

for (z in 1:ncol(sampleGenes)){
  sampleGenes[, z] = as.character(sampleGenes[, z])
}

#load sample mapping
sampleTable_file = "ComparisonMapping_IPA_proteome.csv" # provide your filename
sampleTable_path = paste(project_dir, sampleTable_file, sep=dirsep) # leave this
like it is!
sampleMapping = read.csv(sampleTable_path, header = TRUE)

for (z in 1:ncol(sampleMapping)){
  sampleMapping[, z] = as.character(sampleMapping[, z])
}

sampleMapping$Order = as.numeric(as.character(sampleMapping$Order))
```

## 2 Preparation for Pathway Analysis

```{r set_db}
#listMarts()
mart = useEnsembl("ENSEMBL_MART_ENSEMBL", dataset = "mmusculus_gene_ensembl",
mirror = "useast")

#"hsapiens_gene_ensembl" for human genome
#"rnorvegicus_gene_ensembl" for rattus
#"mmusculus_gene_ensembl" for mouse
#listDatasets(mart)

#Filters define a restriction on the query.  For example you want to restrict the
output to all genes located on the human X chromosome then the filter chromosome
name can be used with value 'X'.
#filters = listFilters(mart)
#filters

#Attributes define the values we are interested in to retrieve.  For example we
want to retrieve the gene symbols
attributes = listAttributes(mart)
#attributes
#head(attributes)
````

```
filters = listFilters(mart)
```

```{r map entrez}
dat_getHall_1 = biomaRt::getBM(attributes = c("uniprotswissprot",
                                              "entrezgene_id"),
                               filters = "uniprotswissprot",
                               values = sample_dat_FC_pvalue$Accession,
                               useCache = FALSE,
                               mart = mart)

colnames(dat_getHall_1) = c("Accession", "Entrez")

dat_getHall_2 = biomaRt::getBM(attributes = c("uniprotsptrembl",
                                              "entrezgene_id"),
                               filters = "uniprotsptrembl",
                               values = sample_dat_FC_pvalue$Accession,
                               useCache = FALSE,
                               mart = mart)

colnames(dat_getHall_2) = c("Accession", "Entrez")

dat_getHall = rbind(dat_getHall_1, dat_getHall_2)

dat_getHall = dat_getHall[!duplicated(dat_getHall$Accession), ]

sample_dat_FC_pvalue_Entrez = merge(sample_dat_FC_pvalue, dat_getHall, by =
"Accession")

filename = "dat_IPA_split_Entrez.csv"
pathname = paste(Rdata_dir, filename, sep = dirsep)

write.csv(x = sample_dat_FC_pvalue_Entrez, file = pathname, row.names = FALSE)
```

```{r chose dataset}
dataset_chosen = "KEGG"

# http://www.gsea-msigdb.org/gsea/msigdb/collections.jsp
# m_df = msigdbr(species = myOrg, category = "H") #hallmark gene sets
m_df = msigdbr(species = myOrg, category = "C2", subcategory = "KEGG") #KEGG gene
sets
# m_df = msigdbr(species = myOrg, category = "C5", subcategory = "MF") #GO BP gene
sets

head(m_df)

m_t2g = m_df %>% dplyr::select(gs_name, entrez_gene) %>% as.data.frame()
```

```{r consider relevant terms}
relevant_processes_pattern = c("KEGG_PPAR_SIGNALING_PATHWAY",
                               "KEGG_OXIDATIVE_PHOSPHORYLATION",
                               "KEGG_CITRATE_CYCLE_TCA_CYCLE",
                               "KEGG_GLYCOLYSIS_GLUCONEOGENESIS"
                               )

relevant_processes_pattern = unique(relevant_processes_pattern)

for (i in 1:length(relevant_processes_pattern)){
  tryCatch({
    relevant_dat = m_df[grepl(m_df$gs_name, pattern =
relevant_processes_pattern[i]), ]

    combine_with_own_dat = merge(relevant_dat, sample_dat_FC_pvalue_Entrez, by.x =
"entrez_gene",
                                 by.y = "Entrez")
```

```
    combine_with_own_dat =
combine_with_own_dat[!duplicated(combine_with_own_dat$Accession), ]

    get_dat_to_cluster = combine_with_own_dat[, sample_vector]
    get_dat_to_cluster[is.na(get_dat_to_cluster)] = 0

    dend_rows = dendsort(hclust(dist(get_dat_to_cluster)))

    get_cluster_order = dend_rows$order

    for (j in 1:length(sample_vector)){
      sample_dat = combine_with_own_dat[, grepl(colnames(combine_with_own_dat),
                                          pattern = sample_vector[j])]

      colnames(sample_dat) = c("FC", "pvalue")

      sample_dat$Treatment = sample_vector[j]
      sample_dat$Process = combine_with_own_dat$gs_name
      sample_dat$Accession = combine_with_own_dat$Accession
      sample_dat$Entrez = combine_with_own_dat$entrez_gene
      sample_dat$ClusterOrder = get_cluster_order

      assign(paste("relevant_dat_to_ggplot", relevant_processes_pattern[i],
sample_vector[j],
                   sep = "_"),
             value = sample_dat)

    }

    dat_rbind = do.call(rbind, lapply(ls(pattern = paste("relevant_dat_to_ggplot",

relevant_processes_pattern[i],
                                                         sep = "_")),
                                      function(x) {
                                        get_dat = get(x)
                                        get_dat
                                      }))

    dat_genes = merge(dat_rbind, sampleGenes, by.x = "Accession", by.y =
"row.names")

    dat_to_plot = dat_genes

    dat_to_plot$Label = ""
    dat_to_plot$Label[dat_to_plot$pvalue <= 0.05] = "*"
    dat_to_plot$Label[dat_to_plot$pvalue <= 0.01] = "**"
    dat_to_plot$Label[dat_to_plot$pvalue <= 0.001] = "***"

    dat_all = merge(dat_to_plot, sampleMapping, by = "Treatment")
    dat_all = dat_all[order(dat_all$Order), ]

    dat_all$Treatment = factor(dat_all$Treatment, levels =
unique(dat_all$Treatment))
    dat_all = dat_all[order(dat_all$Treatment), ]

    dat_all$Name = factor(dat_all$Name, levels = unique(dat_all$Name))
    dat_all = dat_all[order(dat_all$Name), ]

    gg_keydrivers = ggplot(dat_all, aes(x = Name,
                                        y = reorder(Gene, ClusterOrder),
                                        fill = FC, label = Label)) +
      geom_tile(width = 1, height = 1) +
      geom_text(aes(label = Label), hjust = 0.5, vjust = 0.8, size = 8*5/14) +
      scale_fill_gradient2(low = color_down,
                           mid = "white",
                           high = color_up, name = "Log2(FC)",
                           midpoint = 0,
                           na.value = "grey",
                           breaks = c(-5, -2.5, 0, 2.5, 5),
                           limits = c(-5, 5),
```

```
                           oob = squish) +
      theme_classic() +
      theme(legend.position = "right",
            legend.title = element_text(size = 8, color = "black", face = "plain"),
            legend.text = element_text(size = 6, color = "black", face = "plain"),
            legend.key.size = unit(0.5, "cm"),
            panel.border = element_rect(fill = NA, colour = "black", size = 0.5),
            axis.ticks = element_line(colour = "black", size = 0.5),
            axis.line = element_blank(),
            plot.title = element_text(size = 8, color = "black", face = "bold"),
            axis.title.y = element_blank(),
            axis.title.x = element_blank(),
            axis.text.x = element_text(size = 8, face = "plain",
                                       angle = 90,
                                       color = "black",
                                       #color = color_list,
                                       vjust = 0.5,
                                       hjust = 1),
            axis.text.y = element_text(size = 8, face = "plain",
                                       color = "black",
                                       #color = color_list,
                                       hjust = 1)) +
      ggtitle(relevant_processes_pattern[i]) +
      #facet_grid(Comparison ~ .,
      #          drop = TRUE, scales = "free", space = "free", switch = "y") +
      facet_grid(. ~ Comparison,
                 drop = TRUE, scales = "free", space = "free", switch = "y")  +
      theme(panel.background = element_rect(fill = NA, color = "black"),
            strip.placement = "outside",
            strip.text.x = element_text(size=8, face = "bold", color = "black"),
            strip.text.y = element_text(size=8, face = "bold", color = "black"),
            strip.background = element_rect(colour="white", fill="grey"))

    filename = paste("Summary_", relevant_processes_pattern[i], "_ggplot.pdf", sep
= "")
    plotfile = paste(plots_dir, filename, sep=dirsep)
    pdf(file = plotfile,
        width = (length(unique(dat_all$Treatment))/8) +4,
        height = (length(unique(dat_all$Accession))/10) +8,
        pointsize = 10)

    print(gg_keydrivers)
    dev.off()

    filename = paste("Summary_", relevant_processes_pattern[i], "_ggplot.png", sep
= "")
    plotfile = paste(plots_dir, filename, sep=dirsep)
    png(file = plotfile,
        units = "in",
        res = 800,
        width = (length(unique(dat_all$Treatment))/8) +4,
        height = (length(unique(dat_all$Accession))/10) +8,
        pointsize = 10)

    print(gg_keydrivers)
    dev.off()

    gg_keydrivers = ggplot(dat_all, aes(y = Name,
                                        x = reorder(Gene, ClusterOrder),
                                        fill = FC, label = Label)) +
      geom_tile(width = 1, height = 1) +
      geom_text(aes(label = Label), hjust = 0.5, vjust = 0.8, size = 8*5/14) +
      scale_fill_gradient2(low = color_down,
                           mid = "white",
                           high = color_up, name = "Log2(FC)",
                           midpoint = 0,
                           na.value = "grey",
                           breaks = c(-5, -2.5, 0, 2.5, 5),
                           limits = c(-5, 5),
                           oob = squish) +
```

~~~~
      theme_classic() +
      theme(legend.position = "right",
            legend.title = element_text(size = 8, color = "black", face = "plain"),
            legend.text = element_text(size = 6, color = "black", face = "plain"),
            legend.key.size = unit(0.3, "cm"),
            #legend.key.height = unit(0.5, "cm"),
            panel.border = element_rect(fill = NA, colour = "black", size = 0.5),
            axis.ticks = element_line(colour = "black", size = 0.5),
            axis.line = element_blank(),
            plot.title = element_text(size = 8, color = "black", face = "bold"),
            axis.title.y = element_blank(),
            axis.title.x = element_blank(),
            axis.text.x = element_text(size = 8, face = "plain",
                                       angle = 90,
                                       color = "black",
                                       #color = color_list,
                                       vjust = 0.5,
                                       hjust = 1),
            axis.text.y = element_text(size = 8, face = "plain",
                                       color = "black",
                                       #color = color_list,
                                       hjust = 1)) +
      ggtitle(relevant_processes_pattern[i])+
      facet_grid(. ~ Comparison,
                 drop = TRUE, scales = "free", space = "free", switch = "y") +
      theme(panel.background = element_rect(fill = NA, color = "black"),
            strip.placement = "outside",
            strip.text.x = element_text(size=8, face = "bold", color = "black"),
            strip.text.y = element_text(size=8, face = "bold", color = "black"),
            strip.background = element_rect(colour="white", fill="grey"))

    filename = paste("Summary_", relevant_processes_pattern[i],
"_horizontally_ggplot.pdf", sep = "")
    plotfile = paste(plots_dir, filename, sep=dirsep)
    pdf(file = plotfile,
        height = (length(unique(dat_all$Treatment))/10) +2,
        width = (length(unique(dat_all$Accession))/2) +3,
        pointsize = 10)

    print(gg_keydrivers)
    dev.off()

    filename = paste("Summary_", relevant_processes_pattern[i],
"_horizontally_ggplot.png", sep = "")
    plotfile = paste(plots_dir, filename, sep=dirsep)
    png(file = plotfile,
        units = "in",
        res = 800,
        height = (length(unique(dat_all$Treatment))/10) +2,
        width = (length(unique(dat_all$Accession))/2) +3,
        pointsize = 10)

    print(gg_keydrivers)
    dev.off()

    filename = paste("Summary_", relevant_processes_pattern[i], ".csv", sep = "")
    pathname = paste(Rdata_dir, filename, sep = dirsep)

    write.csv(dat_all, file = pathname)
  }, error = function(e){cat("ERROR:", conditionMessage(e),
relevant_processes_pattern[i], "\n")})
}

```



## 2 Enrichment Analysis

```{r download hallmark}
~~~~

```
for (i in sample_vector){
  tryCatch({
    get_dat = sample_dat_FC_pvalue_Entrez[,
which(grepl(colnames(sample_dat_FC_pvalue_Entrez),
                                                        pattern = i) == TRUE)]

    get_dat$KEGG = sample_dat_FC_pvalue_Entrez$Entrez

    subset_dat = get_dat[which(get_dat[, 2] <= 0.05), ]

    dat_getKEGG = as.vector(as.character(subset_dat$KEGG))

    enrich_KEGG = clusterProfiler::enricher(gene = dat_getKEGG, TERM2GENE = m_t2g)

    assign(value = enrich_KEGG,x = paste("enrich_done", i, sep = "_"))

    #Calculate Enrichment Factor
    Enrichment_result = as.data.frame(enrich_KEGG@result)

    Enrichment_result_GeneRatio = as.data.frame(Enrichment_result$GeneRatio)
    colnames(Enrichment_result_GeneRatio) = c("GeneRatio")
    Enrichment_result_GeneRatio_split = cSplit(Enrichment_result_GeneRatio,
splitCols = "GeneRatio", sep = "/")
    Enrichment_result_GeneRatio_final =
Enrichment_result_GeneRatio_split$GeneRatio_1 /
      Enrichment_result_GeneRatio_split$GeneRatio_2

    Enrichment_result_BgRatio = as.data.frame(Enrichment_result$BgRatio)
    colnames(Enrichment_result_BgRatio) = c("BgRatio")
    Enrichment_result_BgRatio_split = cSplit(Enrichment_result_BgRatio, splitCols =
"BgRatio", sep = "/")
    Enrichment_result_BgRatio_final = Enrichment_result_BgRatio_split$BgRatio_1 /
      Enrichment_result_BgRatio_split$BgRatio_2

    Enrichment_result$Enrichment =
Enrichment_result_GeneRatio_final/Enrichment_result_BgRatio_final

    mean_df = as.data.frame(matrix(nrow = nrow(Enrichment_result), ncol =
length(sample_vector),
                                   NA))

    colnames(mean_df) = paste("Median_", sample_vector, sep = "")
    rownames(mean_df) = rownames(Enrichment_result)

    for (j in 1:nrow(Enrichment_result)){
      get_targets = t(data.frame(do.call("rbind",
strsplit(Enrichment_result$geneID[j], "/",
                                                           fixed = TRUE))))
      colnames(get_targets) = c("Entrez")

      get_candidate_data = merge(get_targets, sample_dat_FC_pvalue_Entrez, by =
"Entrez")
      get_candidate_data = get_candidate_data[, sample_vector]

      get_median = apply(get_candidate_data, 2, function(x){median(x, na.rm =
TRUE)})

      mean_df[j, ] = get_median
    }

    combine_results = merge(Enrichment_result, mean_df, by = "row.names")

    Enrichment_result = combine_results
    Enrichment_result$Treatment = i

    dataframe.name = paste("Enrichment_done", dataset_chosen, i, sep = "_")
    assign(value = Enrichment_result, x = dataframe.name)
    file = paste(dataframe.name, ".csv", sep = "")
    resultname = paste(Rdata_dir, file, sep = "/")
```

```
    write.csv(Enrichment_result, file = resultname)

    dat_save_all = Enrichment_result[, c("Row.names",
                                         "Treatment",
                                         "p.adjust",
                                         paste("Median", i, sep = "_"))]

    colnames(dat_save_all) = c("Process", "Treatment", "p.adjust", "Median")

    assign(paste("dat_to_ggplot_all", i, sep = "_"), value = dat_save_all)

    get_sign = Enrichment_result[Enrichment_result$p.adjust <= 0.05, ]

    dat_save_sign = get_sign[, c("Row.names",
                                 "Treatment",
                                 "p.adjust",
                                 paste("Median", i, sep = "_"))]

    colnames(dat_save_sign) = c("Process", "Treatment", "p.adjust", "Median")

    assign(paste("dat_to_ggplot_sign", i, sep = "_"), value = dat_save_sign)

    Enrichment_result = Enrichment_result[order(Enrichment_result$p.adjust), ]

    if (nrow(Enrichment_result) > topx){
      get_topx = Enrichment_result[1:topx, ]
    } else {get_topx = Enrichment_result}

    dat_save_topx = get_topx[, c("Row.names",
                                 "Treatment",
                                 "p.adjust",
                                 paste("Median", i, sep = "_"))]

    colnames(dat_save_topx) = c("Process", "Treatment", "p.adjust", "Median")

    assign(paste("dat_to_ggplot_topx", i, sep = "_"), value = dat_save_topx)


  }, error = function(e){cat("ERROR:", conditionMessage(e), i, "\n")})
}
```

3 Visualization

```{r KEGG Enrichment}
enrichKEGG_Enrichment_result_list = lapply(ls(pattern = "Enrichment_done_"),get)
enrichKEGG_Enrichment_result_names = as.vector(ls(pattern = "Enrichment_done_"))
enrichKEGG_Enrichment_result_names = gsub(enrichKEGG_Enrichment_result_names,
                                          pattern = "Enrichment_done_", replacement
= "")

enrichKEGG_result_list = lapply(ls(pattern = "enrich_done_"),get)
enrichKEGG_result_names = as.vector(ls(pattern = "enrich_done_"))
enrichKEGG_result_names = gsub(enrichKEGG_result_names, pattern = "enrich_done_",
replacement = "")

# enrichKEGG_pathway_dataframe = do.call(rbind, lapply(ls(pattern =
"enrichHALLMARK_done_"),
#                                                      function(x){
#                                                        get_dat = get(x)
#                                                        get_dat =
as.data.frame(get_dat@result)
#                                                        #get_dat =
get_dat[get_dat$p.adjust <= 0.05, ]
#                                                            }))
#
# enrichKEGG_pathway_list = unique(enrichKEGG_pathway_dataframe$ID)
```

````
datafile = paste(Rdata_dir, "KEGG_enrich_result.RData", sep=dirsep)

save(#enrichKEGG_pathway_list,
  enrichKEGG_result_names,
  enrichKEGG_result_list,
  enrichKEGG_Enrichment_result_list,
  enrichKEGG_Enrichment_result_names,
  #sample_dat_FC_pvalue_KEGG,
  sample_dat_FC_pvalue,
  sample_vector,
  file=datafile)
```

```{r load KEGG results, eval=FALSE, include=FALSE}
load(file = datafile)
```


### 3.1 Visualize results

```{r bubble plot}
for (i in 1:length(enrichKEGG_Enrichment_result_list)){
  tryCatch({

    get_Enrichment_result = as.data.frame(enrichKEGG_Enrichment_result_list[[i]])
    get_Enrichment_result = get_Enrichment_result[get_Enrichment_result$p.adjust <=
0.05, ]
    get_Enrichment_result = get_Enrichment_result[order(-
get_Enrichment_result$Enrichment), ]
    #get_Enrichment_result = get_Enrichment_result[1:30, ]

    get_Enrichment_result = transform(get_Enrichment_result, variable =
reorder(Description, Enrichment))
    get_Enrichment_result = na.exclude(get_Enrichment_result)

    maxp = ifelse(-log10(min(get_Enrichment_result$p.adjust)) <=10, 10, -
log10(min(get_Enrichment_result$p.adjust)))

    maxc = ifelse(max(get_Enrichment_result$Count) <=10, 10,
max(get_Enrichment_result$Count))

    b = ggplot(get_Enrichment_result, aes(x = Enrichment, y = variable,
                                          size = Count, fill = -log10(p.adjust))) +
      geom_point(shape = 21) +
      scale_fill_gradient2(low = "white", high = color_up, name = "-
Log10(p.adjust)",
                           breaks = c(1.3, 10), limits = c(1.3,maxp)) +
      scale_size_area(breaks = c(1, 2, 5, 10, 20), limits = c(1, maxc)) +
      theme_classic() +
      theme(legend.position = "right",
            legend.title = element_text(size = 8, color = "black", face = "plain"),
            legend.text = element_text(size = 6, color = "black", face = "plain"),
            legend.key.size = unit(0.5, "cm"),
            panel.border = element_rect(fill = NA, colour = "black", size = 0.5),
            axis.ticks = element_line(colour = "black", size = 0.5),
            axis.line = element_blank(),
            plot.title = element_text(size = 10, color = "black", face = "bold"),
            legend.title.align = 0.5,
            axis.title.y = element_blank(),
            axis.title.x = element_text(size = 8, face = "bold", color = "black"),
            axis.text.x = element_text(size = 8, color = "black"),
            axis.text.y = element_text(size = 8, color = "black", hjust = 1)) +
      labs(size = "Count",
           x = "Enrichment factor (GeneRatio/BgRatio)") +
      ggtitle(enrichKEGG_Enrichment_result_names[i]) +
      guides(fill = guide_colorbar(order = 1, title.hjust = 0.5, title.vjust = 0.5,
ticks = TRUE, label = TRUE),
             size = guide_legend(order = 2, title.hjust = 0.5, title.vjust = 0.5))
````

~~~
    filename = paste("Results_bubbleplot_sign",
enrichKEGG_Enrichment_result_names[i], sep = "_")
    filename = paste(filename, ".pdf", sep = "")
    file = paste(plots_dir, filename, sep = "/")

    pdf(file = file,
        width = 10,
        height = (nrow(get_Enrichment_result)/4) +1,
        pointsize = 10)

    print(b)

    dev.off()

    filename = paste("Results_bubbleplot_sign",
enrichKEGG_Enrichment_result_names[i], sep = "_")
    filename = paste(filename, ".png", sep = "")
    file = paste(plots_dir, filename, sep = "/")

    png(file = file,
        units = "in",
        res = 800,
        width = 10,
        height = (nrow(get_Enrichment_result)/4) +1,
        pointsize = 10)

    print(b)

    dev.off()

  }, error = function(e){cat("ERROR:", conditionMessage(e),
enrichKEGG_Enrichment_result_names[i], "\n")})
}

```

```{r visualize significant processes}
dat_complete = do.call(rbind, lapply(ls(pattern = "dat_to_ggplot_all_"),
                                     function(x) {
                                       get_dat = get(x)
                                       get_dat
                                     }))

dat_to_cluster_all = reshape(dat_complete, idvar = "Process", timevar =
"Treatment",
                             direction = "wide")

rownames(dat_to_cluster_all) = dat_to_cluster_all$Process
dat_to_cluster_wide = dat_to_cluster_all[, grepl(colnames(dat_to_cluster_all),
                                                 pattern = "Median")]

dat_to_cluster_wide[is.na(dat_to_cluster_wide)] = 0

dend_rows = dendsort(hclust(dist(dat_to_cluster_wide)))

order_df = as.data.frame(dend_rows$order)

get_cluster_order = as.data.frame(dend_rows$order)
get_cluster_order$Process = row.names(dat_to_cluster_wide)
colnames(get_cluster_order) = c("ClusterOrder", "Process")

dat_sign = do.call(rbind, lapply(ls(pattern = "dat_to_ggplot_sign_"),
                                 function(x) {
                                   get_dat = get(x)
                                   get_dat
                                 }))

dat_sign_processes = as.data.frame(unique(dat_sign$Process))
colnames(dat_sign_processes) = c("Process")
~~~

```
dat_sign_complete = merge(dat_sign_processes, dat_complete, by = "Process")

dat_sign = dat_sign_complete

dat_sign_merge = merge(dat_sign, get_cluster_order, by = "Process")

dat_to_plot = dat_sign_merge

dat_to_plot$Label = ""
dat_to_plot$Label[dat_to_plot$p.adjust <= 0.05] = "*"
dat_to_plot$Label[dat_to_plot$p.adjust <= 0.01] = "**"
dat_to_plot$Label[dat_to_plot$p.adjust <= 0.001] = "***"

dat_all = merge(dat_to_plot, sampleMapping, by = "Treatment")
dat_all = dat_all[order(dat_all$Order), ]

dat_all$Treatment = factor(dat_all$Treatment, levels = unique(dat_all$Treatment))
dat_all = dat_all[order(dat_all$Treatment), ]

dat_all$Name = factor(dat_all$Name, levels = unique(dat_all$Name))
dat_all = dat_all[order(dat_all$Name), ]

gg_keydrivers = ggplot(dat_all, aes(x = Name,
                                    y = reorder(Process, Median),
                                    fill = Median, label = Label)) +
  geom_tile(width = 1, height = 1) +
  geom_text(aes(label = Label), hjust = 0.5, vjust = 0.8, size = 8*5/14) +
  scale_fill_gradient2(low = color_down,
                       mid = "white",
                       high = color_up, name = "Median Log2(FC)",
                       midpoint = 0,
                       na.value = "grey",
                       breaks = c(-5, -2.5, 0, 2.5, 5),
                       limits = c(-5, 5),
                       oob = squish) +
  theme_classic() +
  theme(legend.position = "right",
        legend.title = element_text(size = 8, color = "black", face = "plain"),
        legend.text = element_text(size = 6, color = "black", face = "plain"),
        legend.key.size = unit(0.5, "cm"),
        panel.border = element_rect(fill = NA, colour = "black", size = 0.5),
        axis.ticks = element_line(colour = "black", size = 0.5),
        axis.line = element_blank(),
        plot.title = element_text(size = 8, color = "black", face = "bold"),
        axis.title.y = element_blank(),
        axis.title.x = element_blank(),
        axis.text.x = element_text(size = 8, face = "plain",
                                   angle = 90,
                                   color = "black",
                                   #color = color_list,
                                   vjust = 0.5,
                                   hjust = 1),
        axis.text.y = element_text(size = 8, face = "plain",
                                   color = "black",
                                   #color = color_list,
                                   hjust = 1)) +
      # facet_grid(Comparison ~ .,
      #            drop = TRUE, scales = "free", space = "free", switch = "y") +
      facet_grid(. ~ Comparison,
                 drop = TRUE, scales = "free", space = "free", switch = "y")  +
      theme(panel.background = element_rect(fill = NA, color = "black"),
            strip.placement = "outside",
            strip.text.x = element_text(size=8, face = "bold", color = "black"),
            strip.text.y = element_text(size=8, face = "bold", color = "black"),
            strip.background = element_rect(colour="white", fill="grey"))

filename = paste("Heatmap_sign_", dataset_chosen, "_ggplot.pdf", sep = "")
plotfile = paste(plots_dir, filename, sep=dirsep)
pdf(file = plotfile,
    width = (length(unique(dat_all$Treatment))/4) +10,
```

```
    height = (length(unique(dat_all$Process))/6) +2,
    pointsize = 10)

print(gg_keydrivers)
dev.off()

filename = paste("Heatmap_sign_", dataset_chosen, "_ggplot.png", sep = "")
plotfile = paste(plots_dir, filename, sep=dirsep)
png(file = plotfile,
    units = "in",
    res = 800,
    width = (length(unique(dat_all$Treatment))/4) +10,
    height = (length(unique(dat_all$Process))/6) +2,
    pointsize = 10)

print(gg_keydrivers)
dev.off()

```

```{r visualize topx processes}
dat_sign = do.call(rbind, lapply(ls(pattern = "dat_to_ggplot_topx_"),
                                 function(x) {
                                   get_dat = get(x)
                                   get_dat
                                 }))

dat_sign_processes = as.data.frame(unique(dat_sign$Process))
colnames(dat_sign_processes) = c("Process")

dat_sign_complete = merge(dat_sign_processes, dat_complete, by = "Process")

dat_sign = dat_sign_complete

dat_sign_merge = merge(dat_sign, get_cluster_order, by = "Process")

dat_to_plot = dat_sign_merge

dat_to_plot$Label = ""
dat_to_plot$Label[dat_to_plot$p.adjust <= 0.05] = "*"
dat_to_plot$Label[dat_to_plot$p.adjust <= 0.01] = "**"
dat_to_plot$Label[dat_to_plot$p.adjust <= 0.001] = "***"

dat_all = merge(dat_to_plot, sampleMapping, by = "Treatment")
dat_all = dat_all[order(dat_all$Order), ]

dat_all$Treatment = factor(dat_all$Treatment, levels = unique(dat_all$Treatment))
dat_all = dat_all[order(dat_all$Treatment), ]

dat_all$Name = factor(dat_all$Name, levels = unique(dat_all$Name))
dat_all = dat_all[order(dat_all$Name), ]

gg_keydrivers = ggplot(dat_all, aes(x = Name,
                                    y = reorder(Process, ClusterOrder),
                                    fill = Median, label = Label)) +
  geom_tile(width = 1, height = 1) +
  geom_text(aes(label = Label), hjust = 0.5, vjust = 0.8, size = 8*5/14) +
  scale_fill_gradient2(low = color_down,
                       mid = "white",
                       high = color_up, name = "Median Log2(FC)",
                       midpoint = 0,
                       na.value = "grey",
                       breaks = c(-5, -2.5, 0, 2.5, 5),
                       limits = c(-5, 5),
                       oob = squish) +
  theme_classic() +
  theme(legend.position = "right",
        legend.title = element_text(size = 8, color = "black", face = "plain"),
        legend.text = element_text(size = 6, color = "black", face = "plain"),
        legend.key.size = unit(0.5, "cm"),
```

```
        panel.border = element_rect(fill = NA, colour = "black", size = 0.5),
        axis.ticks = element_line(colour = "black", size = 0.5),
        axis.line = element_blank(),
        plot.title = element_text(size = 8, color = "black", face = "bold"),
        axis.title.y = element_blank(),
        axis.title.x = element_blank(),
        axis.text.x = element_text(size = 8, face = "plain",
                                   angle = 90,
                                   color = "black",
                                   #color = color_list,
                                   vjust = 0.5,
                                   hjust = 1),
        axis.text.y = element_text(size = 8, face = "plain",
                                   color = "black",
                                   #color = color_list,
                                   hjust = 1)) +
      # facet_grid(Comparison ~ .,
      #            drop = TRUE, scales = "free", space = "free", switch = "y") +
      facet_grid(. ~ Comparison,
                 drop = TRUE, scales = "free", space = "free", switch = "y")  +
      theme(panel.background = element_rect(fill = NA, color = "black"),
            strip.placement = "outside",
            strip.text.x = element_text(size=8, face = "bold", color = "black"),
            strip.text.y = element_text(size=8, face = "bold", color = "black"),
            strip.background = element_rect(colour="white", fill="grey"))

filename = paste("Heatmap_Top", topx, "_combined_", dataset_chosen, "_ggplot.pdf",
sep = "")
plotfile = paste(plots_dir, filename, sep=dirsep)
pdf(file = plotfile,
    width = (length(unique(dat_all$Treatment))/4) +8,
    height = (length(unique(dat_all$Process))/6) +2,
    pointsize = 10)

print(gg_keydrivers)
dev.off()

filename = paste("Heatmap_Top", topx, "_combined_", dataset_chosen, "_ggplot.png",
sep = "")
plotfile = paste(plots_dir, filename, sep=dirsep)
png(file = plotfile,
    units = "in",
    res = 800,
    width = (length(unique(dat_all$Treatment))/4) +8,
    height = (length(unique(dat_all$Process))/6) +2,
    pointsize = 10)

print(gg_keydrivers)
dev.off()

```

```
```{r visualize_KEGG_results_cnetplot, eval=FALSE, include=FALSE}
enrichKEGG_result_names
getKEGG_result_names

for (i in 1:length(enrichKEGG_result_list)){
  tryCatch({
    get_dat = enrichKEGG_result_list[[i]]

    for (k in 1:length(getKEGG_result_list)){
      tryCatch({

        KEGG_dat = getKEGG_result_list[[k]]


        for (j in sample_vector){
          tryCatch({
            dat_subset = as.data.frame(KEGG_dat[ , grepl(j, colnames(KEGG_dat))])
```
```

```
            dat_subset = as.data.frame(dat_subset[, 1])
            dat_subset$KEGG = KEGG_dat$KEGG
            dat_subset = na.exclude(dat_subset)
            rownames(dat_subset) = dat_subset$KEGG
            colnames(dat_subset) = c("FCs", "KEGG")
            dat_subset = dat_subset[order(-dat_subset$FCs), ]
            dat_subset_final = as.vector(dat_subset$FCs)
            names(dat_subset_final) = rownames(dat_subset)

            filename = paste("KEGG_results_cnetplot", enrichKEGG_result_names[i],
j,
                             sep = "_")
            filename = paste(filename, ".wmf", sep = "")
            file = paste(plots_dir, filename, sep = "/")

            win.metafile(file = file,
                         width = 10, height = 10,
                         pointsize = 10,
                         family = define_font)

            cnetplot(get_dat, foldChange = dat_subset_final)

            dev.off()

            filename = paste("KEGG_results_cnetplot", enrichKEGG_result_names[i],
j,
                             sep = "_")
            filename = paste(filename, ".pdf", sep = "")
            file = paste(plots_dir, filename, sep = "/")

            pdf(file = file,
                width = 10, height = 10,
                pointsize = 10,
                family = define_font)

            cnetplot(get_dat, foldChange = dat_subset_final)

            dev.off()

          }, error = function(e){cat("ERROR:", conditionMessage(e),
enrichKEGG_result_names[i], j, "\n")})
        }

      }, error = function(e){cat("ERROR:", conditionMessage(e),
enrichKEGG_result_names[i], j, "\n")})
    }

  }, error = function(e){cat("ERROR:", conditionMessage(e),
enrichKEGG_result_names[i], j, "\n")})
}
```

````
---
title: "WGCNA - 0 Data preparation"
author: "Isabel Karkossa, Alix Aldehoff"
date: "5 April 2017"
---

Please enter your main root directory here. This will be used by all the chunks.

```{r setup, include=FALSE}
knitr::opts_chunk$set(echo = TRUE)
knitr::opts_chunk$set(error = TRUE)
knitr::opts_knit$set(root.dir = "//working-directory") # directory containing this
Rscript
```

# WGCNA Analysis

## 0. Define paths for your output

This script will produce some output.
Please decide here the base paths to the directories where you want to have it! The
paths for each folder has to be entered twice. Ones for creating and ones for
saving the path. Furthermore the desktop should be chosen as one path to facilitate
the loading of saved data for the next WGCNA scripts.

```{r define_output_paths}
dirsep = "/" # the separator for directories on your system
project_dir = getwd()
plots = "Plots" # subdirectory that will hold all plots in .pdf format
plots_dir = paste(project_dir, plots, sep=dirsep) # leave this like it is!
Rdata = "Rdata" # subdirectory that will hold all intermediate .RData files
Rdata_dir = paste(project_dir, Rdata, sep=dirsep) # leave this like it is!
```

All filenames that you provide for output don't need to be provided with the full
path now!

```{r define your font}
define_font = "Arial"
```

## 1. Data input, cleaning and pre-processing

Install packages if necessary. Don't change!

```{r install_packages, include=FALSE, message=FALSE, results='hide'}
list.of.packages = c("WGCNA", "flashClust", "extrafont", "DESeq2", "biomaRt",
"GO.db")
new.packages = list.of.packages[!(list.of.packages %in%
installed.packages()[,"Package"])]
if(length(new.packages)) install.packages(new.packages)

new.packages = list.of.packages[!(list.of.packages %in%
installed.packages()[,"Package"])]

if(length(new.packages)){

  if (!requireNamespace("BiocManager", quietly = TRUE))
    install.packages("BiocManager")

  for (i in new.packages){
    BiocManager::install(i)
  }
}
````
````

````
```

Load required packages and enable multi-threading. Don't change!

- **DESeq2:** to anaylse the samples and calculate a transformation for the network
- **WGCNA:** to generate and analyse the network
- **flasClust:** to calculate clusters with high speed
- **biomaRt:** for the annotation of the genes (later?!)

```{r load_packages}
for (i in list.of.packages){
  library(i, character.only = TRUE)
}

options(stringsAsFactors = FALSE)
```

```{r define font}
#font_import(pattern = "[A/a]rial")
#fonts()
#fonttable()

loadfonts(device = "win")
```

Provide the filenames and directories of your input. Please save your sampleTable
file along with this Rscript for your own conveniance.

1.1 Trait data

WGCNA needs trait data - clinical measurements/treatments - for the samples.
It is encouraged to transform trait data to numerical data, binary in the ideal
case.

*Explain the samples and treatments of your experiment here *
The SampleDescriptionMatrix has to contain the samples in the first column and the
groups that are defined with 1 or 0 (meaning TRUE or FALSE) in the following
columns. This means samples in rows and groups in columns.

```{r define_input_paths}
# sample-treatment info
sampleTable_file = "dat_relreps_INTEGRATED.csv" # provide your filename
sampleTable_path = paste(project_dir, sampleTable_file, sep=dirsep) # leave this
like it is!
```
Load sample table.

```{r load_sample_table}
dat_expr = read.csv(sampleTable_path, header = TRUE, row.names = 1)

dat_expr[dat_expr == 0] = NA
```

```{r define_input_paths_traits}
# sample-treatment info
sampleTable_file = "SampleDescriptionMatrix.csv" # provide your filename
sampleTable_path = paste(project_dir, sampleTable_file, sep=dirsep) # leave this
like it is!
```
Load sample table.

```{r load_sample_table_traits}
sampleTable = read.csv(sampleTable_path, header = TRUE)
```

Nothing has to be changed in the following chunks! Just run the script (Knit)!
After running the script the first time, it should be decided whether some samples
should be excluded from the analysis because they are assembled in a different
cluster than all the other samples. In this case change the height2cut value to an
````
````

````
appropriate value. For now (300) it is defined that high, that no samples should be
excluded.

```{r height2cut}
height2cut = 200
```

_________________________________________________________________________________
__________________
This script will cluster the data the first time and allows you to remove outliers
before the actual WGCNA.
_________________________________________________________________________________
__________________

```{r save_dds}
# you may need to filter again after normalization
# exprData = vst.df[rowSums(apply(vst.df > log2(20), c(1,2), as.numeric)) >= 3, ]
exprData = dat_expr # has to be changed if the normalized data were filtered
datafile = paste(Rdata_dir, "01_exprData.RData", sep=dirsep)
save(exprData, file=datafile)
# load(datafile) # will reload this data structure
```

The normalized expression data ```exprData``` can be found in the file **```{r
exprData_path}```**.

### 1.3 Initial clustering of samples

This helps to identify outlying samples, consider removing them from further
analysis.

For WGCNA we need to reorganize the dataframe containing the normalized expr. data.

```{r WGCNA_input}
datExpr0 = as.data.frame(t(exprData))
dim(datExpr0)
```

Exclude missing values and identify outliers

If allOK gives TRUE, it means that all the genes went through the cuts.
If it gives FALSE, then some genes and/or samples need to be removed from the data.

```{r WGCNA_gsg}
gsg = goodSamplesGenes(datExpr0,
                       minFraction = 2/3,
                       verbose=0)
gsg$allOK
if (!gsg$allOK){
    # Optionally, print the gene and sample names that were removed:
    if (sum(!gsg$goodGenes)>0)
    printFlush(paste("Removing genes:", paste(names(datExpr0)[!gsg$goodGenes],
collapse = ", ")));
    if (sum(!gsg$goodSamples)>0)
    printFlush(paste("Removing samples:",
paste(rownames(datExpr0)[!gsg$goodSamples], collapse = ", ")));
    # Remove the offending genes and samples from the data:
    datExpr0 = datExpr0[gsg$goodSamples, gsg$goodGenes]
}
```

Initial **clustering of the samples** w/o trait information.

```{r WGCNA_cluster_initial, fig.show='hold', message=FALSE}
sampleTree = hclust(dist(datExpr0), method="average")

plotfile = paste(plots_dir, "WGCNA_01_sampleClustering_Initial.wmf", sep=dirsep)
win.metafile(file = plotfile,
      width = 10, height = 15,
      pointsize = 8,
````
````

````
      family = define_font)
plot(sampleTree, main="Sample clustering to detect outliers", sub="",
     xlab="", cex.lab = 1.5, cex.axis = 1.5, cex.main = 2)
dev.off()

plotfile = paste(plots_dir, "WGCNA_01_sampleClustering_Initial.pdf", sep=dirsep)
pdf(file = plotfile,
      width = 10, height = 15,
      pointsize = 8)
plot(sampleTree, main="Sample clustering to detect outliers", sub="",
     xlab="", cex.lab = 1.5, cex.axis = 1.5, cex.main = 2)
dev.off()

plot(sampleTree, main="Sample clustering to detect outliers", sub="",
     xlab="", cex.lab = 1.5, cex.axis = 1.5, cex.main = 1.5)
# Plot a line to show the potential cut
abline(h = height2cut, col = "red");
```

Clustering looks fine.
For now, no Sample is removed (```cutHeight``` set to 300, which will not remove
any sample).
Let's see..

```{r WGCNA_rm_outliers}
clust = cutreeStatic(sampleTree, cutHeight = height2cut , minSize = 0)
table(clust)
keepSamples = (clust==1)
keepSamples
# these samples are not kept
sampleTable[,1][!keepSamples]
datExpr = datExpr0[keepSamples, ]
#dim(datExpr0)
#datExpr = datExpr0
dim(datExpr)
```

1.4 Matching of sample data and expression data
Now match sampleTable and expression data.

```{r match_info_expression}
infoData = sampleTable
infoRows = match(rownames(datExpr), infoData[,1])
datTraits = infoData[infoRows,-c(1)] # take away the colunm with the sample names
rownames(datTraits) = rownames(datExpr) #place the sample names as rownames
datafile = paste(Rdata_dir, "01_datTraits.RData", sep=dirsep)
save(datTraits, file=datafile)
```

Cluster the samples again w/ added trait information

```{r recluster_traitInfo}
sampleTree2 = hclust(dist(datExpr), method="average")
traitColors = numbers2colors(datTraits, signed=TRUE)

plotfile2 = paste(plots_dir, "WGCNA_01_sampleClustering_02_Traits.wmf", sep=dirsep)
win.metafile(file = plotfile2,
      width = 10, height = 10,
      pointsize = 12,
      family = define_font)
plotDendroAndColors(sampleTree2, traitColors, groupLabels=names(datTraits),
                    main="Sample dendrogram and trait heatmap")
dev.off()

plotfile2 = paste(plots_dir, "WGCNA_01_sampleClustering_02_Traits.pdf", sep=dirsep)
pdf(file = plotfile2,
      width = 10, height = 10,
      pointsize = 12)
plotDendroAndColors(sampleTree2, traitColors, groupLabels=names(datTraits),
                    main="Sample dendrogram and trait heatmap")
````

````
dev.off()

plotDendroAndColors(sampleTree2, traitColors, groupLabels=names(datTraits),
                    main="Sample dendrogram and trait heatmap")
```

Remove genes that are not present in current biomaRt annotation (gencode).

```{r removeGenes, eval=FALSE, include=FALSE}
#allGenes = colnames(datExpr)
#mart.current = useMart("ensembl", dataset="mmusculus_gene_ensembl")
#bm.current = getBM(attributes=c("ensembl_gene_id"),
                        #values=allGenes,
                        #mart=mart.current)
#datafile = paste(Rdata_dir, "01_bm.current.RData", sep=dirsep)
#save (file=datafile, bm.current)
#load(datafile)
#dim(bm.current)
#length(allGenes)
#missingGenes = allGenes[! allGenes %in% bm.current$ensembl_gene_id]
#length(missingGenes)
#datafile = paste(Rdata_dir, "01_missingENSids.csv", sep=dirsep)
#write.csv(missingGenes, file=datafile, row.names=F)
# keep a list of all remaining genes
#genes = allGenes[allGenes %in% bm.current$ensembl_gene_id]
#datExprReduced = datExpr[colnames(datExpr) %in% genes]
#dim(datExprReduced)
```

### 1.5 Saving of data that are required for the next steps

Save expr. data for network construction

```{r save_datExpr}
#datExpr = datExprReduced
dim(datExpr)
datafile = paste(Rdata_dir, "01_datExpr.RData", sep=dirsep)
save(datExpr, file=datafile)

filename = paste(Rdata_dir, "dat_used_for_WGCNA.csv", sep = dirsep)
write.csv(file = filename, x = t(datExpr))
```

Define data set dimentions


```{r data_set_dimensions}
exprSize = dim(datExpr)
exprSize
nGenes = exprSize[2]
nGenes
nSamples = exprSize[1]
nSamples
Traits = as.vector(colnames(datTraits))
Traits
```

Save all necessary data for next step - WGCNA 02 Automatic Network Construction

```{r save_data}
datafile = paste(Rdata_dir, "Consensus-dataInput_01.RData", sep=dirsep)
save(datExpr, Traits, nSamples, nGenes, file=datafile)
```
````
````

## Code used | Weighted gene correlation network analysis, part 2

```
---
title: "WGCNA - 2 Automatic Network construction and module detection"
author: "Isabel Karkossa, Alix Aldehoff"
date: "4 May 2017"
---

Please enter your main root directory here. This will be used by all the chunks.

```{r setup, include=FALSE}
knitr::opts_chunk$set(echo = TRUE)
knitr::opts_chunk$set(error = TRUE)
knitr::opts_knit$set(root.dir = "//working-directory") # directory containing this
Rscript
```


# WGCNA Analysis

## 1. Data input

Load the data saved in the first part. Set your desktop path.

```{r load_data, message=FALSE, results='hide'}
dirsep = "/" # the separator for directories on your system
project_dir = getwd()
plots = "Plots" # subdirectory that will hold all plots in .pdf format
plots_dir = paste(project_dir, plots, sep=dirsep) # leave this like it is!
Rdata = "Rdata" # subdirectory that will hold all intermediate .RData files
Rdata_dir = paste(project_dir, Rdata, sep=dirsep) # leave this like it is!
setwd(Rdata_dir) #doesn't change the directory in subsequent chunks
load("Consensus-dataInput_01.RData")
```

```{r define your font}
define_font = "Arial"
```

Load packages. Don't change!

```{r install_packages, include=FALSE, message=FALSE, results='hide'}
list.of.packages = c("WGCNA", "flashClust", "extrafont", "DESeq2")
new.packages = list.of.packages[!(list.of.packages %in%
installed.packages()[,"Package"])]
if(length(new.packages)) install.packages(new.packages)

new.packages = list.of.packages[!(list.of.packages %in%
installed.packages()[,"Package"])]

if(length(new.packages)){

  if (!requireNamespace("BiocManager", quietly = TRUE))
    install.packages("BiocManager")

  for (i in new.packages){
    BiocManager::install(i)
  }
}
```

Load required packages and enable multi-threading. Don't change!

- **WGCNA:** to generate and analyse the network
- **flasClust:** to calculate clusters with high speed
```

````
```{r load_packages}
options(stringsAsFactors = FALSE)

for (i in list.of.packages){
  library(i, character.only = TRUE)
}
```

```{r define font}
#font_import(pattern = "[A/a]rial")
#fonts()
#fonttable()

loadfonts(device = "win")
```

1.1 Create multi expression data set
A multi expression data set is created even if in general the number of sets will
be 1.
Set a label for your data set.
Don't change anything else!

```{r create_multiExpr}
nSets = 1 #number of data set - probably 1
setLabels = "shortlist" #if more then vector of colors e.g. c("female", "male")
multiExpr = vector(mode = "list", length = nSets)

multiExpr[[1]] = list(data = as.data.frame(datExpr));
names(multiExpr[[1]]$data) = names(datExpr);
rownames(multiExpr[[1]]$data) = rownames(datExpr)

# Check that the data has the correct format for many functions operating on
multiple sets:
exprSize = checkSets(multiExpr)
exprSize

datafile = paste(Rdata_dir, "multiExpr.RData", sep=dirsep)
save(multiExpr, file=datafile)
```
1.2 Define Network Construction Parameters

```{r set network construction parameters}
maxBlockSize = 10000 # needs to be adjusted if the computer can't handle that many
genes in one block
  #(16GB workstation should handle up to 20000 probes)

softPower = 17

# We like large modules, so we set the minimum module size relatively high (30):
minModuleSize = 50
maxModuleSize = 500
deepSplit = 2 #default:2

detectCutHeight = 0.995 #dendrogram cut height for module detection, default: 0.995

#define height cut
#a height cut of 0.25, corresponds to correlation of 0.75
MEDissThres = 0.3

#set TOM Type
TOMType = "signed" #signed in case of proteomics data
networkType = "signed" #signed in case of proteomics data

minKMEtoStay = 0.2 # genes whose eigengene connectivity to their module eigengene
is lower are removed from the module.

corType = "pearson"
```

Nothing has to be changed from here! Just run (Knit) the script!
````

```
If you have problems with the modules (e.g. two lists of modules), please adjust
the parameters in the network_construction_module_detection chunk (chapter 3). The
power (soft thresholding power) that es entered in this step has to be adjusted
anyway after running this script the first time. For ideas how to choose this
value, see chapter 3 with the already mentioned chunk.
___________________________________________________________________________________
_______________
This script builds the modules and visualizes the result.
___________________________________________________________________________________
_______________

## 2. One-step network construction and module detection
### 2.1 Choosing the soft-thresholding power: analysis of network topology

Constructing a weighted gene network entails the choice of the soft thresholding
power
to which co-expression similarity is raised to calculate adjacency.  The authors B.
Zhang and S. Horvath (2005, Statistical Applications in Genetics and Molecular
Biology) have proposed to choose the soft thresholding power based on the criterion
of approximate scale-free topology

In the following steps power tables are generated and plotted to visualize the
result and find the optimal soft thresholding power for your experiment.

```{r analysis_network_topology}
powers = c(seq(1,40,by=2)) # Choose a set of soft-thresholding powers
# Initialize a list to hold the results of scale-free analysis
powerTables = vector(mode = "list", length = nSets)

for (set in 1:nSets)
  powerTables[[set]] = list(data = pickSoftThreshold(multiExpr[[set]]$data,
powerVector = powers, verbose=2)[[2]]);

# data: expression data in a matrix or data frame. Rows correspond to samples and
columns to genes
# dataIsExpr: should the data be interpreted as expression (or other numeric) data,
or as a similarity matrix of network nodes?
# RsquaredCut: desired minimum scale free topology fitting index R^2 (default 0.85)
# powerVector: a vector of soft thresholding powers for which the scale free
topology fit indices are to be calculated.
#verbose: integer level of verbosity. Zero means silent, higher values make the
output progressively more and more verbose.

```


Plot the results

```{r plot_topology_results}
colors=c("black") # for nSets=2 e.g. c("black", "red")
plotCols = c(2,5,6,7) # Will plot these columns of the returned scale free analysis
tables
colNames = c("Scale Free Topology Model Fit", "Mean connectivity", "Median
connectivity",
"Max connectivity")
# Get the minima and maxima of the plotted points
ylim = matrix(NA, nrow = 2, ncol = 4)

for (set in 1:nSets){
  for (col in 1:length(plotCols)){
  ylim[1, col] = min(ylim[1, col], powerTables[[set]]$data[,plotCols[col]], na.rm
=TRUE);
  ylim[2, col] = max(ylim[2, col], powerTables[[set]]$data[,plotCols[col]], na.rm
=TRUE)
  }
}

ylim

plotfile = paste(plots_dir, "WGCNA_02_scaleFreeAnalysis.wmf", sep=dirsep)
```
```

```
win.metafile(file = plotfile,
      width = 10, height = 10,
      pointsize = 12,
      family = define_font)
par(mfcol = c(2,2));
par(mar = c(4.2, 4.2, 2.2, 0.5))
cex1 = 0.7

for (col in 1:length(plotCols)) for (set in 1:nSets){
  if (set ==1){
    plot(powerTables[[set]]$data[,1], -
sign(powerTables[[set]]$data[,3])*powerTables[[set]]$data[,2],
       xlab = "Soft Threshold (power)",
       ylab = colNames[col],
       type = "n",
       ylim = ylim[, col],
       main = colNames[col]);
  addGrid();
  }
  if (col == 1){
    text(powerTables[[set]]$data[,1], -
sign(powerTables[[set]]$data[,3])*powerTables[[set]]$data[,2],
         labels = powers,
         cex = cex1,
         col = colors[set]);
  } else
    text(powerTables[[set]]$data[,1], powerTables[[set]]$data[,plotCols[col]],
         labels = powers,
         cex = cex1,
         col = colors[set]);
    if (col == 1){
      legend("bottomright", legend = setLabels, col=colors, pch = 20);
    } else
      legend("topright", legend = setLabels, col = colors, pch = 20);
}
dev.off()

plotfile = paste(plots_dir, "WGCNA_02_scaleFreeAnalysis.pdf", sep=dirsep)
pdf(file = plotfile,
      width = 10, height = 10,
      pointsize = 12)
par(mfcol = c(2,2));
par(mar = c(4.2, 4.2, 2.2, 0.5))
cex1 = 0.7

for (col in 1:length(plotCols)) for (set in 1:nSets){
  if (set ==1){
    plot(powerTables[[set]]$data[,1], -
sign(powerTables[[set]]$data[,3])*powerTables[[set]]$data[,2],
       xlab = "Soft Threshold (power)",
       ylab = colNames[col],
       type = "n",
       ylim = ylim[, col],
       main = colNames[col]);
  addGrid();
  }
  if (col == 1){
    text(powerTables[[set]]$data[,1], -
sign(powerTables[[set]]$data[,3])*powerTables[[set]]$data[,2],
         labels = powers,
         cex = cex1,
         col = colors[set]);
  } else
    text(powerTables[[set]]$data[,1], powerTables[[set]]$data[,plotCols[col]],
         labels = powers,
         cex = cex1,
         col = colors[set]);
    if (col == 1){
      legend("bottomright", legend = setLabels, col=colors, pch = 20);
    } else
```

````
      legend("topright", legend = setLabels, col = colors, pch = 20);
}
dev.off()

for (col in 1:length(plotCols)) for (set in 1:nSets){
  if (set ==1){
    plot(powerTables[[set]]$data[,1], -
sign(powerTables[[set]]$data[,3])*powerTables[[set]]$data[,2],
       xlab = "Soft Threshold (power)",
       ylab = colNames[col],
       type = "n",
       ylim = ylim[, col],
       main = colNames[col]);
  addGrid();
  }
  if (col == 1){
    text(powerTables[[set]]$data[,1], -
sign(powerTables[[set]]$data[,3])*powerTables[[set]]$data[,2],
         labels = powers,
         cex = cex1,
         col = colors[set]);
  } else
    text(powerTables[[set]]$data[,1], powerTables[[set]]$data[,plotCols[col]],
         labels = powers,
         cex = cex1,
         col = colors[set]);
    if (col == 1){
      legend("bottomright", legend = setLabels, col=colors, pch = 20);
    } else
      legend("topright", legend = setLabels, col = colors, pch = 20);
}
datafile = paste(Rdata_dir, "02_multiExpr_Threshold.RData", sep=dirsep)
save(multiExpr, file=datafile)

```
Summary network indices (y-axes) as functions of the soft thresholding power (x-
axes).  Numbers in
the  plots  indicate  the  corresponding  soft  thresholding  powers.   The  plots
indicate  that  approximate  scale-free topology is attained around the soft-
thresholding power of ? (power).  Because the summary connectivity
measures decline steeply with increasing soft-thresholding power, it is
advantageous to choose the lowest power that satisfies the approximate scale-free
topology criterion.

### 2.2 Double-checking the chosen parameters

```{r define TOM}
#We now calculate the adjacencies, using the soft thresholding power

adjacency = adjacency(datExpr, power = softPower, type = networkType) #default
correlation: pearson

#To minimize effects of noise and spurious associations, we transform the adjacency
into Topological Overlap Matrix,
#and calculate the corresponding dissimilarity

TOM = TOMsimilarity(adjacency, TOMType = TOMType)
dissTOM = 1-TOM

TOM_Cytoscape = TOMsimilarityFromExpr(datExpr, power = softPower, TOMType =
TOMType)
```
We now use hierarchical clustering to produce a hierarchical clustering tree
(dendrogram) of genes. Note that we use the function hclust that provides a much
faster hierarchical clustering routine than the standard hclust function.

```{r clustering TOM}
# Call the hierarchical clustering function
geneTree = hclust(as.dist(dissTOM), method = "average");
# Plot the resulting clustering tree (dendrogram)
````

````
plotfile = paste(plots_dir, "WGCNA_02_TOM_Clustering_Genes.wmf", sep=dirsep)
win.metafile(file = plotfile,
      width = 10, height = 8,
      pointsize = 12,
      family = define_font)
plot(geneTree, xlab="", sub="", main = "Gene clustering on TOM-based
dissimilarity",
labels = FALSE, hang = 0.04)
dev.off()
```
In the clustering tree (dendrogram), each leaf, that is a short vertical line,
corresponds to a gene.  Branches of the dendrogram group together densely
interconnected, highly co-expressed genes.  Module identification amounts to the
identification of individual branches (”cutting the branches off the dendrogram”).
There are several methods for branch cutting; our standard method is the Dynamic
Tree Cut from the package dynamicTreeCut.  The next snippet of code illustrates its
use.

```{r double-check dynamic tree cutting}

# Module identification using dynamic tree cut:
dynamicMods = cutreeDynamic(dendro = geneTree, distM = dissTOM, deepSplit =
deepSplit, pamRespectsDendro = FALSE,
                            minClusterSize = minModuleSize,
                            cutHeight = detectCutHeight);
table(dynamicMods)

#The function returned modules labeled largest to smallest.  Label 0 is reserved
for unassigned genes.  The above command lists the sizes of the modules.  We now
plot the module assignment under the gene dendrogram

# Convert numeric lables into colors
dynamicColors = labels2colors(dynamicMods)
table(dynamicColors)
# Plot the dendrogram and colors underneath
plotfile = paste(plots_dir, "WGCNA_02_TOM_InitialClustering_Modules.wmf",
sep=dirsep)
win.metafile(file = plotfile,
      width = 10, height = 8,
      pointsize = 12,
      family = define_font)
plotDendroAndColors(geneTree, dynamicColors, "Dynamic Tree Cut",
dendroLabels = FALSE, hang = 0.03,
addGuide = TRUE, guideHang = 0.05,
main = "Gene dendrogram and module colors")
dev.off()

#Merging of modules whose expression profiles are very similar
#To quantify co-expression similarity of entire modules, we calculate their
eigengenes and cluster them on their correlation
# Calculate eigengenes
MEList = moduleEigengenes(datExpr, colors = dynamicColors)
MEs = MEList$eigengenes
# Calculate dissimilarity of module eigengenes
MEDiss = 1-cor(MEs);
# Cluster module eigengenes
METree = hclust(as.dist(MEDiss), method = "average");

# Plot the result
plotfile = paste(plots_dir, "WGCNA_02_TOM_InitialClustering_ModuleEigengenes.wmf",
sep=dirsep)
win.metafile(file = plotfile,
      width = 10, height = 8,
      pointsize = 12,
      family = define_font)
plot(METree, main = "Clustering of module eigengenes",
xlab = "", sub = "")

# Plot the cut line into the dendrogram
abline(h=MEDissThres, col = "red")
````

````
dev.off()
# Call an automatic merging function
merge = mergeCloseModules(datExpr, dynamicColors, cutHeight = MEDissThres, verbose
= 3)
# The merged module colors
mergedColors = merge$colors;
# Eigengenes of the new merged modules:
mergedMEs = merge$newMEs;

#To see what the merging did to our module colors, we plot the gene dendrogram
again, with the original and merged module colors underneath
plotfile = paste(plots_dir,
"WGCNA_02_TOM_InitialClustering_ModuleEigengenes_Merged.wmf", sep=dirsep)
win.metafile(file = plotfile,
      width = 10, height = 8,
      pointsize = 12,
      family = define_font)
plotDendroAndColors(geneTree, cbind(dynamicColors, mergedColors),
c("Dynamic Tree Cut", "Merged dynamic"),
dendroLabels = FALSE, hang = 0.03,
addGuide = TRUE, guideHang = 0.05)
dev.off()
```


### 3 Network construction and consensus module detection

Choose the power (soft thresholding power) based on the graphs of the previous
step.The Scale Free Topology Model Fit should be >= 0.9 and the mean connectivity
close to 0. On the other hand this power should be as low as possible.
If you obtained multiple lists with modules with the first run of the script, you
should check if you have more proteins than chosen in maxBlockSize. Having multiple
lists will lead to trouble in the following steps.
If too many or too few modules are built, the minModuleSize and the mergeCutHeight
can be adjusted. If the number of modules is appropriate can be concluded from the
consensus gene dendrogram that is built in the next steps.

```{r network_construction_module_detection, message=FALSE, results='hide'}
net = blockwiseConsensusModules( # redefine parameters!!! read help!!!
  multiExpr,
  #checkMissingData = FALSE,
  #replaceMissingAdjacencies = TRUE,
  power = softPower, # soft thresholding power
  maxBlockSize = maxBlockSize, # needs to be adjusted if the computer can't handle
that many genes in one block
  #(16GB workstation should handle up to 20000 probes)
  minModuleSize = minModuleSize, # min module size
  maxModuleSize = maxModuleSize,
  #checkMinModuleSize = TRUE,
  checkMaxModuleSize = TRUE,
  detectCutHeight = detectCutHeight, #dendrogram cut height for module detection,
default: 0.995
  deepSplit = deepSplit, # module detection sensitivity with 0 least and 4 most
sensitive, default:2
  pamRespectsDendro = FALSE,
  mergeCutHeight = MEDissThres, # cut height for merging of modules , default: 0.15
  #modules whose eigengenes are correlated above 1 - 0.15 = 0.85 will be merged
  numericLabels = TRUE, # function return numeric module labels rather than color
labels
  minKMEtoStay = minKMEtoStay, # genes whose eigengene connectivity to their module
eigengene is lower are removed from the module.
  saveTOMs = TRUE, # save the calculated consensus topological overlap
  #saveTOMFileBase = "savedTOMs",
  #verbose = 5, #Zero means silent, higher values make the output progressively
more and more verbose.
  #indent = 0, #Zero means no indentation, each unit adds two spaces.
  networkType = networkType, #signed in case of proteomics data
  TOMtype = TOMType, #signed in case of proteomics data
````

````
  #checkMissingData = TRUE, #checks for excessive numbers of missing data and genes
with zero variance
  corType = corType
)

datafile = paste(Rdata_dir, "02_net.RData", sep=dirsep)
save(net, softPower, TOM_Cytoscape, file=datafile)
```

### 3.1 Results

Have a first quick look at the results
```{r first_results}
names(net)

consMEs = net$multiMEs
moduleLabels = net$colors
moduleColors = labels2colors(moduleLabels)
consTree = net$dendrograms[[1]]

datafile = paste(Rdata_dir, "02_consTree.RData", sep=dirsep)
save(consTree, file=datafile)

datafile = paste(Rdata_dir, "02_moduleColors.RData", sep=dirsep)
save(moduleColors, file=datafile)

datafile = paste(Rdata_dir, "02_moduleLabels.RData", sep=dirsep)
save(moduleLabels, file=datafile)
```

Plot the first results
```{r plot_dendrogram}
# if this doesn't work, run next chunk
plotfile = paste(plots_dir, "WGCNA_02_ConsensusDendrogram_auto.wmf", sep=dirsep)
win.metafile(file = plotfile,
      width = 10, height = 6,
      pointsize = 12,
      family = define_font)
plotDendroAndColors(consTree, moduleColors,
                    "Module colors",
                    dendroLabels = FALSE,
                    hang = 0.03,
                    addGuide = TRUE,
                    guideHang = 0.05,
                    main = "Consensus gene dendrogram and module colors")
dev.off()

plotfile = paste(plots_dir, "WGCNA_02_ConsensusDendrogram_auto.pdf", sep=dirsep)
pdf(file = plotfile,
      width = 10, height = 8,
      pointsize = 12)
plotDendroAndColors(consTree, moduleColors,
                    "Module colors",
                    dendroLabels = FALSE,
                    hang = 0.03,
                    addGuide = TRUE,
                    guideHang = 0.05,
                    main = "Consensus gene dendrogram and module colors")
dev.off()

plotDendroAndColors(consTree, moduleColors,
                    "Module colors",
                    dendroLabels = FALSE,
                    hang = 0.03,
                    addGuide = TRUE,
                    guideHang = 0.05,
                    main = "Consensus gene dendrogram and module colors")

```
````
````

````
```{r plot_blocks, eval=FALSE, include=FALSE}
#If plotting doesn't work because several blocks have been built:
nBlocks = length(net$dendrograms)
# Plot the dendrogram and the module colors underneath for each block
for (block in 1:nBlocks)
  plotDendroAndColors(bnet$dendrograms[[block]],
moduleColors[bnet$blockGenes[[block]]],
                    "Module colors",
                    main = paste("Gene dendrogram and module colors in block",
block),
                    dendroLabels = FALSE, hang = 0.03,
                    addGuide = TRUE, guideHang = 0.05,
                    setLayout = FALSE)
```


Gene dendrogram obtained by clustering the dissimilarity based on consensus
Topological Overlap with
the corresponding module colors indicated by the color row.

Save results for next step: Identification of modules
```{r}
datafile = paste(Rdata_dir, "Consensus-dataInput_02.RData", sep=dirsep)
save(consMEs, moduleLabels, moduleColors, consTree, file = datafile)
```
````

## Code used | Weighted gene correlation network analysis, part 3

```
---
title: "WGCNA - 2 Automatic Network construction and module detection"
author: "Isabel Karkossa, Alix Aldehoff"
date: "4 May 2017"
---
# WGCNA Analysis


Don't change anything in this chunk!
```{r setup, include=FALSE}
knitr::opts_chunk$set(echo = TRUE)
knitr::opts_chunk$set(error = TRUE)
knitr::opts_knit$set(root.dir = "//working-directory") # directory containing this
Rscript
```

## 1. Data input

Load the data that were saved in the first two parts. Set your desktop directory as
you did it previously. Don't change anything else in this chunk!

```{r load_data, message=FALSE, results='hide'}
dirsep = "/" # the separator for directories on your system
project_dir = getwd()
plots = "Plots" # subdirectory that will hold all plots in .pdf format
plots_dir = paste(project_dir, plots, sep=dirsep) # leave this like it is!
Rdata = "Rdata" # subdirectory that will hold all intermediate .RData files
Rdata_dir = paste(project_dir, Rdata, sep=dirsep) # leave this like it is!

sampleTable_file = "Consensus-dataInput_01.RData" # provide your filename
sampleTable_path = paste(Rdata_dir, sampleTable_file, sep=dirsep)
load(sampleTable_path)

sampleTable_file = "Consensus-dataInput_02.RData" # provide your filename
sampleTable_path = paste(Rdata_dir, sampleTable_file, sep=dirsep)
load(sampleTable_path)

sampleTable_file = "multiExpr.Rdata" # provide your filename
sampleTable_path = paste(Rdata_dir, sampleTable_file, sep=dirsep)
load(sampleTable_path)

sampleTable_file = "01_datTraits.Rdata" # provide your filename
sampleTable_path = paste(Rdata_dir, sampleTable_file, sep=dirsep)
load(sampleTable_path)
```

```{r define your font}
define_font = "Arial"
```

```{r define colors}
#define colors for heatmaps with FCs
color_up = ggsci::pal_npg(palette = "nrc")(10)[8]
color_down = ggsci::pal_npg(palette = "nrc")(10)[4]

#define colors for heatmaps with Abundances
color_high = ggsci::pal_npg(palette = "nrc")(10)[9]
color_mid = "white"
color_low = ggsci::pal_npg(palette = "nrc")(10)[3]

#define colors for GS-MM plots
color_medium = ggsci::pal_npg(palette = "nrc")(10)[2]
color_strict = ggsci::pal_npg(palette = "nrc")(10)[3]
```
```

````
barplot(seq(1,10, by = 1), col = ggsci::pal_npg(palette = "nrc")(10))
```

To get a better overview of your results it is helpful to plot results that are
significant. Therefore, choose your thresholds for correlation and p-value in the
next chunk.
```{r set significance thresholds}
#Define a minimum correlation coefficient and a minimum p-value and select for both
values, resp.:
myCor = 0.1
myCor_better = 0.2
myCor_best = 0.3
myPval_low = 0.1
myPval = 0.05
myPval_better = 0.01
myPval_best = 0.001
```

Don't change anything else in this script! Just run (Knit)!
____

This script results in a heatmap that shows you which modules contain proteins that
correllated significantly to the particular treatment!
____

Install necessary packages and load them.

```{r install_packages, include=FALSE, message=FALSE, results='hide'}
list.of.packages = c("WGCNA", "flashClust", "extrafont", "ComplexHeatmap",
"circlize", "dendsort", "dendextend", "plyr")
new.packages = list.of.packages[!(list.of.packages %in%
installed.packages()[,"Package"])]
if(length(new.packages)) install.packages(new.packages)

new.packages = list.of.packages[!(list.of.packages %in%
installed.packages()[,"Package"])]

if(length(new.packages)){

  if (!requireNamespace("BiocManager", quietly = TRUE))
    install.packages("BiocManager")

  for (i in new.packages){
    BiocManager::install(i)
  }
}
```

Load required packages and enable multi-threading.

- **WGCNA:** to generate and analyse the network
- **flasClust:** to calculate clusters with high speed

```{r load_packages}
options(stringsAsFactors = FALSE)

for (i in list.of.packages){
  library(i, character.only = TRUE)
}

#get session info
  sessionInfo()

  # get versions of packages
  for (i in list.of.packages){
    get_version = packageVersion(i)
    print(paste(i, get_version, sep = ": version "))
  }
```
````
````

````
```{r define font}
#font_import(pattern = "[A/a]rial")
#fonts()
#fonttable()

loadfonts(device = "win")
```

```{r load Accession-Gene mapping data}
# the following file has to contain the Accession in the first column and then
columns with Genes and Descriptions for example
sampleTable_file = "genes_INTEGRATED.csv" # provide your filename - don't forget
file type!
sampleTable_path = paste(project_dir, sampleTable_file, sep=dirsep) # leave this
like it is!

sampleGenes = read.csv(sampleTable_path, header = TRUE, row.names = 1)

# sampleGenes = sampleGenes[!duplicated(sampleGenes$Majority.protein.IDs), ]
# row.names(sampleGenes) =sampleGenes[, 1]
# sampleGenes = sampleGenes[, -1]
```


## 1.1 Check multi expression data set

```{r check_multiExpr}

# Check that the data has the correct format for many functions operating on
multiple sets:
exprSize = checkSets(multiExpr)
exprSize

nSets = exprSize$nSets
nSets
```

## 1.2 Create multi expression data set for Traits

Read in the sample table:

```{r read_sampleTable}
traitData = datTraits
head(traitData)

dim(traitData)
names(traitData)

#traitData = traitData[, c(4, 7, 1, 5, 8, 2, 6, 9, 3)]

filename = paste(project_dir,"dat_accession_dataset_mapping.csv", sep = dirsep)
dat_dataset_mapping = read.csv(filename, header = TRUE, row.names = 1)

Traits = vector(mode = "list", length = nSets)

for (set in 1:nSets){
  setSamples = rownames(multiExpr[[set]]$data);
  traitRows = match(setSamples, row.names(traitData));
  Traits[[set]] = list(data = traitData[traitRows, ]);
  rownames(Traits[[set]]$data) = row.names(traitData)[traitRows];
}

datafile = paste(Rdata_dir, "multiTrait.RData", sep=dirsep)
save(Traits, file=datafile)
```

## 2. Relating consensus modules to external microarray sample information

In  this  section  we  illustrate  the  use  of  module  eigengenes  to  relate
consensus  modules  to  external  microarray
````

````
sample information such as classical clinical traits.  In this analysis we have
available several clinical traits.  We
relate the traits to consensus module eigengenes.

```{r module_trait_correlations}
# Set up variables to contain the module-trait correlations
moduleTraitCor = list();
moduleTraitPvalue = list();

# Calculate the correlations and p-values (Fisher)
#For epithelial cell data (NanoToxClass) it made no difference to use Fisher's test
or Student's test
#for (set in 1:nSets){
#moduleTraitCor[[set]] = cor(consMEs[[set]]$data, Traits[[set]]$data, use = "p");
#moduleTraitPvalue[[set]] = corPvalueFisher(moduleTraitCor[[set]],
exprSize$nSamples[set]);
#}

# Calculate the correlations and p-values (Student)
for (set in 1:nSets){
moduleTraitCor[[set]] = cor(consMEs[[set]]$data, Traits[[set]]$data, use = "p",
                            method = "pearson");
moduleTraitPvalue[[set]] = corPvalueStudent(moduleTraitCor[[set]],
exprSize$nSamples[set]);
}

datafile = paste(Rdata_dir, "03_moduleTraitCor.RData", sep=dirsep)
save(moduleTraitCor, file=datafile)

datafile = paste(Rdata_dir, "03_moduleTraitPvalue.RData", sep=dirsep)
save(moduleTraitPvalue, file=datafile)
```


We  now  display  the  module-trait  relationships  using  a  color-coded  table.
We  print  the  correlations  and the corresponding p-values, and color-code the
entris by the p-value signi cance.

```{r plot_correlations}
# Convert numerical lables to colors for labeling of modules in the plot
MEColors = labels2colors(as.numeric(substring(names(consMEs[[1]]$data), 3)));
MEColorNames = paste("ME", MEColors, sep="");

set = 1
textMatrix = paste(signif(moduleTraitCor[[set]], 2), "\n(",
signif(moduleTraitPvalue[[set]], 1), ")", sep = "");
dim(textMatrix) = dim(moduleTraitCor[[set]])
par(mar = c(6, 8.8, 3, 2.2));
setLabels = "scaled" #if more then vector of colors e.g. c("female", "male")

plotfile = paste(plots_dir, "WGCNA_03_ModuleTraitRelationships.wmf", sep=dirsep)
win.metafile(file = plotfile,
      width = 25, height = 10,
      pointsize = 10,
      family = define_font)

# Plot the module-trait relationship table for set number 1

labeledHeatmap(Matrix = moduleTraitCor[[set]],
xLabels = names(Traits[[set]]$data),
yLabels = MEColorNames,
ySymbols = MEColors,
colorLabels = FALSE,
colors = blueWhiteRed(50),
textMatrix = textMatrix,
setStdMargins = TRUE,
cex.text = 0.8,
cex.lab = 1,
zlim = c(-1,1)#,
#main = paste("Module-trait relationships", setLabels[set])
````
````

```
)
dev.off()

par(mar = c(6, 8.8, 3, 2.2));
#setLabels = "scaled" #if more then vector of colors e.g. c("female", "male")

plotfile = paste(plots_dir, "WGCNA_03_ModuleTraitRelationships_all.wmf",
sep=dirsep)
win.metafile(file = plotfile,
      width = (ncol(moduleTraitCor[[set]])/2) +2, height =
(nrow(moduleTraitCor[[set]])/2) +2,
      pointsize = 10,
      family = define_font)

# Plot the module-trait relationship table for set number 1

labeledHeatmap(Matrix = moduleTraitCor[[set]],
xLabels = names(Traits[[set]]$data),
yLabels = MEColorNames,
ySymbols = MEColors,
colorLabels = FALSE,
colors = blueWhiteRed(50),
textMatrix = textMatrix,
setStdMargins = TRUE,
cex.text = 0.8,
cex.lab = 1,
textAdj = c(0.5, 0.6),
zlim = c(-1,1)#,
#main = paste("Module-trait relationships", setLabels[set])
)
dev.off()

clustered_heatmap = moduleTraitCor[[set]]
rownames(clustered_heatmap) = MEColors

dend_rows = dendsort(hclust(dist(clustered_heatmap)))
dend_cols = dendsort(hclust(dist(t(clustered_heatmap))))
dend_cols = color_branches(dend_cols, k=3)

annotation_data_frame = as.data.frame(row.names(clustered_heatmap))
colnames(annotation_data_frame) = c("Module")
rownames(annotation_data_frame) = annotation_data_frame$Module
color_list = setNames(as.vector(annotation_data_frame$Module),
annotation_data_frame$Module)

ha = rowAnnotation(df = annotation_data_frame, col = list(Module = color_list),
show_legend = FALSE)

plotfile = paste(plots_dir, "WGCNA_03_ModuleTraitRelationships_all.wmf",
sep=dirsep)
win.metafile(file = plotfile,
      width = (ncol(clustered_heatmap)/2) +2, height = (nrow(clustered_heatmap)/2)
+2,
      pointsize = 10,
      family = define_font)

ht = Heatmap(clustered_heatmap,
        col=colorRamp2(c(-1,0,1),c(color_down,"white",color_up)),
        color_space = "sRGB",
        na_col="grey",
        cluster_rows = FALSE,
        cluster_columns=FALSE,
        clustering_distance_columns = "euclidean",
        #clustering_method_columns = "complete",
        column_dend_side="top",
        column_dend_height= unit(3,"cm"),
        column_names_side="top",
        show_row_names = "TRUE",
        row_names_side = "left",
        row_names_max_width = unit(2, "cm"),
```

````
        row_names_gp = gpar(cex = 1, fontface = "bold"),
        column_names_max_height = unit(5, "cm"),
        show_row_dend = FALSE,
        show_column_dend = TRUE,
        #row_title_gp = gpar(cex = 0.6),
        column_names_gp = gpar(cex = 1, fontface = "bold"),
        cell_fun = function(j, i, x, y, width, height, fill){
          grid.text(textMatrix[i, j], x, y, gp = gpar(cex = 0.8))},
        #column_dend_reorder=c(1,3,4,5,2),
        #row_dend_reorder=F,
        heatmap_legend_param = list(title = "Correlation",
                                    title_gp = gpar(cex = 1, fontface = "bold"),
                                    color_bar = c("continuous")),
        show_heatmap_legend=TRUE
)

ht + ha
dev.off()

plotfile = paste(plots_dir, "WGCNA_03_ModuleTraitRelationships_all.pdf",
sep=dirsep)
pdf(file = plotfile,
      width = (ncol(clustered_heatmap)/2) +2, height = (nrow(clustered_heatmap)/2)
+2,
      pointsize = 10)

ht + ha
dev.off()

plotfile = paste(plots_dir, "WGCNA_03_ModuleTraitRelationships_all.png",
sep=dirsep)
png(file = plotfile,
    units = "in",
    res = 800,
      width = (ncol(clustered_heatmap)/2) +2, height = (nrow(clustered_heatmap)/2)
+2,
      pointsize = 10,
      family = define_font)

ht + ha
dev.off()
```
````

Shows relationships of consensus module eigengenes and clinical traits. Each row in the table corresponds to a consensus module, and each column to a trait. Numbers in the table report the correlations of the corresponding module eigengenes and traits, with the p-values printed below the correlations in parentheses.
The table is color coded by correlation according to the color legend.

Several datasets (nSets) can be combined into one heatmap if necessary! But therefore this script has to be adjusted!

2. Exporting results of the network analysis

We now put together a data frame that summarizes the results of network analysis, namely the gene significances (GS) and module memberships (also known as kME) of all probes. Loading the Gene Annotation Table is not necessary because Uniprot Accessions can be used for GO Annotation analysis.
Here, all the modules are chosen as intModules to get an overview.

````
```{r save_module_accessions}

allLLIDs = names(multiExpr[[1]]$data) # get IDs - for genes LocusLinkIDs necessary
intModules = MEColors # Choose interesting modules (c("brown", "blue")) or all
(MEColors)

setwd(Rdata_dir)

#save Accessions for interesting modules
````

````
for (module in intModules){
  modGenes = (moduleColors == module) # Select module probes
  modLLIDs = allLLIDs[modGenes] # Get their entrez ID codes
  # Write them into a file
  fileName = paste("UniprotAccessions-", module, ".txt", sep="")
  write.table(as.data.frame(modLLIDs), file = fileName, row.names = FALSE,
col.names = FALSE)

  dat_to_export = as.data.frame(modLLIDs)
  colnames(dat_to_export) = c("Accessions")
  dat_to_export = merge(dat_to_export, sampleGenes, all.x = TRUE, by.x =
"Accessions", by.y = "row.names")

  fileName = paste("UniprotAccessions-Genes-", module, ".csv", sep="")
  pathname = paste(Rdata_dir, fileName, sep = dirsep)
  write.csv(dat_to_export, file = pathname, row.names = FALSE)
}

accession_module_df = as.data.frame(cbind(allLLIDs, moduleColors))
colnames(accession_module_df) = c("Accession", "Module")

fileName = paste("UniprotAccessions-Modules", ".csv", sep="")
  pathname = paste(Rdata_dir, fileName, sep = dirsep)
  write.csv(accession_module_df, file = pathname, row.names = FALSE)

# As background in the enrichment analysis, we will use all probes in the analysis.
#fileName = paste("UniprotAccessions-all.txt", sep="");
#write.table(as.data.frame(allLLIDs), file = fileName,
#row.names = FALSE, col.names = FALSE)
```

txt files for all the interesting modules have been generated. These files contain
the uniprot accession numbers.

Finally we put together the full information data frame and write it into a plain
text CSV file that can be read by standard spreadsheet programs.  Note that the
probes are not sorted in any particular way; many sort orders are possible and we
leave it to the reader to either modify the code or to perform the sort in a
spreadsheet software.

```{r save_data}
probes = names(multiExpr[[1]]$data)
info = data.frame(EntrezID = allLLIDs,
                  ModuleLabel = moduleLabels,
                  ModuleColor = labels2colors(moduleLabels))

head(info)

datafile = paste(Rdata_dir, "consensusAnalysis-CombinedNetworkResults.csv",
sep=dirsep)
write.csv(info, file=datafile, row.names=FALSE, quote = FALSE)
```

Export numbers of Proteins per module
```{r export_nCounts}

counts = as.data.frame(table(info$ModuleColor))
names(counts) = c("ModuleColors", "nCounts")
counts

datafile = paste(Rdata_dir, "consensusAnalysis-ModuleContentCount.csv", sep=dirsep)
write.csv(counts, file=datafile, row.names=FALSE, quote = FALSE)

counts_dataset = as.data.frame(cbind(info$EntrezID, info$ModuleColor))
colnames(counts_dataset) = c("Analyte", "ModuleColor")
counts_dataset_match = merge(counts_dataset, dat_dataset_mapping, by.x = "Analyte",
by.y = "row.names")

dataset_vector = unique(counts_dataset_match$Dataset)
````

```
out = plyr::count(counts_dataset_match[which(counts_dataset_match$Dataset ==
dataset_vector[1]), ], vars = "ModuleColor")
colnames(out) = c("ModuleColor", dataset_vector[1])

for (i in 2:length(dataset_vector)){
  get_dat = plyr::count(counts_dataset_match[which(counts_dataset_match$Dataset ==
dataset_vector[i]), ],
                        vars = "ModuleColor")
colnames(get_dat) = c("ModuleColor", dataset_vector[i])

merge_dat = merge(out, get_dat, by = "ModuleColor", all.x = TRUE)

out = merge_dat
}

counts_dataset_summary = out

datafile = paste(Rdata_dir, "consensusAnalysis-ModuleContentCount-datasets.csv",
sep=dirsep)
write.csv(counts_dataset_summary, file=datafile, row.names=FALSE, quote = FALSE)

plot_dataset_summary = counts_dataset_summary[, -1]
rownames(plot_dataset_summary) = counts_dataset_summary$ModuleColor
barplot(as.matrix(t(plot_dataset_summary)), las = 2, legend =
colnames(plot_dataset_summary))
```


Save data for next steps: trait dependent identification of genes with high GS and
MM

```{r save_Data}
datafile = paste(Rdata_dir, "Consensus-dataInput_03.RData", sep=dirsep)
save(datExpr, Traits, datTraits, nSamples, nGenes, consMEs, moduleLabels,
moduleColors, consTree, intModules, allLLIDs, file=datafile)
```


3 Simplify results

3.1 Identify significant correlations

```{r select significant correlation results}
#Define a minimum correlation coefficient and a minimum p-value and select for both
values, resp.:

sigCors  = abs(as.data.frame(signif(moduleTraitCor[[set]], 2))) >= myCor
sigCors_better  = abs(as.data.frame(signif(moduleTraitCor[[set]], 2))) >=
myCor_better
sigCors_best  = abs(as.data.frame(signif(moduleTraitCor[[set]], 2))) >= myCor_best
sigPvals_low = abs(as.data.frame(signif(moduleTraitPvalue[[set]], 1))) <=
myPval_low
sigPvals = abs(as.data.frame(signif(moduleTraitPvalue[[set]], 1))) <= myPval
sigPvals_better = abs(as.data.frame(signif(moduleTraitPvalue[[set]], 1))) <=
myPval_better
sigPvals_best = abs(as.data.frame(signif(moduleTraitPvalue[[set]], 1))) <=
myPval_best
```

```{r visualize significant correlations}

# will display only significant correlations

textMatrix = rep("", dim(moduleTraitCor[[set]])[1]*dim(moduleTraitCor[[set]])[2])

  textMatrix[sigCors == 1 & sigPvals] = paste(round(moduleTraitCor[[set]][sigCors
== 1 & sigPvals], 2),
                                              "\n", "*", sep = "")
  textMatrix[sigCors == 1 & sigPvals_better] =
paste(round(moduleTraitCor[[set]][sigCors == 1 & sigPvals_better], 2),
```

```
                                                    "\n", "**", sep = "")
  textMatrix[sigCors == 1 & sigPvals_best] =
paste(round(moduleTraitCor[[set]][sigCors == 1 & sigPvals_best], 2),
                                                  "\n", "***", sep = "")

dim(textMatrix) = dim(moduleTraitCor[[set]])

par(mar = c(6, 8.8, 3, 2.2));
#setLabels = "scaled" #if more then vector of colors e.g. c("female", "male")

plotfile = paste(plots_dir, "WGCNA_03_ModuleTraitRelationships_sign.wmf",
sep=dirsep)
win.metafile(file = plotfile,
      width = (ncol(moduleTraitCor[[set]])/2) +2, height =
(nrow(moduleTraitCor[[set]])/2) +2,
      pointsize = 10,
      family = define_font)

# Plot the module-trait relationship table for set number 1

labeledHeatmap(Matrix = moduleTraitCor[[set]],
xLabels = names(Traits[[set]]$data),
yLabels = MEColorNames,
ySymbols = MEColors,
colorLabels = FALSE,
colors = blueWhiteRed(50),
textMatrix = textMatrix,
setStdMargins = TRUE,
cex.text = 0.8,
cex.lab = 1,
textAdj = c(0.5, 0.6),
zlim = c(-1,1)#,
#main = paste("Module-trait relationships", setLabels[set])
)
dev.off()

clustered_heatmap = moduleTraitCor[[set]]
rownames(clustered_heatmap) = MEColors

dend_rows = dendsort(hclust(dist(clustered_heatmap)))
dend_cols = dendsort(hclust(dist(t(clustered_heatmap))))
dend_cols = color_branches(dend_cols, k=3)

annotation_data_frame = as.data.frame(row.names(clustered_heatmap))
colnames(annotation_data_frame) = c("Module")
rownames(annotation_data_frame) = annotation_data_frame$Module
color_list = setNames(as.vector(annotation_data_frame$Module),
annotation_data_frame$Module)

ha = rowAnnotation(df = annotation_data_frame, col = list(Module = color_list),
show_legend = FALSE)

plotfile = paste(plots_dir, "WGCNA_03_ModuleTraitRelationships_sign_clustered.wmf",
sep=dirsep)
win.metafile(file = plotfile,
      width = (ncol(clustered_heatmap)/2) +2, height = (nrow(clustered_heatmap)/2)
+2,
      pointsize = 10,
      family = define_font)

ht = Heatmap(clustered_heatmap,
        col=colorRamp2(c(-1,0,1),c(color_down,"white",color_up)),
        color_space = "sRGB",
        na_col="grey",
        cluster_rows = TRUE,
        cluster_columns=FALSE,
        clustering_distance_columns = "euclidean",
        #clustering_method_columns = "complete",
        column_dend_side="top",
        column_dend_height= unit(3,"cm"),
```

```
        column_names_side="top",
        show_row_names = "TRUE",
        row_names_side = "left",
        row_names_max_width = unit(2, "cm"),
        row_names_gp = gpar(cex = 1, fontface = "bold"),
        column_names_max_height = unit(5, "cm"),
        show_row_dend = FALSE,
        show_column_dend = TRUE,
        #row_title_gp = gpar(cex = 0.6),
        column_names_gp = gpar(cex = 1, fontface = "bold"),
        cell_fun = function(j, i, x, y, width, height, fill){
          grid.text(textMatrix[i, j], x, y, gp = gpar(cex = 0.8))},
        #column_dend_reorder=c(1,3,4,5,2),
        #row_dend_reorder=F,
        heatmap_legend_param = list(title = "Correlation",
                                    title_gp = gpar(cex = 1, fontface = "bold"),
                                    color_bar = c("continuous")),
        show_heatmap_legend=TRUE
        )

ht + ha
dev.off()

plotfile = paste(plots_dir, "WGCNA_03_ModuleTraitRelationships_sign_clustered.pdf",
sep=dirsep)
pdf(file = plotfile,
      width = (ncol(clustered_heatmap)/2) +2, height = (nrow(clustered_heatmap)/2)
+2,
      pointsize = 10)

ht + ha
dev.off()

plotfile = paste(plots_dir, "WGCNA_03_ModuleTraitRelationships_sign_clustered.png",
sep=dirsep)
png(file = plotfile,
    units = "in",
    res = 800,
      width = (ncol(clustered_heatmap)/2) +2, height = (nrow(clustered_heatmap)/2)
+2,
      pointsize = 10,
      family = define_font)

ht + ha
dev.off()

#export data
clustered_heatmap = as.data.frame(clustered_heatmap)
row.names(clustered_heatmap)

textMatrix = as.data.frame(textMatrix)
row.names(textMatrix) = row.names(clustered_heatmap)
colnames(textMatrix) = paste(colnames(clustered_heatmap), "label", sep = "_")

clustered_heatmap$Order = dend_rows$order

merge_dat = merge(clustered_heatmap, textMatrix, by = "row.names")
row.names(merge_dat) = merge_dat$Row.names
merge_dat = merge_dat[, -1]

filename = "WGCNA_03_ModuleTraitRelationships_sign.csv"
pathname = paste(Rdata_dir, filename, sep = dirsep)

write.csv(x = merge_dat, file = pathname)

#complete information
export_dat = as.data.frame(moduleTraitPvalue[[1]])
colnames(export_dat) = paste(colnames(export_dat), "pvalue", sep = "_")
rownames(export_dat) = MEColorNames
```

````
export_cor = as.data.frame(moduleTraitCor[[1]])
rownames(export_cor) = MEColorNames

merge_dat = merge(export_cor, export_dat, by = "row.names")

filename = "WGCNA_03_Module-Trait-Correlation.csv"
pathname = paste(Rdata_dir, filename, sep = dirsep)
write.csv(x = merge_dat, file = pathname, row.names = FALSE)
```

```{r visualize significant correlations - low threshold}

# will display only significant correlations

textMatrix = rep("", dim(moduleTraitCor[[set]])[1]*dim(moduleTraitCor[[set]])[2])

textMatrix[sigCors == 1 & sigPvals_low] = paste(round(moduleTraitCor[[set]][sigCors
== 1 & sigPvals_low], 2),
                                              "\n", "#", sep = "")

  textMatrix[sigCors == 1 & sigPvals] = paste(round(moduleTraitCor[[set]][sigCors
== 1 & sigPvals], 2),
                                              "\n", "*", sep = "")
  textMatrix[sigCors == 1 & sigPvals_better] =
paste(round(moduleTraitCor[[set]][sigCors == 1 & sigPvals_better], 2),
                                                     "\n", "**", sep = "")
  textMatrix[sigCors == 1 & sigPvals_best] =
paste(round(moduleTraitCor[[set]][sigCors == 1 & sigPvals_best], 2),
                                                   "\n", "***", sep = "")

dim(textMatrix) = dim(moduleTraitCor[[set]])

par(mar = c(6, 8.8, 3, 2.2));
#setLabels = "scaled" #if more then vector of colors e.g. c("female", "male")

plotfile = paste(plots_dir, "WGCNA_03_ModuleTraitRelationships_signlow.wmf",
sep=dirsep)
win.metafile(file = plotfile,
      width = (ncol(moduleTraitCor[[set]])/2) +2, height =
(nrow(moduleTraitCor[[set]])/2) +2,
      pointsize = 10,
      family = define_font)

# Plot the module-trait relationship table for set number 1

labeledHeatmap(Matrix = moduleTraitCor[[set]],
xLabels = names(Traits[[set]]$data),
yLabels = MEColorNames,
ySymbols = MEColors,
colorLabels = FALSE,
colors = blueWhiteRed(50),
textMatrix = textMatrix,
setStdMargins = TRUE,
cex.text = 0.8,
cex.lab = 1,
textAdj = c(0.5, 0.6),
zlim = c(-1,1)#,
#main = paste("Module-trait relationships", setLabels[set])
)
dev.off()

clustered_heatmap = moduleTraitCor[[set]]
rownames(clustered_heatmap) = MEColors

dend_rows = dendsort(hclust(dist(clustered_heatmap)))
dend_cols = dendsort(hclust(dist(t(clustered_heatmap))))
dend_cols = color_branches(dend_cols, k=3)

annotation_data_frame = as.data.frame(row.names(clustered_heatmap))
colnames(annotation_data_frame) = c("Module")
````

```
rownames(annotation_data_frame) = annotation_data_frame$Module
color_list = setNames(as.vector(annotation_data_frame$Module),
annotation_data_frame$Module)

ha = rowAnnotation(df = annotation_data_frame, col = list(Module = color_list),
show_legend = FALSE)

plotfile = paste(plots_dir,
"WGCNA_03_ModuleTraitRelationships_signlow_clustered.wmf", sep=dirsep)
win.metafile(file = plotfile,
      width = (ncol(clustered_heatmap)/2) +2, height = (nrow(clustered_heatmap)/2)
+2,
      pointsize = 10,
      family = define_font)

ht = Heatmap(clustered_heatmap,
        col=colorRamp2(c(-1,0,1),c(color_down,"white",color_up)),
        color_space = "sRGB",
        na_col="grey",
        cluster_rows = FALSE,
        cluster_columns=FALSE,
        clustering_distance_columns = "euclidean",
        #clustering_method_columns = "complete",
        column_dend_side="top",
        column_dend_height= unit(3,"cm"),
        column_names_side="top",
        show_row_names = "TRUE",
        row_names_side = "left",
        row_names_max_width = unit(2, "cm"),
        row_names_gp = gpar(cex = 1, fontface = "bold"),
        column_names_max_height = unit(5, "cm"),
        show_row_dend = FALSE,
        show_column_dend = TRUE,
        #row_title_gp = gpar(cex = 0.6),
        column_names_gp = gpar(cex = 1, fontface = "bold"),
        cell_fun = function(j, i, x, y, width, height, fill){
          grid.text(textMatrix[i, j], x, y, gp = gpar(cex = 0.8))},
        #column_dend_reorder=c(1,3,4,5,2),
        #row_dend_reorder=F,
        heatmap_legend_param = list(title = "Correlation",
                                    title_gp = gpar(cex = 1, fontface = "bold"),
                                    color_bar = c("continuous")),
        show_heatmap_legend=TRUE
        )

ht + ha
dev.off()

plotfile = paste(plots_dir,
"WGCNA_03_ModuleTraitRelationships_signlow_clustered.pdf", sep=dirsep)
pdf(file = plotfile,
      width = (ncol(clustered_heatmap)/2) +2, height = (nrow(clustered_heatmap)/2)
+2,
      pointsize = 10)

ht + ha
dev.off()

plotfile = paste(plots_dir,
"WGCNA_03_ModuleTraitRelationships_signlow_clustered.png", sep=dirsep)
png(file = plotfile,
    units = "in",
    res = 800,
      width = (ncol(clustered_heatmap)/2) +2, height = (nrow(clustered_heatmap)/2)
+2,
      pointsize = 10,
      family = define_font)

ht + ha
dev.off()
```

````
```

### 3.2 Identify interesting modules

```{r select modules with high correlations}
# multiply matrices to get interesting modules
sigCombined = sigCors*sigPvals

# our trait of interest - for me all treaits are interesting!!

int.trait = colnames(datTraits)

for (i in int.trait){
  tryCatch({
# which modules (indices) are those of interest
int.modules.indices <- as.vector(which(sigCombined[, i] == 1))
# which modules (names) are those of interest
int.modules.names <- moduleColors[int.modules.indices]

#plot interesting modules for traits

showValues = 0 # set to 0 if you want to see '*' signs in the module trait
relationships heatmap, instead of cor and p-values (-> 0)
if(showValues == 1) {
  # Will display correlations and their p-values (rounded to 2 digits)
  textMatrix =  paste0(signif(moduleTraitCor[[set]], 2), "\n(",
signif(moduleTraitPvalue[[set]], 1), ")");
  myCexText = 1
  myTextAdj = c(0.5, 0.5)
} else {
  # will display only significant correlations

  textMatrix = rep("", dim(moduleTraitCor[[set]])[1]*dim(moduleTraitCor[[set]])[2])
  textMatrix[sigCors == 1 & sigPvals] = paste(round(moduleTraitCor[[set]][sigCors
== 1 & sigPvals], 2),
                                              "\n(",

signif(moduleTraitPvalue[[set]][sigCors == 1 & sigPvals], 1),
                                              ")", sep = "")
  myCexText = 0.8
  myTextAdj = c(0.5, 0.4)
}

dim(textMatrix) = dim(moduleTraitCor[[set]])

filename = paste("WGCNA_03_ModuleTraitRelationships_int_", i, ".wmf", sep = "")
plotfile = paste(plots_dir, filename, sep=dirsep)
win.metafile(file = plotfile,
      width = (ncol(moduleTraitCor[[set]][int.modules.indices, ])/2) +2,
      height = (nrow(moduleTraitCor[[set]][int.modules.indices, ])/2) +2,
      pointsize = 10,
      family = define_font)

# Plot the module-trait relationships


labeledHeatmap(Matrix = moduleTraitCor[[set]][int.modules.indices, ],
xLabels = names(Traits[[set]]$data),
yLabels = MEColorNames[int.modules.indices],
ySymbols = MEColors[int.modules.indices],
colorLabels = FALSE,
colors = blueWhiteRed(50),
textMatrix = textMatrix[int.modules.indices, ],
setStdMargins = TRUE,
cex.text = myCexText,
cex.lab = 1,
textAdj = myTextAdj,
zlim = c(-1,1)#,
#main = paste("Module-trait relationships", setLabels[set])
)
````

````
dev.off()

# filename = paste("WGCNA_03_ModuleTraitRelationships_int_", i, ".pdf", sep = "")
# plotfile = paste(plots_dir, filename, sep=dirsep)
# pdf(file = plotfile,
#       width = 10, height = 3,
#       pointsize = 10,
#       family = define_font)
#
# # Plot the module-trait relationship table for set number 1
#
# labeledHeatmap(Matrix = moduleTraitCor[[set]][int.modules.indices, ],
# xLabels = names(Traits[[set]]$data),
# yLabels = MEColorNames[int.modules.indices],
# ySymbols = MEColors[int.modules.indices],
# colorLabels = FALSE,
# colors = blueWhiteRed(50),
# textMatrix = textMatrix[int.modules.indices, ],
# setStdMargins = TRUE,
# cex.text = myCexText,
# cex.lab = 2,
# textAdj = myTextAdj,
# zlim = c(-1,1)#,
# #main = paste("Module-trait relationships", setLabels[set])
# )
# dev.off()

}, error = function(e){cat("ERROR:", conditionMessage(e), i, "\n")})
}
```

### 3.3 Plot Sample Description Matrix

```{r plot Sample Description Matrix}
cor_dat = traitData
cor_result = corAndPvalue(as.matrix(cor_dat), use = "pairwise.complete.obs", method
= "pearson",
                          alternative = "two.sided")

sigCors  = abs(as.data.frame(signif(cor_result$cor, 2))) >= myCor
sigCors_better  = abs(as.data.frame(signif(cor_result$cor, 2))) >= myCor_better
sigCors_best  = abs(as.data.frame(signif(cor_result$cor, 2))) >= myCor_best
sigPvals = abs(as.data.frame(signif(cor_result$p, 1))) <= myPval
sigPvals_better = abs(as.data.frame(signif(cor_result$p, 1))) <= myPval_better
sigPvals_best = abs(as.data.frame(signif(cor_result$p, 1))) <= myPval_best

# textMatrix = rep("", dim(cor_result$cor)[1]*dim(cor_result$cor)[2])
#
#
#   textMatrix[sigCors == 1 & sigPvals] = paste(round(cor_result$cor[sigCors == 1 &
sigPvals], 2),
#                                                "\n", "*", sep = "")
#   textMatrix[sigCors == 1 & sigPvals_better] = paste(round(cor_result$cor[sigCors
== 1 & sigPvals_better], 2),
#                                                       "\n", "**", sep = "")
#   textMatrix[sigCors == 1 & sigPvals_best] = paste(round(cor_result$cor[sigCors
== 1 & sigPvals_best], 2),
#                                                     "\n", "***", sep = "")
#
# dim(textMatrix) = dim(cor_result$cor)


plotfile = paste(plots_dir, "WGCNA_03_SampleDescriptionMatrix_Cor_sign.wmf",
sep=dirsep)
win.metafile(file = plotfile,
      width = (ncol(cor_result$cor)/3) +3,
      height = (nrow(cor_result$cor)/3) +2,
      pointsize = 10,
      family = define_font)
````

```
# Plot the module-trait relationship table for set number 1

labeledHeatmap(Matrix = cor_result$cor,
xLabels = rownames(cor_result$cor),
xLabelsAngle = 90,
yLabels = rownames(cor_result$cor),
#ySymbols = MEColors,
colorLabels = FALSE,
colors = blueWhiteRed(50),
#textMatrix = textMatrix,
setStdMargins = TRUE,
cex.text = 0.8,
cex.lab = 1,
textAdj = c(0.5, 0.6),
zlim = c(-1,1)#,
#main = paste("Module-trait relationships", setLabels[set])
)
dev.off()

clustered_heatmap = cor_result$cor

dend_rows = dendsort(hclust(dist(clustered_heatmap)))
dend_rows = color_branches(dend_rows, k=3)
dend_cols = dendsort(hclust(dist(t(clustered_heatmap))))
dend_cols = color_branches(dend_cols, k=3)

plotfile = paste(plots_dir, "WGCNA_03_SampleDescriptionMatrix_Cor_clust_sign.wmf",
sep=dirsep)
win.metafile(file = plotfile,
      width = (ncol(cor_result$cor)/3) +3,
      height = (nrow(cor_result$cor)/3) +2,
      pointsize = 10,
      family = define_font)

ht = Heatmap(cor_result$cor,
        col=colorRamp2(c(-1,0,1),c(color_down,"white",color_up)),
        color_space = "sRGB",
        na_col="grey",
        cluster_rows = dend_rows,
        cluster_columns = dend_cols,
        clustering_distance_columns = "euclidean",
        #clustering_method_columns = "complete",
        column_dend_side="top",
        column_dend_height= unit(3,"cm"),
        row_dend_width = unit(3, "cm"),
        column_names_side="top",
        show_row_names = "TRUE",
        row_names_side = "left",
        row_names_max_width = unit(5, "cm"),
        row_names_gp = gpar(cex = 1, fontface = "bold"),
        column_names_max_height = unit(5, "cm"),
        show_row_dend = TRUE,
        show_column_dend = TRUE,
        #row_title_gp = gpar(cex = 0.6),
        column_names_gp = gpar(cex = 1, fontface = "bold"),
        #cell_fun = function(j, i, x, y, width, height, fill){
          #grid.text(textMatrix[i, j], x, y, gp = gpar(cex = 0.8))},
        #column_dend_reorder=c(1,3,4,5,2),
        #row_dend_reorder=F,
        heatmap_legend_param = list(title = "Correlation",
                                    title_gp = gpar(cex = 1, fontface = "bold"),
                                    color_bar = c("continuous")),
        show_heatmap_legend=TRUE
        )

print(ht)

dev.off()
```

````
plotfile = paste(plots_dir, "WGCNA_03_SampleDescriptionMatrix_Cor_clust_sign.pdf",
sep=dirsep)
pdf(file = plotfile,
      width = (ncol(cor_result$cor)/3) +3,
      height = (nrow(cor_result$cor)/3) +2,
      pointsize = 10)

print(ht)

dev.off()

plotfile = paste(plots_dir, "WGCNA_03_SampleDescriptionMatrix_Cor_clust_sign.png",
sep=dirsep)
png(file = plotfile,
    units = "in",
    res = 800,
      width = (ncol(cor_result$cor)/3) +3,
      height = (nrow(cor_result$cor)/3) +2,
      pointsize = 10,
      family = define_font)

print(ht)

dev.off()


```

### 3.4 Plot Correlation of Module-Trait-Matrix

```{r plot correlation of module-trait-matrix}
cor_dat = moduleTraitCor[[set]]
cor_result = corAndPvalue(as.matrix(cor_dat), use = "pairwise.complete.obs", method
= "pearson",
                          alternative = "two.sided")

sigCors  = abs(as.data.frame(signif(cor_result$cor, 2))) >= myCor
sigCors_better  = abs(as.data.frame(signif(cor_result$cor, 2))) >= myCor_better
sigCors_best  = abs(as.data.frame(signif(cor_result$cor, 2))) >= myCor_best
sigPvals = abs(as.data.frame(signif(cor_result$p, 1))) <= myPval
sigPvals_better = abs(as.data.frame(signif(cor_result$p, 1))) <= myPval_better
sigPvals_best = abs(as.data.frame(signif(cor_result$p, 1))) <= myPval_best

# textMatrix = rep("", dim(cor_result$cor)[1]*dim(cor_result$cor)[2])
#
#
#   textMatrix[sigCors == 1 & sigPvals] = paste(round(cor_result$cor[sigCors == 1 &
sigPvals], 2),
#                                                "\n", "*", sep = "")
#   textMatrix[sigCors == 1 & sigPvals_better] = paste(round(cor_result$cor[sigCors
== 1 & sigPvals_better], 2),
#                                                       "\n", "**", sep = "")
#   textMatrix[sigCors == 1 & sigPvals_best] = paste(round(cor_result$cor[sigCors
== 1 & sigPvals_best], 2),
#                                                     "\n", "***", sep = "")
#
# dim(textMatrix) = dim(cor_result$cor)


clustered_heatmap = cor_result$cor

dend_rows = dendsort(hclust(dist(clustered_heatmap)))
dend_rows = color_branches(dend_rows, k=3)
dend_cols = dendsort(hclust(dist(t(clustered_heatmap))))
dend_cols = color_branches(dend_cols, k=3)

plotfile = paste(plots_dir, "WGCNA_03_ModuleTraitRelationships_Correlation.wmf",
sep=dirsep)
win.metafile(file = plotfile,
      width = (ncol(cor_result$cor)/5) +3,
````

```
      height = (nrow(cor_result$cor)/5) +2,
      pointsize = 10,
      family = define_font)

ht = Heatmap(cor_result$cor,
        col=colorRamp2(c(-1,0,1),c(color_down,"white",color_up)),
        color_space = "sRGB",
        na_col="grey",
        cluster_rows = dend_rows,
        cluster_columns = dend_cols,
        clustering_distance_columns = "euclidean",
        #clustering_method_columns = "complete",
        column_dend_side="top",
        column_dend_height= unit(3,"cm"),
        row_dend_width = unit(3, "cm"),
        column_names_side="top",
        show_row_names = "TRUE",
        row_names_side = "left",
        row_names_max_width = unit(5, "cm"),
        row_names_gp = gpar(cex = 1, fontface = "bold"),
        column_names_max_height = unit(5, "cm"),
        show_row_dend = TRUE,
        show_column_dend = TRUE,
        #row_title_gp = gpar(cex = 0.6),
        column_names_gp = gpar(cex = 1, fontface = "bold"),
        #cell_fun = function(j, i, x, y, width, height, fill){
          #grid.text(textMatrix[i, j], x, y, gp = gpar(cex = 0.8))},
        #column_dend_reorder=c(1,3,4,5,2),
        #row_dend_reorder=F,
        heatmap_legend_param = list(title = "Correlation",
                                    title_gp = gpar(cex = 1, fontface = "bold"),
                                    color_bar = c("continuous")),
        show_heatmap_legend=TRUE
        )

print(ht)

dev.off()


plotfile = paste(plots_dir, "WGCNA_03_ModuleTraitRelationships_Correlation.pdf",
sep=dirsep)
pdf(file = plotfile,
      width = (ncol(cor_result$cor)/5) +3,
      height = (nrow(cor_result$cor)/5) +2,
      pointsize = 10)

print(ht)

dev.off()

plotfile = paste(plots_dir, "WGCNA_03_ModuleTraitRelationships_Correlation.png",
sep=dirsep)
png(file = plotfile,
    units = "in",
    res = 800,
      width = (ncol(cor_result$cor)/5) +3,
      height = (nrow(cor_result$cor)/5) +2,
      pointsize = 10,
      family = define_font)

print(ht)

dev.off()
```

```
---
title: "WGCNA - 4 - GS/MM Correlation"
author: "Isabel Karkossa, Alix Aldehoff"
date: "May 11, 2017"
---

```{r setup, include=FALSE}
knitr::opts_chunk$set(echo = TRUE)
knitr::opts_chunk$set(error = TRUE)
knitr::opts_knit$set(root.dir = "//working-directory") # directory containing this
Rscript
```


WGCNA Analysis

1. Data input

Load the data that were saved in the first two parts. Set your desktop directory as
you did it previously. Don't change anything else in this chunk!

```{r load_data, message=FALSE, results='hide'}
dirsep = "/" # the separator for directories on your system
project_dir = getwd()
plots = "Plots" # subdirectory that will hold all plots in .pdf format
plots_dir = paste(project_dir, plots, sep=dirsep) # leave this like it is!
Rdata = "Rdata" # subdirectory that will hold all intermediate .RData files
Rdata_dir = paste(project_dir, Rdata, sep=dirsep) # leave this like it is!
setwd(Rdata_dir) #doesn't change the directory in subsequent chunks
load("Consensus-dataInput_01.RData")
load("Consensus-dataInput_02.RData")
load("Consensus-dataInput_03.RData")
load("multiExpr.Rdata")
load("01_datTraits.Rdata")
load("02_net.Rdata")
load("02_moduleColors.RData")
```

Define correlation thresholds for MM and GS

```{r define correlation thresholds}
  corMM = 0.9
  corGS = 0.9
```

```{r define top x key drivers and cytoscape threshold}
# top x key drivers to be exported
topx = 30
# connectivity threshold for key drivers to be exported to cytoscape
cytoscape_threshold = 0.1
```


```{r define your font}
define_font = "Arial"
```

```{r load dataset mapping}

filename = paste(Rdata_dir,"dat_accession_dataset_mapping.csv", sep = dirsep)
dat_dataset_mapping = read.csv(filename, header = TRUE, row.names = 1)

```
```

```
Nothing else has to be changed within this script! Just run (Knit) it!
____

This script will plot all the correllation maps. For each of the interesting
modules. So for each module per default.
____

Install necessary packages.

```{r install_packages, include=FALSE, message=FALSE, results='hide'}
list.of.packages = c("WGCNA", "flashClust", "ggplot2", "grid", "extrafont", "qpcR",
"biomaRt", "ggsci")
new.packages = list.of.packages[!(list.of.packages %in%
installed.packages()[,"Package"])]
if(length(new.packages)) install.packages(new.packages)

new.packages = list.of.packages[!(list.of.packages %in%
installed.packages()[,"Package"])]

if(length(new.packages)){

  if (!requireNamespace("BiocManager", quietly = TRUE))
    install.packages("BiocManager")

  for (i in new.packages){
    BiocManager::install(i)
  }
}
```


Load required packages and enable multi-threading.

```{r load_packages}
options(stringsAsFactors = FALSE)

for (i in list.of.packages){
  library(i, character.only = TRUE)
}

```

```{r define font}
#font_import(pattern = "[A/a]rial")
#fonts()
#fonttable()

loadfonts(device = "win")
```

```{r define colors}
#define colors for heatmaps with FCs
color_up = ggsci::pal_npg(palette = "nrc")(10)[8]
color_down = ggsci::pal_npg(palette = "nrc")(10)[4]

#define colors for heatmaps with Abundances
color_high = ggsci::pal_npg(palette = "nrc")(10)[9]
color_mid = "white"
color_low = ggsci::pal_npg(palette = "nrc")(10)[3]

#define colors for GS-MM plots
color_medium = ggsci::pal_npg(palette = "nrc")(10)[2]
color_strict = ggsci::pal_npg(palette = "nrc")(10)[3]

barplot(seq(1,10, by = 1), col = ggsci::pal_npg(palette = "nrc")(10))
```
````

```
## 2 Gene relationship to trait and important modules: Gene Significance and Module
Membership

We quantify associations of individual genes with our trait of interest by defining
Gene Significance GS as (the absolute value of) the correlation between the gene
and the trait.  For each module, we also define a quantitative measure of module
membership MM as the correlation of the module eigengene and the gene expression
pro le. This allows us to quantify the similarity of all genes on the array to
every module.

Using the GS and MM measures, we can identify genes that have a high signi cance
for weight as well as high module membership in interesting modules.  As an
example, we look at the brown module that has the highest association with weight.
We plot a scatterplot of Gene Signi cance vs.  Module Membership.

```{r Gene_Trait_association}
#Define MEs
MEs0 = moduleEigengenes(datExpr, moduleColors)$eigengenes
MEs = orderMEs(MEs0)
```

```{r load FCs and p-values}
#load FC and p-value data proteins
sampleTable_file = "dat_IPA_rbind_padj_LPS.csv" # provide your filename
sampleTable_path = paste(project_dir, sampleTable_file, sep=dirsep) # leave this
like it is!
sample_dat_proteins = read.csv(sampleTable_path, header = TRUE)

dat_vector = colnames(sample_dat_proteins)
dat_vector = dat_vector[-1]
dat_vector

sample_vector = unique(gsub(dat_vector, pattern = "_pvalue", replacement = ""))
sample_vector

colnames(sample_dat_proteins) = c("Accession", dat_vector)

#import gene information
# the following file has to contain the Accession in the first column and then
columns with Genes and Descriptions for example
sampleTable_file = "genes_INTEGRATED.csv" # provide your filename - don't forget
file type!
sampleTable_path = paste(project_dir, sampleTable_file, sep=dirsep) # leave this
like it is!

sampleGenes = read.csv(sampleTable_path, header = TRUE, row.names = NULL)

#sampleGenes = sampleGenes[!duplicated(sampleGenes$Majority.protein.IDs), ]
row.names(sampleGenes) =sampleGenes[, 1]
sampleGenes = sampleGenes[, -1]

#head(sample_dat_proteins)

#load FC and p-value data metabolites
# sampleTable_file = "dat_IPA_metabolites.csv" # provide your filename
# sampleTable_path = paste(Rdata_dir, sampleTable_file, sep=dirsep) # leave this
like it is!
# sample_dat_metabolites = read.csv(sampleTable_path, header = TRUE)
#
# dat_vector = colnames(sample_dat_metabolites)
# dat_vector = dat_vector[-1]
# dat_vector
#
# sample_vector = unique(gsub(dat_vector, pattern = "_pvalue", replacement = ""))
# sample_vector
#
# colnames(sample_dat_metabolites) = c("Accession", dat_vector)
#
```
```

```
# #sample_dat_metabolites =
sample_dat_metabolites[order(sample_dat_metabolites$Accession), ]
#
# head(sample_dat_metabolites)
```

```{r count DE proteins}
pvalue_df = sample_dat_proteins[, which(grepl(colnames(sample_dat_proteins),
                                              pattern = "_pvalue") == TRUE)]
row.names(pvalue_df) = sample_dat_proteins[, 1]

count = as.data.frame(apply(pvalue_df, 1, function(x) sum(x <= 0.05, na.rm =
TRUE)))
rownames(count) = row.names(pvalue_df)
colnames(count) = c("Count_DEs")
```



```{r modules_to_treatment_comparison}
myCorGS = gsub(pattern="^.+[.]", replacement = "", corGS)
myCorMM = gsub(pattern="^.+[.]", replacement = "", corMM)

#names (colors of the modules)
modNames = substring(names(MEs), 3)

geneModuleMembership = as.data.frame(cor(datExpr, MEs, use = "p"))
MMPvalue = as.data.frame(corPvalueStudent(as.matrix(geneModuleMembership),
nSamples))

names(geneModuleMembership) = paste("MM", modNames, sep="");
names(MMPvalue) = paste("p.MM", modNames, sep="");

filename = paste(Rdata_dir, "geneModulMembership.csv", sep = dirsep)
write.csv(x = geneModuleMembership, file = filename)

filename = paste(Rdata_dir, "MMPvalue.csv", sep = dirsep)
write.csv(x = MMPvalue, file = filename)

trait_vector = colnames(datTraits)

for ( i in 1:length(trait_vector)){
  tryCatch({
trait = as.data.frame(datTraits[,i])
names(trait) = trait_vector[i]
name = names(trait)

geneTraitSignificance = as.data.frame(cor(datExpr, trait, use = "p",
                                          method = "pearson"));

GSPvalue = as.data.frame(corPvalueStudent(as.matrix(geneTraitSignificance),
nSamples));

names(geneTraitSignificance) = paste("p.GS.", names(trait), sep="");

names(GSPvalue) = paste("p.GS.", names(trait), sep="");

assign(paste("geneTraitSignificance", trait_vector[i], sep = "_"), value =
geneTraitSignificance)
assign(paste("GSPvalue", trait_vector[i], sep = "_"), value = GSPvalue)

#plot relationships
for (module in intModules){
  tryCatch({
  column = match(module, modNames);
  moduleGenes = moduleColors==module;


  df = as.data.frame(abs(geneModuleMembership[moduleGenes, column]),
                     row.names = rownames(geneModuleMembership[moduleGenes,]))
```

```
  colnames(df) = c("MM")
  df$GS = abs(geneTraitSignificance[moduleGenes, 1])

  # genes above thresholds
  labeled.genes = subset(df, MM >= corMM | GS >= corGS)
  labeled.genes.best = subset(df, MM >= corMM & GS >= corGS)

  # top x genes
  df_top20 = df
  df_top20$sum = df$MM + df$GS
  df_top20 = df_top20[order(-df_top20$sum), ]
  top20 = df_top20[1:topx, ]
  top20 = top20[, -ncol(top20)]

  # fileName = paste("UniprotAccessions-", module, name, "medium", ".txt", sep="")
  # pathname = paste(Rdata_dir, fileName, sep = dirsep)
  # write.table(as.data.frame(row.names(labeled.genes)), file = pathname, row.names
= FALSE, col.names = FALSE)

  fileName = paste("UniprotAccessions-", module, "_", name, "_top", topx, ".txt",
sep="")
  pathname = paste(Rdata_dir, fileName, sep = dirsep)
  write.table(as.data.frame(row.names(top20)), file = pathname, row.names = FALSE,
col.names = FALSE)

  fileName = paste("UniprotAccessionsTop20-", module, "_", name, sep="")
  assign(row.names(top20), x = fileName)

  dat_to_export = as.data.frame(row.names(top20))
  colnames(dat_to_export) = c("Accessions")
  dat_to_export = merge(dat_to_export, sampleGenes, all.x = TRUE, by.x =
"Accessions", by.y = "row.names")

  fileName = paste("UniprotAccessions-Genes-", module, "_", name, "_top", topx,
".csv", sep="")
  pathname = paste(Rdata_dir, fileName, sep = dirsep)
  write.csv(dat_to_export, file = pathname, row.names = FALSE)

   if(dim(labeled.genes.best)[1] > 0){
  fileName = paste("UniprotAccessions-", module, "_", name, "_strict_GS", myCorGS,
"_MM", myCorMM, ".txt", sep="")
  pathname = paste(Rdata_dir, fileName, sep = dirsep)
  write.table(as.data.frame(row.names(labeled.genes.best)), file = pathname,
row.names = FALSE, col.names = FALSE)

  fileName = paste("UniprotAccessions-", module, "_", name, sep="")
  assign(row.names(labeled.genes.best), x = fileName)

  dat_to_export = as.data.frame(row.names(labeled.genes.best))
  colnames(dat_to_export) = c("Accessions")
  dat_to_export = merge(dat_to_export, sampleGenes, all.x = TRUE, by.x =
"Accessions", by.y = "row.names")

  fileName = paste("UniprotAccessions-Genes-", module, "_", name, "_strict_GS",
                   myCorGS, "_MM", myCorMM, ".csv", sep="")
  pathname = paste(Rdata_dir, fileName, sep = dirsep)
  write.csv(dat_to_export, file = pathname, row.names = FALSE)
   }

  df$Colors = "black"
  df[which(abs(df$MM) >= corMM | abs(df$GS) >= corGS), "Colors"] = color_medium
  df[which(abs(df$MM) >= corMM & abs(df$GS) >= corGS), "Colors"] = color_strict
  df = merge(df, sampleGenes, by = "row.names")
  row.names(df) = df$Row.names
  df = df[, -1]
  df$Label = NA
  df[which(abs(df$MM) >= corMM & abs(df$GS) >= corGS), "Label"] =
df$Gene[which(abs(df$MM) >= corMM &

abs(df$GS) >= corGS)]
```

```
  if(dim(labeled.genes.best)[1] == 0){

  figure_name = paste("GS_GM_plot_ggplot_medium", name, module, "GS", myCorGS,
"MM", myCorMM, sep = "_")
  pdf_name = paste(figure_name, ".wmf", sep="")
  plotfile = paste(plots_dir, pdf_name, sep=dirsep)
  win.metafile(file = plotfile,
      width = 6, height = 6,
      pointsize = 12,
      family = define_font)

  g = ggplot(df, aes(x = MM, y = GS, label = Label)) +
    geom_point(shape=16, size = 2, colour = df$Colors) +
    theme_classic() +
    scale_x_continuous(limits = c(0,1), expand = c(0,0)) +
    scale_y_continuous(limits = c(0,1), expand = c(0,0)) +
    xlab(paste("Module Membership ", module, " module", sep = "")) +
    ylab(paste("Gene Significance ", name, sep = "")) +
    ggtitle(paste("Module Membership vs. Gene Significance ", module, " module",
sep = "")) +
    geom_vline(xintercept = corMM, linetype =2, col = "black") +
    geom_hline(yintercept = corGS, linetype =2, col = "black") +
    # geom_text(data = df, aes(MM, GS, label = Label, hjust = -0.1),
    #           size = rel(3)) +
    theme(axis.title.y = element_text(size = rel(1), face = "bold", color =
"black",
                                       margin = margin(t=0.5, r = 0.5, b = 0.5, l =
0.5, "cm")),
            axis.title.x = element_text(size = rel(1), face = "bold", color =
"black",
                                         margin = margin(t=0.5, r = 0.5, b = 0.5, l
= 0.5, "cm")),
            axis.text.x = element_text(size = rel(1), color = "black"),
            axis.text.y = element_text(size = rel(1), color = "black", hjust = 1),
          axis.line = element_line(colour = "black", size = 1),
          axis.ticks = element_line(colour = "black", size = 1),
          plot.margin = margin(1,1,1,1, "cm"),
          plot.title = element_text(size = rel(1.2), face = "bold", color =
"black", hjust = 0,
                                     margin = margin(0.5, 0.5, 0.5, 0.5, "cm")))

  print(g)
  dev.off()

  }

  if(dim(labeled.genes.best)[1] > 0){

     figure_name = paste("GS_GM_plot_ggplot_strict", name, module, "GS", myCorGS,
"MM", myCorMM, sep = "_")
  pdf_name = paste(figure_name, ".wmf", sep="")
  plotfile = paste(plots_dir, pdf_name, sep=dirsep)
  win.metafile(file = plotfile,
      width = 6, height = 6,
      pointsize = 12,
      family = define_font)

  g = ggplot(df, aes(x = MM, y = GS, label = Label)) +
    geom_point(shape=16, size = 2, colour = df$Colors) +
    theme_classic() +
    scale_x_continuous(limits = c(0,1), expand = c(0,0)) +
    scale_y_continuous(limits = c(0,1), expand = c(0,0)) +
    xlab(paste("Module Membership ", module, " module", sep = "")) +
    ylab(paste("Gene Significance ", name, sep = "")) +
    ggtitle(paste("Module Membership vs. Gene Significance ", module, " module",
sep = "")) +
    geom_vline(xintercept = corMM, linetype =2, col = "black") +
    geom_hline(yintercept = corGS, linetype =2, col = "black") +
    # geom_text(data = df, aes(MM, GS, label = Label, hjust = -0.1),
```

```
    #           size = rel(3)) +
    theme(axis.title.y = element_text(size = rel(1), face = "plain", color =
"black",
                                      margin = margin(t=0.5, r = 0.5, b = 0.5, l =
0.5, "cm")),
            axis.title.x = element_text(size = rel(1), face = "plain", color =
"black",
                                        margin = margin(t=0.5, r = 0.5, b = 0.5, l
= 0.5, "cm")),
            axis.text.x = element_text(size = rel(1), color = "black", face =
"plain"),
            axis.text.y = element_text(size = rel(1), color = "black", hjust = 1,
face = "plain"),
          axis.line = element_line(colour = "black", size = 0.5),
          axis.ticks = element_line(colour = "black", size = 0.5),
          plot.margin = margin(1,1,1,1, "cm"),
          plot.title = element_text(size = rel(1.2), face = "bold", color =
"black", hjust = 0,
                                    margin = margin(0.5, 0.5, 0.5, 0.5, "cm")))
   print(g)

  dev.off()

  figure_name = paste("GS_GM_plot_ggplot_strict", name, module, "GS", myCorGS,
"MM", myCorMM, sep = "_")
  pdf_name = paste(figure_name, ".pdf", sep="")
  plotfile = paste(plots_dir, pdf_name, sep=dirsep)
 pdf(file = plotfile,
      width = 6, height = 6,
      pointsize = 12)

   print(g)

  dev.off()
  }

  df = df_top20
  df$Colors = "black"
  df[1:topx, "Colors"] = color_strict
  df = merge(df, sampleGenes, by = "row.names")
  row.names(df) = df$Row.names
  df = df[, -1]
  df$Label = NA
  df = df[order(df$sum, decreasing = TRUE), ]
  df[1:topx, "Label"] = df$Gene[1:topx]

  figure_name = paste("GS_GM_plot_ggplot_top", topx, name, module, sep = "_")
  pdf_name = paste(figure_name, ".wmf", sep="")
  plotfile = paste(plots_dir, pdf_name, sep=dirsep)
  win.metafile(file = plotfile,
      width = 6, height = 6,
      pointsize = 12,
      family = define_font)

  g = ggplot(df, aes(x = MM, y = GS, label = Label)) +
    geom_point(shape=16, size = 2, colour = df$Colors) +
    theme_classic() +
    scale_x_continuous(limits = c(0,1), expand = c(0,0)) +
    scale_y_continuous(limits = c(0,1), expand = c(0,0)) +
    xlab(paste("Module Membership ", module, " module", sep = "")) +
    ylab(paste("Gene Significance ", name, sep = "")) +
    ggtitle(paste("Module Membership vs. Gene Significance ", module, " module",
sep = "")) +
    geom_vline(xintercept = corMM, linetype =2, col = "black") +
    geom_hline(yintercept = corGS, linetype =2, col = "black") +
    # geom_text(data = df, aes(MM, GS, label = Label, hjust = -0.1),
    #           size = rel(3)) +
    theme(axis.title.y = element_text(size = rel(1), face = "plain", color =
"black",
```

```
                                      margin = margin(t=0.5, r = 0.5, b = 0.5, l =
0.5, "cm")),
            axis.title.x = element_text(size = rel(1), face = "plain", color =
"black",
                                        margin = margin(t=0.5, r = 0.5, b = 0.5, l
= 0.5, "cm")),
            axis.text.x = element_text(size = rel(1), color = "black"),
            axis.text.y = element_text(size = rel(1), color = "black", hjust = 1),
          axis.line = element_line(colour = "black", size = 0.5),
          axis.ticks = element_line(colour = "black", size = 0.5),
          plot.margin = margin(1,1,1,1, "cm"),
          plot.title = element_text(size = rel(1.2), face = "bold", color =
"black", hjust = 0,
                                    margin = margin(0.5, 0.5, 0.5, 0.5, "cm")))

  print(g)

  dev.off()

  paste("GS_GM_plot_ggplot_top", topx, name, module, sep = "_")
  pdf_name = paste(figure_name, ".pdf", sep="")
  plotfile = paste(plots_dir, pdf_name, sep=dirsep)
  pdf(file = plotfile,
      width = 6, height = 6,
      pointsize = 12)

  print(g)

  dev.off()

  }, error = function(e){cat("ERROR:", conditionMessage(e), module, i, "\n")})
}
 }, error = function(e){cat("ERROR:", conditionMessage(e), i, "\n")})
}
```

Save results.

```{r save results}
summary_GTS = do.call(cbind, lapply(ls(pattern = "geneTraitSignificance_"),
                                  function(x){
                                    get_dat = get(x)
                                    get_dat
                                  }))

filename = paste(Rdata_dir, "geneTraitSignificance.csv", sep = dirsep)
write.csv(x = summary_GTS, file = filename)

summary_GSP = do.call(cbind, lapply(ls(pattern = "GSPvalue_"),
                                  function(x){
                                    get_dat = get(x)
                                    get_dat
                                  }))

filename = paste(Rdata_dir, "GSPvalue.csv", sep = dirsep)
write.csv(x = summary_GSP, file = filename)
```

Clearly, GS and MM are highly correlated for some modules, illustrating that genes highly significantly
associated with a trait are often also the most important (central) elements of modules associated with the trait. The reader is encouraged to try this code with other significance trait/module correlation (for example, the magenta, midnightblue, and red modules with weight).

Plot Key drivers (GS and MM > threshold)

```{r plot key drivers in bubble plot}
```

```
key_dat_merge_proteins = merge(sampleGenes, sample_dat_proteins, by.x =
"row.names",
                               by.y = "Accession")

# key_dat_metabolites = as.data.frame(sample_dat_metabolites$Accession)
# key_dat_metabolites$Name = key_dat_metabolites$`sample_dat_metabolites$Accession`
# colnames(key_dat_metabolites) = c("Accession", "Name")
#
# key_dat_merge_metabolites = merge(key_dat_metabolites, sample_dat_metabolites)

key_dat_complete = do.call(rbind, lapply(ls(pattern = "key_dat_merge_"),
                                         function(x){
                                           get_dat = get(x)
                                           get_dat
                                         }))

rownames(key_dat_complete) = key_dat_complete$Row.names
key_dat_complete = key_dat_complete[, -1]
#
# filename = paste(Rdata_dir, "dat_IPA_genes.csv", sep = dirsep)
# write.csv(key_dat_complete, filename)

sample_dat_complete = do.call(rbind, lapply(ls(pattern = "sample_dat_"),
                                            function(x){
                                              get_dat = get(x)
                                              get_dat
                                            }))


for (i in trait_vector){
  tryCatch({

    search_i = paste("_", i, "$", sep = "")

    pattern_search = as.list(ls(pattern = search_i)[grepl(ls(pattern = search_i),
                                                   pattern = "UniprotAccessions-")
== TRUE])

    key_dat = do.call(rbind, lapply(pattern_search,
                                    function(x){
                                      get_dat = as.data.frame(get(x))
                                      module = gsub(x, pattern =
"UniprotAccessions-", replacement = "")
                                      module = gsub(module, pattern = search_i,
replacement = "")

                                      get_dat$Module = module
                                      get_dat
                                    }))
    colnames(key_dat) = c("Accession", "Module")
    rownames(key_dat) = key_dat$Accession

    key_dat_genes_merge = merge(key_dat, key_dat_complete, by = "row.names", all.x
= TRUE)

    filename = paste("UniprotAccessions-", i, "-KeyDrivers-Genes", "-GS", myCorGS,
"-MM", myCorMM, ".csv", sep = "")
    pathname = paste(Rdata_dir, filename, sep = dirsep)

    write.csv(pathname, x = key_dat_genes_merge)

    key_dat_accessions_merge = merge(key_dat, sample_dat_complete, by =
"Accession")

    filename = paste("UniprotAccessions-", i, "-KeyDrivers-Accessions", "-GS",
myCorGS, "-MM", myCorMM, ".csv", sep = "")
    pathname = paste(Rdata_dir, filename, sep = dirsep)

    write.csv(pathname, x = key_dat_accessions_merge)
```

```
    #Cluster key drivers
    sample_dat_cluster = key_dat_accessions_merge
    sample_dat_cluster[is.na(sample_dat_cluster)] = 0

    cluster_vector = seq(from = 3, to = ncol(sample_dat_cluster)-1, by = 2)
    dend_rows = hclust(dist(sample_dat_cluster[, cluster_vector]))
    cluster_membership = cutree(dend_rows, ifelse(nrow(sample_dat_cluster) > 20,

floor(nrow(sample_dat_cluster)/10),
                                                  2))
    key_dat_accessions_merge$Order = dend_rows$order
    key_dat_accessions_merge$Cluster = cluster_membership

    for (j in sample_vector){
      tryCatch({
        dat_subset = as.data.frame(key_dat_accessions_merge[ , grepl(paste("^", j,
sep = ""),

colnames(key_dat_accessions_merge))])
        dat_subset$Treatment = j

        dat_cbind = as.data.frame(cbind(key_dat_accessions_merge$Accession,
                                        key_dat_accessions_merge$Order,
                                        key_dat_accessions_merge$Cluster,
                                        key_dat_accessions_merge$Module,
dat_subset))

        colnames(dat_cbind) = c("Accession", "Order", "Cluster", "Module", "FC",
"pvalue", "Treatment")

        assign(x = paste("dat_dotplot", j, sep = "_"), value = dat_cbind)
      }, error = function(e){cat("ERROR:", conditionMessage(e), i, j, "\n")})
    }

    dat_rbind = do.call(rbind, lapply(ls(pattern = "dat_dotplot_"),
                                      function(x){
                                        get_dat = get(x)
                                        get_dat
                                      }))

    # dat_to_plot = dat_rbind[ which(dat_rbind$pvalue < 0.05 &
    #                                  (dat_rbind$FC > 0.1 | dat_rbind$FC < -0.1)),
]

    dat_to_plot = dat_rbind

    dat_to_plot = dat_to_plot

    FC_min = abs(min(dat_to_plot$FC, na.rm = TRUE))
    FC_max = abs(max(dat_to_plot$FC, na.rm = TRUE))
    FC_limit = ceiling(max(FC_min, FC_max))

    maxp = ifelse(-log10(min(dat_to_plot$pvalue, na.rm = TRUE)) <=5, 5,
                  -log10(min(dat_to_plot$pvalue, na.rm = TRUE)))

    color_list = setNames(as.vector(dat_cbind$Module), dat_cbind$Module)

    dat_to_plot = merge(dat_to_plot, sampleGenes, by.x = "Accession", by.y =
"row.names")

    plot_file = paste("KeyDrivers_BubblePlot_", i, "-GS", myCorGS, "-MM", myCorMM,
".wmf", sep = "")
    plot_path = paste(plots_dir, plot_file, sep = dirsep)
    win.metafile(file = plot_path,
                 width = 8,
                 height = dim(dat_to_plot)[1]/(length(sample_vector)*4) + 3,
                 pointsize = 12,
                 family = define_font)

    b = ggplot(dat_to_plot, aes(x = Treatment, y = reorder(Gene, Order),
```

````
                                size = -log10(pvalue), fill = FC)) +
      geom_point(shape = 21) +
      scale_fill_gradient2(low = color_down,
                           mid = "white",
                           high = color_up, name = "Log2(FC)",
                           midpoint = 0,
                           breaks = c(-FC_limit, 0, FC_limit),
                           limits = c(-FC_limit, FC_limit)) +
      scale_size_area(breaks = c(1.3, 2, 5), limits = c(1.3, maxp)) +
      theme_classic() +
      theme(legend.position = "right",
            legend.title = element_text(size = rel(1), color = "black", face =
"bold"),
            axis.title.y = element_blank(),
            axis.title.x = element_blank(),
            axis.text.x = element_text(size = rel(1), face = "bold", color =
"black", angle = 90,
                                       hjust = 1, vjust = 0.5),
            axis.text.y = element_text(size = rel(1), face = "bold", color =
color_list, hjust = 1)) +
      labs(size = "-Log10(p-value)") +
      facet_grid(Cluster ~ ., scales = "free", space = "free") +
      theme(panel.background = element_rect(fill = NA, color = "black"),
            strip.text.y = element_blank())

    #b + annotate("text", label = dat_cbind$Module, y = dat_cbind$Accession, x = -
1,
    #color = color_list)

    print(b)

    dev.off()

    plot_file = paste("KeyDrivers_BubblePlot_", i, "-GS", myCorGS, "-MM", myCorMM,
".pdf", sep = "")
    plot_path = paste(plots_dir, plot_file, sep = dirsep)
    pdf(file = plot_path,
        width = 8,
        height = dim(dat_to_plot)[1]/(length(sample_vector)*4) + 3,
        pointsize = 12)

    print(b)

    dev.off()
  }, error = function(e){cat("ERROR:", conditionMessage(e), i, "\n")})
}
```


```{r top 20 key drivers in bubble plot}
for (i in trait_vector){
  tryCatch({

    search_i = paste("_", i, "$", sep = "")

    pattern_search = as.list(ls(pattern = search_i)[grepl(ls(pattern = search_i),
                                                          pattern =
"UniprotAccessionsTop20-") == TRUE])


  key_dat = do.call(rbind, lapply(pattern_search,
                                  function(x){
                                    get_dat = as.data.frame(get(x))
                                    module = gsub(x, pattern =
"UniprotAccessionsTop20-",
                                                  replacement = "")
                                    module = gsub(module, pattern = search_i,
replacement = "")
                                    get_dat$Module = module
````

```
                                    get_dat
                                  }))
  colnames(key_dat) = c("Accession", "Module")
  rownames(key_dat) = key_dat$Accession

  key_dat_genes_merge = merge(key_dat, key_dat_complete, by = "row.names")

  filename = paste("UniprotAccessions-Top", topx, "-", i, "-KeyDrivers-Genes",
".csv", sep = "")
  pathname = paste(Rdata_dir, filename, sep = dirsep)

  write.csv(pathname, x = key_dat_genes_merge)

  key_dat_accessions_merge = merge(key_dat, sample_dat_complete, by = "Accession")

  filename = paste("UniprotAccessions-Top", topx, "-", i, "-KeyDrivers-Accessions",
".csv", sep = "")
  pathname = paste(Rdata_dir, filename, sep = dirsep)

  write.csv(pathname, x = key_dat_accessions_merge)

  #Cluster key drivers
    sample_dat_cluster = key_dat_accessions_merge
    sample_dat_cluster[is.na(sample_dat_cluster)] = 0

    cluster_vector = seq(from = 3, to = ncol(sample_dat_cluster)-1, by = 2)
    dend_rows = hclust(dist(sample_dat_cluster[, cluster_vector]))
    cluster_membership = cutree(dend_rows, ifelse(nrow(sample_dat_cluster) > 20,

floor(nrow(sample_dat_cluster)/10),
                                                2))
    key_dat_accessions_merge$Order = dend_rows$order
    key_dat_accessions_merge$Cluster = cluster_membership

  for (j in sample_vector){
    tryCatch({
      dat_subset = as.data.frame(key_dat_accessions_merge[ , grepl(paste("^", j,
sep = ""),

colnames(key_dat_accessions_merge))])
      dat_subset$Treatment = j

      dat_cbind = as.data.frame(cbind(key_dat_accessions_merge$Accession,
                                      key_dat_accessions_merge$Order,
                                      key_dat_accessions_merge$Cluster,
                                      key_dat_accessions_merge$Module, dat_subset))

      colnames(dat_cbind) = c("Accession", "Order", "Cluster", "Module", "FC",
"pvalue", "Treatment")

      assign(x = paste("datTop20_dotplot", j, sep = "_"), value = dat_cbind)
    }, error = function(e){cat("ERROR:", conditionMessage(e), i, j, "\n")})
  }

  dat_rbind = do.call(rbind, lapply(ls(pattern = "datTop20_dotplot_"),
                                          function(x){
                                          get_dat = get(x)
                                          get_dat
                                          }))

  # dat_to_plot = dat_rbind[ which(dat_rbind$pvalue < 0.05 &
  #                                   (dat_rbind$FC > 0.1 | dat_rbind$FC < -0.1)), ]

  dat_to_plot = dat_rbind

  dat_to_plot = dat_to_plot

  FC_min = abs(min(dat_to_plot$FC, na.rm = TRUE))
  FC_max = abs(max(dat_to_plot$FC, na.rm = TRUE))
  FC_limit = ceiling(max(FC_min, FC_max))
```

```
  maxp = ifelse(-log10(min(dat_to_plot$pvalue, na.rm = TRUE)) <=5, 5,
                -log10(min(dat_to_plot$pvalue, na.rm = TRUE)))

  color_list = setNames(as.vector(dat_cbind$Module), dat_cbind$Module)

  dat_to_plot = merge(dat_to_plot, sampleGenes, by.x = "Accession", by.y =
"row.names")

  plot_file = paste("KeyDrivers_BubblePlot_Top", topx, "_", i, ".wmf", sep = "")
    plot_path = paste(plots_dir, plot_file, sep = dirsep)
   win.metafile(file = plot_path,
      width = 8,
      height = dim(dat_to_plot)[1]/(length(sample_vector)*4) + 3,
      pointsize = 12,
      family = define_font)

    b = ggplot(dat_to_plot, aes(x = Treatment, y = reorder(Gene, Order),
                               size = -log10(pvalue), fill = FC)) +
      geom_point(shape = 21) +
      scale_fill_gradient2(low = color_down,
                           mid = "white",
                           high = color_up, name = "Log2(FC)",
                           midpoint = 0,
                           breaks = c(-FC_limit, 0, FC_limit),
                           limits = c(-FC_limit, FC_limit)) +
      scale_size_area(breaks = c(1.3, 2, 5), limits = c(1.3, maxp)) +
      theme_classic() +
      theme(legend.position = "right",
            legend.title = element_text(size = rel(1), color = "black", face =
"bold"),
            axis.title.y = element_blank(),
            axis.title.x = element_blank(),
            axis.text.x = element_text(size = rel(1), face = "bold", color =
"black", angle = 90,
                                        hjust = 1, vjust = 0.5),
            axis.text.y = element_text(size = rel(1), face = "bold", color =
color_list, hjust = 1)) +
      labs(size = "-Log10(p-value)") +
      facet_grid(Cluster ~ ., scales = "free", space = "free") +
      theme(panel.background = element_rect(fill = NA, color = "black"),
            strip.text.y = element_blank())

    print(b)

    dev.off()

    plot_file = paste("KeyDrivers_BubblePlot_Top", topx, "_", i, ".pdf", sep = "")
    plot_path = paste(plots_dir, plot_file, sep = dirsep)
    pdf(file = plot_path,
        width = 8,
        height = dim(dat_to_plot)[1]/(length(sample_vector)*4) + 3,
        pointsize = 12)

    print(b)

    dev.off()


  }, error = function(e){cat("ERROR:", conditionMessage(e), i, "\n")})
}
```

Key drivers in scatter plot

```{r plot key drivers in scatter plot with gene names}
MM_dat = geneModuleMembership
  colnames(MM_dat) = gsub(colnames(MM_dat), pattern = "MM", replacement = "")
```

```
  # MM_dat_genes = as.data.frame(matrix(nrow = dim(MM_dat)[1], ncol =
dim(MM_dat)[2]))
  # colnames(MM_dat_genes) = colnames(MM_dat)
  # rownames(MM_dat_genes) = rownames(MM_dat)

  MM_dat_genes = MM_dat
  MM_dat_genes$Name = NA

  for ( i in 1:nrow(MM_dat)){
    get_dat = MM_dat_genes[i, ]
    merge_dat = merge(get_dat, sampleGenes, by = "row.names")

    if(nrow(merge_dat) == 0){
      MM_dat_genes$Name[i] = rownames(MM_dat_genes)[i]
    } else {
      MM_dat_genes$Name[i] = merge_dat$Gene[1]
    }
  }

  MM_dat_pvalue = MMPvalue
  colnames(MM_dat_pvalue) = gsub(colnames(MM_dat_pvalue), pattern = "p.MM",
replacement = "")
  colnames(MM_dat_pvalue) = paste(colnames(MM_dat_pvalue), "_pvalue", sep = "")


for (i in trait_vector){
  tryCatch({
  search_i = paste("_", i, "$", sep = "")

    pattern_search = as.list(ls(pattern = search_i)[grepl(ls(pattern = search_i),
                                                      pattern = "UniprotAccessions-")
== TRUE])


  key_dat = do.call(rbind, lapply(pattern_search,
                                  function(x){
                                    get_dat = as.data.frame(get(x))
                                    module = gsub(x, pattern = "UniprotAccessions-
", replacement = "")
                                    module = gsub(module, pattern = search_i,
replacement = "")
                                    get_dat$Module = module
                                    get_dat
                                  }))

  colnames(key_dat) = c("Accession", "Module")
  rownames(key_dat) = key_dat$Accession

  GS_dat = get(paste("geneTraitSignificance", i, sep = "_"))
  GS_pvalue = get(paste("GSPvalue", i, sep = "_"))

  modules_selected = unique(key_dat$Module)


  for (j in modules_selected){
    tryCatch({
      get_dat_key = key_dat[key_dat$Module == j, ]
      get_dat_MM = as.data.frame(MM_dat_genes[, which(colnames(MM_dat) == j)])

      get_dat_MMp = as.data.frame(MM_dat_pvalue[,
                                                which(colnames(MM_dat_pvalue) ==
                                                        paste(j, "_pvalue", sep =
""))])

      get_dat_MM = as.data.frame(cbind(get_dat_MM, get_dat_MMp, MM_dat_genes$Name))

      colnames(get_dat_MM) = c("MM", "MM_pvalue", "Gene")
      rownames(get_dat_MM) = rownames(MM_dat_genes)

      get_dat_summary = merge(get_dat_key, get_dat_MM, by = "row.names")
```

```
      get_dat_complete = merge(get_dat_summary, GS_dat, by.x = "Row.names", by.y =
"row.names")
      rownames(get_dat_complete) = get_dat_complete$Row.names
      get_dat_complete = get_dat_complete[, -1]
      get_dat_complete = get_dat_complete[, -1]
      get_dat_complete = merge(get_dat_complete, GS_pvalue, by = "row.names")
      rownames(get_dat_complete) = get_dat_complete$Row.names
      get_dat_complete = get_dat_complete[, -1]
      colnames(get_dat_complete) = c("Module", "MM", "MM_pvalue","Gene", "GS",
"GS_pvalue")
      assign(x = paste("GS_MM_Plot_GeneKeyDrivers", i, j, sep = "_"), value =
get_dat_complete)

      get_dat_dataset = merge(get_dat_complete, dat_dataset_mapping, by =
"row.names")

      get_dat_dataset$MM = abs(get_dat_dataset$MM)
  get_dat_dataset$GS = abs(get_dat_dataset$GS)

      figure_name = paste("GS_GM_plot_GeneKeyDrivers_Datasets", i, j, "GS",
myCorGS, "MM", myCorMM, sep = "_")
  pdf_name = paste(figure_name, ".wmf", sep="")
  plotfile = paste(plots_dir, pdf_name, sep=dirsep)
  win.metafile(file = plotfile,
      width = 6.5, height = 5,
      pointsize = 14,
      family = define_font)

  g = ggplot(get_dat_dataset, aes(x = MM, y = GS, color = Dataset)) +
    geom_point(size = 3) +
    scale_color_npg() +
    theme_classic() +
    xlim(corMM,1) +
    ylim(corGS,1) +
    xlab("Module Membership") +
    ylab("Gene Trait Significance") +
    ggtitle(paste("Key Drivers ", i,sep = "")) +
    #geom_vline(xintercept = ((1-corMM)/2)+corMM, linetype =2, col = "black") +
    #geom_hline(yintercept = ((1-corGS)/2)+corGS, linetype =2, col = "black") +
    theme(axis.title.y = element_text(size = rel(1), face = "bold", color =
"black",
                                      margin = margin(t=0.5, r = 0.5, b = 0.5, l =
0.5, "cm")),
            axis.title.x = element_text(size = rel(1), face = "bold", color =
"black",
                                        margin = margin(t=0.5, r = 0.5, b = 0.5, l
= 0.5, "cm")),
            axis.text.x = element_text(size = rel(1), color = "black"),
            axis.text.y = element_text(size = rel(1), color = "black", hjust = 1),
          axis.line = element_line(colour = "black", size = 1),
          axis.ticks = element_line(colour = "black", size = 1),
          legend.title = element_text(size = rel(1), face = "bold", color =
"black"),
          plot.margin = margin(1,1,1,1, "cm"),
          plot.title = element_text(size = rel(1), face = "bold", color = "black",
hjust = 0.5,
                                    margin = margin(0.5, 0.5, 0.5, 0.5, "cm")))
   print(g)
  dev.off()

  figure_name = paste("GS_GM_plot_GeneKeyDrivers_Datasets", i, j, "GS", myCorGS,
"MM", myCorMM, sep = "_")
  pdf_name = paste(figure_name, ".png", sep="")
  plotfile = paste(plots_dir, pdf_name, sep=dirsep)
 png(file = plotfile,
     units = "in",
     res = 800,
      width = 6.5, height = 5,
      pointsize = 14,
      family = define_font)
```

```
   print(g)

  dev.off()
    }, error = function(e){cat("ERROR:", conditionMessage(e), i, j, "\n")})
  }



  df = do.call(rbind, lapply(ls(pattern = paste("GS_MM_Plot_GeneKeyDrivers", i, sep
= "_")),
                                  function(x){
                                    get_dat = as.data.frame(get(x))
                                    get_dat
                                  }))

  df = merge(df, count, by = "row.names")
  row.names(df) = df$Row.names
  df = df[, -1]

  df$Colors = "white"
  df[which(df$Count_DEs > 0), "Colors"] = df$Module[which(df$Count_DEs > 0)]
  df$Label = ""
  df[which(df$Count_DEs > 0), "Label"] = df$Gene[which(df$Count_DEs > 0)]

  df$MM = abs(df$MM)
  df$GS = abs(df$GS)

  labeled.genes.best = subset(df, MM >= corMM & GS >= corGS)

  figure_name = paste("GS_GM_plot_GeneKeyDrivers", i, "GS", myCorGS, "MM", myCorMM,
sep = "_")
  pdf_name = paste(figure_name, ".wmf", sep="")
  plotfile = paste(plots_dir, pdf_name, sep=dirsep)
  win.metafile(file = plotfile,
      width = 5, height = 5,
      pointsize = 14,
      family = define_font)

  g = ggplot(df, aes(x = MM, y = GS)) +
    geom_point(shape=21, size = 3, colour = df$Module, fill = df$Colors) +
    theme_classic() +
    xlim(corMM,1) +
    ylim(corGS,1) +
    xlab("Module Membership") +
    ylab("Gene Trait Significance") +
    ggtitle(paste("Key Drivers ", i,sep = "")) +
    #geom_vline(xintercept = ((1-corMM)/2)+corMM, linetype =2, col = "black") +
    #geom_hline(yintercept = ((1-corGS)/2)+corGS, linetype =2, col = "black") +
    geom_text(data = labeled.genes.best, aes(MM, GS, label =
labeled.genes.best$Label, hjust = -0.1),
              size = rel(3)) +
    theme(axis.title.y = element_text(size = rel(1), face = "bold", color =
"black",
                                      margin = margin(t=0.5, r = 0.5, b = 0.5, l =
0.5, "cm")),
            axis.title.x = element_text(size = rel(1), face = "bold", color =
"black",
                                        margin = margin(t=0.5, r = 0.5, b = 0.5, l
= 0.5, "cm")),
            axis.text.x = element_text(size = rel(1), color = "black"),
            axis.text.y = element_text(size = rel(1), color = "black", hjust = 1),
          axis.line = element_line(colour = "black", size = 1),
          axis.ticks = element_line(colour = "black", size = 1),
          plot.margin = margin(1,1,1,1, "cm"),
          plot.title = element_text(size = rel(1), face = "bold", color = "black",
hjust = 0.5,
                                  margin = margin(0.5, 0.5, 0.5, 0.5, "cm")))
   print(g)
  dev.off()
```

```
  figure_name = paste("GS_GM_plot_GeneKeyDrivers", i, "GS", myCorGS, "MM", myCorMM,
sep = "_")
  pdf_name = paste(figure_name, ".pdf", sep="")
  plotfile = paste(plots_dir, pdf_name, sep=dirsep)
  pdf(file = plotfile,
      width = 5, height = 5,
      pointsize = 14)

     print(g)

  dev.off()

  figure_name = paste("GS_GM_plot_GeneKeyDrivers", i, "GS", myCorGS, "MM", myCorMM,
"noLabel", sep = "_")
  pdf_name = paste(figure_name, ".wmf", sep="")
  plotfile = paste(plots_dir, pdf_name, sep=dirsep)
  win.metafile(file = plotfile,
      width = 5, height = 5,
      pointsize = 14,
      family = define_font)

  g = ggplot(df, aes(x = MM, y = GS)) +
    geom_point(shape=21, size = 3, colour = df$Module, fill = df$Colors) +
    theme_classic() +
    xlim(corMM,1) +
    ylim(corGS,1) +
    xlab("Module Membership") +
    ylab("Gene Trait Significance") +
    ggtitle(paste("Key Drivers ", i,sep = "")) +
    #geom_vline(xintercept = ((1-corMM)/2)+corMM, linetype =2, col = "black") +
    #geom_hline(yintercept = ((1-corGS)/2)+corGS, linetype =2, col = "black") +
    # geom_text(data = labeled.genes.best, aes(MM, GS, label =
labeled.genes.best$Label, hjust = -0.1),
    #           size = rel(3)) +
    theme(axis.title.y = element_text(size = rel(1), face = "bold", color =
"black",
                                      margin = margin(t=0.5, r = 0.5, b = 0.5, l =
0.5, "cm")),
            axis.title.x = element_text(size = rel(1), face = "bold", color =
"black",
                                        margin = margin(t=0.5, r = 0.5, b = 0.5, l
= 0.5, "cm")),
            axis.text.x = element_text(size = rel(1), color = "black"),
            axis.text.y = element_text(size = rel(1), color = "black", hjust = 1),
          axis.line = element_line(colour = "black", size = 1),
          axis.ticks = element_line(colour = "black", size = 1),
          plot.margin = margin(1,1,1,1, "cm"),
          plot.title = element_text(size = rel(1), face = "bold", color = "black",
hjust = 0.5,
                                    margin = margin(0.5, 0.5, 0.5, 0.5, "cm")))
   print(g)
  dev.off()

  figure_name = paste("GS_GM_plot_GeneKeyDrivers", i, "GS", myCorGS, "MM", myCorMM,
"noLabel", sep = "_")
  pdf_name = paste(figure_name, ".png", sep="")
  plotfile = paste(plots_dir, pdf_name, sep=dirsep)
  png(file = plotfile,
      units = "in",
      res = 800,
      width = 5, height = 5,
      pointsize = 14,
      family = define_font)


   print(g)
  dev.off()

  df$Trait = i
```

```
  assign(value = df, x = paste("GS_MM_Summary_KeyDrivers", i, sep = "_"))

  }, error = function(e){cat("ERROR:", conditionMessage(e), i, "\n")})
}

  KeyDrivers_Summary = do.call(rbind, lapply(ls(pattern =
"GS_MM_Summary_KeyDrivers"),
                                  function(x){
                                    get_dat = as.data.frame(get(x))
                                    get_dat$Accession = rownames(get_dat)
                                    rownames(get_dat) = NULL
                                    get_dat
                                  }))

  filename = paste("KeyDrivers_Summary", "-GS", myCorGS, "-MM", myCorMM, ".csv",
sep = "")
  filename = paste(Rdata_dir, filename, sep = dirsep)
  write.csv(x = KeyDrivers_Summary, file = filename)

  KeyDrivers_Summary_FCs_pvalues = merge(KeyDrivers_Summary, sample_dat_complete,
by = "Accession",
                                         all.x = TRUE)

  filename = paste("KeyDrivers_Summary", "-GS", myCorGS, "-MM", myCorMM,
"_FCs_pvalues", ".csv", sep = "")
  filename = paste(Rdata_dir, filename, sep = dirsep)
  write.csv(x = KeyDrivers_Summary_FCs_pvalues, file = filename)
```

```{r plot top 20 key drivers in scatter plot with gene names}
MM_dat = geneModuleMembership
  colnames(MM_dat) = gsub(colnames(MM_dat), pattern = "MM", replacement = "")

  # MM_dat_genes = as.data.frame(matrix(nrow = dim(MM_dat)[1], ncol =
dim(MM_dat)[2]))
  # colnames(MM_dat_genes) = colnames(MM_dat)
  # rownames(MM_dat_genes) = rownames(MM_dat)

  MM_dat_genes = MM_dat
  MM_dat_genes$Name = NA

  for ( i in 1:nrow(MM_dat)){
    get_dat = MM_dat_genes[i, ]
    merge_dat = merge(get_dat, sampleGenes, by = "row.names")

    if(nrow(merge_dat) == 0){
      MM_dat_genes$Name[i] = rownames(MM_dat_genes)[i]
    } else {
      MM_dat_genes$Name[i] = merge_dat$Gene[1]
    }
  }


for (i in trait_vector){
  tryCatch({
  search_i = paste("_", i, "$", sep = "")

    pattern_search = as.list(ls(pattern = search_i)[grepl(ls(pattern = search_i),
                                                      pattern =
"UniprotAccessionsTop20-") == TRUE])


  key_dat = do.call(rbind, lapply(pattern_search,
                                  function(x){
                                    get_dat = as.data.frame(get(x))
                                    module = gsub(x, pattern =
"UniprotAccessionsTop20-",
                                                  replacement = "")
                                    module = gsub(module, pattern = search_i,
replacement = "")
```

```
                                    get_dat$Module = module
                                    get_dat
                                  }))

  colnames(key_dat) = c("Accession", "Module")
  rownames(key_dat) = key_dat$Accession

  GS_dat = get(paste("geneTraitSignificance", i, sep = "_"))
  GS_pvalue = get(paste("GSPvalue", i, sep = "_"))

  modules_selected = unique(key_dat$Module)


  for (j in modules_selected){
    tryCatch({
      get_dat_key = key_dat[key_dat$Module == j, ]
      get_dat_MM = as.data.frame(MM_dat_genes[, which(colnames(MM_dat) == j)])

      get_dat_MMp = as.data.frame(MM_dat_pvalue[,
                                                which(colnames(MM_dat_pvalue) ==
                                                        paste(j, "_pvalue", sep =
""))])

      get_dat_MM = as.data.frame(cbind(get_dat_MM, get_dat_MMp, MM_dat_genes$Name))

      colnames(get_dat_MM) = c("MM", "MM_pvalue", "Gene")
      rownames(get_dat_MM) = rownames(MM_dat_genes)

      get_dat_summary = merge(get_dat_key, get_dat_MM, by = "row.names")
      get_dat_complete = merge(get_dat_summary, GS_dat, by.x = "Row.names", by.y =
"row.names")
      rownames(get_dat_complete) = get_dat_complete$Row.names
      get_dat_complete = get_dat_complete[, -1]
      get_dat_complete = get_dat_complete[, -1]
      get_dat_complete = merge(get_dat_complete, GS_pvalue, by = "row.names")
      rownames(get_dat_complete) = get_dat_complete$Row.names
      get_dat_complete = get_dat_complete[, -1]
      colnames(get_dat_complete) = c("Module", "MM", "MM_pvalue","Gene", "GS",
"GS_pvalue")
      assign(x = paste("GS_MM_Plot_Top20GeneKeyDrivers", i, j, sep = "_"), value =
get_dat_complete)
    }, error = function(e){cat("ERROR:", conditionMessage(e), i, j, "\n")})
  }



  df = do.call(rbind, lapply(ls(pattern = paste("GS_MM_Plot_Top20GeneKeyDrivers",
i, sep = "_")),
                                  function(x){
                                    get_dat = as.data.frame(get(x))
                                    get_dat
                                  }))

  df = merge(df, count, by = "row.names")
  row.names(df) = df$Row.names
  df = df[, -1]

  df$Colors = "white"
  df[which(df$Count_DEs > 0), "Colors"] = df$Module[which(df$Count_DEs > 0)]
  df$Label = ""
  df[which(df$Count_DEs > 0), "Label"] = df$Gene[which(df$Count_DEs > 0)]

  df$MM = abs(df$MM)
  df$GS = abs(df$GS)

  df$sum = df$MM + df$GS
  df = df[order(df$Module), ]

  figure_name = paste("GS_GM_plot_GeneKeyDrivers_Top", topx, i, sep = "_")
  pdf_name = paste(figure_name, ".wmf", sep="")
```

```
  plotfile = paste(plots_dir, pdf_name, sep=dirsep)
  win.metafile(file = plotfile,
      width = 10, height = 10,
      pointsize = 14,
      family = define_font)

  g = ggplot(df, aes(x = MM, y = GS)) +
    geom_point(shape=21, size = 3, colour = df$Module, fill = df$Colors) +
    theme_classic() +
    xlim(min(df$MM, na.rm = TRUE),1) +
    ylim(min(df$GS, na.rm = TRUE),1) +
    xlab("Module Membership") +
    ylab("Gene Trait Significance") +
    ggtitle(paste("Top 20 Key Drivers ", i,sep = "")) +
    #geom_vline(xintercept = ((1-corMM)/2)+corMM, linetype =2, col = "black") +
    #geom_hline(yintercept = ((1-corGS)/2)+corGS, linetype =2, col = "black") +
    geom_text(data = df, aes(MM, GS, label = df$Label, hjust = -0.1),
              size = rel(3)) +
    theme(axis.title.y = element_text(size = rel(1), face = "bold", color =
"black",
                                      margin = margin(t=0.5, r = 0.5, b = 0.5, l =
0.5, "cm")),
            axis.title.x = element_text(size = rel(1), face = "bold", color =
"black",
                                        margin = margin(t=0.5, r = 0.5, b = 0.5, l
= 0.5, "cm")),
            axis.text.x = element_text(size = rel(1), color = "black"),
            axis.text.y = element_text(size = rel(1), color = "black", hjust = 1),
          axis.line = element_line(colour = "black", size = 1),
          axis.ticks = element_line(colour = "black", size = 1),
          plot.margin = margin(1,1,1,1, "cm"),
          plot.title = element_text(size = rel(2), face = "bold", color = "black",
hjust = 0.5,
                                    margin = margin(0.5, 0.5, 0.5, 0.5, "cm"))) +
    facet_wrap( ~ Module, nrow = ceiling(length(unique(df$Module))/4)) +
    theme(panel.background = element_rect(fill = NA, color = "black"),
          strip.text.x = element_text(
            size = rel(1.5),
            face = "bold",
            color = "black"),
          strip.text.y = element_text(
            size = rel(1.5),
            face = "bold",
            color = "black"
          ),
          strip.background = element_rect(colour = "white", fill = "grey")
    )
  print(g)
  dev.off()

  figure_name = paste("GS_GM_plot_GeneKeyDrivers_Top", topx, i, sep = "_")
  pdf_name = paste(figure_name, ".pdf", sep="")
  plotfile = paste(plots_dir, pdf_name, sep=dirsep)
  pdf(file = plotfile,
      width = 10, height = 10,
      pointsize = 14)

   print(g)

  dev.off()

  df$Trait = i

  assign(value = df, x = paste("GS_MM_Summary_Top20KeyDrivers", i, sep = "_"))

  }, error = function(e){cat("ERROR:", conditionMessage(e), i, "\n")})
}

  KeyDrivers_Summary = do.call(rbind, lapply(ls(pattern =
"GS_MM_Summary_Top20KeyDrivers_"),
```

```
                                    function(x){
                                      get_dat = as.data.frame(get(x))
                                      get_dat$Accession = rownames(get_dat)
                                      rownames(get_dat) = NULL
                                      get_dat
                                    }))

  setname = paste("KeyDriversTop", topx, "_Summary.csv", sep = "")
  filename = paste(Rdata_dir, setname, sep = dirsep)
  write.csv(x = KeyDrivers_Summary, file = filename)

  KeyDrivers_Summary_FCs_pvalues = merge(KeyDrivers_Summary, sample_dat_complete,
by = "Accession",
                                         all.x = TRUE)

  setname = paste("KeyDriversTop", topx, "_Summary_FCs_pvalues.csv", sep = "")
  filename = paste(Rdata_dir, setname, sep = dirsep)
  write.csv(x = KeyDrivers_Summary_FCs_pvalues, file = filename)
```

```{r export key drivers to Cytoscape}
setwd(Rdata_dir)
probes = names(datExpr)

for (i in trait_vector){
  tryCatch({
    search_i = paste("_", i, "$", sep = "")

    pattern_search = as.list(ls(pattern = search_i)[grepl(ls(pattern = search_i),
                                                   pattern = "UniprotAccessions-")
== TRUE])

    key_dat = do.call(rbind, lapply(pattern_search,
                                    function(x){
                                      get_dat = as.data.frame(get(x))
                                      module = gsub(x, pattern =
"UniprotAccessions-",
                                                    replacement = "")
                                      module = gsub(module, pattern = i,
replacement = "")
                                      get_dat$Module = module
                                      get_dat
                                    }))

    colnames(key_dat) = c("Accession", "Module")
    rownames(key_dat) = key_dat$Accession

    get_dat_key = key_dat
    get_dat_merge = merge(get_dat_key, sampleGenes, by = "row.names")
        rownames(get_dat_merge) = get_dat_merge$Row.names
        get_dat_merge = get_dat_merge[, -1]
        get_dat_merge = get_dat_merge[, -1]

        inModule = is.finite(match(probes, rownames(get_dat_merge)))

        modProbes = probes[inModule]
        modGenes = get_dat_merge$Gene[match(modProbes, rownames(get_dat_merge))]

        dimnames(TOM_Cytoscape) = list(probes, probes)

        modTOM = TOM_Cytoscape[inModule, inModule]

        cyt = exportNetworkToCytoscape(modTOM,
                                       edgeFile = paste("CytoscapeInput-KeyDrivers-
edges-threshold-",
                                                        cytoscape_threshold, "-GS-
", myCorGS, "-MM-", myCorMM,
                                                        "-",
                                                        paste(i , "complete",
```

```
                                                              sep="-"), ".txt",
sep=""),
                                       nodeFile = paste("CytoscapeInput-KeyDrivers-
nodes-threshold-",
                                                        cytoscape_threshold, "-GS-
", myCorGS, "-MM-", myCorMM,
                                                        "-",
                                                        paste(i , "complete",
                                                              sep="-"), ".txt",
sep=""),
                                       weighted = TRUE,
                                       threshold = cytoscape_threshold,
                                       nodeNames = modProbes,
                                       altNodeNames = modGenes,
                                       nodeAttr = moduleColors[inModule])

        cyt = exportNetworkToCytoscape(modTOM,
                                       edgeFile = paste("CytoscapeInput-KeyDrivers-
nothreshold-edges-",
                                                        paste(i, "complete", "GS",
myCorGS, "MM", myCorMM,
                                                              sep="-"), ".txt",
sep=""),
                                       nodeFile = paste("CytoscapeInput-KeyDrivers-
nothreshold-nodes-",
                                                        paste(i, "complete", "GS",
myCorGS, "MM", myCorMM,
                                                              sep="-"), ".txt",
sep=""),
                                       weighted = TRUE,
                                       threshold = 0.0,
                                       nodeNames = modProbes,
                                       altNodeNames = modGenes,
                                       nodeAttr = moduleColors[inModule])

    modules_selected = unique(key_dat$Module)


    for (j in modules_selected){
      tryCatch({
        get_dat_key = key_dat[key_dat$Module == j, ]
        get_dat_merge = merge(get_dat_key, sampleGenes, by = "row.names")
        rownames(get_dat_merge) = get_dat_merge$Row.names
        get_dat_merge = get_dat_merge[, -1]
        get_dat_merge = get_dat_merge[, -1]

        inModule = is.finite(match(probes, rownames(get_dat_merge)))

        modProbes = probes[inModule]
        modGenes = get_dat_merge$Gene[match(modProbes, rownames(get_dat_merge))]

        dimnames(TOM_Cytoscape) = list(probes, probes)

        modTOM = TOM_Cytoscape[inModule, inModule]

        cyt = exportNetworkToCytoscape(modTOM,
                                       edgeFile = paste("CytoscapeInput-KeyDrivers-
edges-threshold-",
                                                        cytoscape_threshold, "-GS-
", myCorGS, "-MM-", myCorMM,
                                                        "-",
                                                        paste(i, j,
                                                              sep="-"), ".txt",
sep=""),
                                       nodeFile = paste("CytoscapeInput-KeyDrivers-
nodes-threshold-",
                                                        cytoscape_threshold, "-GS-
", myCorGS, "-MM-", myCorMM,
                                                        "-",
                                                        paste(i, j,
```

```
                                                              sep="-"), ".txt",
sep=""),
                                       weighted = TRUE,
                                       threshold = cytoscape_threshold,
                                       nodeNames = modProbes,
                                       altNodeNames = modGenes,
                                       nodeAttr = moduleColors[inModule])

        cyt = exportNetworkToCytoscape(modTOM,
                                       edgeFile = paste("CytoscapeInput-KeyDrivers-
nothreshold-edges-",
                                                        "GS-", myCorGS, "-MM-",
myCorMM, "-",
                                                        paste(i, j,
                                                              sep="-"), ".txt",
sep=""),
                                       nodeFile = paste("CytoscapeInput-KeyDrivers-
nothreshold-nodes-",
                                                        "GS-", myCorGS, "-MM-",
myCorMM, "-",
                                                        paste(i, j,
                                                              sep="-"), ".txt",
sep=""),
                                       weighted = TRUE,
                                       threshold = 0.0,
                                       nodeNames = modProbes,
                                       altNodeNames = modGenes,
                                       nodeAttr = moduleColors[inModule])

      }, error = function(e){cat("ERROR:", conditionMessage(e), i, j, "\n")})
    }
  }, error = function(e){cat("ERROR:", conditionMessage(e), i, "\n")})
}
```

```{r export top 20 key drivers to Cytoscape}
setwd(Rdata_dir)
probes = names(datExpr)

for (i in trait_vector){
  tryCatch({
    search_i = paste("_", i, "$", sep = "")

    pattern_search = as.list(ls(pattern = search_i)[grepl(ls(pattern = search_i),
                                                   pattern =
"UniprotAccessionsTop20-") == TRUE])


    key_dat = do.call(rbind, lapply(pattern_search,
                                    function(x){
                                      get_dat = as.data.frame(get(x))
                                      module = gsub(x, pattern =
"UniprotAccessionsTop20-",
                                                    replacement = "")
                                      module = gsub(module, pattern = i,
replacement = "")
                                      get_dat$Module = module
                                      get_dat
                                    }))

    colnames(key_dat) = c("Accession", "Module")
    rownames(key_dat) = key_dat$Accession

    modules_selected = unique(key_dat$Module)


    for (j in modules_selected){
      tryCatch({
        get_dat_key = key_dat[key_dat$Module == j, ]
        get_dat_merge = merge(get_dat_key, sampleGenes, by = "row.names")
```

```
        rownames(get_dat_merge) = get_dat_merge$Row.names
        get_dat_merge = get_dat_merge[, -1]
        get_dat_merge = get_dat_merge[, -1]

        inModule = is.finite(match(probes, rownames(get_dat_merge)))

        modProbes = probes[inModule]
        modGenes = get_dat_merge$Gene[match(modProbes, rownames(get_dat_merge))]

        dimnames(TOM_Cytoscape) = list(probes, probes)

        modTOM = TOM_Cytoscape[inModule, inModule]

        cyt = exportNetworkToCytoscape(modTOM,
                                       edgeFile = paste("CytoscapeInput-Top", topx,
"-KeyDrivers-nothreshold-edges-",
                                                        paste(i, j,
                                                              sep="-"), ".txt",
sep=""),
                                       nodeFile = paste("CytoscapeInput-Top", topx,
"-KeyDrivers-nothreshold-nodes-",
                                                        paste(i, j,
                                                              sep="-"), ".txt",
sep=""),
                                       weighted = TRUE,
                                       threshold = 0,
                                       nodeNames = modProbes,
                                       altNodeNames = modGenes,
                                       nodeAttr = moduleColors[inModule])

      }, error = function(e){cat("ERROR:", conditionMessage(e), i, j, "\n")})
    }
  }, error = function(e){cat("ERROR:", conditionMessage(e), i, "\n")})
}
```
```
```
